# Synthesis and Reactions of 3-Halogenated 2-CF_3_-Indoles

**DOI:** 10.3390/molecules27248822

**Published:** 2022-12-12

**Authors:** Vasiliy M. Muzalevskiy, Zoia A. Sizova, Valentine G. Nenajdenko

**Affiliations:** Department of Chemistry, Lomonosov Moscow State University, 119899 Moscow, Russia

**Keywords:** CF_3_-group, halogenation, nucleophilic substitution, indole, fluorine

## Abstract

Halogenation of 2-trifluoromethylindole afforded 3-chloro-, 3-bromo- and 3-iodo derivatives in up to 98% yield. Methyl-, benzyl- and tosyl-groups can be installed at the nitrogen atom of prepared indoles in high yields by base catalyzed reaction with the corresponding alkylating (sulfonylating) reagents. A high synthetic utility of the prepared haloindoles in the reaction with various nucleophilies was shown. The reaction with 4-methylthiophenol and copper cyanide afforded the corresponding sulfides and nitriles in high yield. Palladium catalyzed cross-coupling with phenyl boronic acid and phenylacetylene gave the corresponding 3-phenyl-2-CF_3_-indoles and acetylenic derivatives in 72–98% yield.

## 1. Introduction

Indole is one of the most important heterocycles in organic chemistry. Since its discovery in 1866 by Bayer [1], this molecule attracted a lot of attention from chemists [2,3,4,5,6,7]. Many useful, practical methods for the preparation of indoles have been developed, and some of them became name reactions (Fischer, Madelung, Hemetsberger, etc.) [8,9]. An indole is a “privileged structure” in drug discovery [10]. This scaffold can be found in many pharmaceuticals and natural products [11] and has been shown to provide various biological activities, including anti-inflammatory, anti-HIV, antitubercular, antimalarial, anticonvulsant, antidiabetic, antihypertensive, analgesics, antidepressant, anticancer, antioxidant, antifungal, and antimicrobial, etc. [12]. Eight derivatives of indole can be found in the list of 200 best-selling drugs in 2021. Tagrisso ($5.015 Bn), Trikafta ($5.697 Bn), Ofev ($2.743 Bn), Leuprorelin ($0.865 Bn), Alecensa ($1.459 Bn), Zoladex ($ 0948 Bn), Sutent ($0.673 Bn), and Decapeptyl ($0.506 Bn) were sold for about $18 billion totally in 2021 worldwide [13].

On the other hand, fluorinated organic compounds have been the object of intensive investigations for the past few decades owing to their unique complex of physicochemical and biological properties [14,15,16,17,18,19,20,21,22,23,24,25]. Thus, about 20% (more than 300 compounds) of currently used drugs [26,27,28,29,30,31,32,33] contain at least one fluorine atom [34]. In 2021, approximately 33% of “small-molecule” drugs approved by the FDA are fluorinated compounds [35]. At the same time, about 59% of all small-molecule drugs have in the structure a nitrogen heterocyclic motif [11]. Four out of every five (82%) small-molecule drugs approved by the FDA in 2021 are heterocyclic compounds [35]. There is no doubt that development of novel, effective pathways to fluorinated nitrogen heterocycles is an important task [36,37,38,39,40,41,42,43].

Methods of direct fluorination are not always selective and suffer from quite expensive fluorinating reagents. The building block strategy, using simple fluorinated compounds for the assembling of more complex structures, is a fruitful alternative to the methods of direct fluorination. Our group is deeply involved into development of novel fluorinated building blocks. Recently we have proposed the synthesis of α-CF_3_-β-aryl enamines [44,45,46], which have been shown to be potent CF_3_-building blocks for the synthesis of various fluorinated compounds. Using α-CF_3_-β-aryl enamines, we have elaborated convenient approaches towards α-CF_3_-phenethylamines [47], CF_3_-enones [48,49,50], CF_3_-β-carbolines [51], 2-CF_3_-3-arylindoles [51], 2-CF_3_-3-benzylindoles [52] and unsubstituted 2-CF_3_-indoles [53,54]. We have also examined several electrophilic reagents for modification of 2-CF_3_-indoles at C-3 atom [54]. 2-CF_3_-Indoles are perspective compounds for drug design. Thus, derivatives of these indoles have demonstrated properties of selective COX-2 inhibitors [55], anti-inflammatory and neuroprotective actions [56], antiproliferative [57], antineoplastic properties [58] and antifungal properties [59]. In this article we continue investigation of synthetic potential of 2-CF_3_-indoles to study preparation of 3-halogenated derivatives and their subsequent modification.

## 2. Results

**Scheme 1:**
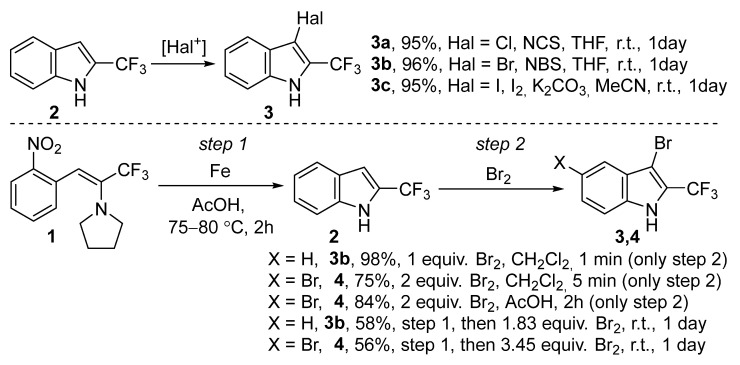
Halogenation of 2-CF_3_-indoles.

Thinking about further transformations of 2-CF_3_-indoles, we found surprisingly, that halogenation of these indoles has not been thoroughly and systematically investigated. To the best of our knowledge, only the iodination of 2-CF_3_-indole using *N*-iodosuccinimide was reported so far [60]. 3-Chloro-2-trifluromethylindole and its *N*-methyl derivative were reported but synthesized via the fluoroalkylation processes [61,62]. To fill this gap, we maintained the reaction of indole **2** with NCS, NBS, Br_2_ and iodine. This approach afforded 3-chloro-, 3-bromo- and 3-iodoindoles **3a–3c** in high yields (Scheme 1). In spite of the presence of electron-withdrawing trifluoromethyl group, 2-CF_3_-indole is a very reactive molecule towards electrophiles. All mentioned halogenation reactions were performed without the addition of Lewis acids. Moreover, 3-bromoindole **3b** can be easily brominated in CH_2_Cl_2_ at 5-position by the addition of second equivalent of bromine to give dibromoindole **4** in 75% yield in a few minutes. Bromination of **2** with two equivalents of Br_2_ in AcOH proceeded very slowly (over a few hours) to give indole **4** in higher yield. Such a result can be explained by lower selectivity of bromination in CH_2_Cl_2_, leading for deeper bromination of the indole core. Alternatively, bromoindoles **3b** and **4** were prepared by one-pot synthesis starting from enamine **1**. For that purpose, a solution of bromine in AcOH was added to the reaction mixture obtained after the reduction step. Mono- and di-bromo derivatives were prepared in 58% and 56% yields calculating on two steps (Scheme 1).

**Scheme 2:**
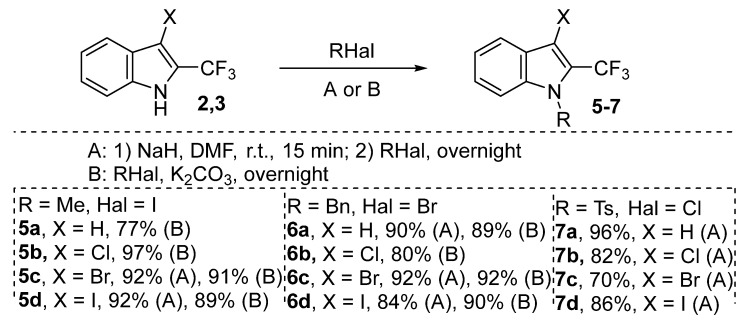
Modification of 2-CF_3_-indoles at NH moiety.

Aryl halides are valuable starting materials for reactions with nucleophiles as well as for metal-catalyzed cross-coupling reactions. Having prepared a set of 3-halogenated 2-CF_3_-indoles, we decided to investigate their reactivity. In order to expand a set of substrates, *N*-methyl, *N*-benzyl and *N*-tosyl derivatives of indoles **5–7** were prepared as well. Thus, treatment of indoles **2**, **3a**, **3b**, **3c** with NaH in DMF followed by the reaction with MeI, BnBr, TsCl afforded *N*-methyl, *N*-benzyl and N-tosyl indoles **5–7** in high yield (conditions A). Alternatively, K_2_CO_3_ was used as a base for alkylation with MeI and BnBr (conditions B) (Scheme 2).

**Scheme 3:**
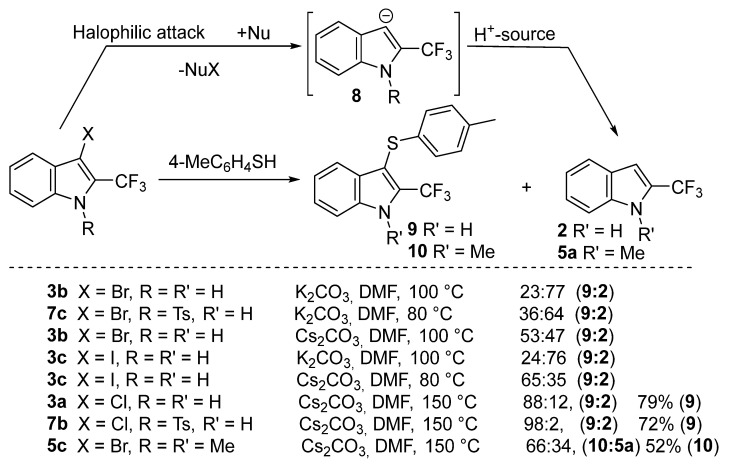
Reactions of 3-halogeno-2-CF_3_-indoles with 4-methylthiophenol.

Next, the reaction of prepared 3-halogeno-2-CF_3_-indoles with 4-methylthiophenol was investigated. It was found that reaction with this potent nucleophile is a very sensitive to the nature of both halogen and a base. Thus, reaction of bromo- and iodoindoles **3b**,**3c** led to the mixture of desired substitution product **9** and indole **2**, which is a result of the reduction. When K_2_CO_3_ was used as a base, the share of indole **9** was about 23–24%, while in the case of Cs_2_CO_3_, indole **9** became major product of the reaction (53–65%). Slightly better selectivity was observed for the reaction with *N*-tosylated bromoindole **7c** (36% compared to 23%). It should be noted that the tosyl group did not survive in the reaction conditions to give deprotected NH-indoles. Dramatically better selectivity was observed for chloroindole **3a**, giving substitution product **9** in 88:12 ratio with indole **2**. Ultimate selectivity was achieved using chloroindole **7b**. We found that reaction of **7b** with 4-methylthiophenol at elevated temperatures led to thioether **9** in 72% yield. Substitution reaction with **7b** was accompanied by detosylation to afford indole **9** without tosyl group at nitrogen atom. Eventually, the reaction of N-methyl-3-bromoindol **5c** gave indole **10** in ratio 66:34 with indole **5a** in 85% total yield (Scheme 3). It should be noted that chloroindoles **3a**,**7b** are less reactive than bromo- and iodoindoles **3b**,**3c**,**7c**. As a result, higher temperatures (150 °C instead of 80–100 °C) were used for full conversion. By the similar reason, reaction of *N*-methylbromoindole **5c** was also performed at 150 °C.

The formation of indoles **2** and **5a** is a result of a two-step process which is initiated by a halophilic attack to bromo- and iodoindoles by a nucleophile to form anion **8**. Subsequent protonation of carbanion **8** led to indoles **2** and **5a** having a hydrogen atom at 3-position (Scheme 3). In contrast, chloroindoles are less prone to be objects for halophilic attack due to the higher energy of C-Cl bond and nucleophilic substitution is preferable. However, only very potent nucleophiles, like thiophenols, can participate in the reaction.

**Scheme 4:**
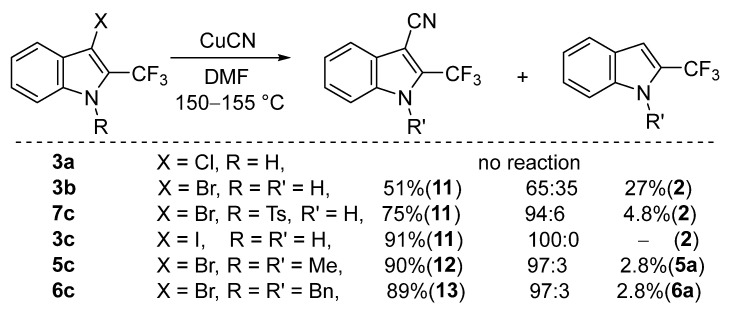
Reactions of 3-halogeno-2-CF_3_-indoles with CuCN.

Less complicated results were obtained in the reactions with CuCN (Rosenmund von Braun reaction [63]). It is not surprising, because this reaction does not occur via the S_N_Ar mechanism [64,65]. The reaction of **3b** with CuCN led to the mixture of nitrile **11** with indole **2** in 65:35 ratio. *N*-Tosylated bromoindole **7c** afforded a 96:4 mixture of **11** and **2**. One hundred percent chemoselectivity was achieved using iodoindole **3c**. The corresponding nitrile **11** was isolated in 91% yield. Selectivity of the reaction of bromoindoles with CuCN raised dramatically after installation of methyl and benzyl groups at the nitrogen atom. Both N-methyl- and N-benzylindoles provided high yields of cyanation products (near 90%) **12** and **13**. However, chloroindole **3a** does not participate in the reaction (Scheme 4).

**Scheme 5:**
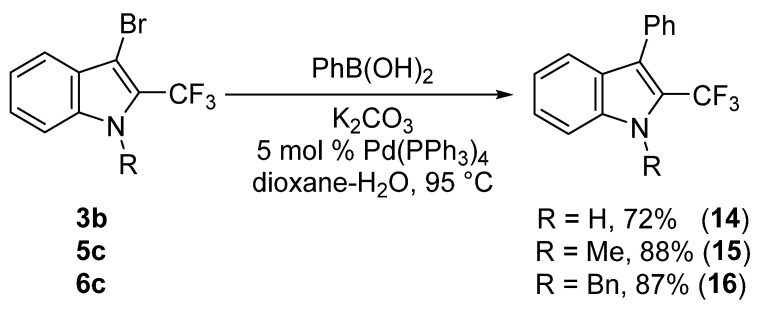
Reactions of 3-bromo-2-CF_3_-indoles with phenyl boronic acid.

Next, we investigated palladium-catalyzed cross-coupling reactions of bromo- and iodoindoles with phenyl boronic acid (Suzuki reaction) and phenylacetylene (Snogashira reaction) in standard conditions for these reactions. We found that reactions of bromoindoles **3b**,**5c**,**6c** with phenyl boronic acid proceeded very smoothly under catalysis with Pd(PPh_3_)_4_ in dioxane-water as a solvent using K_2_CO_3_ as a base. The corresponding 3-phenylindoles **14–16** were isolated in high yield while the reduction products **2**,**5a**,**6a** were formed in traces amounts (Scheme 5).

**Scheme 6:**
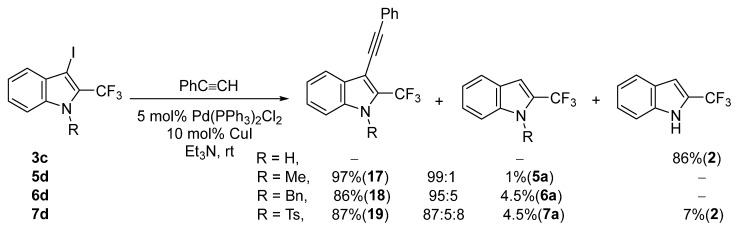
Reactions of 3-bromo-2-CF_3_-indoles with phenylacetylene.

The Sonogashira reaction was carried out using (Pd(PPh_3_)_2_Cl_2_ and CuI as catalyst and Et_3_N as a solvent. We found that iodoindoles react easily with phenylacetylene at room temperature, but the result of the reaction depends on the substituent at the nitrogen atom. Thus, the reaction of N-unsubstituted iodoindole **3c** only afforded the reduction product **2** in an 86% yield. The reaction of N-methyliodoindole afforded acetylene **17** in almost quantitative yield. In a similar manner, N-benzyliodoindole led to acetylene **18** in an 86% yield. In the case of *N*-tosyliodoindole, the desired indole **19** was isolated in 87% yield and the share of by-products **7a** and **2** was about below 13% (Scheme 6).

## 3. Materials and Methods

General remarks. ^1^H, ^13^C and ^19^F NMR spectra (see Supplementary Materials Figures S1–S82) were recorded on a Bruker AVANCE 400 MHz spectrometer(Bruker, Billerica, MA, USA) in CD_3_CN and CDCl_3_ at 400.1, 100.6 and 376.5 MHz, respectively. Chemical shifts (*δ*) in ppm are reported with the use of the residual CHD_2_CN and chloroform signals (1.94, 7.25 for ^1^H and 1.30, 77.0 for ^13^C) as an internal reference. The ^19^F chemical shifts were referenced to C_6_F_6_ (−162.9 ppm). The coupling constants (*J*) are given in Hertz (Hz). HRMS spectra were measured at MicroTof Bruker Daltonics instrument. TLC analysis was performed on “Merck 60 F_254_” plates (Merck, Darmstadt, Germany). Column chromatography was performed on silica gel “Macherey-Nagel 0.063–0.2 nm. All reagents were of reagent grade and were used as such or were distilled prior to use. Enamine **1** [44] and indole **2** [54] were prepared as reported previously. Melting points were determined on an Electrothermal 9100 apparatus.

**Synthesis of 3-chloro-2-(trifluoromethyl)-1*H*-indole** (**3a**). A 50 mL one-necked round-bottomed flask was charged with aa 2-(trifluoromethyl)-1*H*-indole (**2**) (0.990 g, 5.35 mmol), THF (20 mL) and *N*-chlorosuccinimide (0.790 g, 5.90 mmol). The reaction mixture was stirred for 1 min to form a clear solution and 4 drops of TMSCl (~0.020–0.030 g) were added to initiate the reaction. The reaction mixture was left at room temperature for 1 day. After the completion of the reaction (^19^F NMR control), THF was evaporated in vacuo. The residue was dissolved in CH_2_Cl_2_ (20 mL) and washed with a saturated solution of Na_2_SO_3_ (5 mL) followed by water (20 mL). The organic phase was dried over Na_2_SO_4_. Volatiles were evaporated in vacuo, and the residue formed was purified by passing it through a short silica gel pad using hexane-CH_2_Cl_2_ mixture (3:1) as an eluent. Evaporation of the solvents afforded **3-chloro-2-(trifluoromethyl)-1*H*-indole** (**3a**) as beige crystals, m.p. 49–50 °C, yield 1.120 g (95%). ^1^H NMR (CDCl_3_, 400.1 MHz): δ 8.34 (br.s, 1H), 7.71 (d, 1H, ^3^*J* = 8.1 Hz), 7.39 (d, 2H, ^4^*J* = 3.8 Hz), 7.32–7.25 (m, 1H). ^13^C{^1^H} NMR (CDCl_3_, 100.6 MHz): δ 133.9, 125.9, 125.4, 121.7, 120.8 (q, ^2^*J*_CF_ = 38.1 Hz), 120.7 (q, ^1^*J*_CF_ = 268.7 Hz), 119.6, 112.0, 108.1 (q, ^3^*J*_CF_ = 3.2 Hz). ^19^F NMR (CDCl_3_, 376.5 MHz): δ −60.9 (s, 3F). HRMS (ESI-TOF): *m*/*z* [M-H]^−^ Calcd for C_9_H_4_ClF_3_N^−^: 217.9990; found: 217.9995. NMR data of indole **3a** are in agreement with those in the literature [61].

**Synthesis of 3-bromo-2-(trifluoromethyl)-1*H*-indole (3b) by the reaction with NBS**. A 50 mL one-necked round-bottomed flask was charged with 2-(trifluoromethyl)-1*H*-indole (**2**) (1.004 g, 5.43 mmol), THF (20 mL) and *N*-bromosuccinimide (1.014 g, 5.70 mmol). The reaction mixture was stirred for 1 min to form a clear solution and left at room temperature for 1 day. After the completion of the reaction (^19^F NMR control) THF was evaporated in vacuo, the residue was dissolved in CH_2_Cl_2_ (20 mL) and washed with a saturated solution of Na_2_SO_3_ (5 mL), followed by water (20 mL). The organic phase was dried over Na_2_SO_4_. Volatiles were evaporated in vacuo, the residue formed was purified by passing it through a short silica gel pad using hexane-CH_2_Cl_2_ mixture (3:1) as an eluent. Evaporation of the solvents afforded **3-bromo-2-(trifluoromethyl)-1*H*-indole** (**3b**) as a pale green–brown solid, m.p. 39–41 °C, yield 1.377 g (96%). ^1^H NMR (CDCl_3_, 400.1 MHz): δ 8.50 (br.s, 1H), 7.67 (d, 1H, ^3^*J* = 8.1 Hz), 7.43–7.35 (m, 2H), 7.34–7.26 (m, 1H). ^13^C{^1^H} NMR (CDCl_3_, 100.6 MHz): δ 134.4, 127.1, 125.9, 122.7 (q, ^2^*J*_CF_ = 37.9 Hz), 121.9, 120.8 (q, ^1^*J*_CF_ = 269.1 Hz), 120.5, 111.9, 93.3 (q, ^3^*J*_CF_ = 3.4 Hz). ^19^F NMR (CDCl_3_, 376.5 MHz): δ −60.8 (s, 3F). HRMS (ESI-TOF): *m*/*z* [M-H]^−^ Calcd for C_9_H_4_BrF_3_N^−^: 261.9485; found: 261.9482. **Synthesis of 3-bromo-2-(trifluoromethyl)-1*H*-indole (3b) by the reaction with Br_2_**. A 25 mL one-necked round-bottomed flask was charged with 2-(trifluoromethyl)-1*H*-indole (**2**) (1.085 g, 5.86 mmol), CH_2_Cl_2_ (10 mL) and solution of Br_2_ (0.950 g, 5.94 mmol) in CH_2_Cl_2_ (1.5 mL) was added dropwise for 1 min. The reaction mixture was stirred for 1 min and washed with a saturated solution of Na_2_SO_3_ (5 mL) followed by water (20 mL). The organic phase was dried over Na_2_SO_4_. Volatiles were evaporated in vacuo, the residue formed was purified by passing it through a short silica gel pad using hexane-CH_2_Cl_2_ mixture (3:1) as an eluent. Evaporation of the solvents afforded **3-bromo-2-(trifluoromethyl)-1*H*-indole** (**3b**) as a pale green–brown solid, m.p. 39–41 °C, yield 1.51 g (98%).

**Synthesis of 3-iodo-2-(trifluoromethyl)-1*H*-indole** (**3c**). A 20 mL one-necked round-bottomed flask was charged with 2-(trifluoromethyl)-1*H*-indole (**2**) (0.555 g, 3.0 mmol), MeCN (6 mL), K_2_CO_3_ (0.420 g, 3.043 mmol) and I_2_ (1.230 g, 4.843 mmol). The reaction mixture was stirred at room temperature for 1 day. After the completion of the reaction (^19^F NMR control), a saturated solution of Na_2_SO_3_ (10 mL) was added to quench the reaction. The product was extracted by CH_2_Cl_2_ (3 × 20 mL), the organic phase was washed by water (20 mL) and dried over Na_2_SO_4_. Volatiles were evaporated in vacuo, the residue formed was purified by passing it through a short silica gel pad using a hexane-CH_2_Cl_2_ mixture (3:1) as an eluent. Evaporation of the solvents afforded **3-iodo-2-(trifluoromethyl)-1*H*-indole** (**3c**) as a pale yellow solid, m.p. 53–55 °C, yield 0.884 g (95%). ^1^H NMR (CDCl_3_, 400.1 MHz): δ 8.68 (br.s, 1H), 7.55 (d, 1H, ^3^*J* = 8.0 Hz), 7.43–7.35 (m, 2H), 7.33–7.26 (m, 1H). ^13^C{^1^H} NMR (CDCl_3_, 100.6 MHz): δ 135.0, 130.4, 126.4 (q, ^2^*J*_CF_ = 37.2 Hz), 125.9, 122.6, 122.0, 120.8 (q, ^1^*J*_CF_ = 269.6 Hz), 111.9, 59.9 (q, ^3^*J*_CF_ = 3.4 Hz). ^19^F NMR (CDCl_3_, 376.5 MHz): δ −60.6 (s, 3F). HRMS (ESI-TOF): *m*/*z* [M-H]^−^ Calcd for C_9_H_4_IF_3_N^-^: 309.9346; found: 309.9346. NMR data of indole **3c** are in agreement with those in the literature [59].

**Synthesis of 3,5-dibromo-2-(trifluoromethyl)-1*H*-indole** (**4**). A 10 mL one-necked round-bottomed flask was charged with 2-(trifluoromethyl)-1*H*-indole (**2**) (0.098 g, 0.53 mmol), CH_2_Cl_2_ (1 mL) and 1M solution of Br_2_ (1.07 mL, 1.07 mmol) in CH_2_Cl_2_ was added dropwise for 1 min. The reaction mixture was stirred for 5 min and then CH_2_Cl_2_ (10 mL) was added. The solution formed was washed by saturated solution of Na_2_SO_3_ (1 mL) followed by water (20 mL). The organic phase was dried over Na_2_SO_4_. Volatiles were evaporated in vacuo, the residue formed was purified by passing it through a short silica gel pad using a hexane-CH_2_Cl_2_ mixture (3:1) as an eluent. The evaporation of the solvents afforded **3,5-dibromo-2-(trifluoromethyl)-1*H*-indole** (**4**) as a pale yellow solid, m.p. 57–59 °C, yield 0.175 g (75%). ^1^H NMR (CDCl_3_, 400.1 MHz): δ 8.59 (br.s, 1H), 7.78 (s, 1H), 7.44 (dd, 1H, ^3^*J* = 8.7 Hz, ^4^*J* = 1.4 Hz), 7.28 (d, 1H, ^3^*J* = 8.7 Hz). ^13^C{^1^H} NMR (CDCl_3_, 100.6 MHz): δ 133.0, 129.1, 128.7, 123.9 (q, ^2^*J*_CF_ = 37.9 Hz), 123.2, 120.4 (q, ^1^*J*_CF_ = 269.5 Hz), 115.2, 113.5, 92.4 (q, ^3^*J*_CF_ = 3.0 Hz). ^19^F NMR (CDCl_3_, 376.5 MHz): δ −61.2 (s, 3F). HRMS (ESI-TOF): *m*/*z* [M-H]^−^ Calcd for C_9_H_3_Br_2_F_3_N^−^: 339.8590; found: 339.8581.

**One-pot synthesis of 3-bromo-2-(trifluoromethyl)-1*H*-indole (3b) from enamine 1**. A 50 mL one-necked round-bottomed flask was charged with enamine (**1**) (1.52 g, 5.31 mmol), acetic acid (25 mL) and water (5 mL). The reaction mixture was heated to 60 °C and Fe (1.19 g, 21.24 mmol) was added. The reaction mixture was stirred at 80 °C for 2 h. The reaction mixture was cooled to room temperature and 1M solution of Br_2_ in acetic acid (8 mL, 8 mmol) was added. ^19^F NMR analysis of the reaction mixture revealed the rest amounts of indole **2**, which was converted into desired indole **3a** by addition of 1M solution of Br_2_ in acetic acid (1.72 mL). The reaction mixture was diluted with water (100 mL), the reaction product was extracted by CH_2_Cl_2_ (3 × 20 mL), and the organic phase was washed by water (20 mL) and dried over Na_2_SO_4_. Volatiles were evaporated in vacuo, the residue formed was purified by passing through a short silica gel pad using hexane-CH_2_Cl_2_ mixture (3:1) as an eluent. Evaporation of the solvents afforded **3-bromo-2-(trifluoromethyl)-1*H*-indole** (**3b**) as a pale green-brown solid, m.p. 39–41 °C, yield 0.814 g (58%).

**One pot synthesis of 3,5-dibromo-2-(trifluoromethyl)-1*H*-indole (4) from enamine 1**. Similary to the one pot synthesis of **3-bromo-2-(trifluoromethyl)-1*H*-indole** (**3b**) the reaction was repeated in 0.417 mmol scale using 1.44 mL of 1M solution of Br_2_ in acetic acid (3.45 equiv.). Pale yellow solid, m.p. 57–59 °C, yield 0.079 g (56%).

**Modification of indoles at nitrogen atom using NaH and alkylating reagent (general procedure A)**. A 4 mL vial with a screw cap (for 1 mmol scale; 12 mL 2–3 mmol scale, 20 mL for 5–6 mmol scale) was charged with corresponding indole (1.0 mmol), DMF (1 mL) and NaH (0.090 g, 1.5 mmol, 60% suspension in mineral oil) was added at cooling with a cold-water bath. The reaction mixture was stirred at room temperature for 15 min and then corresponding alkylating reagent (MeI, BnBr, TsCl; 1.2 mmol) was added at cooling with a cold-water bath. The reaction mixture was stirred at room temperature overnight and then was broken by 0.1M HCl (5 mL). The product was extracted by CH_2_Cl_2_ (3 × 10 mL), the organic phase was washed by water (2 × 10 mL) and brine (10 mL) and dried over Na_2_SO_4_. Volatiles were evaporated in vacuo, the residue formed was purified by passing through a short silica gel pad using hexane-CH_2_Cl_2_ mixture (3:1) as an eluent. Evaporation of the solvents afforded the corresponding indole.

**Modification of indoles at nitrogen atom using K_2_CO_3_ and alkylating reagent (general procedure B)**. A 4 mL vial with a screw cap was charged with the corresponding indole (1.0 mmol), DMF (1 mL), K_2_CO_3_ (0.207 g, 1.5 mmol) and corresponding alkylating reagent (MeI, BnBr, 1.2 mmol). The reaction mixture was stirred at room temperature overnight and then poured into 0.5 M HCl (10 mL). The product was extracted by CH_2_Cl_2_ (3 × 10 mL), the organic phase was washed by water (2 × 10 mL), brine (10 mL) and dried over Na_2_SO_4_. Volatiles were evaporated in vacuo, the residue formed was purified by passing through a short silica gel pad using hexane-CH_2_Cl_2_ mixture (3:1) as an eluent. Evaporation of the solvents afforded the corresponding indole.

**1-Methyl-2-(trifluoromethyl)-1*H*-indole** (**5a**). Obtained from indole **2** (0.187 g, 1.011 mmol) by procedure (B). White crystals; m.p. 32–34 °C, yield 0.155 g (77%). ^1^H NMR (CDCl_3_, 400.1 MHz): δ 7.72 (d, 1H, ^3^*J* = 8.0 Hz), 7.44–7.38 (m, 2H), 7.24 (ddd, 1H, ^3^*J* = 8.0 Hz, ^3^*J* = 4.8 Hz, ^4^*J* = 3.1 Hz), 6.99 (s, 1H), 3.86 (s, 3H). ^13^C{^1^H} NMR (CDCl_3_, 100.6 MHz): δ 138.4 (q, ^4^*J_CF_* = 1.0 Hz), 127.1 (q, ^2^*J*_CF_ = 37.1 Hz), 125.6, 124.3, 122.2, 121.5 (q, ^1^*J*_CF_ = 267.9 Hz), 120.6, 109.8, 104.2 (q, ^3^*J*_CF_ = 3.1 Hz), 30.6 (q, ^3^*J*_CF_ = 1.8 Hz). ^19^F NMR (CDCl_3_, 376.5 MHz): δ −60.6 (s, 3F). NMR data of indole **5a** are in agreement with those in the literature [66].

**3-Chloro-1-methyl-2-(trifluoromethyl)-1*H*-indole** (**5b**). Obtained from indole **3a** (0.231 g, 1.05 mmol) by procedure (B). Yellow oil; yield 0.238 g (97%). ^1^H NMR (CDCl_3_, 400.1 MHz): δ 7.70 (d, 1H, ^3^*J* = 8.2 Hz), 7.44–7.38 (m, 1H), 7.35 (d, 1H, ^3^*J* = 8.2 Hz), 7.25 (ddd, 1H, ^3^*J* = 8.2 Hz, ^3^*J* = 6.9 Hz, ^4^*J* = 1.0 Hz), 3.83 (s, 3H). ^13^C{^1^H} NMR (CDCl_3_, 100.6 MHz): δ 136.6 (q, ^4^*J_CF_* = 1.0 Hz), 125.7, 124.5, 121.6 (q, ^2^*J*_CF_ = 35.8 Hz), 121.3 (q, ^1^*J*_CF_ = 269.9 Hz), 121.2, 119.8, 109.9, 108.0 (q, ^3^*J*_CF_ = 3.0 Hz), 31.3 (q, ^3^*J*_CF_ = 2.6 Hz). ^19^F NMR (CDCl_3_, 376.5 MHz): δ −57.9 (s, 3F). HRMS (ESI-TOF): *m*/*z* [M + H]^+^ Calcd for C_10_H_8_ClF_3_N^+^: 234.0292; found: 234.1341. NMR data of indole **5b** are in agreement with those in the literature [62].

**3-Bromo-1-methyl-2-(trifluoromethyl)-1*H*-indole** (**5c**). Obtained from indole **3b** (0.535 g, 2.03 mmol (A); 0.0524, 0.198 mmol (B)) by procedure (A) and (B). Pale yellow oil; yield 0.517 g (92%, A), 0.050 g (91%, B). ^1^H NMR (CDCl_3_, 400.1 MHz): δ 7.67 (d, 1H, ^3^*J* = 8.1 Hz), 7.45–7.38 (m, 1H), 7.34 (d, 1H, ^3^*J* = 8.4 Hz), 7.25 (ddd, 1H, ^3^*J* = 7.9 Hz, ^3^*J* = 7.0 Hz, ^4^*J* = 0.9 Hz), 3.84 (s, 3H). ^13^C{^1^H} NMR (CDCl_3_, 100.6 MHz): δ 137.2, 126.1, 125.7, 123.3 (q, ^2^*J*_CF_ = 35.8 Hz), 121.26 (q, ^1^*J*_CF_ = 270.2 Hz), 121.33, 120.9, 109.9, 93.1 (q, ^3^*J*_CF_ = 3.3 Hz), 31.5 (q, ^3^*J*_CF_ = 2.7 Hz). ^19^F NMR (CDCl_3_, 376.5 MHz): δ −57.5 (d, 3F, ^4^*J* = 0.5 Hz). HRMS (ESI-TOF): *m*/*z* [M + H]^+^ Calcd for C_10_H_8_BrF_3_N^+^: 277.9787; found: 277.9340.

**3-Iodo-1-methyl-2-(trifluoromethyl)-1*H*-indole** (**5d**). Obtained from indole **3c** (0.185 g, 0.595 mmol (A); 0.029, 0.093 mmol (B)) by procedure (A) and (B). Pale yellow oil; yield 0.178 g (92%, A), 0.0269 g (89%, B). ^1^H NMR (CDCl_3_, 400.1 MHz): δ 7.58 (d, 1H, ^3^*J* = 8.1 Hz), 7.46–7.40 (m, 1H), 7.34–7.26 (m, 2H), 3.88 (s, 3H). ^13^C{^1^H} NMR (CDCl_3_, 100.6 MHz): δ 138.0 (q, ^4^*J_CF_* = 1.0 Hz), 129.5, 126.6 (q, ^2^*J*_CF_ = 35.3 Hz), 125.6, 123.3, 121.5, 121.1 (q, ^1^*J*_CF_ = 270.7 Hz), 109.9, 59.4 (q, ^3^*J*_CF_ = 3.5 Hz), 31.8 (q, ^3^*J*_CF_ = 2.7 Hz). ^19^F NMR (CDCl_3_, 376.5 MHz): δ −57.0 (d, 3F). HRMS (ESI-TOF): *m*/*z* [M + H]^+^ Calcd for C_10_H_8_F_3_IN^+^: 325.9648; found: 325.9655.

**1-Benzyl-2-(trifluoromethyl)-1*H*-indole** (**6a**). Obtained from indole **2** (0.377 g, 2.038 mmol (A); 0.070, 0.378 mmol (B)) by procedure (A) and (B). Colorless oil; yield 0.5052 g (90%, A), 0.0925 g (89%, B). ^1^H NMR (CDCl_3_, 400.1 MHz): δ 7.79 (d, 1H, ^3^*J* = 7.9 Hz), 7.37–7.23 (m, 6H), 7.12 (s, 1H), 7.11–7.04 (m, 2H), 5.54 (s, 2H). ^13^C{^1^H} NMR (CDCl_3_, 100.6 MHz): δ 138.2 (q, ^4^*J_CF_* = 1.0 Hz), 136.8, 128.7, 127.5, 127.3 (q, ^2^*J*_CF_ = 37.3 Hz), 124.7, 122.3, 121.5 (q, ^1^*J*_CF_ = 268.3 Hz), 121.0, 110.8, 105.0 (q, ^3^*J*_CF_ = 1.9 Hz), 48.1 (q, ^3^*J*_CF_ = 1.9 Hz). ^19^F NMR (CDCl_3_, 376.5 MHz): δ −59.9 (s, 3F). HRMS (ESI-TOF): *m*/*z* [M + H]^+^ Calcd for C_16_H_13_F_3_N^+^: 276.0995; found: 276.1001.

**1-Benzyl-3-chloro-2-(trifluoromethyl)-1*H*-indole** (**6b**). Obtained from indole **3a** (0.226 g, 1.027 mmol) by procedure (B). Colorless oil; yield 0.254 g (80%). ^1^H NMR (CDCl_3_, 400.1 MHz): δ 7.77 (d, 1H, ^3^*J* = 8.0 Hz), 7.37–7.23 (m, 6H), 7.05–6.98 (m, 2H), 5.49 (s, 2H). ^13^C{^1^H} NMR (CDCl_3_, 100.6 MHz): δ 136.5, 128.8, 127.7, 126.1, 125.7, 124.8, 121.7 (q, ^2^*J*_CF_ = 36.4 Hz), 121.6, 121.2 (q, ^1^*J*_CF_ = 270.4 Hz), 120.0, 110.8, 108.9 (q, ^3^*J*_CF_ = 2.8 Hz), 48.7 (q, ^3^*J*_CF_ = 2.2 Hz). ^19^F NMR (CDCl_3_, 376.5 MHz): δ −57.2 (s, 3F). HRMS (ESI-TOF): *m*/*z* [M + H]^+^ Calcd for C_16_H_12_ClF_3_N^+^: 310.0605; found: 310.0609.

**1-Benzyl-3-bromo-2-(trifluoromethyl)-1*H*-indole** (**6c**). Obtained from indole **3b** (1.363 g, 5.16 mmol (A); 0.057, 0.216 mmol (B)) by procedure (A) and (B). Colorless oil; yield 1.6838 g (92%, A), 0.0702 g (92%, B). ^1^H NMR (CDCl_3_, 400.1 MHz): δ 7.76 (d, 1H, ^3^*J* = 7.9 Hz), 7.41–7.23 (m, 6H), 7.09–6.99 (m, 2H), 5.54 (s, 2H). ^13^C{^1^H} NMR (CDCl_3_, 100.6 MHz): δ 137.1 (q, ^4^*J_CF_* = 1.0 Hz), 136.4, 128.8, 127.6, 126.5, 126.1, 125.7, 123.5 (q, ^2^*J*_CF_ = 35.8 Hz), 121.7, 121.2 (q, ^1^*J*_CF_ = 270.7 Hz), 121.1, 110.8, 94.1 (q, ^3^*J*_CF_ = 3.2 Hz), 48.8 (q, ^3^*J*_CF_ = 2.3 Hz). ^19^F NMR (CDCl_3_, 376.5 MHz): δ −56.9 (s, 3F). HRMS (ESI-TOF): *m*/*z* [M + H]^+^ Calcd for C_16_H_12_BrF_3_N^+^: 354.0100; found: 354.0107.

**1-Benzyl-3-iodo-2-(trifluoromethyl)-1*H*-indole** (**6d**). Obtained from indole **3c** (0.729 g, 2.34 mmol (A); 0.017, 0.055 mmol (B)) by procedure (A) and (B). Light yellow oil; yield 0.789 g (84%, A), 0.0198 g (90%, B). ^1^H NMR (CDCl_3_, 400.1 MHz): δ 7.64 (d, 1H, ^3^*J* = 7.6 Hz), 7.39–7.26 (m, 5H), 7.23 (d, 1H, ^3^*J* = 8.2 Hz), 7.05–7.00 (m, 2H), 5.55 (s, 2H). ^13^C{^1^H} NMR (CDCl_3_, 100.6 MHz): δ 137.9 (q, ^4^*J_CF_* = 1.0 Hz), 136.5, 129.9, 128.7, 127.6, 126.8 (q, ^2^*J*_CF_ = 35.4 Hz), 126.0, 125.7, 123.5, 121.9, 121.1 (q, ^1^*J*_CF_ = 271.1 Hz), 110.9, 60.7 (q, ^3^*J*_CF_ = 3.6 Hz), 49.1 (q, ^3^*J*_CF_ = 2.3 Hz). ^19^F NMR (CDCl_3_, 376.5 MHz): δ −56.3 (s, 3F). HRMS (ESI-TOF): *m*/*z* [M]^+^ Calcd for C_16_H_11_F_3_IN^+^: 400.9883; found: 400.9889.

**1-Tosyl-2-(trifluoromethyl)-1*H*-indole** (**7a**). Obtained from indole **2** (0.370 g, 2 mmol) by procedure (A). Clear oil; yield 0.65 g (96%). ^1^H NMR (CDCl_3_, 400.1 MHz): δ 8.27 (d, 1H, ^3^*J* = 8.6 Hz), 7.79 (d, 2H, ^3^*J* = 8.4 Hz), 7.57 (d, 1H, ^3^*J* = 7.9 Hz), 7.52–7.44 (m, 1H), 7.31 (t, 1H, ^3^*J* = 7.5 Hz), 7.20 (s, 1H), 7.17 (d, 2H, ^3^*J* = 8.3 Hz), 2.29 (s, 3H). ^13^C{^1^H} NMR (CDCl_3_, 100.6 MHz): δ 145.5, 137.8, 134.8, 129.7, 127.6 (q, ^2^*J*_CF_ = 39.7 Hz), 127.4, 126.91, 126.86, 124.3, 122.5, 120.3 (q, ^1^*J*_CF_ = 268.8 Hz), 115.8 (q, ^3^*J*_CF_ = 4.9 Hz), 115.2, 21.3. ^19^F NMR (CDCl_3_, 376.5 MHz): δ −57.9 (s, 3F). HRMS (ESI-TOF): *m*/*z* [M + Na]^+^ Calcd for C_16_H_12_F_3_NO_2_SNa^+^: 362.0433; found: 362.0438.

**3-Chloro-1-tosyl-2-(trifluoromethyl)-1*H*-indole** (**7b**). Obtained from indole **3a** (0.584 g, 2.67 mmol) by procedure (A). White crystals; m.p. 100–102 °C, yield 0.823 g (82%). ^1^H NMR (CDCl_3_, 400.1 MHz): δ 8.29 (d, 1H, ^3^*J* = 8.6 Hz), 7.69 (d, 2H, ^3^*J* = 8.4 Hz), 7.62 (d, 1H, ^3^*J* = 8.0 Hz), 7.54 (ddd, 1H, ^3^*J* = 8.5 Hz, ^3^*J* = 7.4 Hz, ^4^*J* = 1.2 Hz), 7.42–7.35 (m, 1H), 7.18 (d, 2H, ^3^*J* = 8.1 Hz), 2.32 (s, 3H). ^13^C{^1^H} NMR (CDCl_3_, 100.6 MHz): δ 145.7, 136.5, 134.3, 129.8, 128.9, 127.06, 127.02, 125.0, 122.3 (q, ^2^*J*_CF_ = 38.5 Hz), 121.6 (q, ^3^*J*_CF_ = 2.7 Hz), 120.4, 120.2 (q, ^1^*J*_CF_ = 271.2 Hz), 116.0, 21.6. ^19^F NMR (CDCl_3_, 376.5 MHz): δ −54.9 (s, 3F). HRMS (ESI-TOF): *m*/*z* [M + Na]^+^ Calcd for C_16_H_11_ClF_3_NO_2_SNa^+^: 396.0043; found: 396.0044.

**3-Bromo-1-tosyl-2-(trifluoromethyl)-1*H*-indole** (**7c**). Obtained from indole **3b** (1.224 g, 4.63 mmol) by procedure (A). Light beige crystals; m.p. 104–106 °C, yield 1.3525 g (70%). ^1^H NMR (CDCl_3_, 400.1 MHz): δ 8.29 (d, 1H, ^3^*J* = 8.6 Hz), 7.70 (d, 2H, ^3^*J* = 8.4 Hz), 7.60–7.51 (m, 2H), 7.40–7.34 (m, 1H), 7.17 (d, 2H, ^3^*J* = 8.1 Hz), 2.31 (s, 3H). ^13^C{^1^H} NMR (CDCl_3_, 100.6 MHz): δ 145.7, 136.9, 134.3, 129.8, 128.8, 128,4, 127.0, 125.1, 124.2 (q, ^2^*J*_CF_ = 38.3 Hz), 121.7, 120.1 (q, ^1^*J*_CF_ = 271.6 Hz), 115.9, 108.2 (q, ^3^*J*_CF_ = 3.1 Hz), 21.5. ^19^F NMR (CDCl_3_, 376.5 MHz): δ −54.3 (s, 3F). HRMS (ESI-TOF): *m*/*z* [M + Na]^+^ Calcd for C_16_H_11_BrF_3_NO_2_SNa^+^: 439.9538; found: 439.9541.

**3-Iodo-1-tosyl-2-(trifluoromethyl)-1*H*-indole** (**7d**). Obtained from indole **3c** (0.161 g, 0.518 mmol) by procedure (A) White crystals; m.p. 97–98 °C, yield 0.207 g (86%). ^1^H NMR (CDCl_3_, 400.1 MHz): δ 8.25 (d, 1H, ^3^*J* = 8.6 Hz), 7.71 (d, 2H, ^3^*J* = 8.4 Hz), 7.56–7.47 (m, 2H), 7.37 (t, 1H, ^3^*J* = 7.6 Hz), 7.17 (d, 2H, ^3^*J* = 8.5 Hz), 2.30 (s, 3H). ^13^C{^1^H} NMR (CDCl_3_, 100.6 MHz): δ 145.7, 137.1, 134.3, 131.4, 129.7, 128.7, 127.3 (q, ^2^*J*_CF_ = 37.8 Hz), 127.0, 125.1, 124.4, 119.9 (q, ^1^*J*_CF_ = 271.8 Hz), 115.8, 77.4 (q, ^3^*J*_CF_ = 3.6 Hz), 21.5. ^19^F NMR (CDCl_3_, 376.5 MHz): δ −53.4 (s, 3F). HRMS (ESI-TOF): *m*/*z* [M + Na]^+^ Calcd for C_16_H_11_F_3_INO_2_SNa^+^: 487.9400; found: 487.9394.

**Reactions of haloindoles with 4-methylbenzenethiol (general procedure).** A 4 mL vial with a screw cap was charged with the corresponding indole (0.3 mmol), DMF (0.5 mL), the corresponding base * (K_2_CO_3_ or Cs_2_CO_3_, 0.45 mmol, 1.5 equiv.), 4-methylbenzenethiol (0.36 mL of 1M solution in DMF, 0.36 mmol) and flushed with argon. The vial was tightly closed and the reaction mixture was stirred at heating* for 8 h (NH- and Ts-indoles) or 18 h (for N-Me indole) until full consuming of the starting material (^19^F NMR control). The reaction mixture was poured into 0.1 M HCl (20 mL). The product was extracted by CH_2_Cl_2_ (3 × 10 mL), the organic phase was washed by water (2 × 10 mL), brine (10 mL) and dried over Na_2_SO_4_. Volatiles were evaporated in vacuo, the residue formed was purified by column chromatography using hexane-CH_2_Cl_2_ mixture (3:1) as an eluent. Evaporation of the solvents afforded the corresponding indole. *-For base, temperature and yields see results and discussion Scheme 3.

**3-(*p*-Tolylthio)-2-(trifluoromethyl)-1*H*-indole** (**9**). Obtained: (1) from indole **3a** (0.0314 g, 0.143 mmol) using Cs_2_CO_3_ as a base (0.069 g, 0.212 mmol), yield 0.039 g (79%); (2) from indole **7b** (0.116 g, 0.311 mmol) using Cs_2_CO_3_ as a base (0.152 g, 0.468 mmol), yield 0.069 g (72%). Colorless oil. ^1^H NMR (CDCl_3_, 400.1 MHz): δ 8.70 (br.s, 1H), 7.69 (d, 1H, ^3^*J* = 8.1 Hz), 7.45 (d, 1H, ^3^*J* = 8.3 Hz), 7.41–7.35 (m, 1H), 7.22 (ddd, 1H, ^3^*J* = 8.0 Hz, ^3^*J* = 7.0 Hz, ^4^*J* = 1.0 Hz), 7.15–7.09 (m, 2H), 7.02 (d, 2H, ^3^*J* = 8.0 Hz), 2.27 (s, 3H). ^13^C{^1^H} NMR (CDCl_3_, 100.6 MHz): δ 135.6, 134.9, 133.3, 129.6, 129.3, 128.5 (q, ^2^*J*_CF_ = 36.2 Hz), 127.6, 125.5, 121.9, 120.89 (q, ^1^*J*_CF_ = 270.0 Hz), 120.91, 112.1, 106.6 (q, ^3^*J*_CF_ = 2.7 Hz), 20.9. ^19^F NMR (CDCl_3_, 376.5 MHz): δ −59.7 (s, 3F). HRMS (ESI-TOF): *m*/*z* [M + K]^+^ Calcd for C_16_H_12_F_3_NSK^+^: 346.0274; found: 346.0487.

**1-Methyl-3-(*p*-tolylthio)-2-(trifluoromethyl)-1*H*-indole** (**10**). Obtained from indole **5c** (0.093 g, 0.335 mmol) using Cs_2_CO_3_ as a base (0.163 g, 0.5 mmol). White crystals; m.p. 77–79 °C, yield 0.0557 g (52%). ^1^H NMR (CDCl_3_, 400.1 MHz): δ 7.73 (d, 1H, ^3^*J* = 8.1 Hz), 7.46–7.36 (m, 2H), 7.22 (ddd, 1H, ^3^*J* = 7.9 Hz, ^3^*J* = 5.7 Hz, ^4^*J* = 2.1 Hz), 7.06 (d, 2H, ^3^*J* = 8.2 Hz), 7.00 (d, 2H, ^3^*J* = 8.3 Hz), 3.93 (s, 3H), 2.26 (s, 3H). ^13^C{^1^H} NMR (CDCl_3_, 100.6 MHz): δ 137.9, 135.2, 134.2, 129.6, 129.0 (q, ^2^*J*_CF_ = 34.0 Hz), 128.6, 127.1, 125.3, 121.6, 121.4 (q, ^1^*J*_CF_ = 271.5 Hz), 121.3, 110.0, 106.6 (q, ^3^*J*_CF_ = 2.5 Hz), 31.6 (q, ^4^*J_CF_* = 2.5 Hz), 20.9 (q, ^4^*J_CF_* = 2.2 Hz). ^19^F NMR (CDCl_3_, 376.5 MHz): δ −57.5 (s, 3F). HRMS (ESI-TOF): *m*/*z* [M + H]^+^ Calcd for C_17_H_15_F_3_NS^+^: 322.0872; found: 322.0872.

**Reactions of haloindoles with CuCN (general procedure).** A 4 mL vial with a screw cap was charged with the corresponding indole (0.2 mmol), DMF (0.5 mL) and CuCN (0.036 g, 0.4 mmol) and flushed with argon. The vial was tightly closed and the reaction mixture was stirred at 150–155 °C for 6h until full consuming of the starting material (^19^F NMR control). The reaction mixture was poured into 0.1 M HCl (20 mL). The product was extracted by CH_2_Cl_2_ (3 × 10 mL); the organic phase was washed by water (2 × 10 mL), brine (10 mL) and dried over Na_2_SO_4_. Volatiles were evaporated in vacuo, the residue formed was purified by passing through a short silica gel pad using hexane-CH_2_Cl_2_ mixture (3:1) as an eluent. Evaporation of the solvents afforded corresponding indole.

**2-(Trifluoromethyl)-1*H*-indole-3-carbonitrile** (**11**). Obtained: (1) from indole **3b** (0.120 g, 0.455 mmol) and CuCN (0.072 g, 0.8 mmol), yield 0.0487 g (51%); (2) from indole **7c** (0.0339 g, 0.1 mmol) and CuCN (0.018 g, 0.2 mmol), yield 0.0157 g (75%); (3) from indole **3c** (0.0205 g, 0.0659 mmol) and CuCN (0.0119 g, 0.132 mmol), yield 0.0126 g (91%). Beige crystals, m.p. 173–177 °C. ^1^H NMR (CDCl_3_, 400.1 MHz): δ 9.13 (br.s, 1H), 7.84 (d, 1H, ^3^*J* = 8.1 Hz), 7.54–7.49 (m, 1H), 7.49–7.44 (m, 1H), 7.32 (ddd, 1H, ^3^*J* = 8.1 Hz, ^3^*J* = 7.0 Hz, ^4^*J* = 1.2 Hz). ^13^C{^1^H} NMR (CD_3_CN, 100.6 MHz): δ 135.9, 131.7 (q, ^2^*J*_CF_ = 39.0 Hz), 127.5, 127.3, 124.3, 120.9 (q, ^1^*J*_CF_ = 269.4 Hz), 120.8, 114.2, 113.6, 87.8 (q, ^4^*J*_CF_ = 2.3 Hz). ^19^F NMR (CDCl_3_, 376.5 MHz): δ −59.6 (s, 3F). HRMS (ESI-TOF): *m*/*z* [M + Na]^+^ Calcd for C_10_H_5_F_3_N_2_Na^+^: 233.0297; found: 233.0295.

**1-Methyl-2-(trifluoromethyl)-1*H*-indole-3-carbonitrile** (**12**). Obtained from indole **5c** (0.055 g, 0.198 mmol) and CuCN (0.036 g, 0.4 mmol). Pale yellow crystals; m.p. 111–113 °C, yield 0.040 g (90%). ^1^H NMR (CDCl_3_, 400.1 MHz): δ 7.79 (d, 1H, ^3^*J* = 8.1 Hz), 7.53–7.43 (m, 2H), 7.37 (ddd, 1H, ^3^*J* = 8.0 Hz, ^3^*J* = 6.3 Hz, ^4^*J* = 1.8 Hz), 3.92 (s, 3H). ^13^C{^1^H} NMR (CDCl_3_, 100.6 MHz): δ 137.0, 131.3 (q, ^2^*J*_CF_ = 37.8 Hz), 126.5, 126.2, 123.5, 120.7, 119.9 (q, ^1^*J*_CF_ = 270.9 Hz), 112.9, 110.8, 88.4 (q, ^4^*J*_CF_ = 2.6 Hz), 31.6 (q, ^4^*J_CF_* = 2.1 Hz). ^19^F NMR (CDCl_3_, 376.5 MHz): δ −60.0 (s, 3F). HRMS (ESI-TOF): *m*/*z* [M + H]^+^ Calcd for C_11_H_8_F_3_N_2_^+^: 225.0634; found: 225.0638.

**1-Benzyl-2-(trifluoromethyl)-1*H*-indole-3-carbonitrile** (**13**). Obtained from indole **6c** (0.068 g, 0.192 mmol) and CuCN (0.035 g, 0.389 mmol). White solid; m.p. 59–61 °C, yield 0.051 g (89%). ^1^H NMR (CDCl_3_, 400.1 MHz): δ 7.90–7.82 (s, 1H), 7.43–7.34 (m, 2H), 7.34–7.25 (m, 4H), 7.06–6.96 (m, 2H), 5.54 (s, 2H). ^13^C{^1^H} NMR (CDCl_3_, 100.6 MHz): δ 136.8, 134.8, 131.5 (q, ^2^*J*_CF_ = 37.6 Hz), 129.0, 128.2, 126.8, 126.4, 125.8, 123.7, 120.8, 119.9 (q, ^1^*J*_CF_ = 271.5 Hz), 111.8, 89.2 (q, ^3^*J*_CF_ = 2.8 Hz), 49.1 (q, ^4^*J_CF_* = 1.8 Hz). ^19^F NMR (CDCl_3_, 376.5 MHz): δ −59.1 (s, 3F). HRMS (ESI-TOF): *m*/*z* [M + H]^+^ Calcd for C_17_H_12_F_3_N_2_^+^: 301.0947; found: 301.0952.

**Cross-coupling reactions of bromoindoles with phenylboronic acid (general procedure).** A 4 mL vial with a screw cap was charged with corresponding bromoindole (0.3 mmol), phenylboronic acid (0.044 g, 0.36 mmol), dioxane (1.6 mL), Pd(PPh_3_)_4_ (0.017 g, 0.015 mmol) and flushed with argon. The reaction mixture was stirred to form a homogeneous solution and then K_2_CO_3_ solution in water was added (0.083 g of K_2_CO_3_ and 1.6 mL of water). The vial was flushed with argon and tightly closed. Next, the reaction mixture was stirred at 95 °C for 4–6h until full consuming of the starting material (^19^F NMR control). The reaction mixture was poured into water (30 mL). The product was extracted by CH_2_Cl_2_ (3 × 10 mL), the organic phase was washed by water (10 mL), brine (10 mL), and dried over Na_2_SO_4_. Volatiles were evaporated in vacuo, and the residue formed was purified by column chromatography using hexane-CH_2_Cl_2_ mixture (3:1) as an eluent. Evaporation of the solvents afforded corresponding indole.

**3-Phenyl-2-(trifluoromethyl)-1*H*-indole** (**14**). Obtained from indole **3b** (0.150 g, 0.57 mmol) and phenylboronic acid (0.084 g, 0.689 mmol). White crystals; m.p. 65–66 °C, yield 0.107 g (72%). ^1^H NMR (CDCl_3_, 400.1 MHz): δ 8.49 (br.s, 1H), 7.67 (d, 1H, ^3^*J* = 8.1 Hz), 7.58–7.52 (m, 2H), 7.52–7.35 (m, 5H), 7.22 (td, 1H, ^3^*J* = 7.1 Hz, ^4^*J* = 3.5 Hz). ^13^C{^1^H} NMR (CDCl_3_, 100.6 MHz): δ 134.9, 132.1, 129.88, 129.87, 128.4, 127.5, 127.3, 125.1, 121.6 (q, ^1^*J*_CF_ = 269.1 Hz), 121.3, 121.13 (q, ^2^*J*_CF_ = 37.3 Hz), 121.06, 119.8 (q, ^3^*J*_CF_ = 2.2 Hz), 111.6. ^19^F NMR (CDCl_3_, 376.5 MHz): δ −58.0 (s, 3F). NMR data of indole **14** are in agreement with those in the literature [51].

**1-Metyl-3-phenyl-2-(trifluoromethyl)-1*H*-indole** (**15**). Obtained from indole **5c** (0.0738 g, 0.265 mmol) and phenylboronic acid (0.039 g, 0.32 mmol). Colorless oil; yield 0.064 g (88%). ^1^H NMR (CDCl_3_, 400.1 MHz): δ 7.57 (d, 1H, ^3^*J* = 8.1 Hz), 7.53–7.35 (m, 7H), 7.22–7.18 (m, 1H), 3.94 (*pseudo*-d, 3H, ^3^*J* = 0.9 Hz). ^13^C{^1^H} NMR (CDCl_3_, 100.6 MHz): δ 137.5 (q, ^4^*J_CF_* = 1.1 Hz), 132.8, 130.4, 128.0, 127.3, 126.6, 124.9, 122.4 (q, ^2^*J*_CF_ = 34.8 Hz), 121.9 (q, ^1^*J*_CF_ = 270.4 Hz), 121.2, 120.8, 120.4 (q, ^3^*J*_CF_ = 3.0 Hz), 109.6, 31.1 (q, ^4^*J_CF_* = 2.8 Hz). ^19^F NMR (CDCl_3_, 376.5 MHz): δ −56.2 (s, 3F). NMR data of indole **15** are in agreement with those in the literature [67].

**1-Benzyl-3-phenyl-2-(trifluoromethyl)-1*H*-indole** (**16**). Obtained from indole **6c** (0.075 g, 0.212 mmol) and phenylboronic acid (0.031 g, 0.254 mmol). Colorless oil; yield 0.065 g (87%). ^1^H NMR (CDCl_3_, 400.1 MHz): δ 7.60 (d, 1H, ^3^*J* = 8.1 Hz), 7.55–7.47 (m, 4H), 7.47–7.40 (m, 1H), 7.37–7.26 (m, 5H), 7.20 (ddd, 1H, ^3^*J* = 8.0 Hz, ^3^*J* = 6.5 Hz, ^4^*J* = 1.4 Hz), 7.12 (d, 2H, ^3^*J* = 6.9 Hz), 5.58 (s, 2H). ^13^C{^1^H} NMR (CDCl_3_, 100.6 MHz): δ 137.4, 137.0, 132.8, 130.4, 128.7, 128.1, 127.47, 127.43, 126.9, 125.9, 125.2, 122.5 (q, ^2^*J*_CF_ = 35.0 Hz), 121.9 (q, ^1^*J*_CF_ = 270.5 Hz), 121.3, 121.1, 120.9 (q, ^3^*J*_CF_ = 3.0 Hz), 110.6, 48.4 (q, ^4^*J_CF_* = 2.2 Hz). ^19^F NMR (CDCl_3_, 376.5 MHz): δ −55.4 (s, 3F). HRMS (ESI-TOF): *m*/*z* [M + H]^+^ Calcd for C_22_H_17_F_3_N^+^: 352.1308; found: 352.1308.

**Cross-coupling reactions of iodoindoles with phenylacetylene (general procedure).** A 8 mL vial with a screw cap was charged with the corresponding iodoindole (0.3 mmol), phenylacetylene (0.046 g, 0.45 mmol), NEt_3_ (1.5 mL), Pd(PPh_3_)_2_Cl_2_ (0.0105 g, 0.015 mmol) and CuI (0.0057 g, 0.03 mmol) and flushed with argon. The vial was tightly closed and stirred for 2 days at room temperature until full consuming of the starting material (^19^F NMR control). Volatiles were evaporated; the residue formed was purified by column chromatography using hexane-CH_2_Cl_2_ mixture (3:1) as an eluent. Evaporation of the solvents afforded corresponding indole.

**1-Methyl-3-(phenylethynyl)-2-(trifluoromethyl)-1*H*-indole** (**17**). Obtained from indole **5d** (0.072 g, 0.222 mmol) and phenylacetylene (0.034 g, 0.333 mmol). Colorless oil; yield 0.0645 g (97%). ^1^H NMR (CDCl_3_, 400.1 MHz): δ 7.90 (d, 1H, ^3^*J* = 8.0 Hz), 7.65–7.57 (m, 2H), 7.46–7.28 (m, 6H), 3.87 (s, 3H). ^13^C{^1^H} NMR (CDCl_3_, 100.6 MHz): δ 137.3, 131.4, 128.3, 128.1, 127.45 (q, ^2^*J*_CF_ = 35.3 Hz), 127.43, 125.5, 123.6, 121.5, 121.33, 120.31 (q, ^1^*J*_CF_ = 270.4 Hz), 110.0, 100.6 (q, ^3^*J*_CF_ = 2.9 Hz), 94.8 (q, ^4^*J*_CF_ = 1.6 Hz), 80.1, 31.2 (q, ^4^*J_CF_* = 2.6 Hz). ^19^F NMR (CDCl_3_, 376.5 MHz): δ −58.7 (s, 3F). HRMS (ESI-TOF): *m*/*z* [M + H]^+^ Calcd for C_18_H_13_F_3_N^+^: 300.0995; found: 300.0999.

**1-Benzyl-3-(phenylethynyl)-2-(trifluoromethyl)-1*H*-indole** (**18**). Obtained from indole **6d** (0.046 g, 0.115 mmol) and phenylacetylene (0.018 g, 0.176 mmol). Beige precipitate, m.p. 86–87 °C, yield 0.0371 g (86%). ^1^H NMR (CDCl_3_, 400.1 MHz): δ 7.93 (dd, 1H, ^3^*J* = 7.1 Hz, ^4^*J* = 1.1 Hz), 7.64–7.59 (m, 2H), 7.42–7.23 (m, 9H), 7.07–6.58 (m, 2H), 5.51 (s, 2H). ^13^C{^1^H} NMR (CDCl_3_, 100.6 MHz): δ 137.1 (q, ^4^*J_CF_* = 1.1 Hz), 136.3, 131.5, 128.8, 128.41, 128.35, 128.2, 127.71, 127.65, 127.6 (q, ^2^*J*_CF_ = 35.6 Hz), 125.81, 125.78, 123.5, 121.8, 121.4, 120.3 (q, ^1^*J*_CF_ = 270.7 Hz), 111.0, 101.3 (q, ^3^*J*_CF_ = 3.4 Hz), 95.1 (q, ^4^*J*_CF_ = 1.6 Hz), 80.0, 48.6 (q, ^3^*J_CF_* = 2.1 Hz). ^19^F NMR (CDCl_3_, 376.5 MHz): δ −58.0 (s, 3F). HRMS (ESI-TOF): *m*/*z* [M + H]^+^ Calcd for C_24_H_17_F_3_N^+^: 376.1308; found: 376.1314.

**3-(Phenylethynyl)-1-tosyl-2-(trifluoromethyl)-1*H*-indole** (**19**). Obtained from indole **7d** (0.189 g, 0.394 mmol) and phenylacetylene (0.060 g, 0.588 mmol). White precipitate; m.p. 139–141 °C, yield 0.151 g (87%). ^1^H NMR (CDCl_3_, 400.1 MHz): δ 8.30 (d, 1H, ^3^*J* = 8.6 Hz), 7.84–7.71 (m, 3H), 7.60–7.50 (m, 3H), 7.45–7.34 (m, 4H), 7.19 (d, 2H, ^3^*J* = 8.2 Hz), 2.32 (s, 3H). ^13^C{^1^H} NMR (CDCl_3_, 100.6 MHz): δ 145.7, 136.9, 134.6, 131.8, 129.8, 129.1, 128.7, 128.5, 128.3, 127.2 (q, ^2^*J*_CF_ = 37.8 Hz), 127.0, 124.8, 122.3, 121.5, 120.3 (q, ^1^*J*_CF_ = 270.1 Hz), 115.7, 112.1 (q, ^3^*J*_CF_ = 3.2 Hz), 99.1 (q, ^4^*J*_CF_ = 1.9 Hz), 78.4, 21.5 (q, ^4^*J_CF_* = 2.4 Hz). ^19^F NMR (CDCl_3_, 376.5 MHz): δ −55.5 (s, 3F). HRMS (ESI-TOF): *m*/*z* [M + H]^+^ Calcd for C_24_H_17_F_3_NO_2_S^+^: 440.0927; found: 440.0931.

## 4. Conclusions

In conclusion, we investigated the modification of 2-CF_3_-indoles at the position C-3 and nitrogen atoms. A set of previously unknown 3-chloro-, 3-bromo- and 3-iodo-2-CF_3_-indoles was prepared in almost quantitative yields (93–98%). These indoles can be easily modified at the nitrogen atom by the reaction with MeI, BnBr and tosyl chloride in the presence of bases. The prepared trifluoromethylated indoles are valuable building blocks for the synthesis of 3-functionalized derivatives by the reactions with nucleophiles. However, the reaction path depends on the halogen’s nature. Thus, the reaction of 3-chloro-2-CF_3_-indoles with 4-methylthiophenol afforded the corresponding sulfides in high yield. The Suzuki reaction of 3-bromo-2-CF_3_-indoles with phenyl boronic acid gave the corresponding 3-phenyl-2-CF_3_-indoles in 72–88% yield. 3-Cyano-2-CF_3_-indoles were synthesized in about 90% yields by the reaction of 3-iodo-2-CF_3_-indoles with CuCN. Sonogashira reactions of 3-iodo-2-CF_3_-indoles with phenylacetylene led smoothly to the corresponding acetylenic derivatives in high yield.

## Data Availability

The data presented in this study are available in Supplementary Materials.

## Supplementary Materials

The following supporting information can be downloaded at: https://www.mdpi.com/article/10.3390/molecules27248822/s1, Figure S2 1H NMR spectrum of 3a (400.1 MHz, CDCl3); Figure S3 19F NMR spectrum of 3a (376.5 MHz, CDCl3); Figure S4 13C{1H} NMR spectrum of 3a (100.6 MHz, CDCl3); Figure S5 1H NMR spectrum of 3b (400.1 MHz, CDCl3); Figure S6 19F NMR spectrum of 3b (376.5 MHz, CDCl3); Figure S7 13C{1H} NMR spectrum of 3b (100.6 MHz, CDCl3); Figure S8 1H NMR spectrum of 3c (400.1 MHz, CDCl3); Figure S9 19F NMR spectrum of 3c (376.5 MHz, CDCl3); 13C{1H} NMR spectrum of 3c (100.6 MHz, CDCl3); Figure S11 1H NMR spectrum of 4 (400.1 MHz, CDCl3); Fig-ure S12 19F NMR spectrum of 4 (376.5 MHz, CDCl3); Figure S13 19F NMR spectrum of 4 (376.5 MHz, CDCl3; Figure S14 1H NMR spectrum of 5a (400.1 MHz, CDCl3); Figure S15 19F NMR spectrum of 5a (376.5 MHz, CDCl3); Figure S16 13C{1H} NMR spectrum of 5a (100.6 MHz, CDCl3); Figure S17 1H NMR spectrum of 5b (400.1 MHz, CDCl3); Figure S18 19F NMR spectrum of 5b (376.5 MHz, CDCl3); Figure S19 13C{1H} NMR spectrum of 5b (100.6 MHz, CDCl3); Figure S20 1H NMR spectrum of 5c (400.1 MHz, CDCl3); Figure S21 19F NMR spectrum of 5c (376.5 MHz, CDCl3); Figure S22 13C{1H} NMR spectrum of 5c (100.6 MHz, CDCl3); Figure S23 1H NMR spectrum of 5d (400.1 MHz, CDCl3); Figure S24 19F NMR spectrum of 5d (376.5 MHz, CDCl3); Figure S25 13C{1H} NMR spectrum of 5d (100.6 MHz, CDCl3); Figure S26 1H NMR spectrum of 6a (400.1 MHz, CDCl3); Figure S27 19F NMR spectrum of 6a (376.5 MHz, CDCl3); Figure S28 13C{1H} NMR spectrum of 6a (100.6 MHz, CDCl3); Figure S29 1H NMR spectrum of 6b (400.1 MHz, CDCl3); Figure S30 19F NMR spectrum of 6b (376.5 MHz, CDCl3); Figure S31 13C{1H} NMR spectrum of 6b (100.6 MHz, CDCl3); Figure S32 1H NMR spectrum of 6c (400.1 MHz, CDCl3); Figure S33 19F NMR spectrum of 6c (376.5 MHz, CDCl3); Figure S34 13C{1H} NMR spectrum of 6c (100.6 MHz, CDCl3); Figure S35 1H NMR spectrum of 6d (400.1 MHz, CDCl3); Figure S36 19F NMR spectrum of 6d (376.5 MHz, CDCl3); Figure S37 13C{1H} NMR spectrum of 6d (100.6 MHz, CDCl3); Figure S38 1H NMR spectrum of 7a (400.1 MHz, CDCl3); Figure S39 19F NMR spectrum of 7a (376.5 MHz, CDCl3); Figure S40 13C{1H} NMR spectrum of 7a (100.6 MHz, CDCl3); Figure S41 1H NMR spectrum of 7b (400.1 MHz, CDCl3); Figure S42 19F NMR spectrum of 7b (376.5 MHz, CDCl3); Figure S43 13C{1H} NMR spectrum of 7b (100.6 MHz, CDCl3); Figure S44 1H NMR spectrum of 7c (400.1 MHz, CDCl3); Figure S45 19F NMR spectrum of 7c (376.5 MHz, CDCl3); Figure S46 13C{1H} NMR spectrum of 7c (100.6 MHz, CDCl3); Figure S47 1H NMR spectrum of 7d (400.1 MHz, CDCl3); Figure S48 19F NMR spectrum of 7d (376.5 MHz, CDCl3); Figure S49 13C{1H} NMR spectrum of 7d (100.6 MHz, CDCl3); Figure S50 1H NMR spectrum of 9 (400.1 MHz, CDCl3); Figure S51 19F NMR spectrum of 9 (376.5 MHz, CDCl3); Figure S52 13C{1H} NMR spectrum of 9 (100.6 MHz, CDCl3); Figure S53 1H NMR spectrum of 10 (400.1 MHz, CDCl3); Figure S54 19F NMR spectrum of 10 (376.5 MHz, CDCl3); Figure S55 13C{1H} NMR spectrum of 10 (100.6 MHz, CDCl3); Figure S56 1H NMR spectrum of 11 (400.1 MHz, CD3CN); Figure S57 19F NMR spectrum of 11 (376.5 MHz, CDCl3); Figure S58 13C{1H} NMR spectrum of 11 (100.6 MHz, CD3CN); Figure S59 1H NMR spectrum of 12 (400.1 MHz, CDCl3); Figure S60 19F NMR spectrum of 12 (376.5 MHz, CDCl3); Figure S61 13C{1H} NMR spectrum of 12 (100.6 MHz, CDCl3); Figure S62 1H NMR spectrum of 13 (400.1 MHz, CDCl3); Figure S63 19F NMR spectrum of 13 (376.5 MHz, CDCl3); Figure S64 13C{1H} NMR spectrum of 13 (100.6 MHz, CDCl3); Figure S65 1H NMR spectrum of 14 (400.1 MHz, CDCl3); Figure S66 19F NMR spectrum of 14 (376.5 MHz, CDCl3); Figure S67 13C{1H} NMR spectrum of 14 (100.6 MHz, CDCl3); Figure S68 1H NMR spectrum of 15 (400.1 MHz, CDCl3); Figure S69 19F NMR spectrum of 15 (376.5 MHz, CDCl3); Figure S70 13C{1H} NMR spectrum of 15 (100.6 MHz, CDCl3); Figure S71 1H NMR spectrum of 16 (400.1 MHz, CDCl3; Figure S72 19F NMR spectrum of 16 (376.5 MHz, CDCl3); Figure S73 13C{1H} NMR spectrum of 16 (100.6 MHz, CDCl3); Figure S74 1H NMR spectrum of 17 (400.1 MHz, CDCl3); Figure S75 19F NMR spectrum of 17 (376.5 MHz, CDCl3); Figure S76 13C{1H} NMR spectrum of 17 (100.6 MHz, CDCl3); Figure S77 1H NMR spectrum of 18 (400.1 MHz, CDCl3); Figure S78 19F NMR spectrum of 18 (376.5 MHz, CDCl3); Figure S79 13C{1H} NMR spectrum of 18 (100.6 MHz, CDCl3); Figure S80 1H NMR spectrum of 19 (400.1 MHz, CDCl3); Figure S81 19F NMR spectrum of 19 (376.5 MHz, CDCl3); Figure S82 13C{1H} NMR spectrum of 19 (100.6 MHz, CDCl3).

## Sample Availability

Samples of the compounds **3** and **4** are available from the authors.
