# Supplementary material for: Synthesis and Reactions of 3-Halogenated 2-CF3-Indoles
Source: Molecules. 2022 Dec 12;27(24):8822. doi: 10.3390/molecules27248822 (PMC9785211; doi:10.3390/molecules27248822)
Supplement: Supplementary file 1 [file molecules-27-08822-s001.zip › molecules-2079731-supplementary.pdf]

Supporting Information

Synthesis and reactions of 3-halogenated 2-CF_3-indoles

Vasiliy M. Muzalevskiy, Zoia A. Sizova and Valentine G. Nenajdenko*

Department of Chemistry, Lomonosov Moscow State University, 119899 Moscow, Russia.;

muzvas@mail.ru (V.M.M); syzova@mail.ru (Z.A.S.)

* Correspondence: nenajdenko@org.chem.msu.ru (V.G.N.)

Copies of all NMR spectra **S2-S82**

Acquisition Time (sec)	4.0894	***Comment***	Imported from UXNMR.			***Date***	07 Jun 2021 14:58:48
File Name	I:\SPEC_H,C_2021\06.июнь\SZA-BM-2188-3.H_001001r			***Frequency (MHz)***	400.13	***Nucleus***	1H
Number of Transients	4	***Original Points Count***	32768	***Points Count***	131072	***Pulse Sequence***	zg30
Solvent	CHLOROFORM-D			***Sweep Width (Hz)***	8012.82	***Temperature (degree C)***	27.000

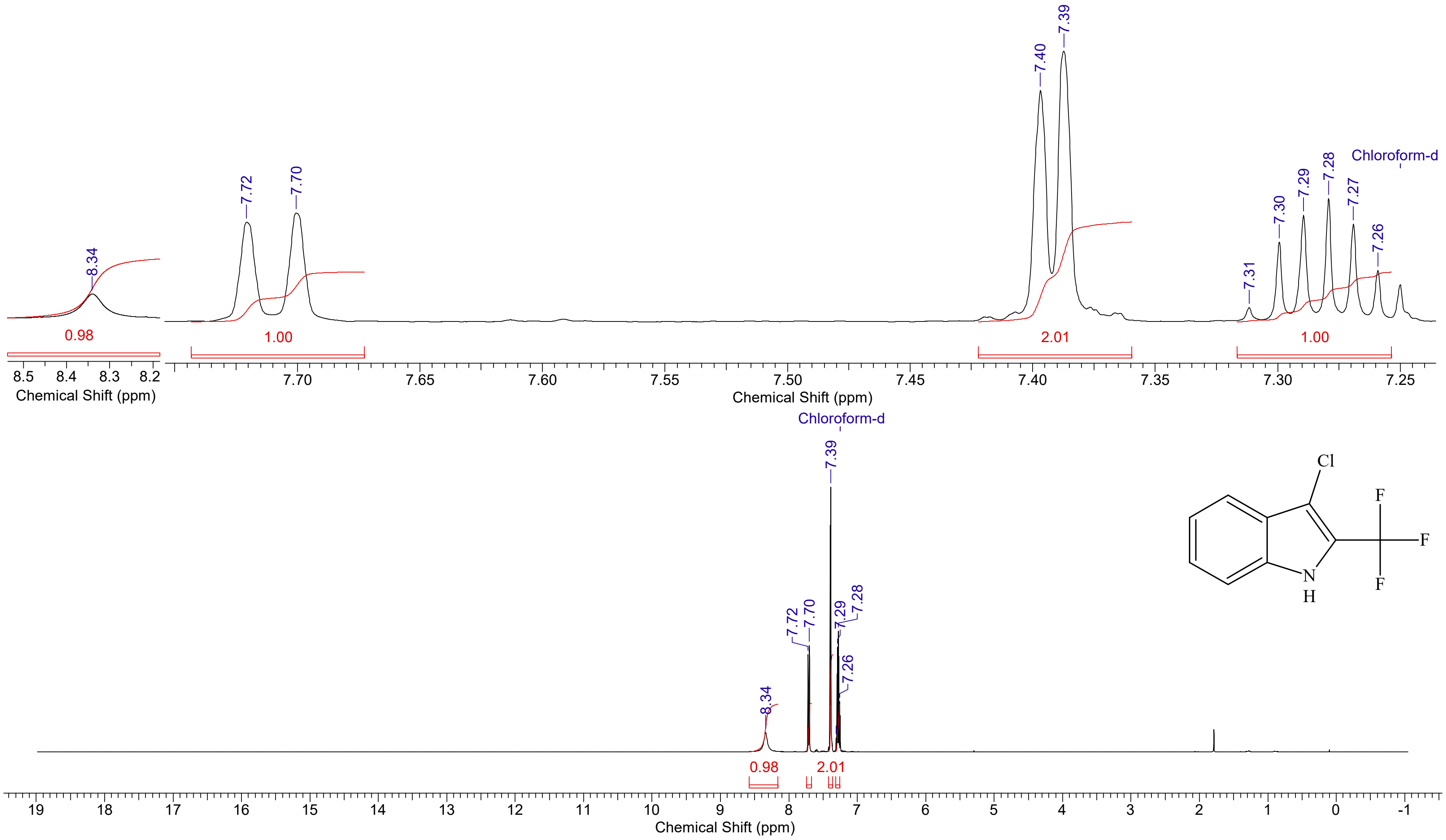

^{1}H NMR spectrum of **3a** (400.1 MHz, $CDCl_3$)

Acquisition Time (sec)	1.7433	Comment	Imported from UXNMR.			Date	08 Jun 2021 15:45:36
File Name	C:\BM_DATA\DOCS\08.06.2021\08.06.2021\SZA-BM-2188-3.F_005001r					Frequency (MHz)	376.50
Nucleus	19F	Number of Transients	16	Original Points Count	131072	Points Count	262144
Pulse Sequence	zgflqn	Solvent	CHLOROFORM-D			Sweep Width (Hz)	75187.97
Temperature (degree C)	27.000						

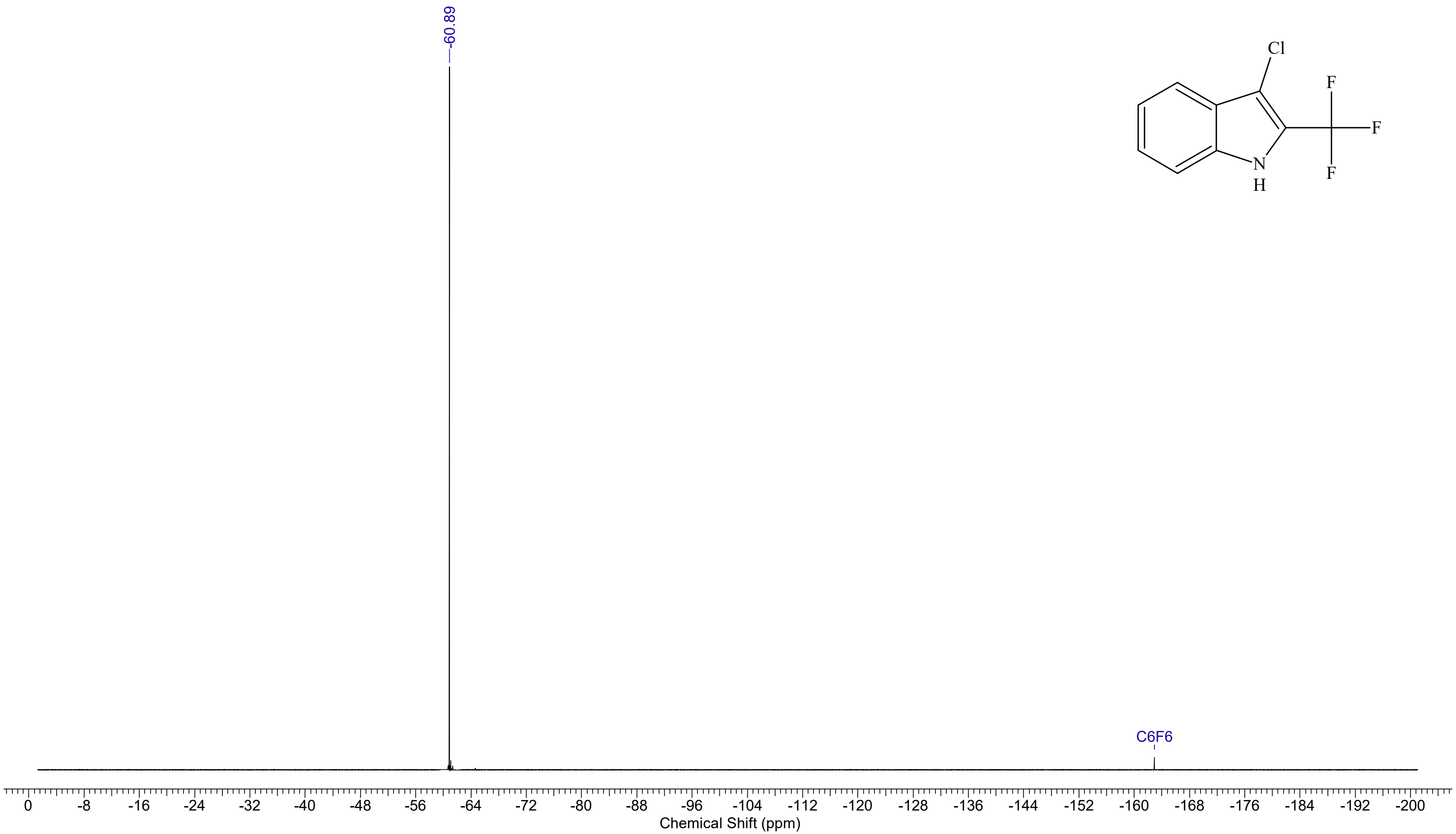

^{19}F NMR spectrum of **3a** (376.5 MHz, $CDCl_3$)

1 Aug 2021

Acquisition Time (sec)	0.6783	***Comment***	Imported from UXNMR.			***Date***	08 Jun 2021 15:32:06
File Name	C:\BM_DATA\DOCS\08.06.2021\08.06.2021\SZA-BM-2188-3.C_002001r					***Frequency (MHz)***	100.61
Nucleus	13C	***Number of Transients***	1356	***Original Points Count***	16384	***Points Count***	131072
Pulse Sequence	zgpg30	***Solvent***	DMSO-D6	***Sweep Width (Hz)***	24154.59	***Temperature (degree C)***	27.000

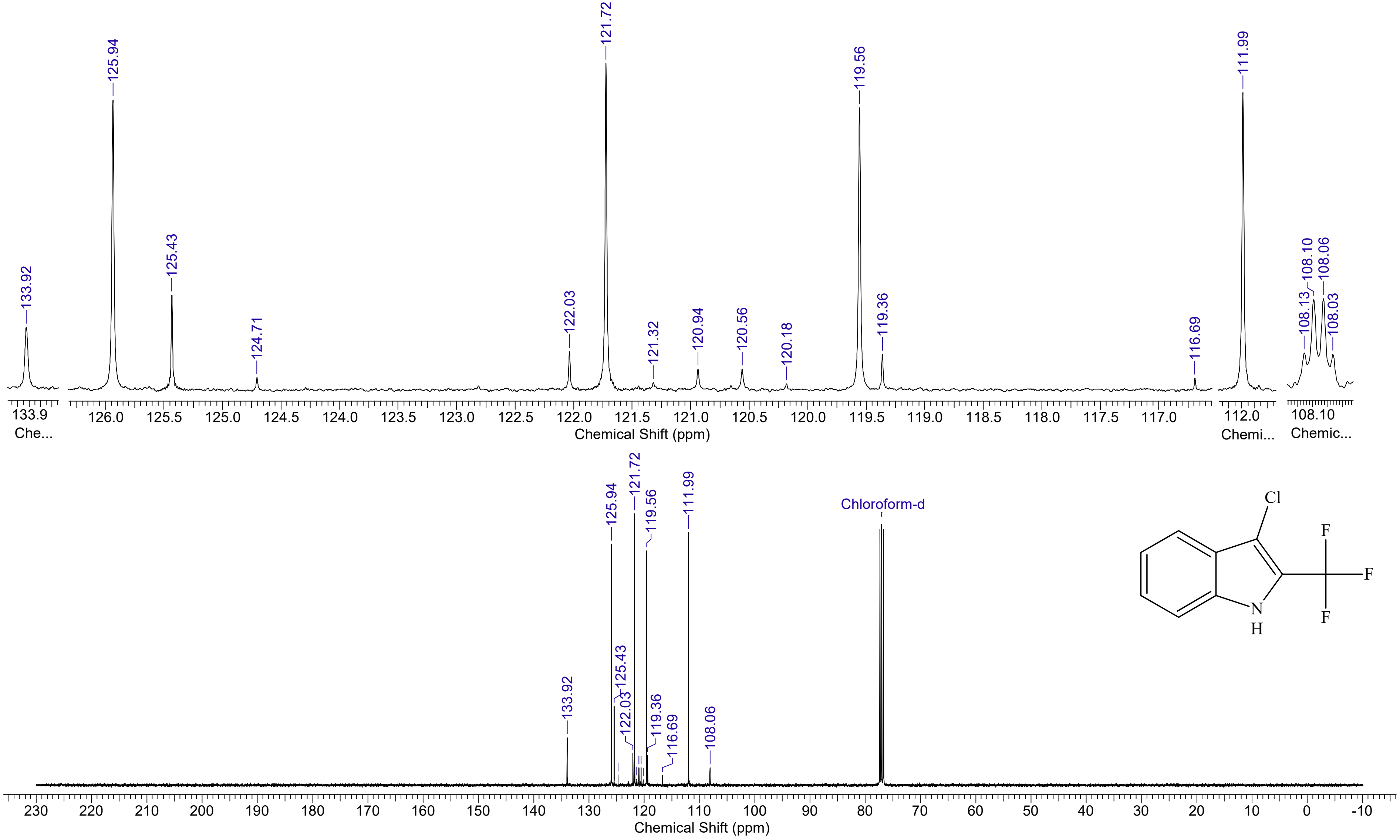

$^{13}C\{^{1}H\}$ NMR spectrum of **3a** (100.6 MHz, $CDCl_3$)

Acquisition Time (sec)	4.0894	Comment	Imported from UXNMR.			Date	29 May 2021 12:15:00
File Name	C:\DOCS\BM\ЯМР_СОСЕДИ\2021\210529\BM-2172-2_001001r					Frequency (MHz)	400.13
Nucleus	1H	Number of Transients	8	Original Points Count	32768	Points Count	131072
Pulse Sequence	zg30	Solvent	CHLOROFORM-D			Sweep Width (Hz)	8012.82
Temperature (degree C)	27.000						

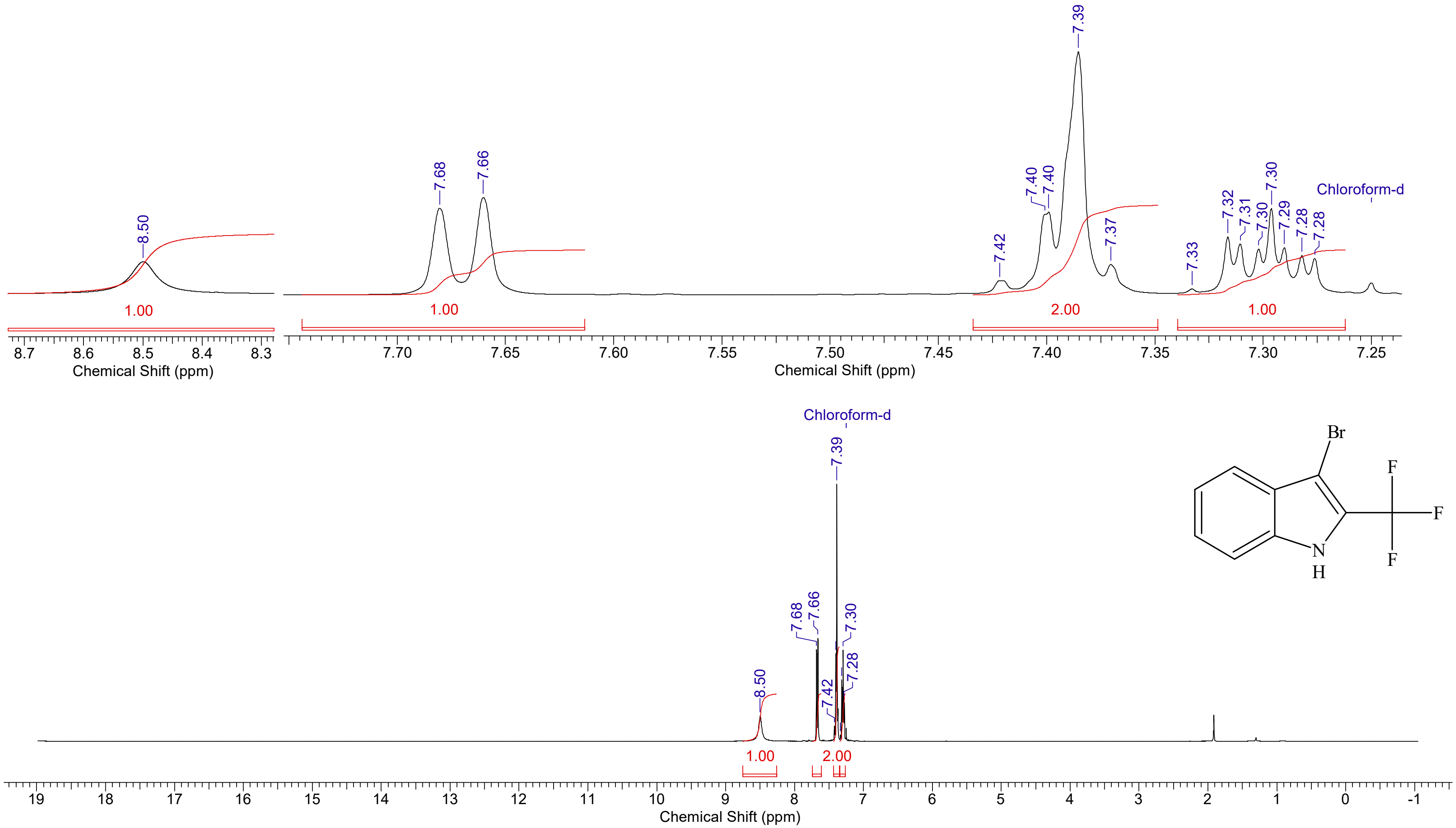

^{1}H NMR spectrum of **3b** (400.1 MHz, $CDCl_3$)

1 Aug 2021

Acquisition Time (sec)	1.7433	Comment	Imported from UXNMR.			Date	31 May 2021 15:44:56
File Name	C:\DOCS\OUTPUT_301\2021\05.май\BM-2172-2.F_005001r					Frequency (MHz)	376.50
Nucleus	19F	Number of Transients	16	Original Points Count	131072	Points Count	262144
Pulse Sequence	zgflqn	Solvent	DMSO-D6	Sweep Width (Hz)	75187.97	Temperature (degree C)	27.000

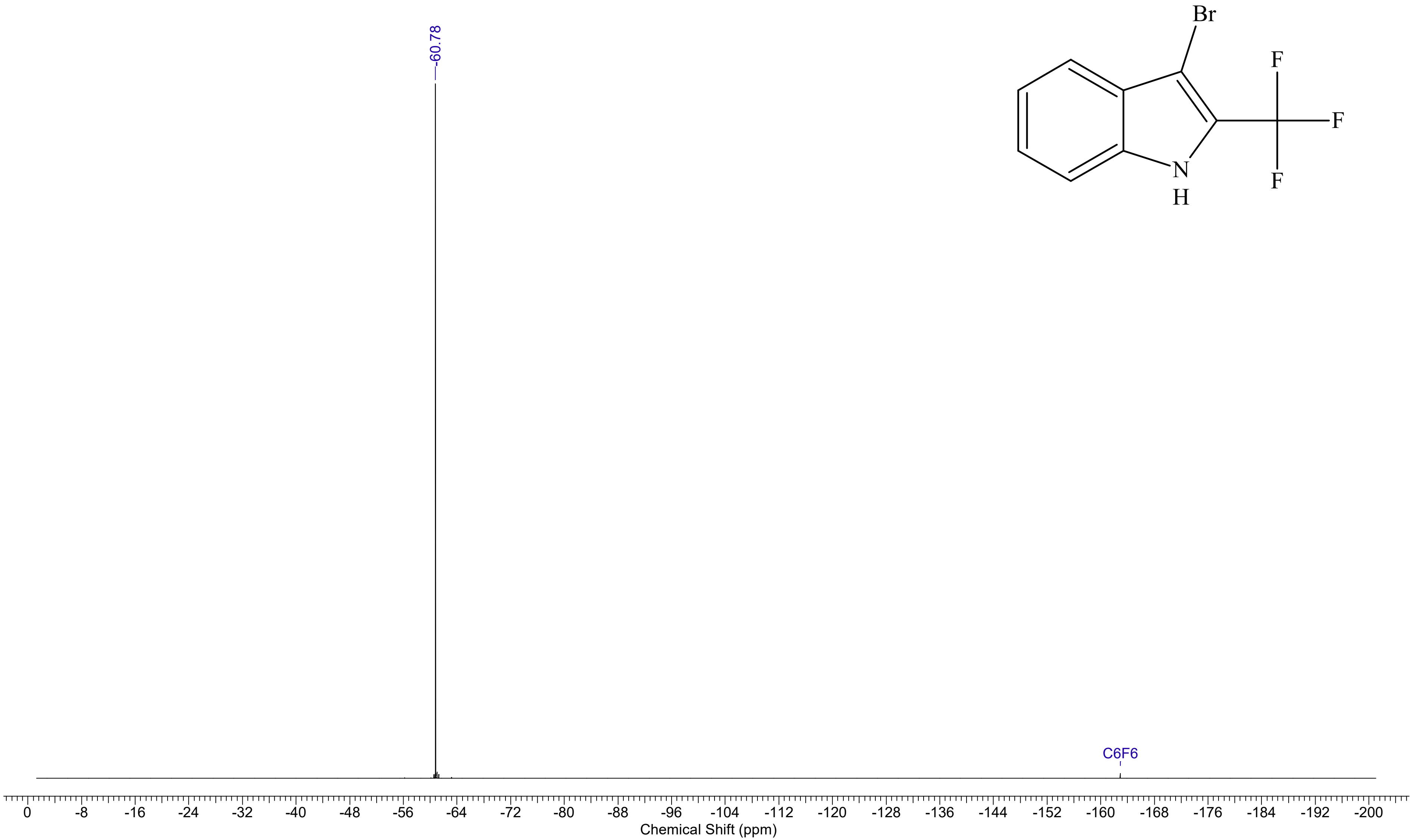

^{19}F NMR spectrum of **3b** (376.5 MHz, $CDCl_3$)

1 Aug 2021

Acquisition Time (sec)	0.6783	***Comment***	Imported from UXNMR.			***Date***	29 May 2021 12:25:10
File Name	C:\DOCS\BM\ЯМР_СОСЕДИ\2021\210529\BM-2172-2_002001r					***Frequency (MHz)***	100.61
Nucleus	13C	***Number of Transients***	227	***Original Points Count***	16384	***Points Count***	131072
Pulse Sequence	zgpg30	***Solvent***	CHLOROFORM-D			***Sweep Width (Hz)***	24154.59
Temperature (degree C)	27.000						

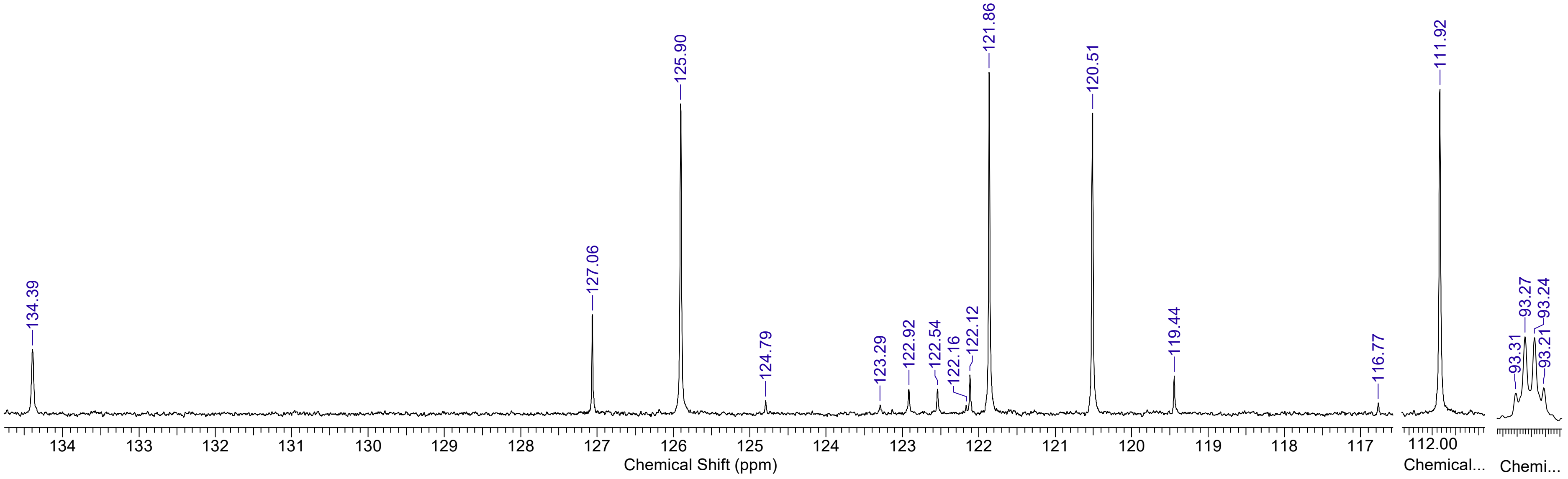

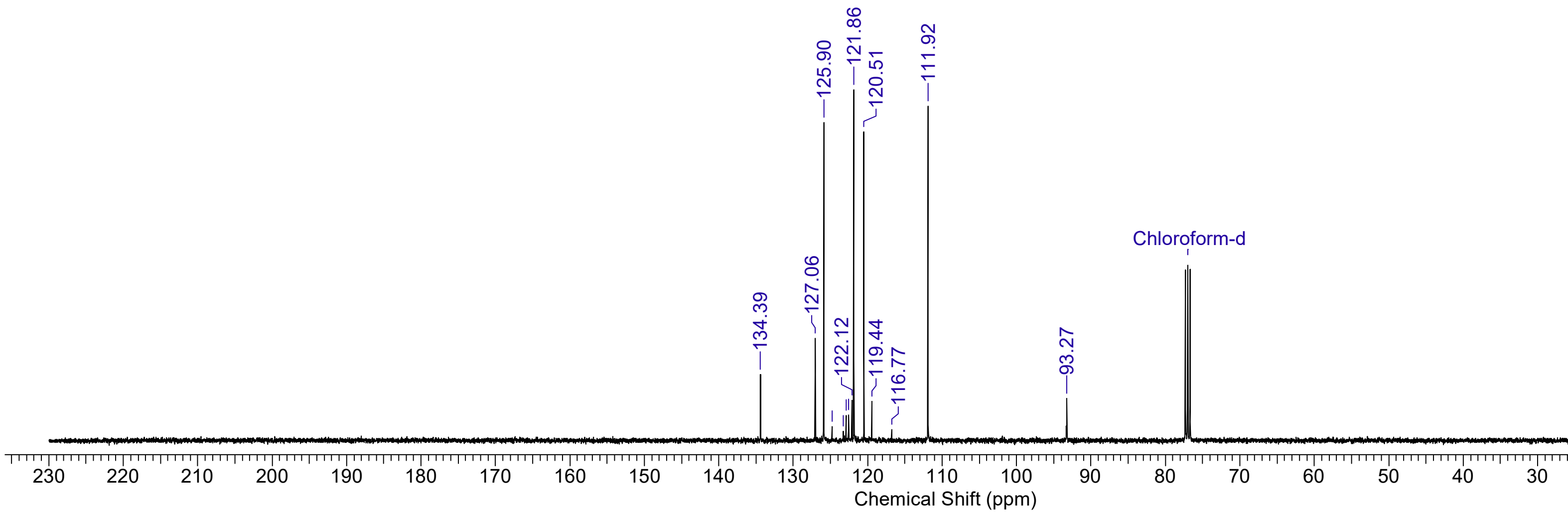

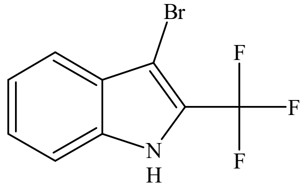

$^{13}C\{^{1}H\}$ NMR spectrum of **3b** (100.6 MHz, $CDCl_3$)

1 Aug 2021

Acquisition Time (sec)	4.0894	***Comment***	Imported from UXNMR.	***Date***	01 Jun 2021 14:24:34
File Name	C:\DOCS\OUTPUT_301\2021\06.июнь\BM-2173.H_001001r	***Frequency (MHz)***	400.13	***Nucleus***	1H
Number of Transients	4	***Original Points Count*** 32768	***Points Count*** 131072	***Pulse Sequence***	zg30
Solvent	CHLOROFORM-D	***Sweep Width (Hz)***	8012.82	***Temperature (degree C)***	27.000

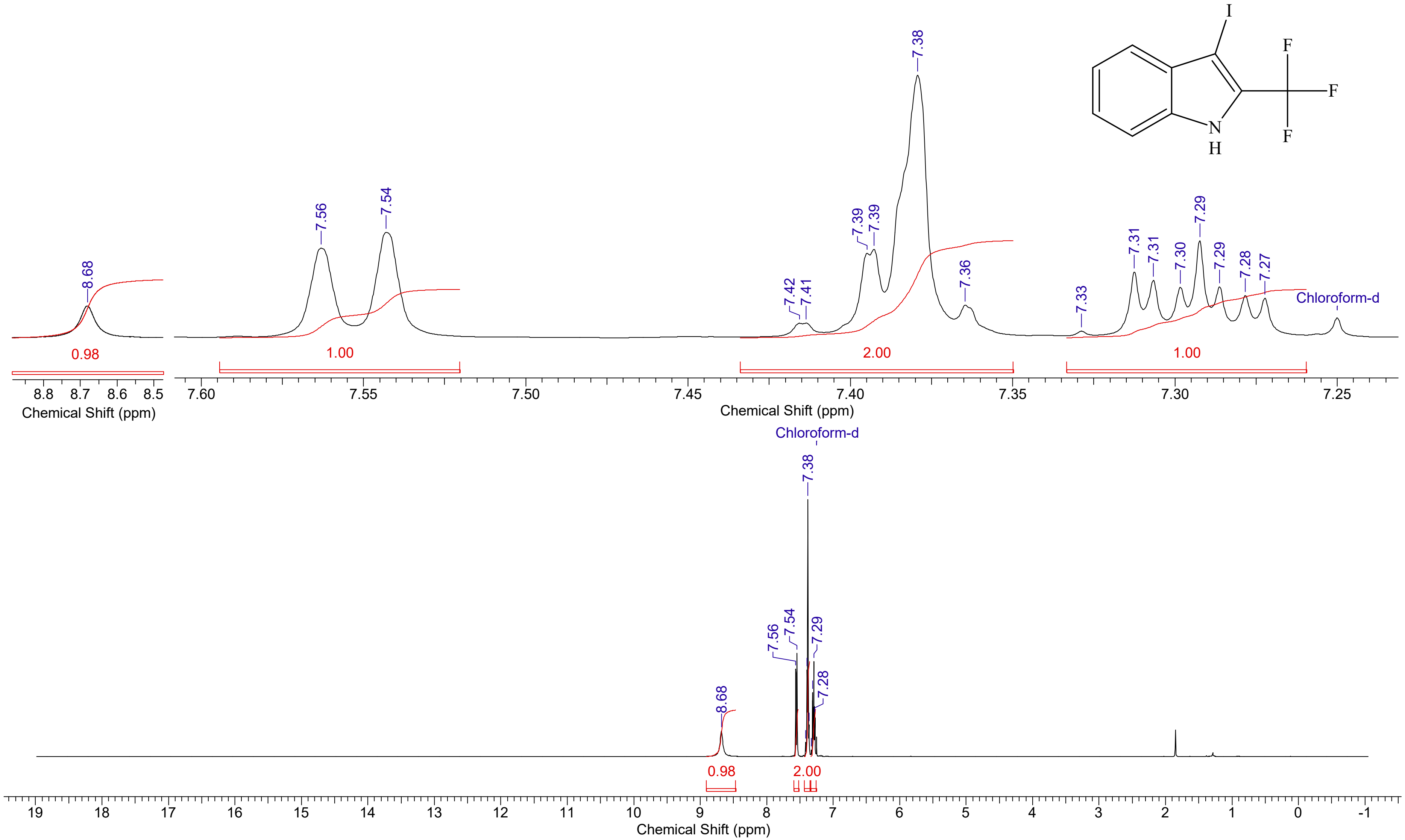

^{1}H NMR spectrum of **3c** (400.1 MHz, $CDCl_3$)

1 Aug 2021

Acquisition Time (sec)	1.7433	***Comment***	Imported from UXNMR.			***Date***	31 May 2021 15:42:10
File Name	C:\DOCS\OUTPUT_301\2021\05.май\BM-2173.F_005001r			***Frequency (MHz)***	376.50	***Nucleus***	19F
Number of Transients	16	***Original Points Count***	131072	***Points Count***	262144	***Pulse Sequence***	zgflqn
Solvent	DMSO-D6	***Sweep Width (Hz)***	75187.97	***Temperature (degree C)***	27.000		

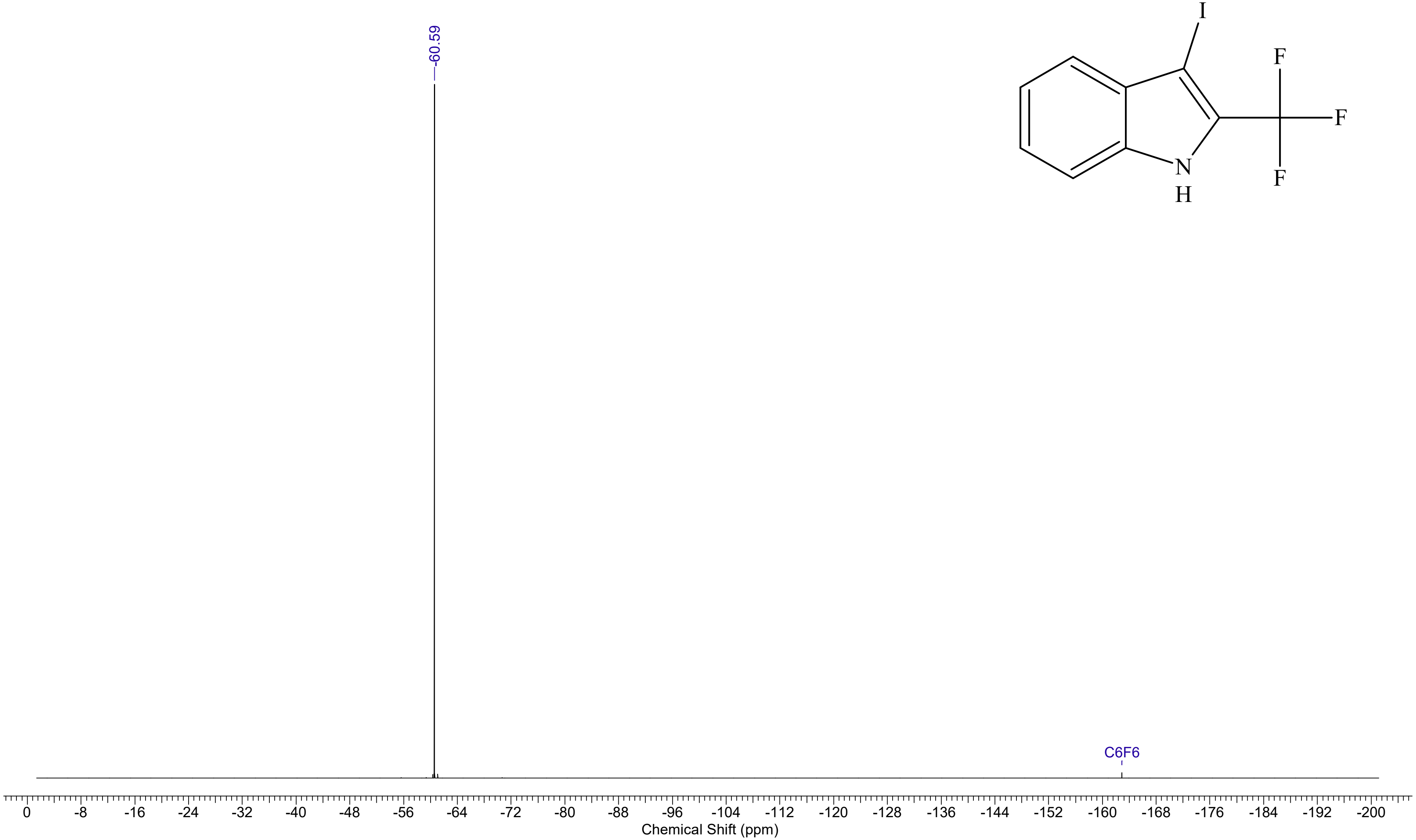

^{19}F NMR spectrum of **3c** (376.5 MHz, $CDCl_3$)

1 Aug 2021

FW	311.0424	*Formula*	$C_9H_5F_3IN$

Acquisition Time (sec)	0.6783	*Comment*	Imported from UXNMR.			*Date*	31 May 2021 15:36:14		
File Name	I:\SPEC_H,C_2021\05.май\BM-2173.C_002001r			*Frequency (MHz)*	100.61	*Nucleus*	13C	*Number of Transients*	257
Original Points Count	16384	*Points Count*	131072	*Pulse Sequence*	zgpg30	*Solvent*	DMSO-D6	*Sweep Width (Hz)*	24154.59
Temperature (degree C)	27.000								

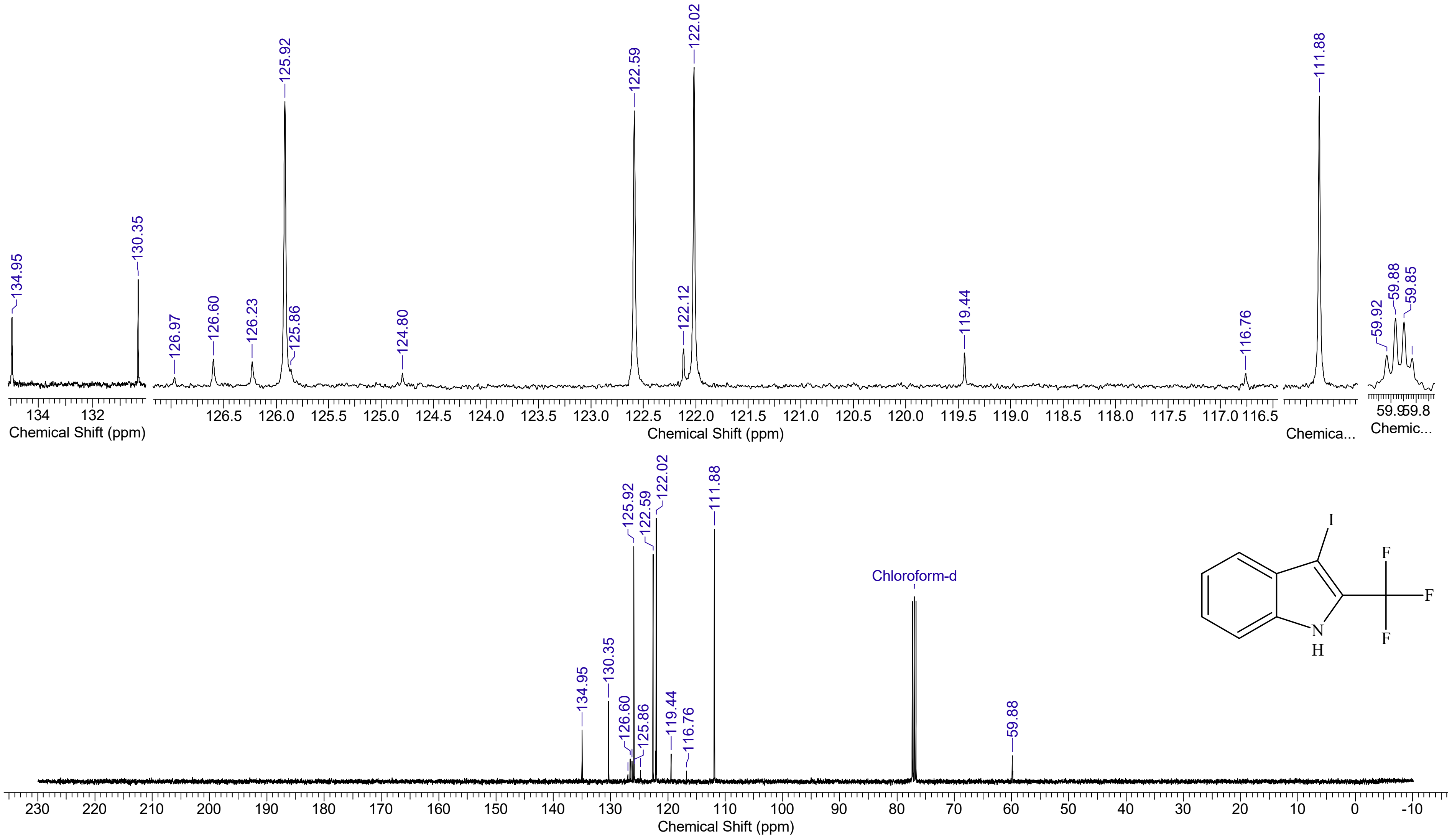

$^{13}C\{^1H\}$ NMR spectrum of **3c** (100.6 MHz, $CDCl_3$)

1 Aug 2021

**Acquisition Time (sec)**	4.0894	**Comment**	Imported from UXNMR.			**Date**	01 Jun 2021 14:28:04
**File Name**	C:\DOCS\OUTPUT_301\2021\06.июнь\BM-2170-3.H_001001r					**Frequency (MHz)**	400.13
**Nucleus**	1H	**Number of Transients**	4	**Original Points Count**	32768	**Points Count**	131072
**Pulse Sequence**	zg30	**Solvent**	CHLOROFORM-D			**Sweep Width (Hz)**	8012.82
**Temperature (degree C)**	27.000						

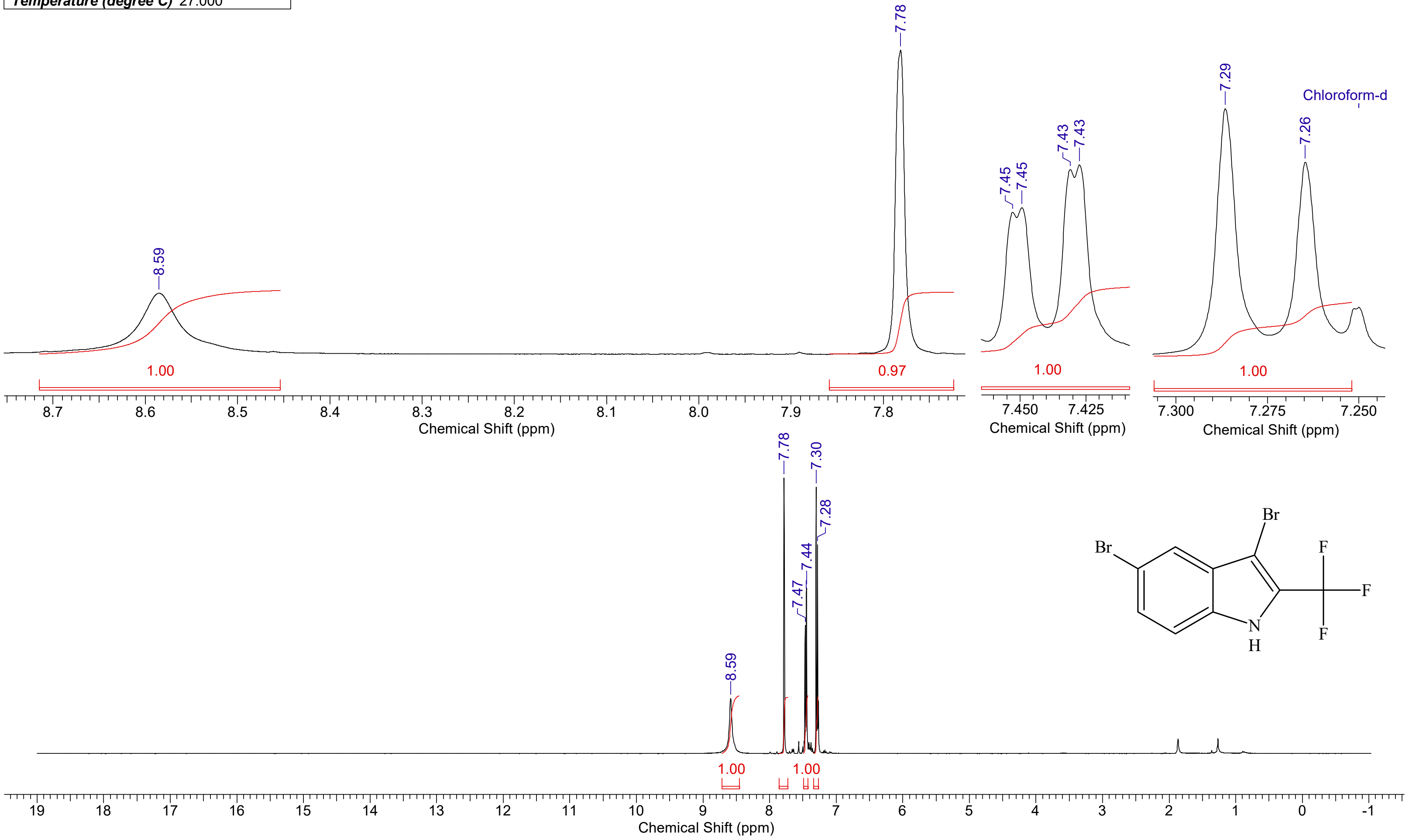

^1H NMR spectrum of **4** (400.1 MHz, $CDCl_3$)

1 Aug 2021

Acquisition Time (sec)	1.7433	***Comment***	Imported from UXNMR.			***Date***	01 Jun 2021 15:02:16
File Name	C:\DOCS\OUTPUT_301\2021\06.июнь\BM-2170-3.F_005001r					***Frequency (MHz)***	376.50
Nucleus	19F	***Number of Transients***	16	***Original Points Count***	131072	***Points Count***	262144
Pulse Sequence	zgflqn	***Solvent***	DMSO-D6	***Sweep Width (Hz)***	75187.97	***Temperature (degree C)***	27.000

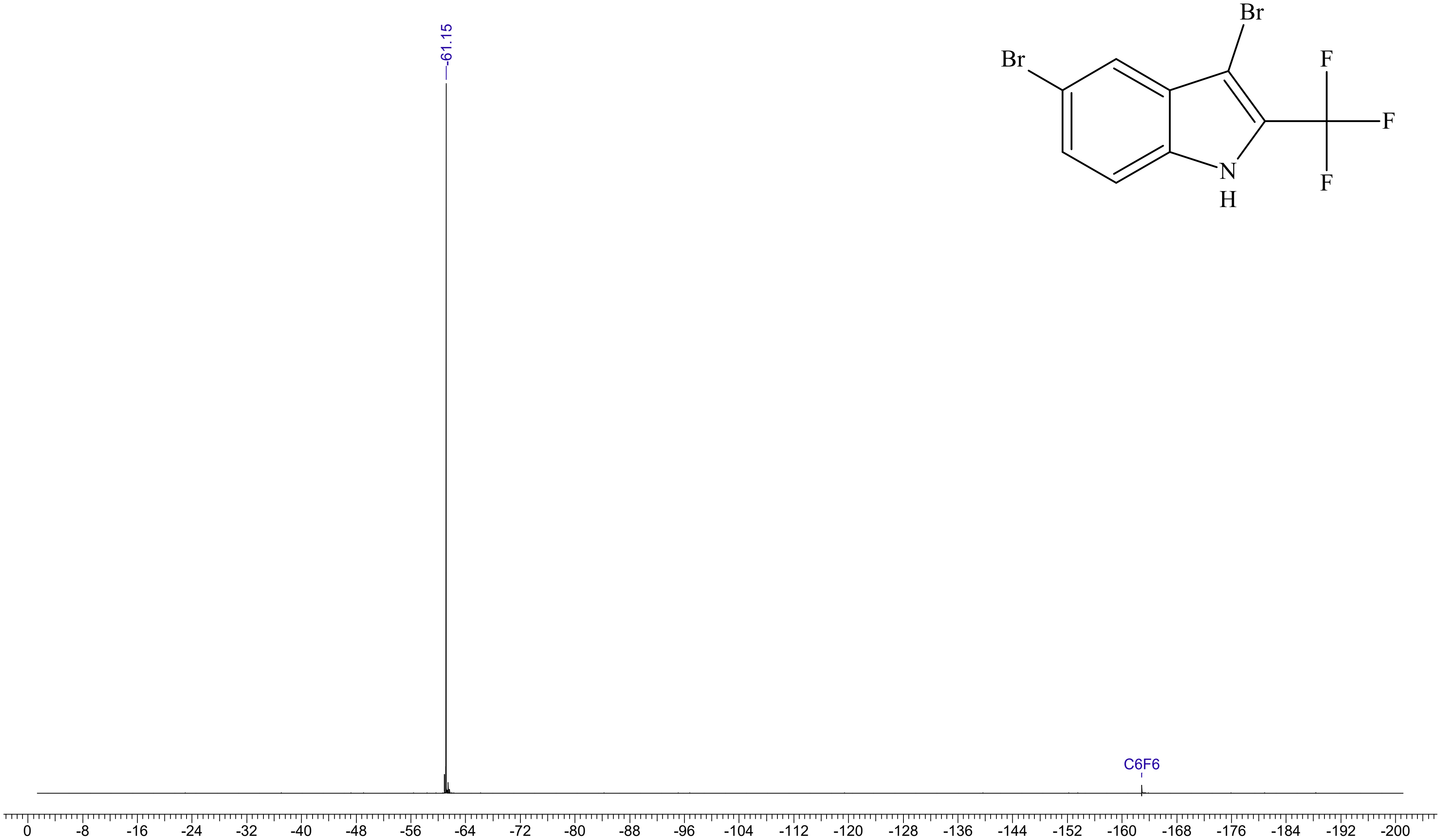

^{19}F NMR spectrum of **4** (376.5 MHz, $CDCl_3$)

1 Aug 2021

Acquisition Time (sec)	0.6783	***Comment***	Imported from UXNMR.			***Date***	01 Jun 2021 14:57:58
File Name	C:\DOCS\OUTPUT_301\2021\06.июнь\BM-2170-3.C_002001r					***Frequency (MHz)***	100.61
Nucleus	13C	***Number of Transients***	753	***Original Points Count***	16384	***Points Count***	131072
Pulse Sequence	zgpg30	***Solvent***	DMSO-D6	***Sweep Width (Hz)***	24154.59	***Temperature (degree C)***	27.000

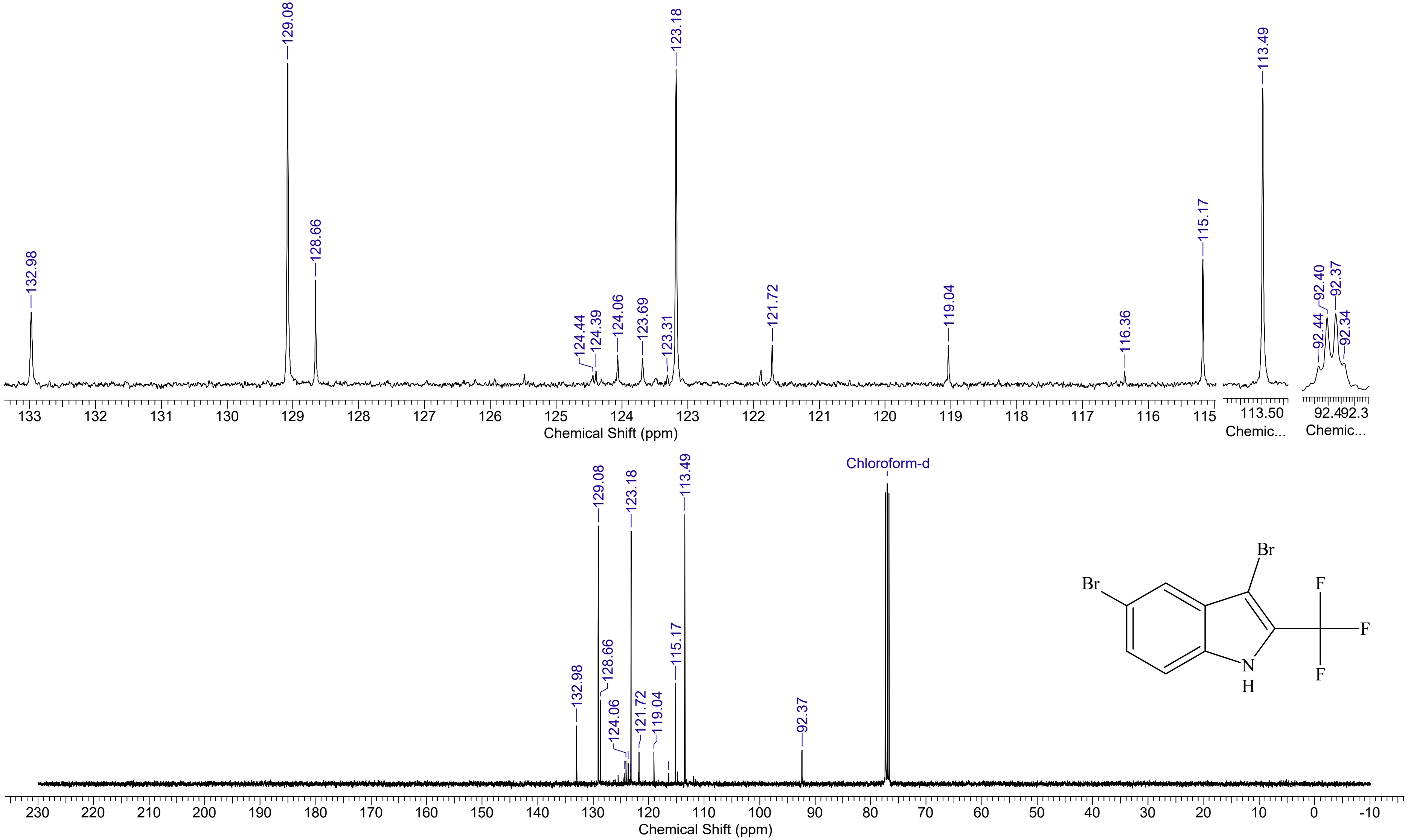

$^{13}C\{^1H\}$ NMR spectrum of **4** (100.6 MHz, $CDCl_3$).

7 Oct 2022

Acquisition Time (sec)	4.0894	***Comment***	Imported from UXNMR.			***Date***	22 Sep 2022 12:54:34
File Name	C:\DOCS\OUTPUT_301\2022\09.сентябрь\SZA-BM-2608.H_001001r					***Frequency (MHz)***	400.13
Nucleus	1H	***Number of Transients***	4	***Original Points Count***	32768	***Points Count***	131072
Pulse Sequence	zg30	***Solvent***	CHLOROFORM-D			***Sweep Width (Hz)***	8012.82
Temperature (degree C)	27.000						

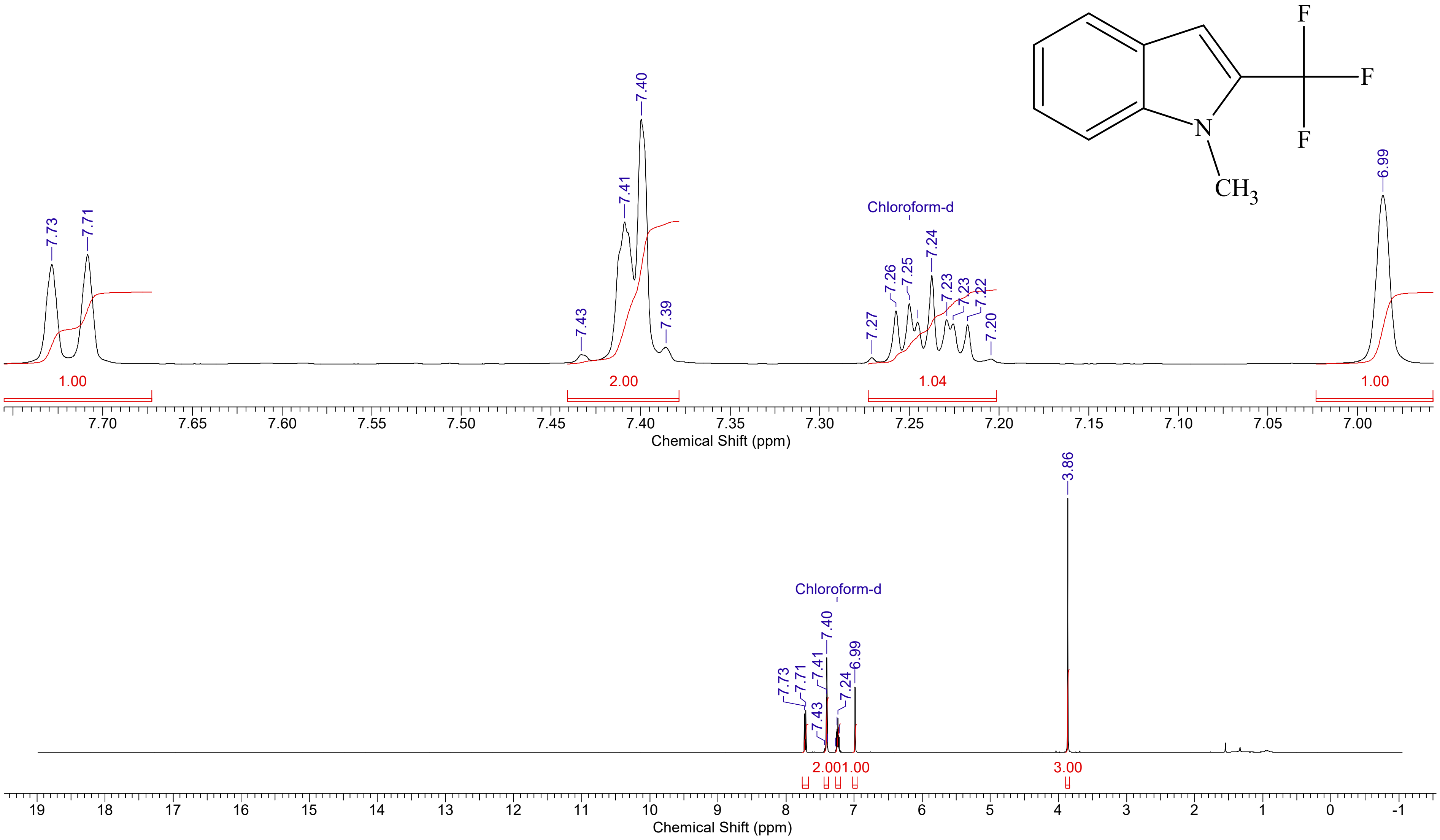

^{1}H NMR spectrum of **5a** (400.1 MHz, $CDCl_3$)

7 Oct 2022

Acquisition Time (sec)	1.7433	***Comment***	Imported from UXNMR.			***Date***	22 Sep 2022 12:52:36
File Name	C:\DOCS\OUTPUT_301\2022\09.сентябрь\SZA-BM-2608.F_005001r					***Frequency (MHz)***	376.50
Nucleus	19F	***Number of Transients***	16	***Original Points Count***	131072	***Points Count***	262144
Pulse Sequence	zgflqn	***Solvent***	CHLOROFORM-D			***Sweep Width (Hz)***	75187.97
Temperature (degree C)	27.000						

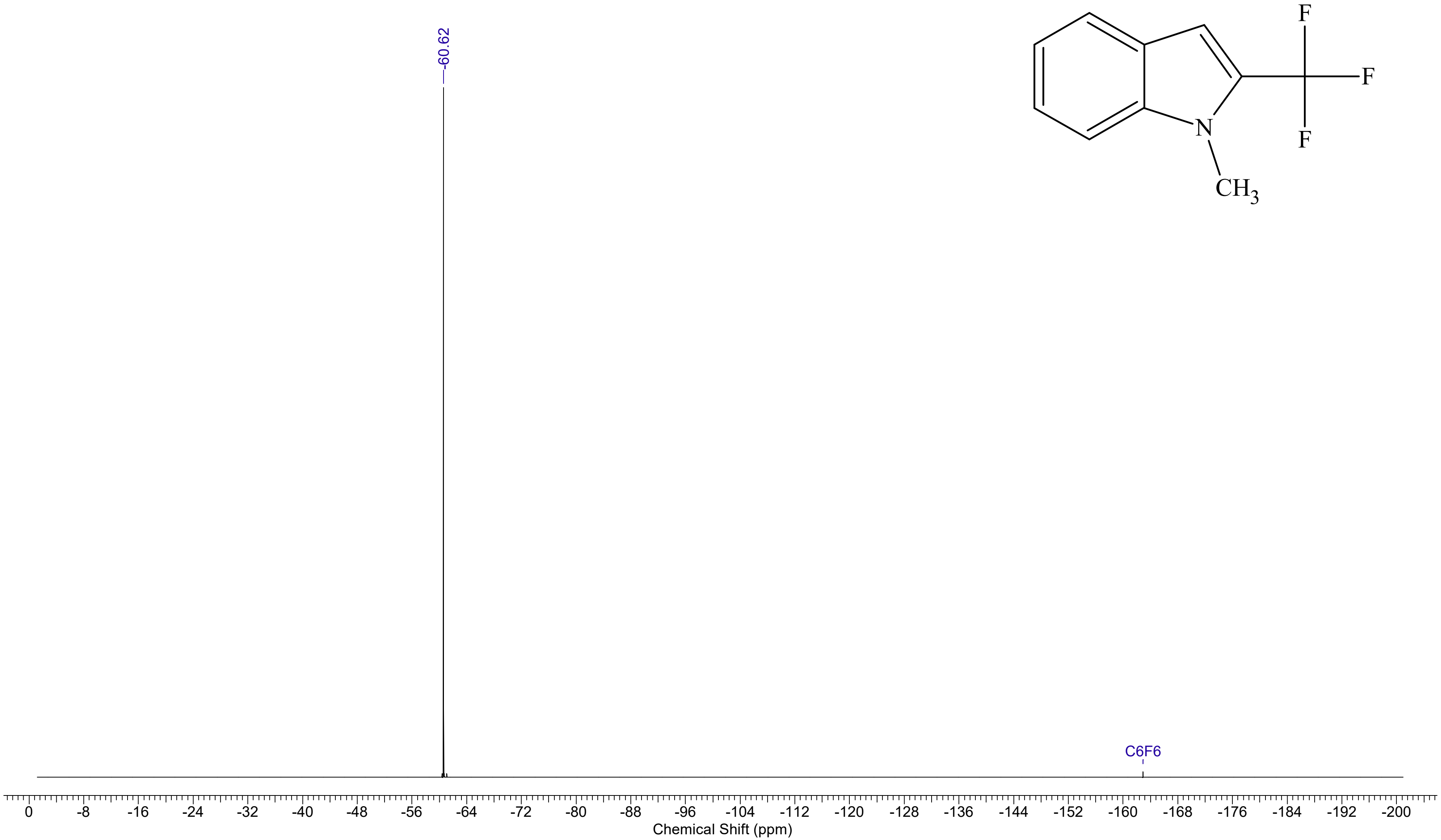

^{19}F NMR spectrum of **5a** (376.5 MHz, $CDCl_3$)

7 Oct 2022

Acquisition Time (sec)	0.6783	Comment	Imported from UXNMR.			Date	22 Sep 2022 13:07:46
File Name	C:\DOCS\OUTPUT_301\2022\09.сентябрь\SZA-BM-2608.C_002001r					Frequency (MHz)	100.61
Nucleus	13C	Number of Transients	249	Original Points Count	16384	Points Count	131072
Pulse Sequence	zgpg30	Solvent	CHLOROFORM-D			Sweep Width (Hz)	24154.59
Temperature (degree C)	27.000						

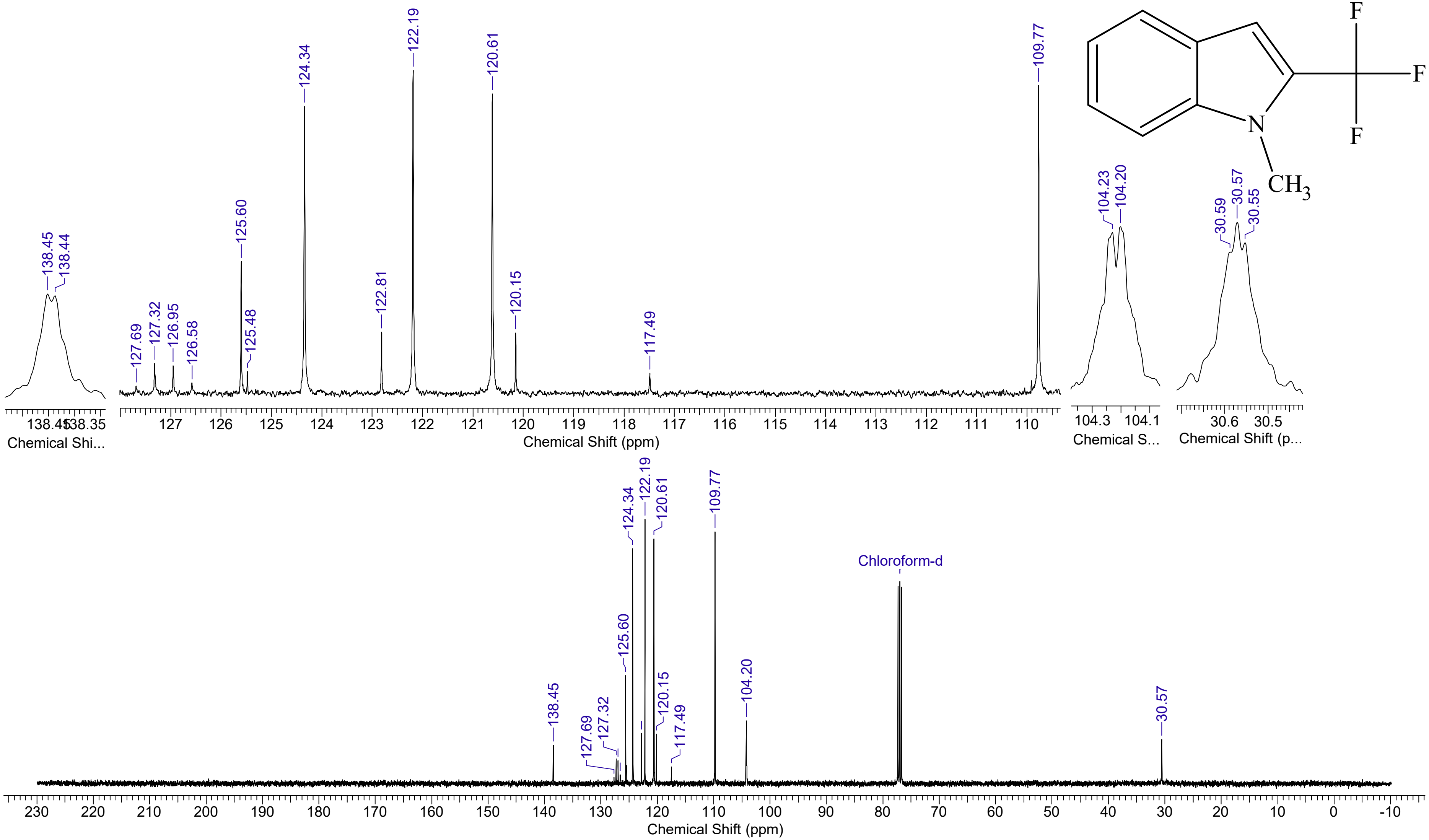

$^{13}C\{^1H\}$ NMR spectrum of **5a** (100.6 MHz, $CDCl_3$)

7 Oct 2022

Acquisition Time (sec)	4.0894	***Comment***	Imported from UXNMR.		***Date***	24 Sep 2022 22:33:54
File Name	C:\DOCS\BM\ЯМР_СОСЕДИ\2022\220924\BM-2614_001001r				***Frequency (MHz)***	400.13
Nucleus	1H	***Number of Transients***	8	***Original Points Count*** 32768	***Points Count***	131072
Pulse Sequence	zg30	***Solvent***	CHLOROFORM-D		***Sweep Width (Hz)***	8012.82
Temperature (degree C)	27.000					

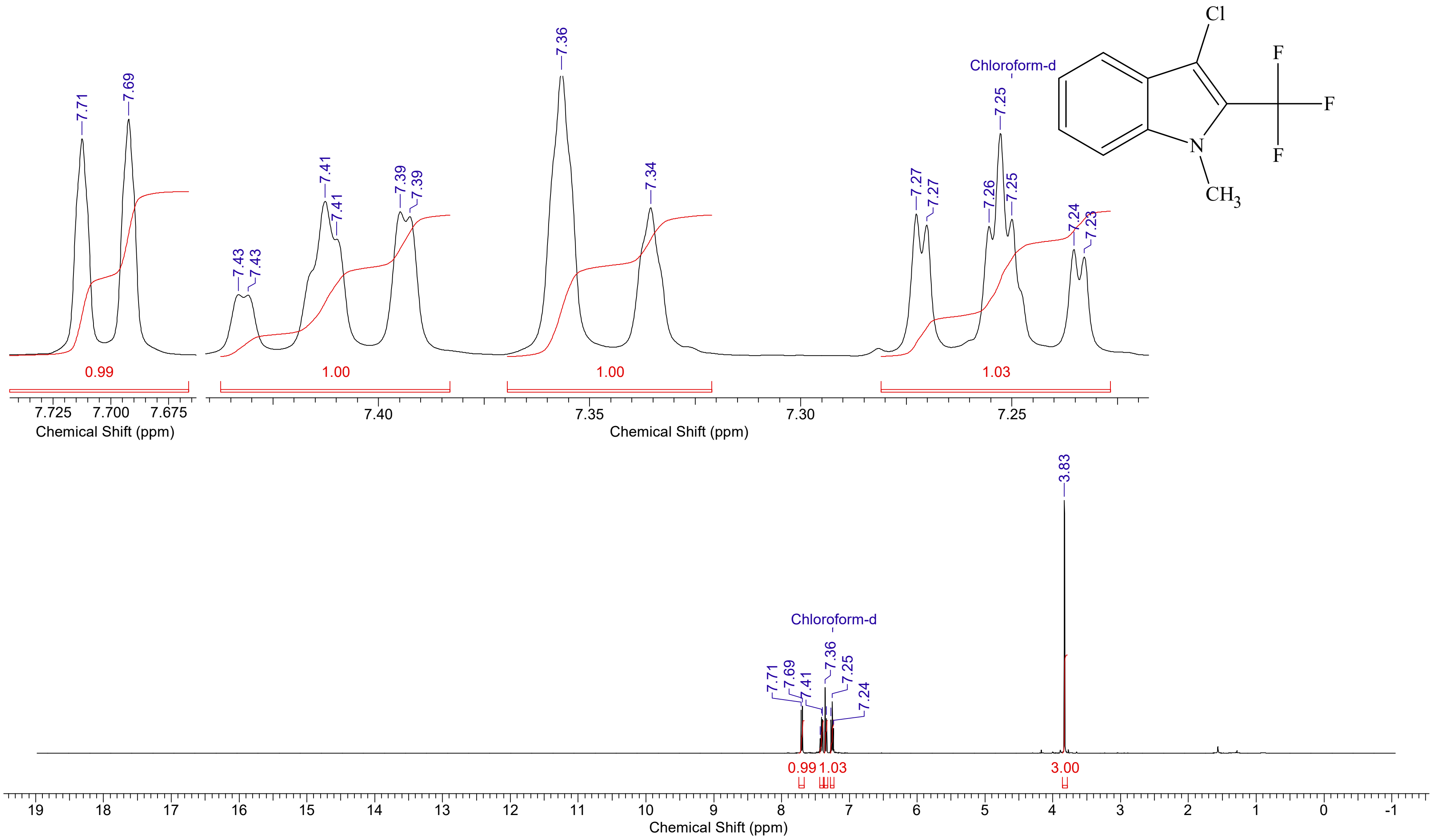

^{1}H NMR spectrum of **5b** (400.1 MHz, $CDCl_3$)

7 Oct 2022

Acquisition Time (sec)	1.7433	***Comment***	Imported from UXNMR.			***Date***	24 Sep 2022 22:37:00
File Name	C:\DOCS\BM\ЯМР_СОСЕДИ\2022\220924\BM-2614_005001r					***Frequency (MHz)***	376.50
Nucleus	19F	***Number of Transients***	16	***Original Points Count***	131072	***Points Count***	262144
Pulse Sequence	zgflqn	***Solvent***	CHLOROFORM-D			***Sweep Width (Hz)***	75187.97
Temperature (degree C)	27.000						

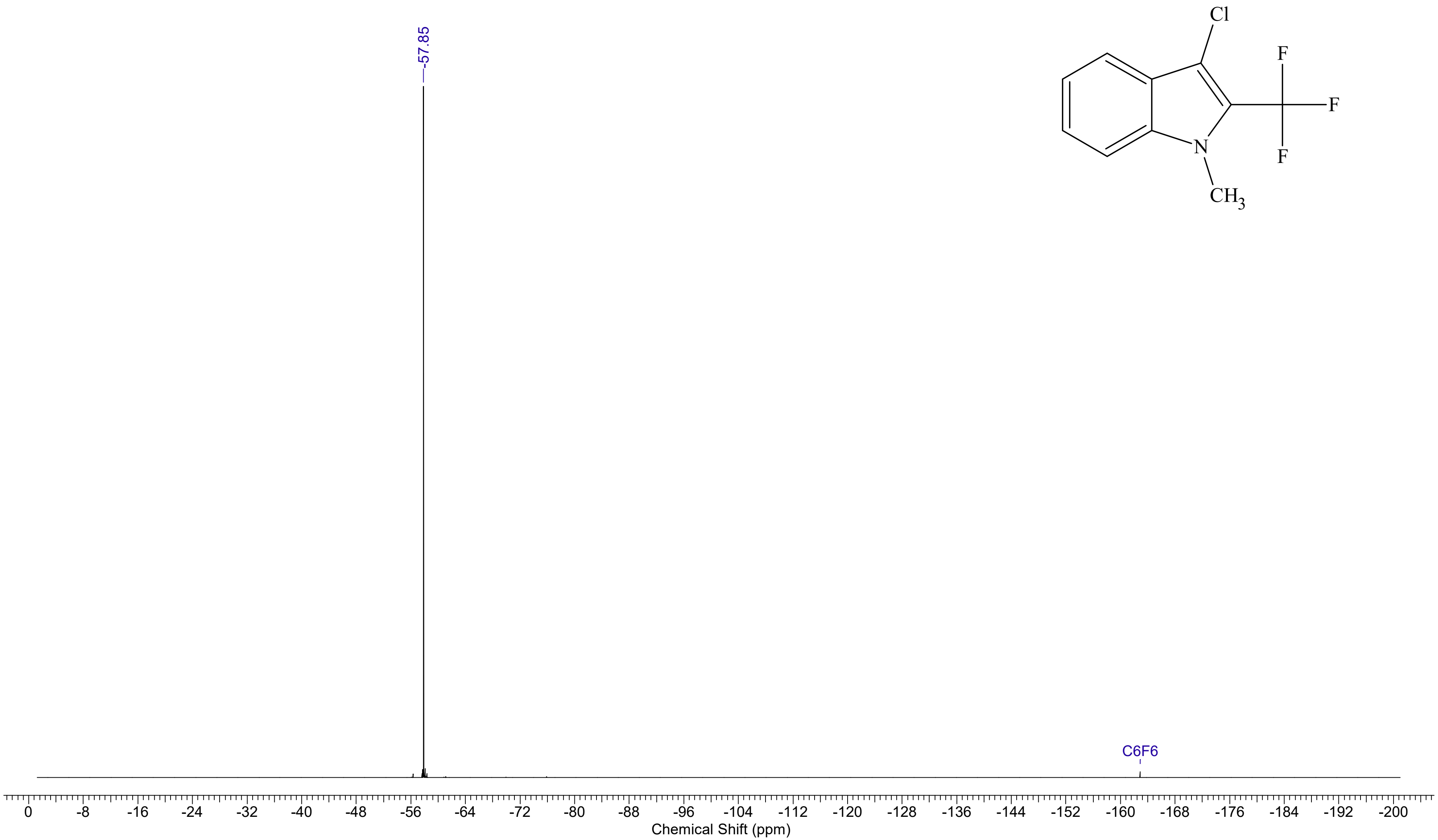

^{19}F NMR spectrum of **5b** (376.5 MHz, $CDCl_3$)

Acquisition Time (sec)	0.6783	***Comment***	Imported from UXNMR.			***Date***	27 Sep 2022 15:36:38
File Name	C:\DOCS\OUTPUT_301\2022\09.сентябрь\BM-2614.C_002001r					***Frequency (MHz)***	100.61
Nucleus	13C	***Number of Transients***	177	***Original Points Count***	16384	***Points Count***	131072
Pulse Sequence	zgpg30	***Solvent***	CHLOROFORM-D			***Sweep Width (Hz)***	24154.59
Temperature (degree C)	27.000						

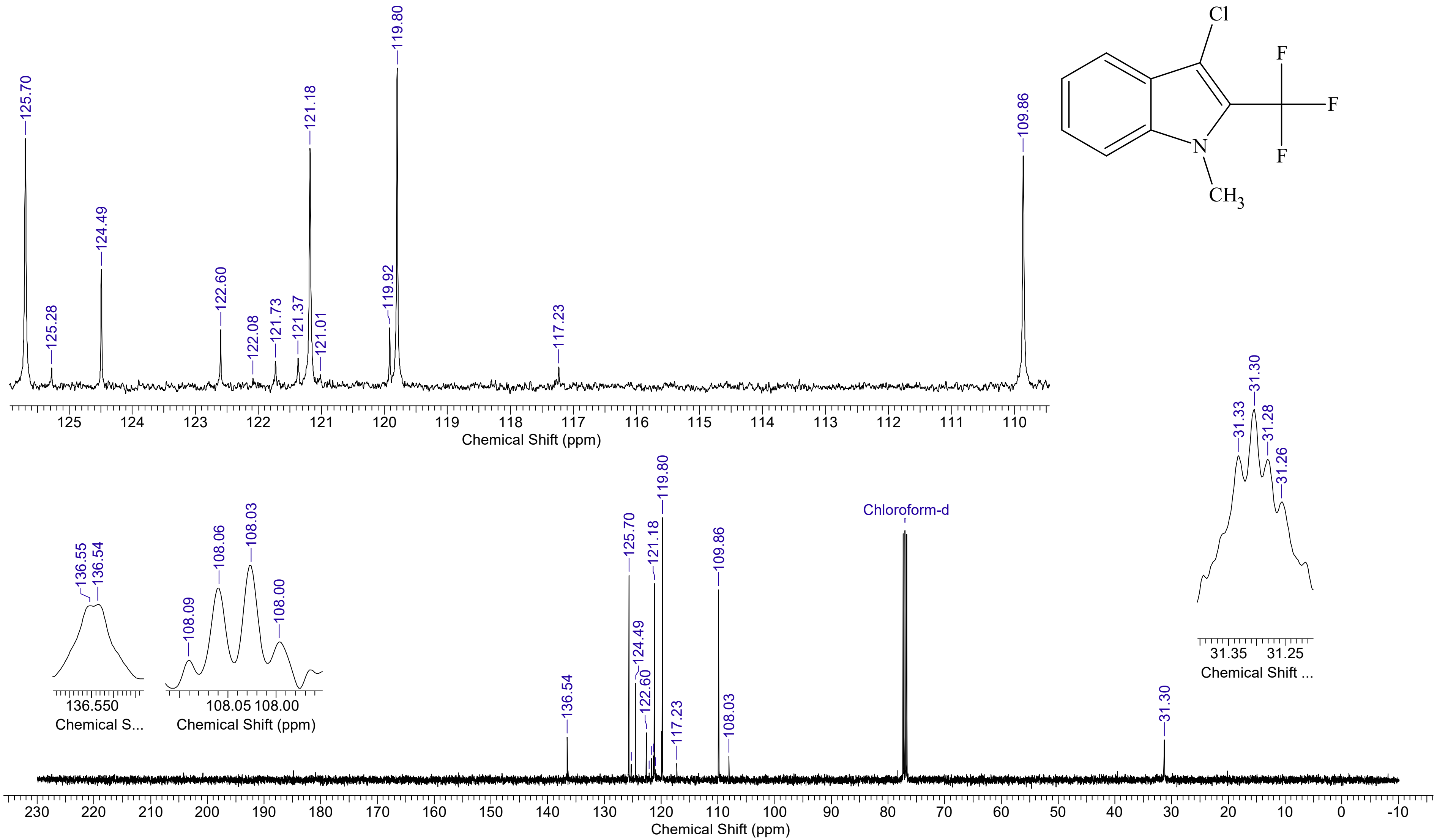

$^{13}C\{^1H\}$ NMR spectrum of **5b** (100.6 MHz, $CDCl_3$)

8 Oct 2022

Acquisition Time (sec)	4.0894	***Comment***	Imported from UXNMR.			***Date***	20 Sep 2022 14:51:44
File Name	C:\DOCS\OUTPUT_301\2022\09.сентябрь\BM-2607-2.H_001001r					***Frequency (MHz)***	400.13
Nucleus	1H	***Number of Transients***	4	***Original Points Count***	32768	***Points Count***	131072
Pulse Sequence	zg30	***Solvent***	CHLOROFORM-D			***Sweep Width (Hz)***	8012.82
Temperature (degree C)	27.000						

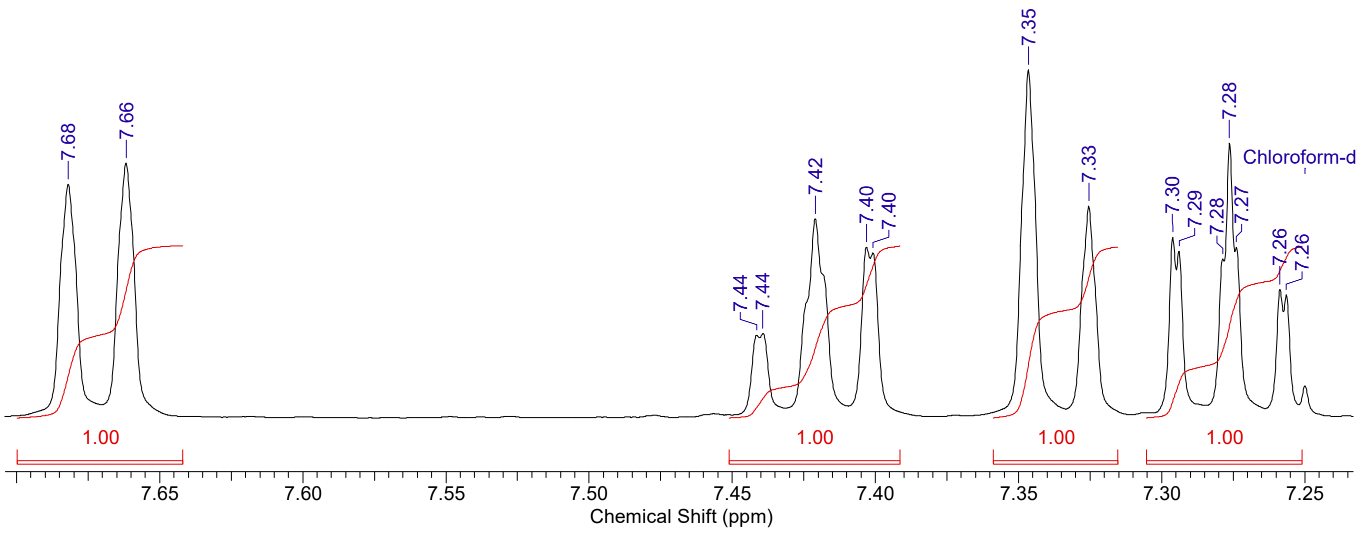

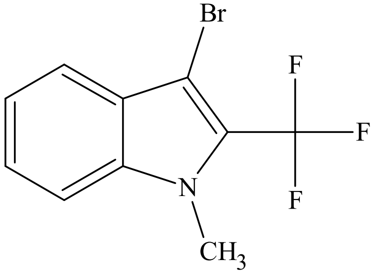

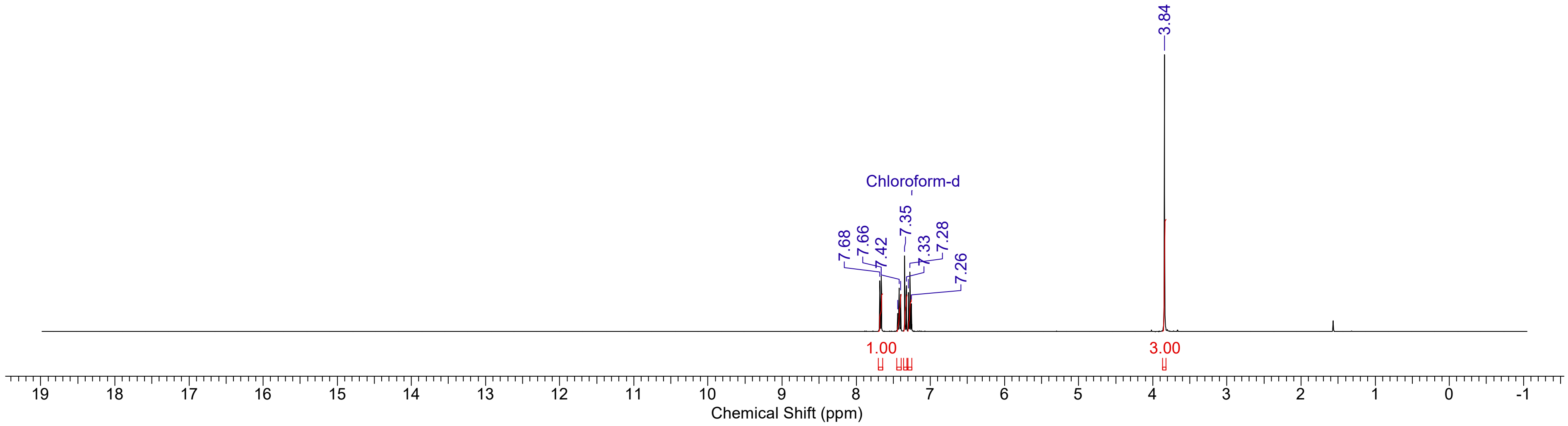

^{1}H NMR spectrum of **5c** (400.1 MHz, $CDCl_3$)

8 Oct 2022

Acquisition Time (sec)	1.7433	***Comment***	Imported from UXNMR.			***Date***	20 Sep 2022 15:39:04
File Name	C:\DOCS\OUTPUT_301\2022\09.сентябрь\BM-2607-2.F_005001r					***Frequency (MHz)***	376.50
Nucleus	19F	***Number of Transients***	16	***Original Points Count***	131072	***Points Count***	262144
Pulse Sequence	zgflqn	***Solvent***	CHLOROFORM-D			***Sweep Width (Hz)***	75187.97
Temperature (degree C)	27.000						

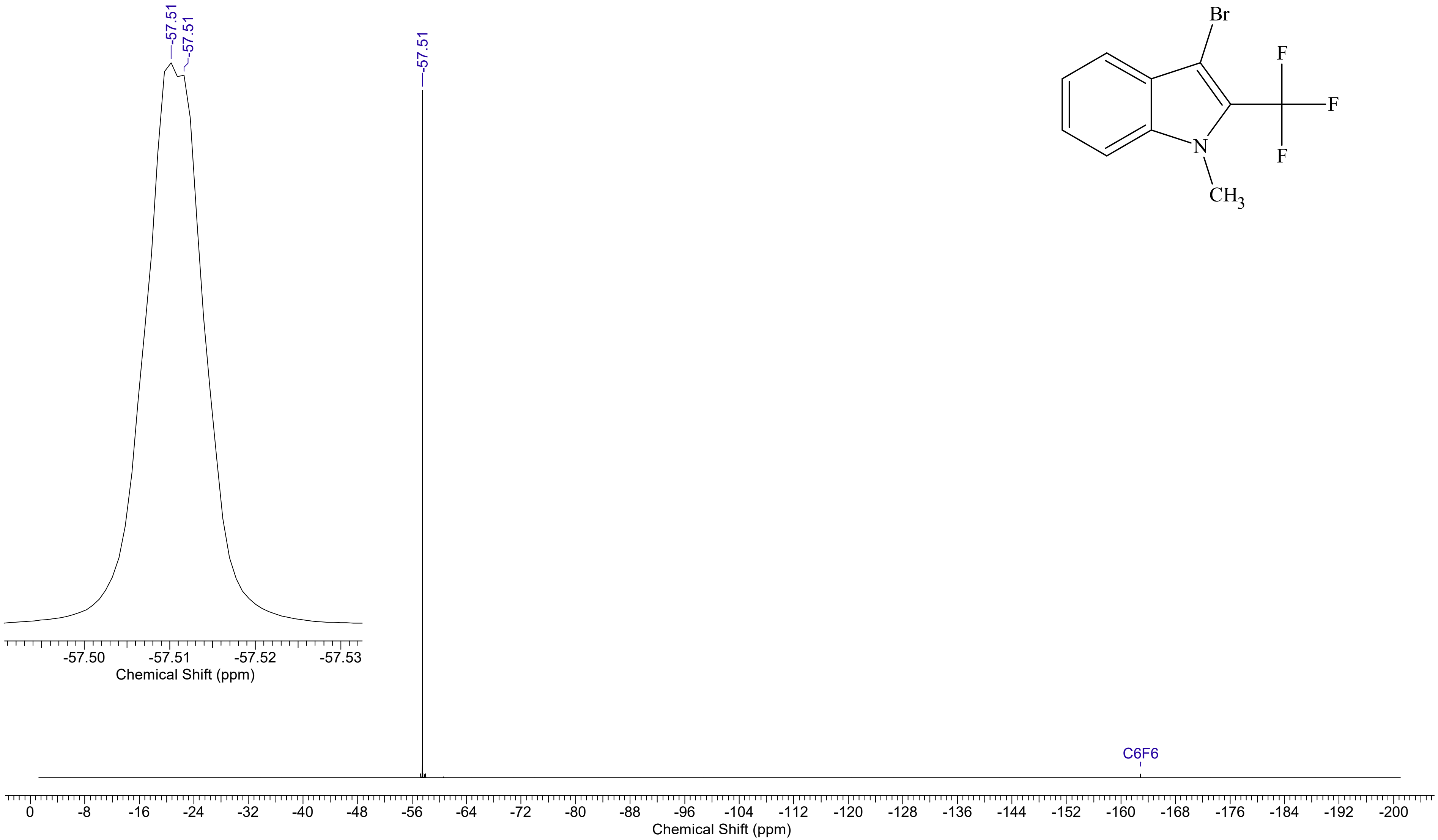

^{19}F NMR spectrum of **5c** (376.5 MHz, $CDCl_3$)

8 Oct 2022

Acquisition Time (sec)	0.6783	***Comment***	Imported from UXNMR.			***Date***	20 Sep 2022 14:49:34
File Name	C:\DOCS\OUTPUT_301\2022\09.сентябрь\BM-2607-2.C_002001r					***Frequency (MHz)***	100.61
Nucleus	13C	***Number of Transients***	236	***Original Points Count***	16384	***Points Count***	131072
Pulse Sequence	zgpg30	***Solvent***	CHLOROFORM-D			***Sweep Width (Hz)***	24154.59
Temperature (degree C)	27.000						

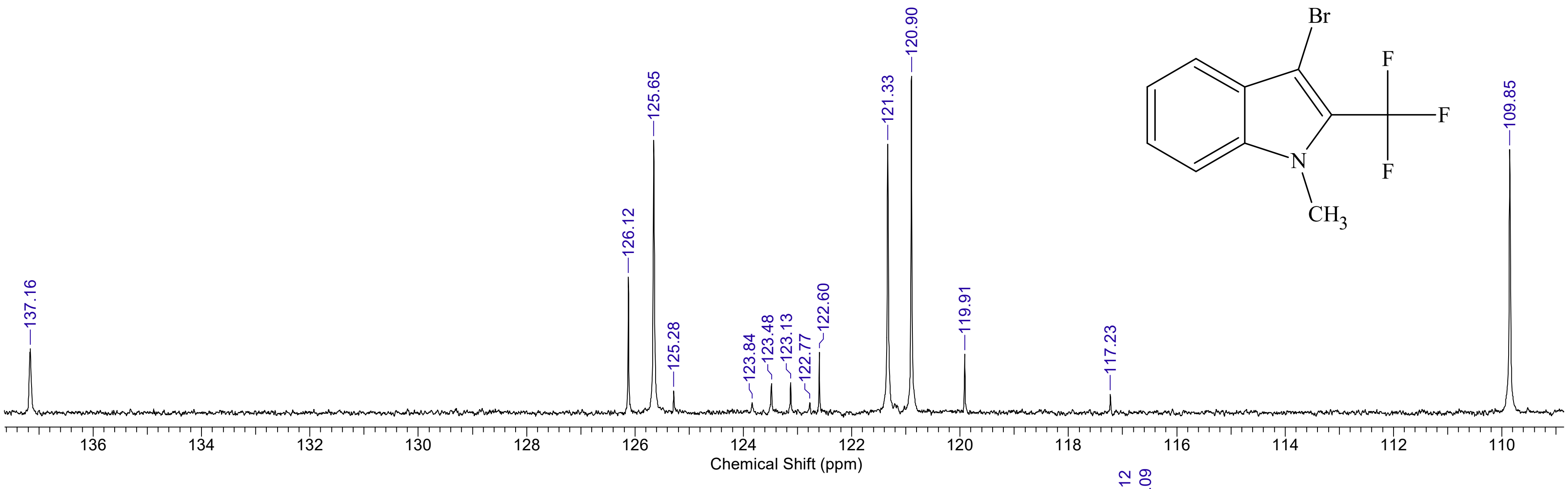

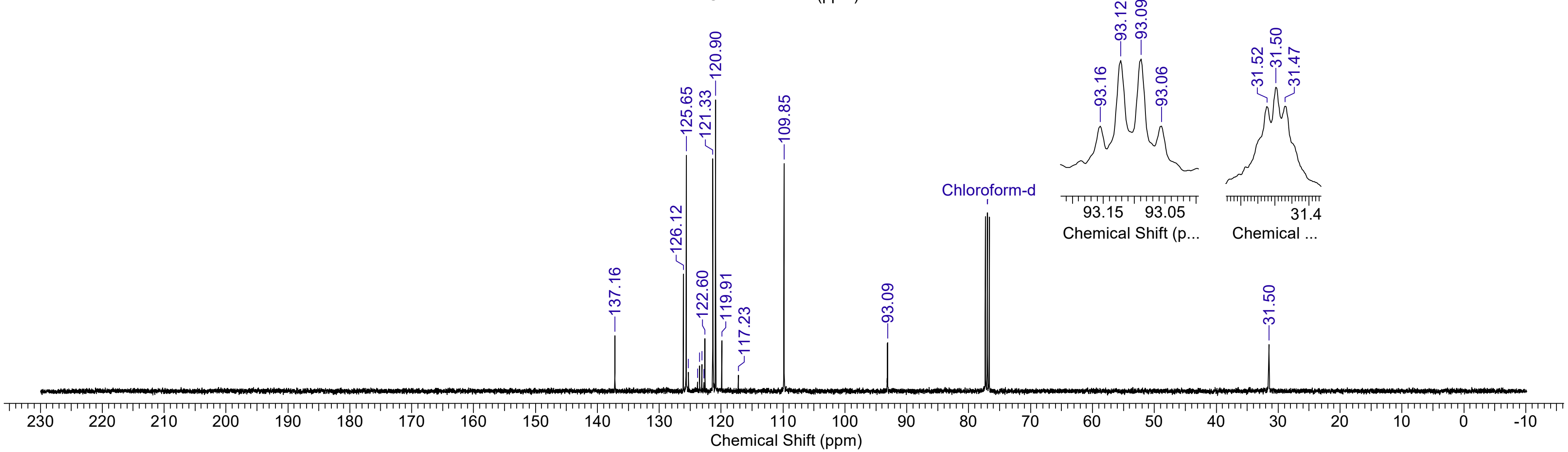

$^{13}C\{^{1}H\}$ NMR spectrum of **5c** (100.6 MHz, $CDCl_3$)

8 Oct 2022

Acquisition Time (sec)	4.0894	Comment	Imported from UXNMR.			Date	20 Sep 2022 14:20:46
File Name	C:\DOCS\OUTPUT_301\2022\09.сентябрь\BM-2605-2.H_001001r					Frequency (MHz)	400.13
Nucleus	1H	Number of Transients	4	Original Points Count	32768	Points Count	131072
Pulse Sequence	zg30	Solvent	CHLOROFORM-D			Sweep Width (Hz)	8012.82
Temperature (degree C)	27.000						

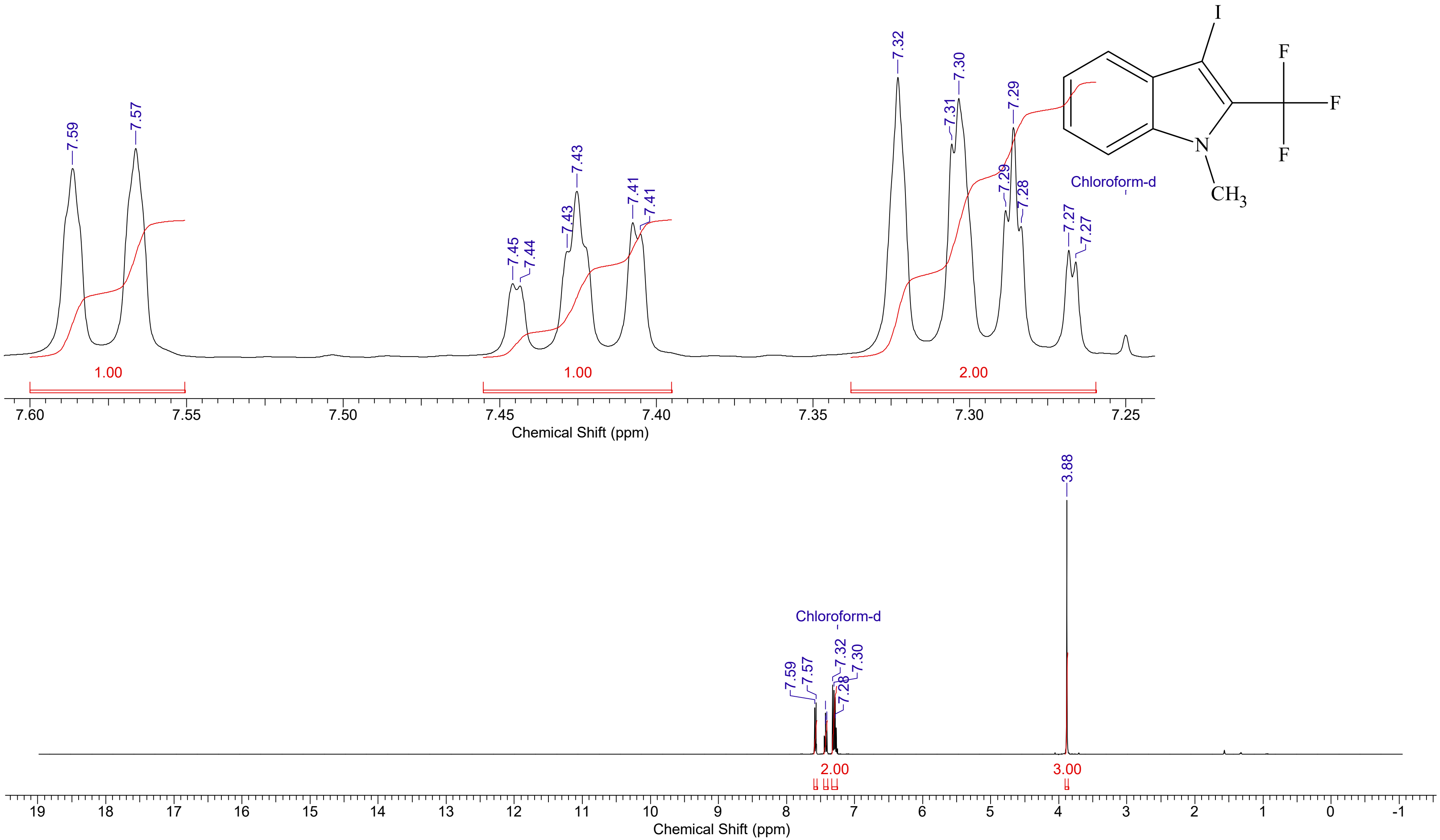

^{1}H NMR spectrum of **5d** (400.1 MHz, $CDCl_3$)

8 Oct 2022

Acquisition Time (sec)	1.7433	Comment	Imported from UXNMR.			Date	20 Sep 2022 15:36:38
File Name	C:\DOCS\OUTPUT_301\2022\09.сентябрь\BM-2605-2.F_005001r					Frequency (MHz)	376.50
Nucleus	19F	Number of Transients	16	Original Points Count	131072	Points Count	262144
Pulse Sequence	zgflqn	Solvent	CHLOROFORM-D			Sweep Width (Hz)	75187.97
Temperature (degree C)	27.000						

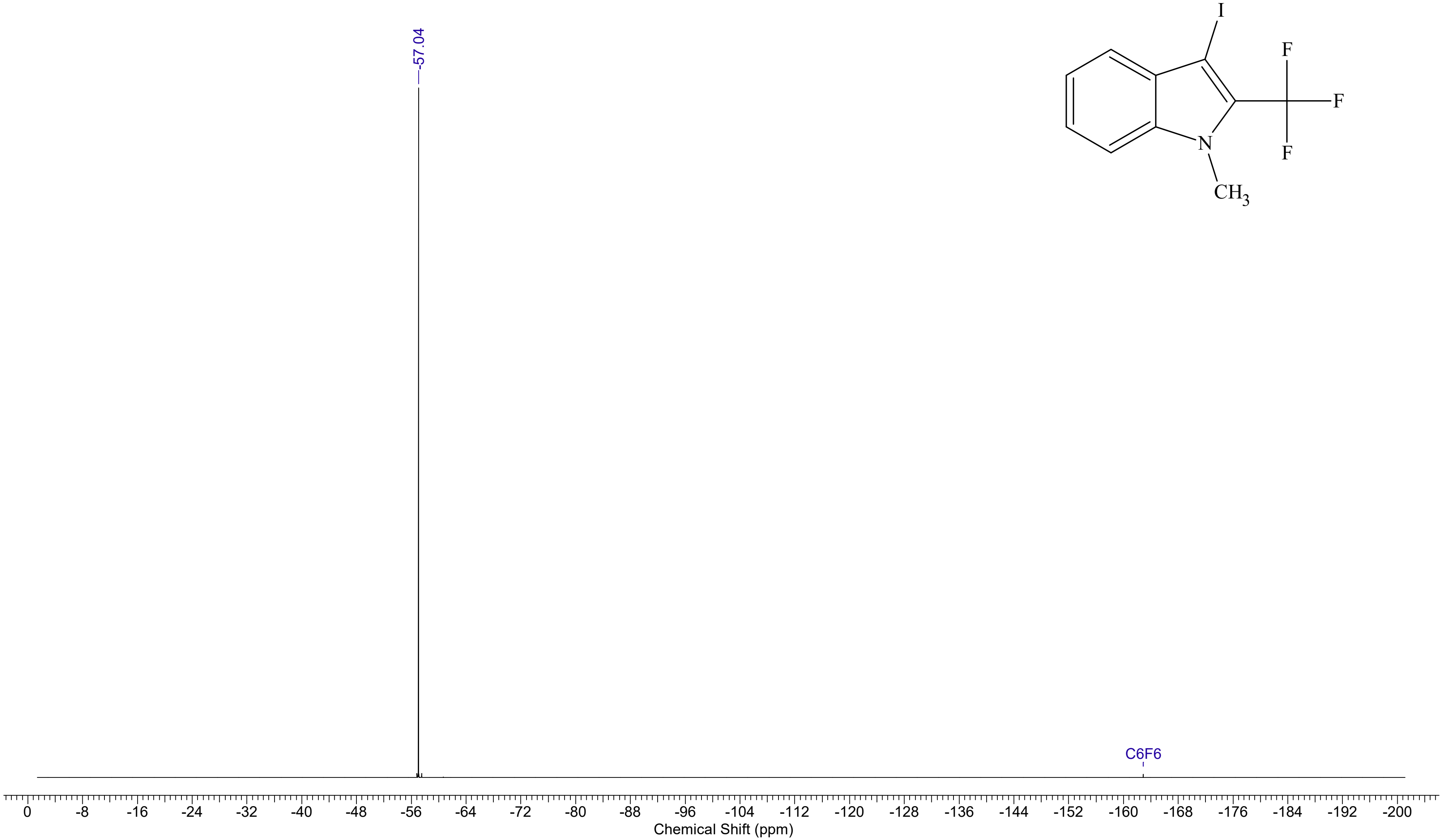

^{19}F NMR spectrum of **5d** (376.5 MHz, $CDCl_3$)

8 Oct 2022

Acquisition Time (sec)	0.6783	***Comment***	Imported from UXNMR.			***Date***	20 Sep 2022 14:25:20
File Name	C:\DOCS\OUTPUT_301\2022\09.сентябрь\BM-2605-2.C_002001r					***Frequency (MHz)***	100.61
Nucleus	13C	***Number of Transients***	98	***Original Points Count***	16384	***Points Count***	131072
Pulse Sequence	zgpg30	***Solvent***	CHLOROFORM-D			***Sweep Width (Hz)***	24154.59
Temperature (degree C)	27.000						

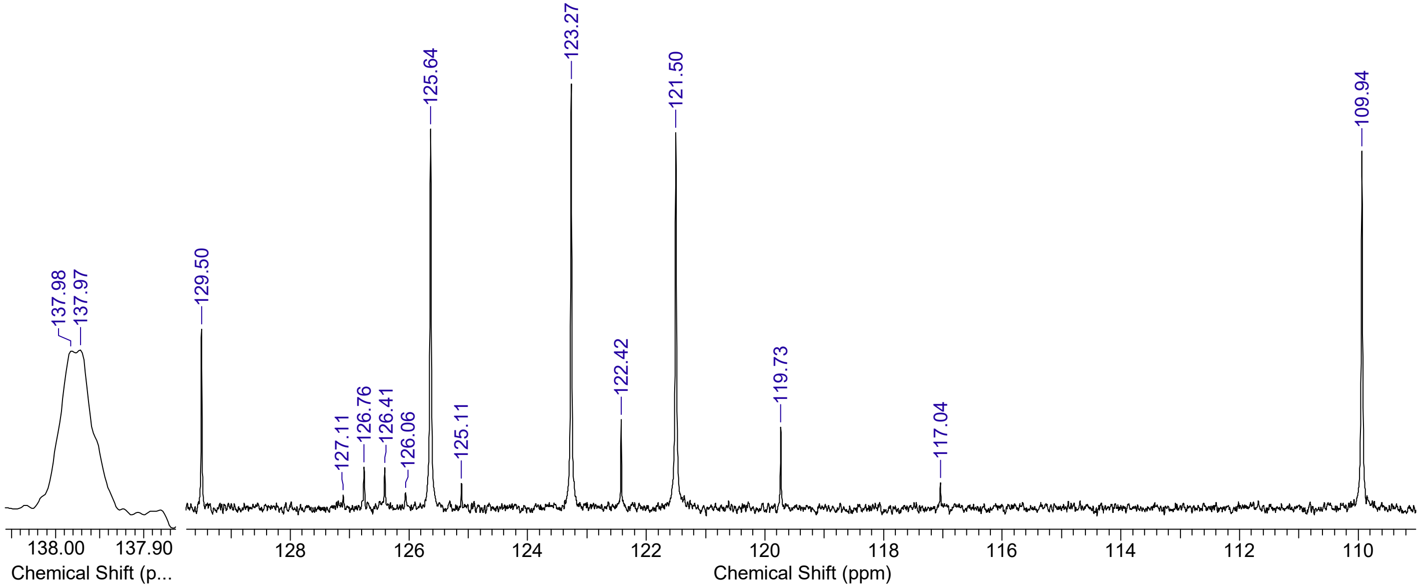

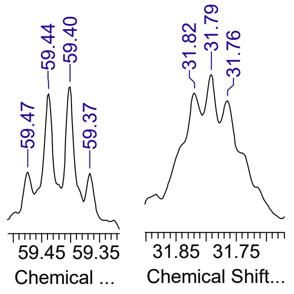

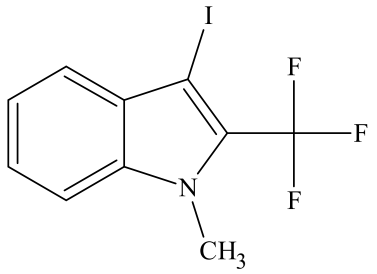

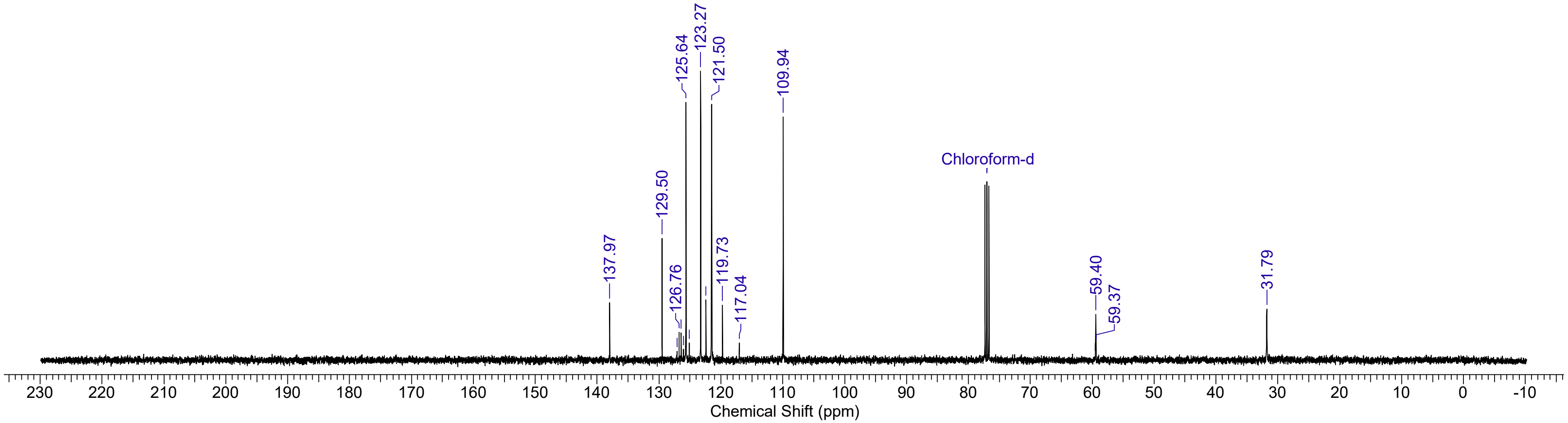

$^{13}C\{^{1}H\}$ NMR spectrum of **5d** (100.6 MHz, $CDCl_3$)

7 Oct 2022

Acquisition Time (sec)	4.0894	***Comment***	Imported from UXNMR.			***Date***	19 Sep 2022 15:43:18
File Name	C:\DOCS\OUTPUT_301\2022\09.сентябрь\SZA-BM-2590-2-4.H_001001r					***Frequency (MHz)***	400.13
Nucleus	1H	***Number of Transients***	4	***Original Points Count***	32768	***Points Count***	131072
Pulse Sequence	zg30	***Solvent***	CHLOROFORM-D			***Sweep Width (Hz)***	8012.82
Temperature (degree C)	27.000						

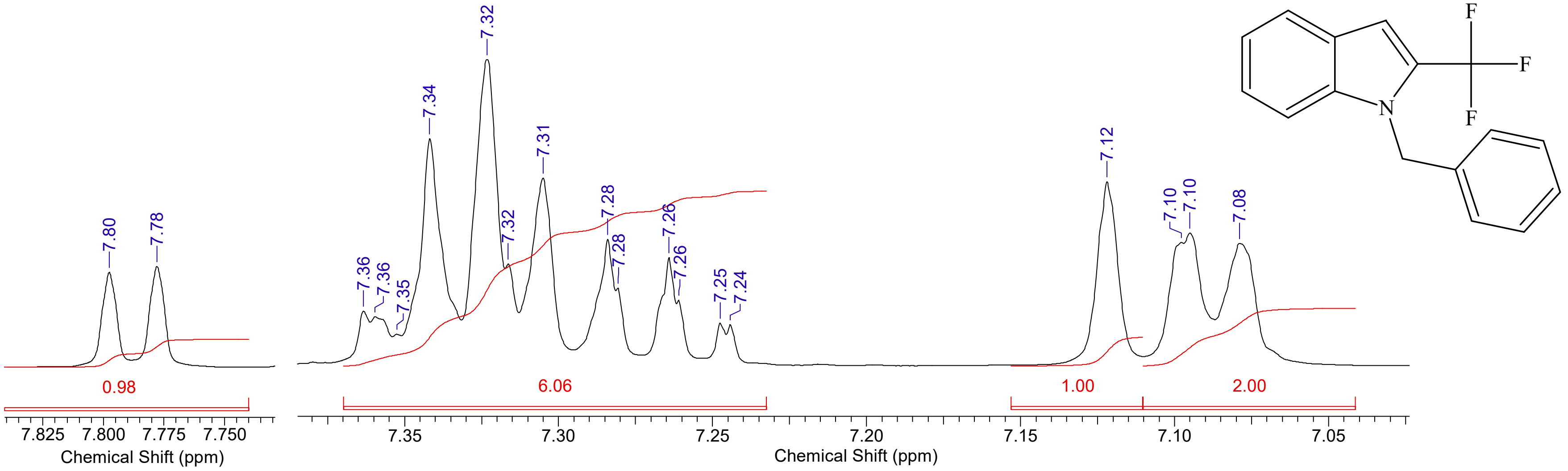

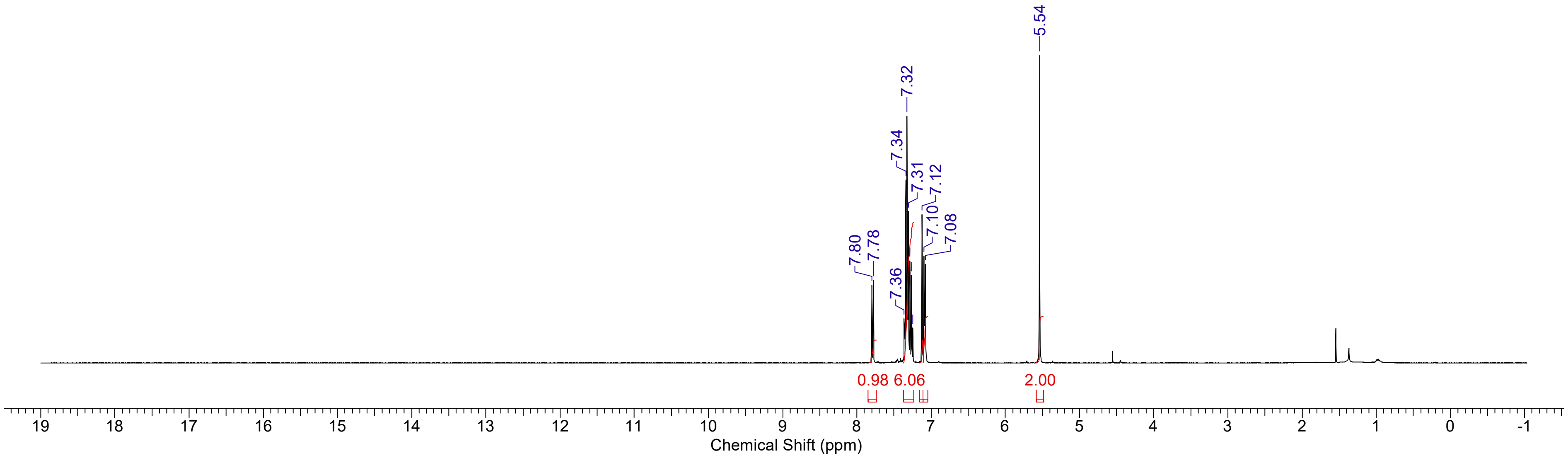

^{1}H NMR spectrum of **6a** (400.1 MHz, $CDCl_3$)

Acquisition Time (sec)	1.7433	***Comment***	Imported from UXNMR.			***Date***	19 Sep 2022 15:41:38
File Name	C:\DOCS\OUTPUT_301\2022\09.сентябрь\SZA-BM-2590-2-4.F_005001r					***Frequency (MHz)***	376.50
Nucleus	19F	***Number of Transients***	16	***Original Points Count***	131072	***Points Count***	262144
Pulse Sequence	zgflqn	***Solvent***	CHLOROFORM-D			***Sweep Width (Hz)***	75187.97
Temperature (degree C)	27.000						

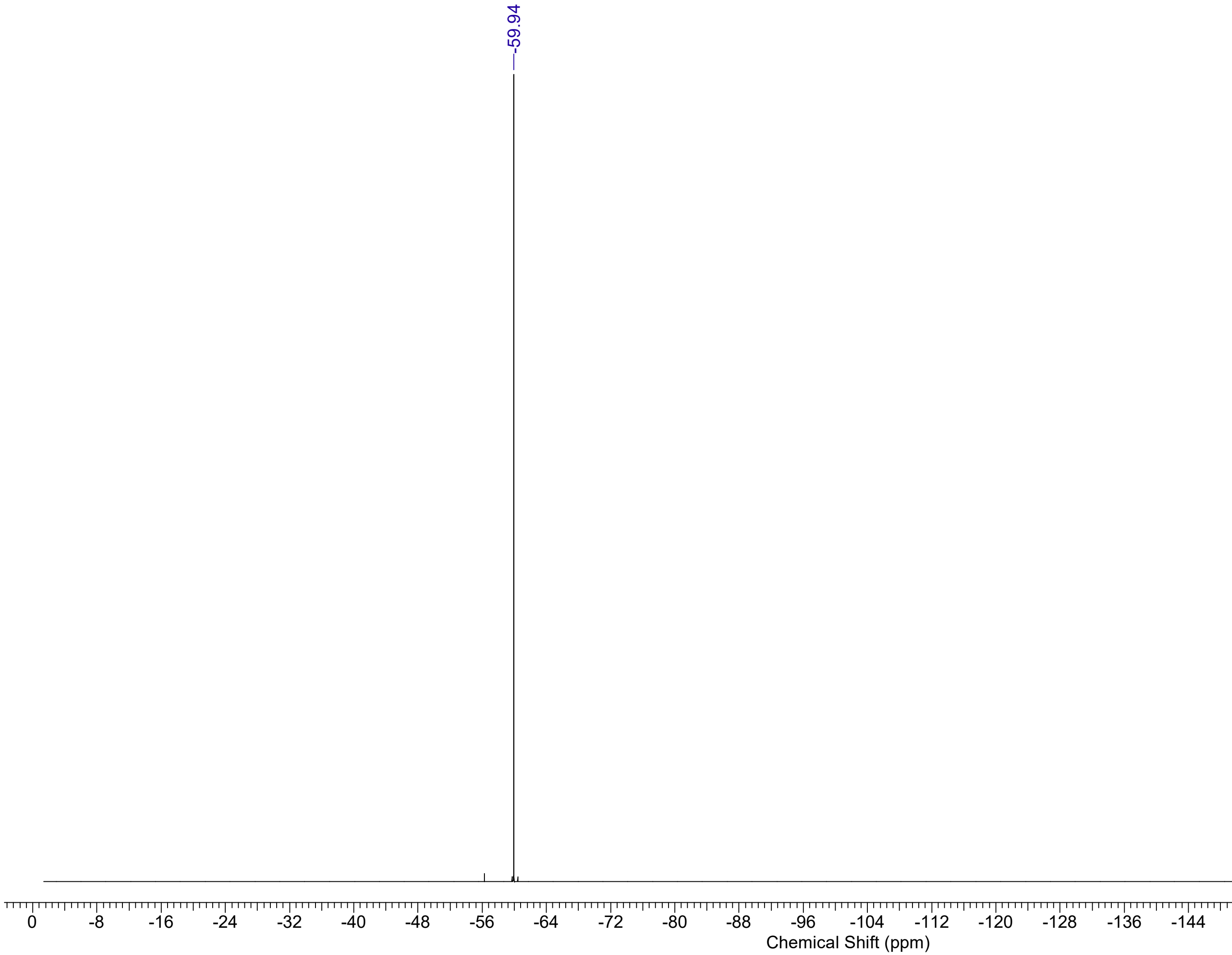

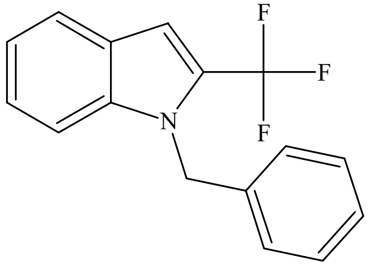

^{19}F NMR spectrum of **6a** (376.5 MHz, $CDCl_3$)

7 Oct 2022

Acquisition Time (sec)	0.6783	***Comment***	Imported from UXNMR.			***Date***	20 Sep 2022 14:38:40
File Name	C:\DOCS\OUTPUT_301\2022\09.сентябрь\SZA-BM-2590-2-4.C_002001r					***Frequency (MHz)***	100.61
Nucleus	13C	***Number of Transients***	249	***Original Points Count***	16384	***Points Count***	131072
Pulse Sequence	zgpg30	***Solvent***	CHLOROFORM-D			***Sweep Width (Hz)***	24154.59
Temperature (degree C)	27.000						

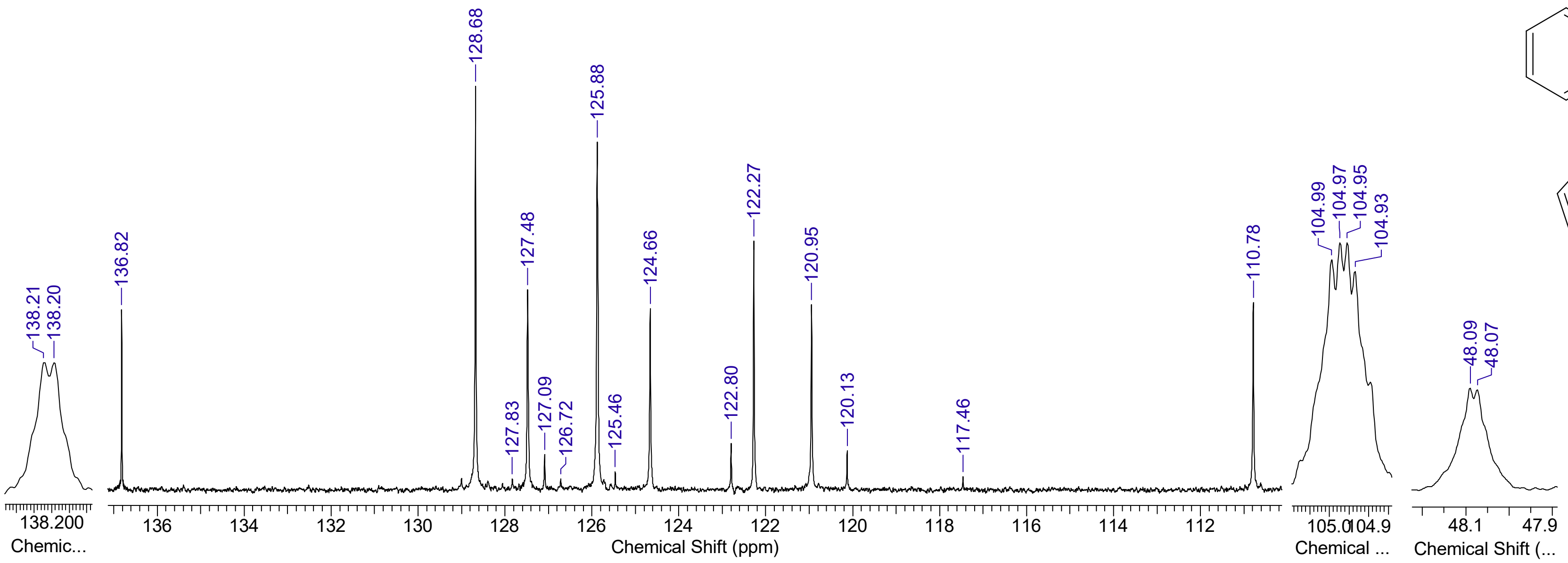

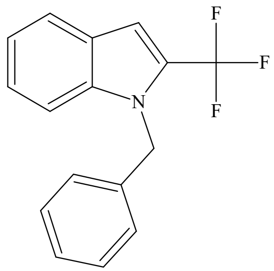

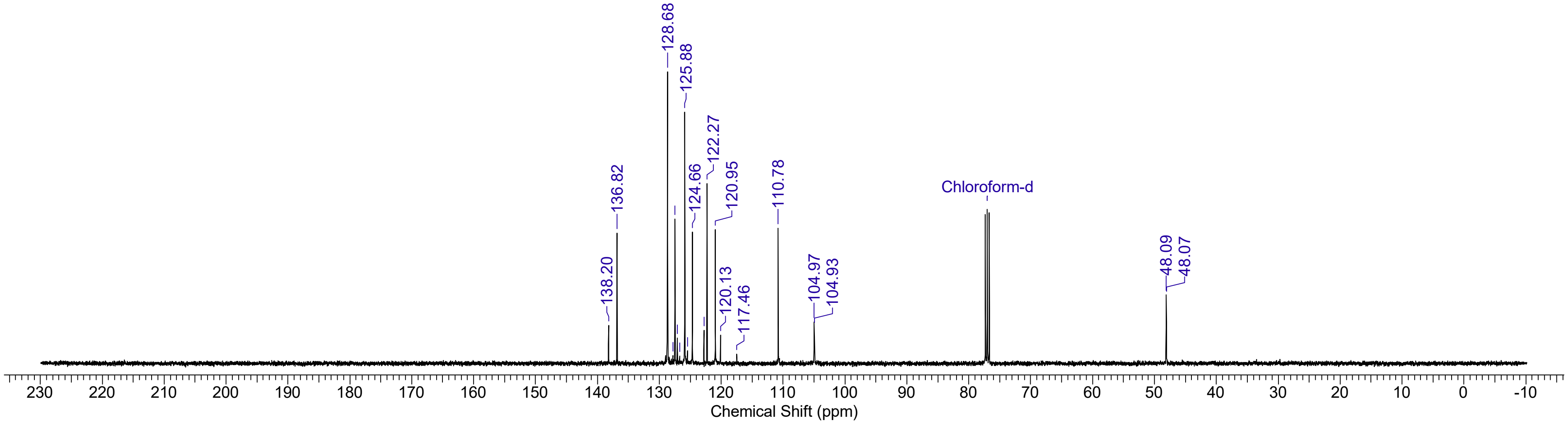

$^{13}C\{^1H\}$ NMR spectrum of **6a** (100.6 MHz, $CDCl_3$)

8 Oct 2022

Acquisition Time (sec)	4.0894	***Comment***	Imported from UXNMR.			***Date***	26 Sep 2022 15:19:32
File Name	C:\DOCS\OUTPUT_301\2022\09.сентябрь\SZA-BM-2615-4.H_001001r					***Frequency (MHz)***	400.13
Nucleus	1H	***Number of Transients***	4	***Original Points Count***	32768	***Points Count***	131072
Pulse Sequence	zg30	***Solvent***	CHLOROFORM-D			***Sweep Width (Hz)***	8012.82
Temperature (degree C)	27.000						

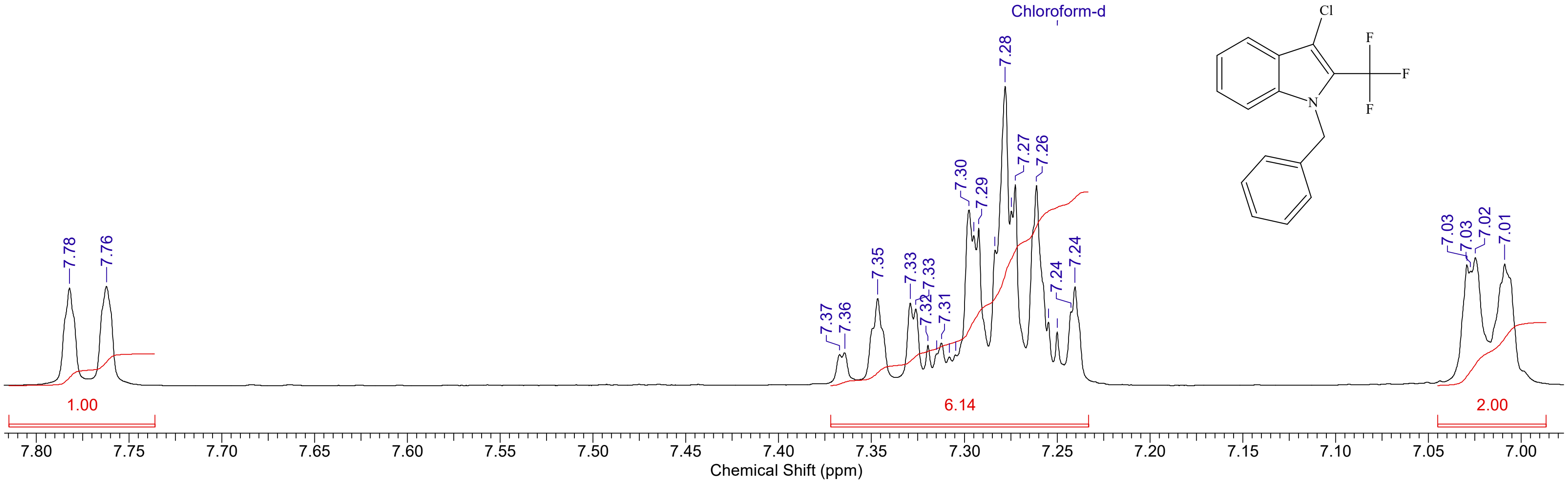

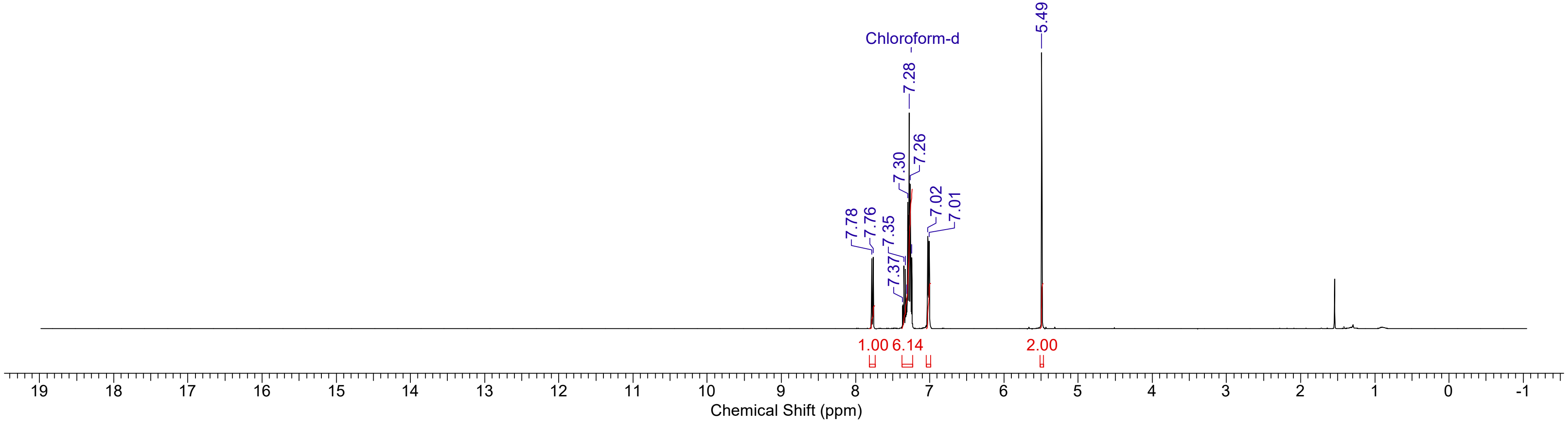

^{1}H NMR spectrum of **6b** (400.1 MHz, $CDCl_3$)

8 Oct 2022

Acquisition Time (sec)	1.7433	***Comment***	Imported from UXNMR.			***Date***	26 Sep 2022 15:23:46
File Name	C:\DOCS\OUTPUT_301\2022\09.сентябрь\SZA-BM-2615-4.F_005001r					***Frequency (MHz)***	376.50
Nucleus	19F	***Number of Transients***	16	***Original Points Count***	131072	***Points Count***	262144
Pulse Sequence	zgflqn	***Solvent***	CHLOROFORM-D			***Sweep Width (Hz)***	75187.97
Temperature (degree C)	27.000						

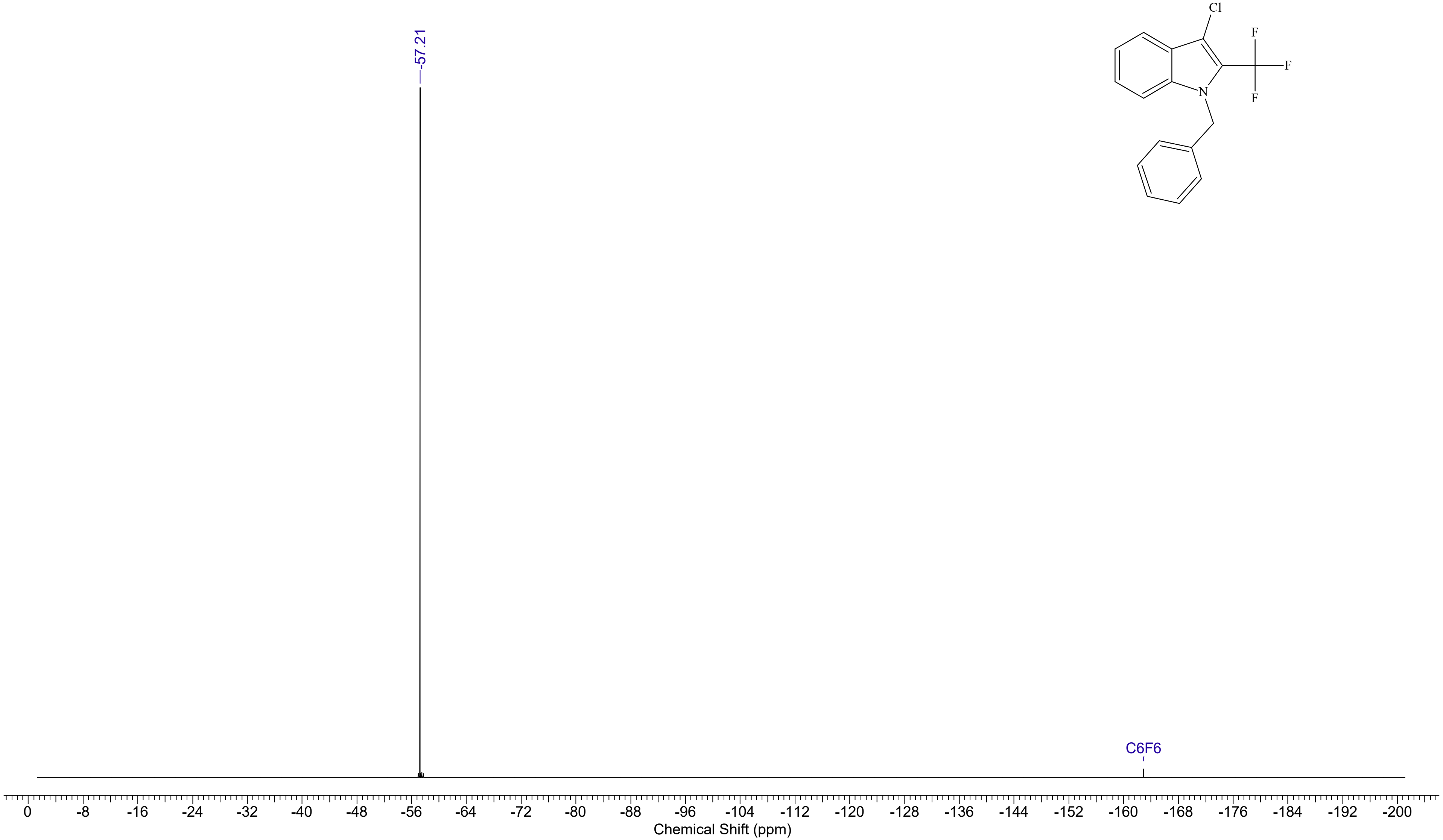

^{19}F NMR spectrum of **6b** (376.5 MHz, $CDCl_3$)

8 Oct 2022

Acquisition Time (sec)	0.6783	***Comment***	Imported from UXNMR.			***Date***	27 Sep 2022 15:51:26
File Name	C:\DOCS\OUTPUT_301\2022\09.сентябрь\SZA-BM-2615-4.C_002001r					***Frequency (MHz)***	100.61
Nucleus	13C	***Number of Transients***	161	***Original Points Count***	16384	***Points Count***	131072
Pulse Sequence	zgpg30	***Solvent***	CHLOROFORM-D			***Sweep Width (Hz)***	24154.59
Temperature (degree C)	27.000						

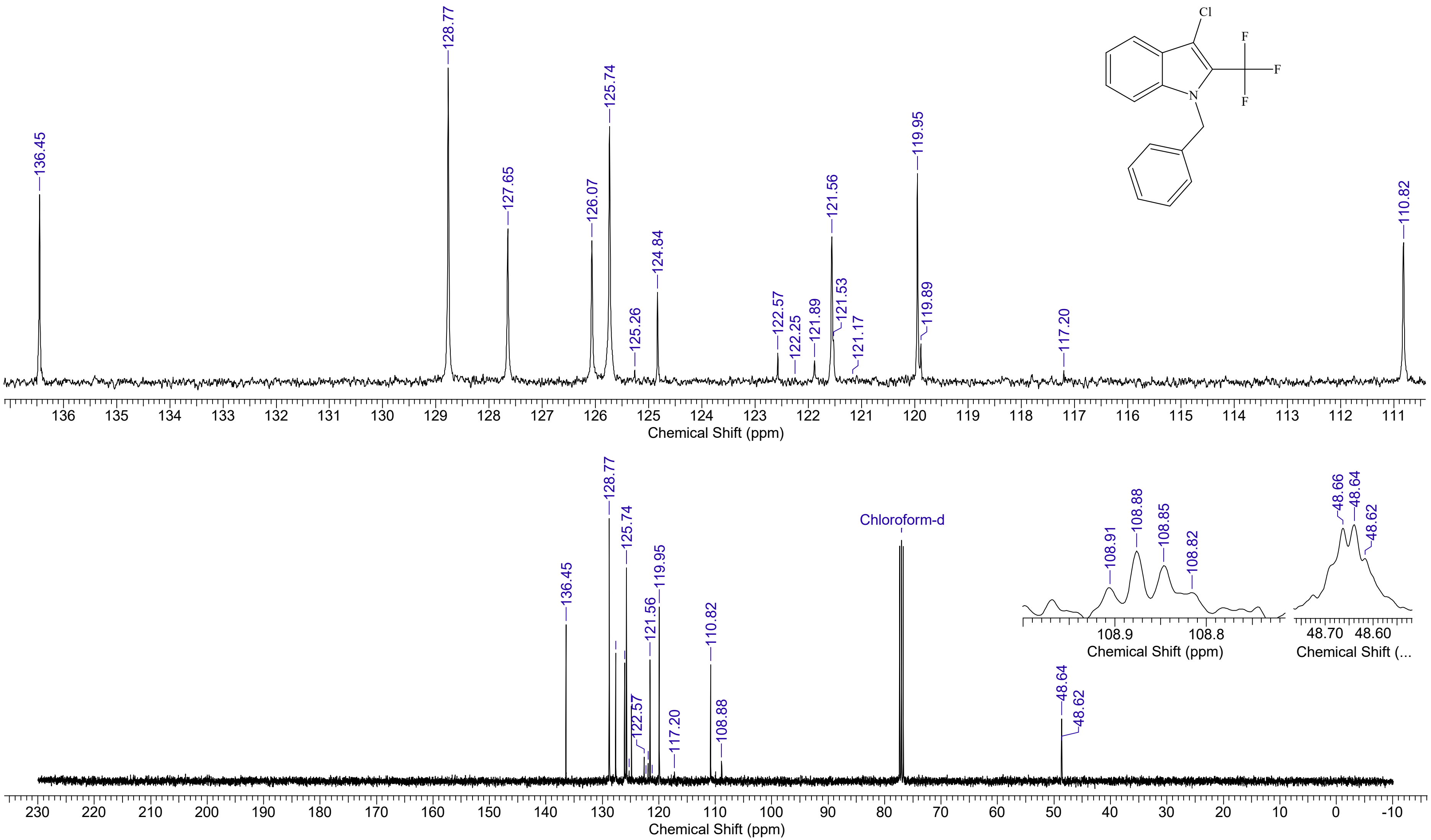

$^{13}C\{^1H\}$ NMR spectrum of **6b** (100.6 MHz, $CDCl_3$)

8 Oct 2022

Acquisition Time (sec) 4.0894	***Comment***	Imported from UXNMR.		***Date***	14 Sep 2022 17:21:40
File Name	C:\DOCS\OUTPUT_301\2022\09.сентябрь\SZA-BM-2582-2.H_001001r			***Frequency (MHz)***	400.13
Nucleus 1H	***Number of Transients*** 4	***Original Points Count***	32768	***Points Count***	131072
Pulse Sequence zg30	***Solvent***	CHLOROFORM-D		***Sweep Width (Hz)***	8012.82
Temperature (degree C) 27.000					

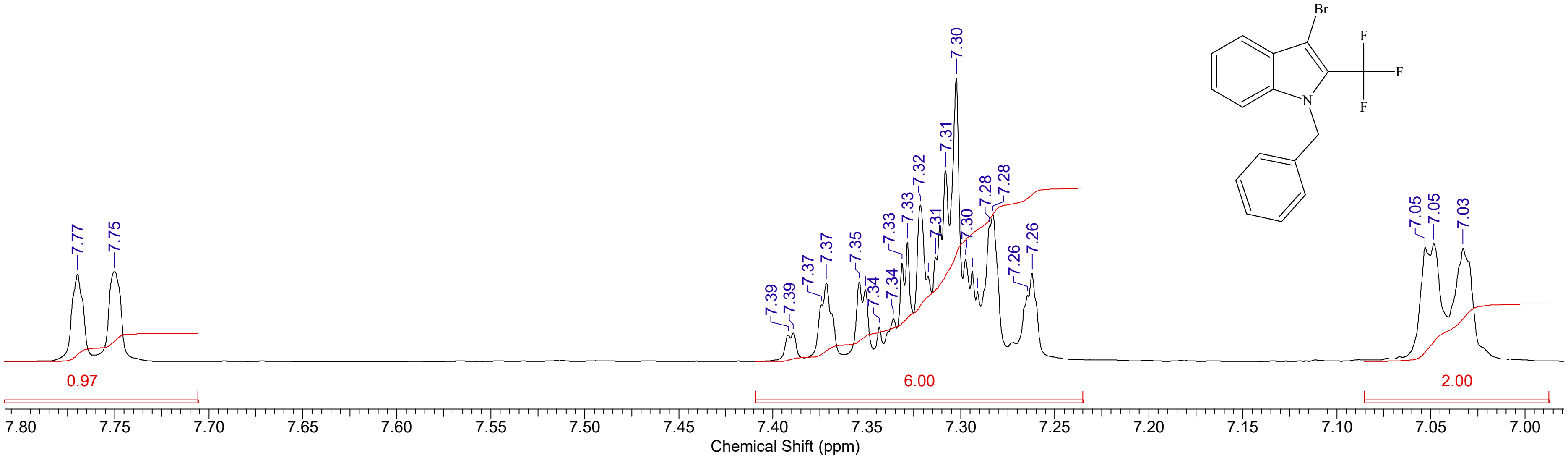

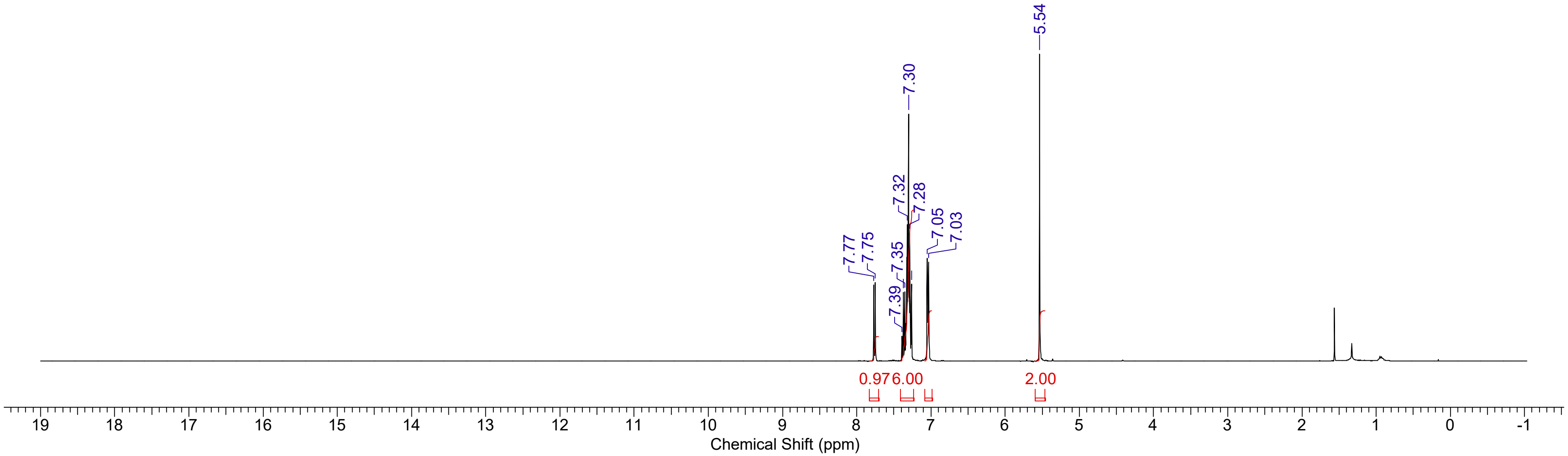

^{1}H NMR spectrum of **6c** (400.1 MHz, $CDCl_3$)

8 Oct 2022

Acquisition Time (sec)	1.7433	***Comment***	Imported from UXNMR.			***Date***	14 Sep 2022 17:30:46
File Name	C:\DOCS\OUTPUT_301\2022\09.сентябрь\SZA-BM-2582-2.F_005001r					***Frequency (MHz)***	376.50
Nucleus	19F	***Number of Transients***	16	***Original Points Count***	131072	***Points Count***	262144
Pulse Sequence	zgflqn	***Solvent***	CHLOROFORM-D			***Sweep Width (Hz)***	75187.97
Temperature (degree C)	27.000						

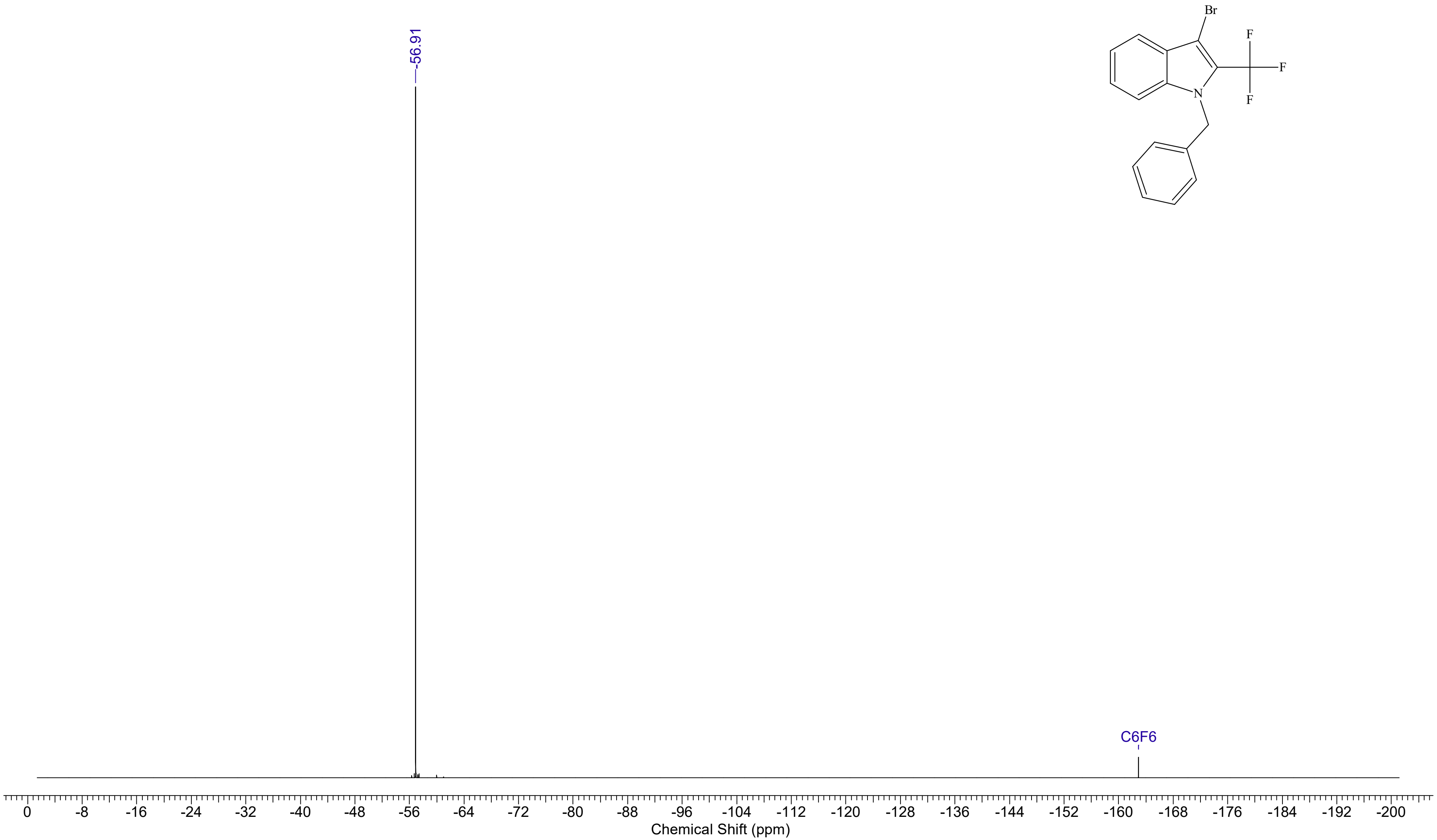

^{19}F NMR spectrum of **6c** (376.5 MHz, $CDCl_3$)

8 Oct 2022

Acquisition Time (sec)	0.6783	***Comment***	Imported from UXNMR.			***Date***	15 Sep 2022 11:56:58
File Name	C:\DOCS\OUTPUT_301\2022\09.сентябрь\SZA-BM-2582-2.C_002001r					***Frequency (MHz)***	100.61
Nucleus	13C	***Number of Transients***	193	***Original Points Count***	16384	***Points Count***	131072
Pulse Sequence	zgpg30	***Solvent***	CHLOROFORM-D			***Sweep Width (Hz)***	24154.59
Temperature (degree C)	27.000						

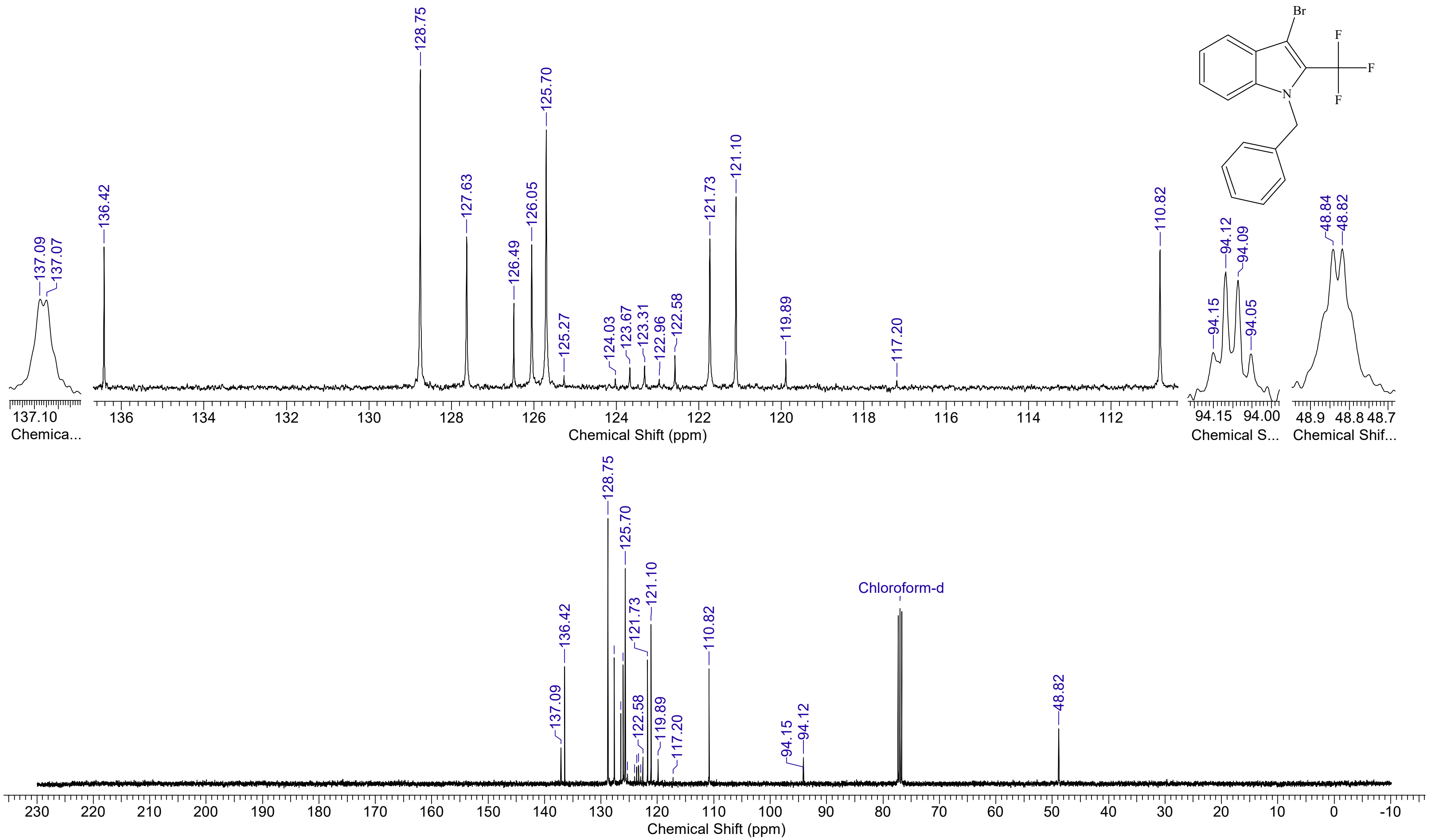

$^{13}C\{^1H\}$ NMR spectrum of **6c** (100.6 MHz, $CDCl_3$)

8 Oct 2022

Acquisition Time (sec)	4.0894	***Comment***	Imported from UXNMR.	***Date***	21 Sep 2022 17:12:20
File Name	C:\DOCS\OUTPUT_301\2022\09.сентябрь\SZA-BM-2606-5.H_001001r			***Frequency (MHz)***	400.13
Nucleus	1H	***Number of Transients***	4 ***Original Points Count*** 32768	***Points Count***	131072
Pulse Sequence	zg30	***Solvent***	CHLOROFORM-D	***Sweep Width (Hz)***	8012.82
Temperature (degree C)	27.000				

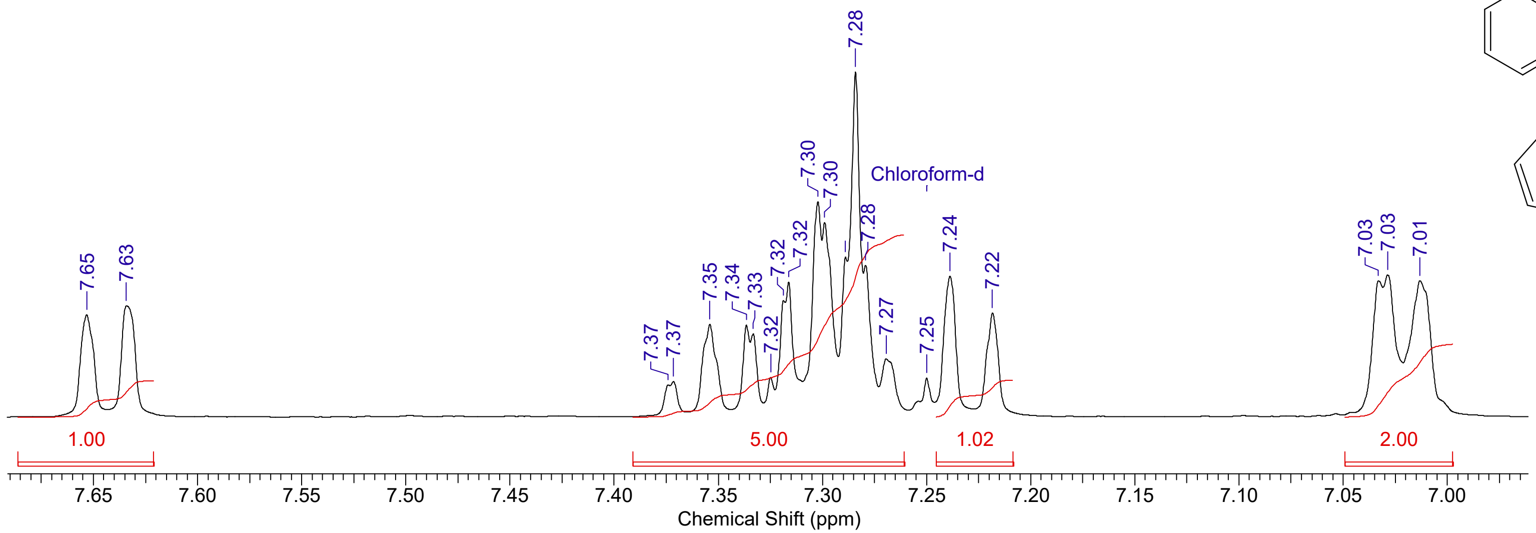

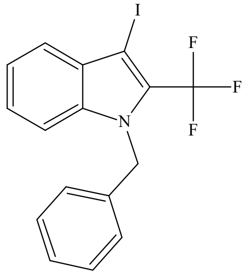

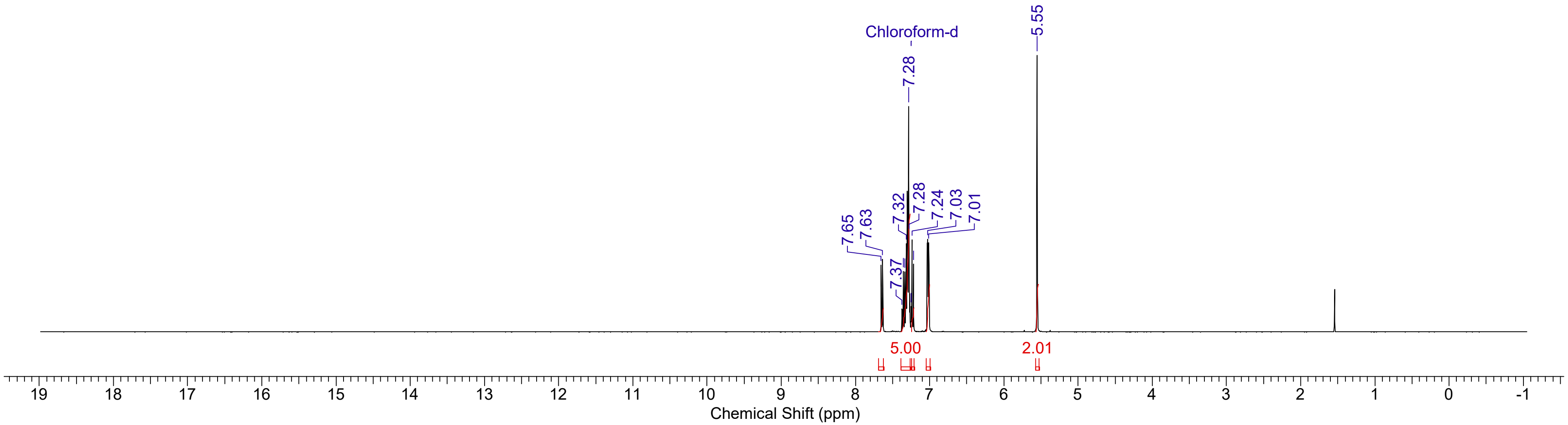

^{1}H NMR spectrum of **6d** (400.1 MHz, $CDCl_3$)

8 Oct 2022

Acquisition Time (sec)	1.7433	***Comment***	Imported from UXNMR.			***Date***	20 Sep 2022 15:33:44
File Name	C:\DOCS\OUTPUT_301\2022\09.сентябрь\SZA-BM-2606-1.F_005001r					***Frequency (MHz)***	376.50
Nucleus	19F	***Number of Transients***	16	***Original Points Count***	131072	***Points Count***	262144
Pulse Sequence	zgflqn	***Solvent***	CHLOROFORM-D			***Sweep Width (Hz)***	75187.97
Temperature (degree C)	27.000						

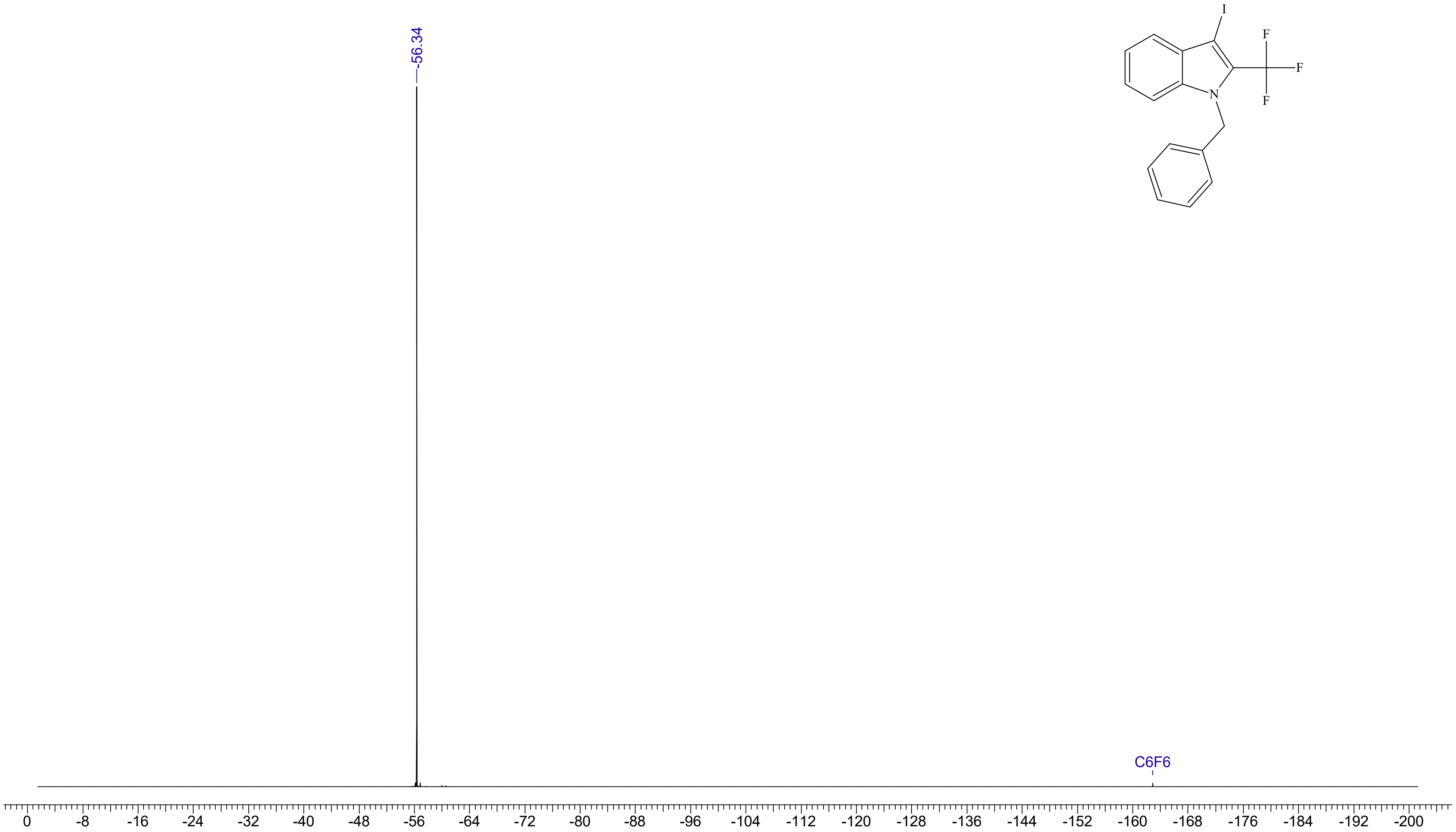

^{19}F NMR spectrum of **6d** (376.5 MHz, $CDCl_3$)

8 Oct 2022

Acquisition Time (sec)	0.6783	***Comment***	Imported from UXNMR.			***Date***	22 Sep 2022 11:33:00
File Name	C:\DOCS\OUTPUT_301\2022\09.сентябрь\SZA-BM-2606-5.C_002001r					***Frequency (MHz)***	100.61
Nucleus	13C	***Number of Transients***	463	***Original Points Count***	16384	***Points Count***	131072
Pulse Sequence	zgpg30	***Solvent***	CHLOROFORM-D			***Sweep Width (Hz)***	24154.59
Temperature (degree C)	27.000						

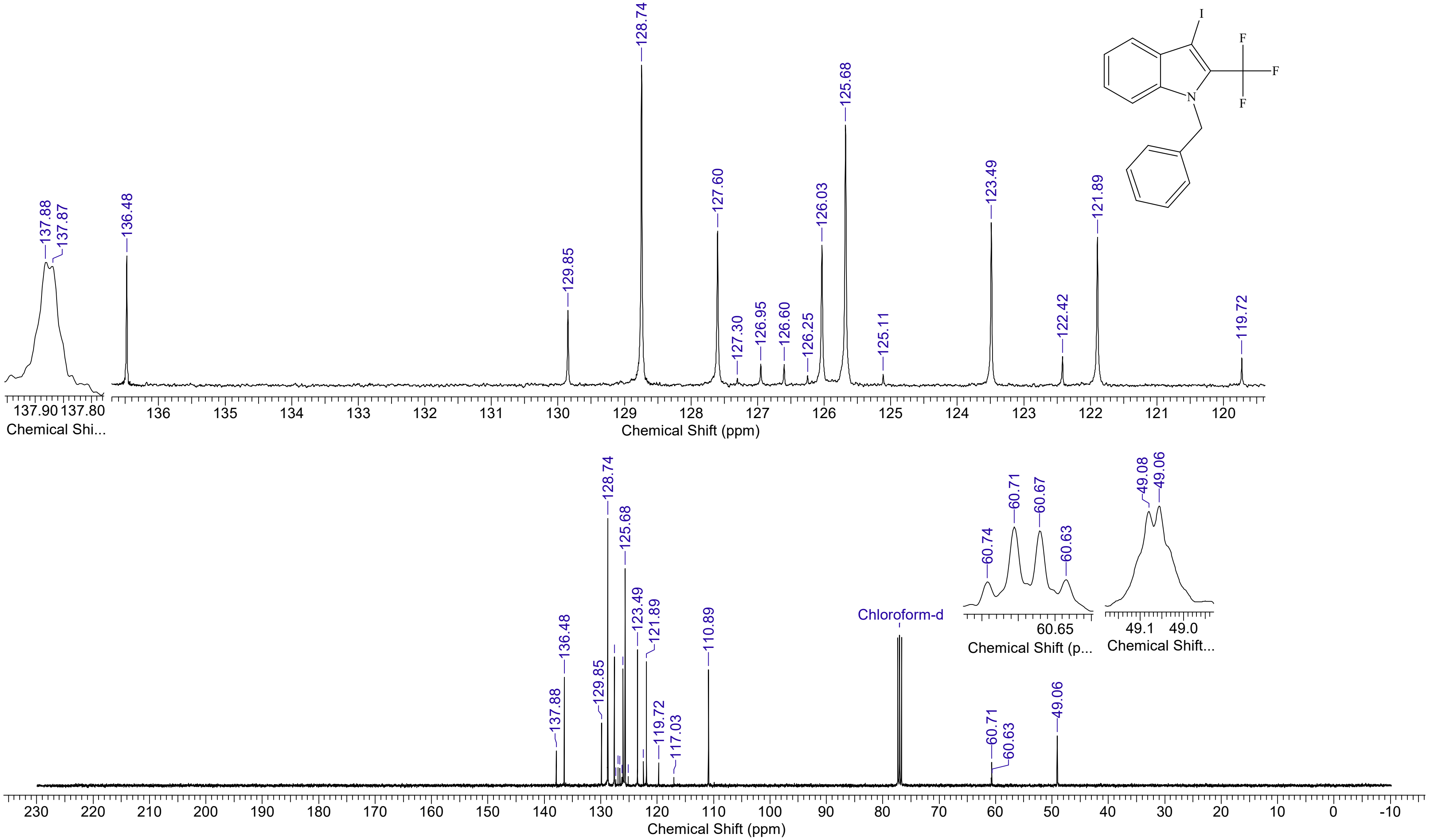

$^{13}C\{^1H\}$ NMR spectrum of **6d** (100.6 MHz, $CDCl_3$)

Acquisition Time (sec)	4.0894	***Comment***	Imported from UXNMR.	***Date***	24 Jun 2021 11:54:22
File Name	C:\DOCS\OUTPUT_301\2021\06.июнь\SZA-BM-2199-9.H_001001r			***Frequency (MHz)***	400.13
Nucleus	1H	***Number of Transients***	8	***Original Points Count*** 32768	***Points Count*** 131072
Pulse Sequence	zg30	***Solvent***	CHLOROFORM-D	***Sweep Width (Hz)***	8012.82
Temperature (degree C)	27.000				

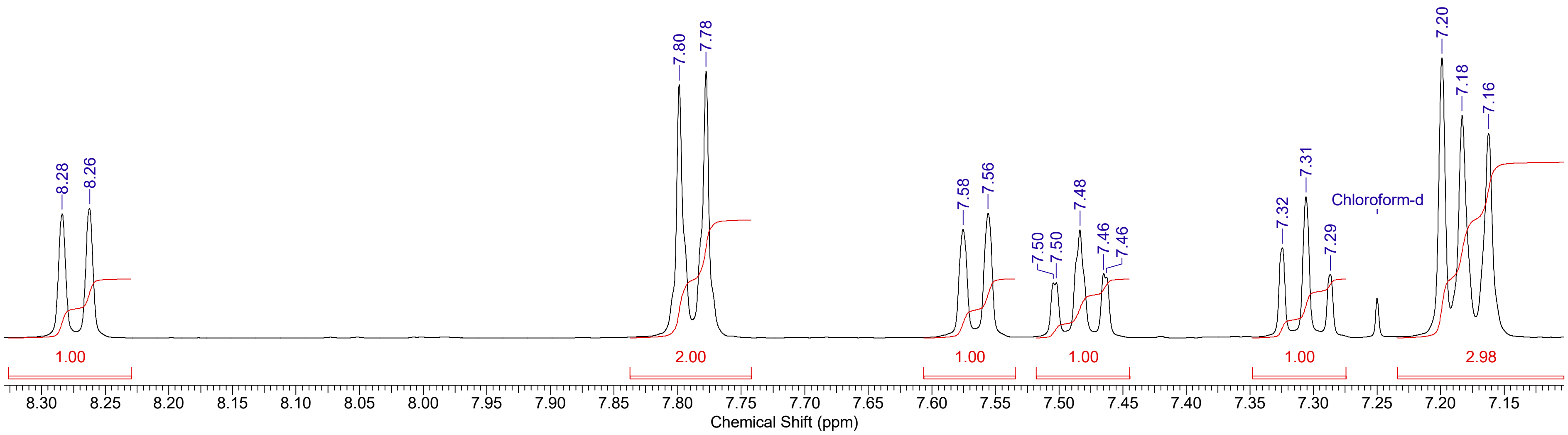

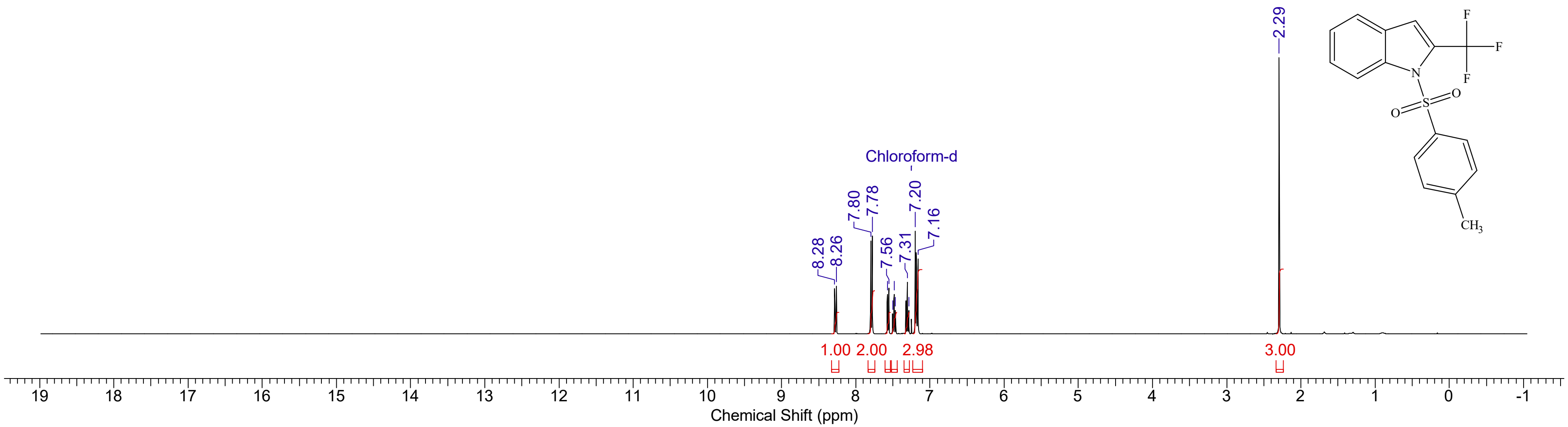

^{1}H NMR spectrum of **7a** (400.1 MHz, $CDCl_3$)

Acquisition Time (sec)	1.7433	***Comment***	Imported from UXNMR.			***Date***	24 Jun 2021 12:14:44
File Name	C:\DOCS\OUTPUT_301\2021\06.июнь\SZA-BM-2199-9.F_005001r					***Frequency (MHz)***	376.50
Nucleus	19F	***Number of Transients***	16	***Original Points Count***	131072	***Points Count***	262144
Pulse Sequence	zgflqn	***Solvent***	CHLOROFORM-D			***Sweep Width (Hz)***	75187.97
Temperature (degree C)	27.000						

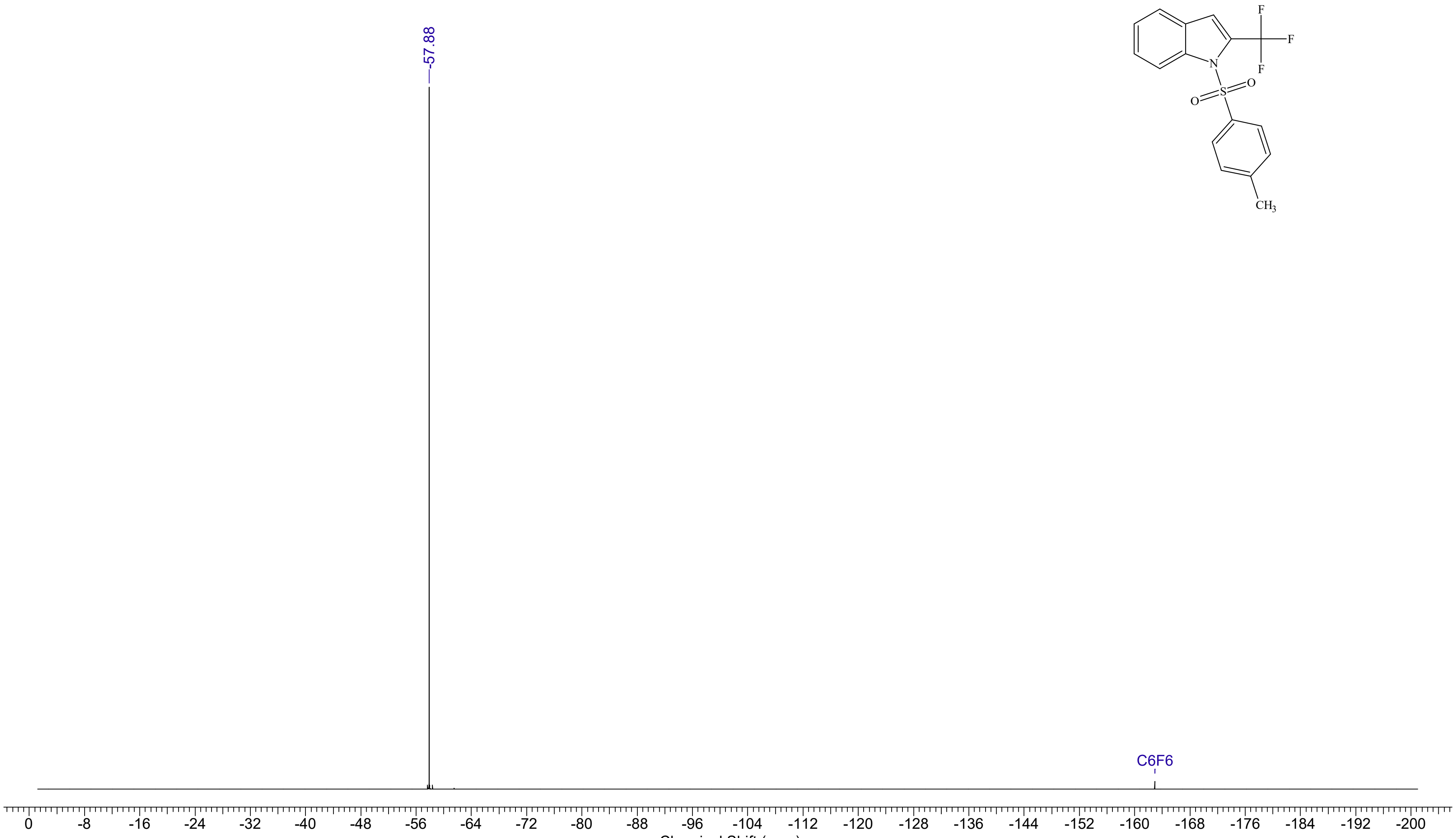

^{19}F NMR spectrum of **7a** (376.5 MHz, $CDCl_3$)

Acquisition Time (sec)	0.6783	***Comment***	Imported from UXNMR.			***Date***	24 Jun 2021 12:10:08
File Name	C:\DOCS\OUTPUT_301\2021\06.июнь\SZA-BM-2199-9.C_002001r					***Frequency (MHz)***	100.61
Nucleus	13C	***Number of Transients***	393	***Original Points Count***	16384	***Points Count***	131072
Pulse Sequence	zgpg30	***Solvent***	ACETONITRILE-D3			***Sweep Width (Hz)***	24154.59
Temperature (degree C)	27.000						

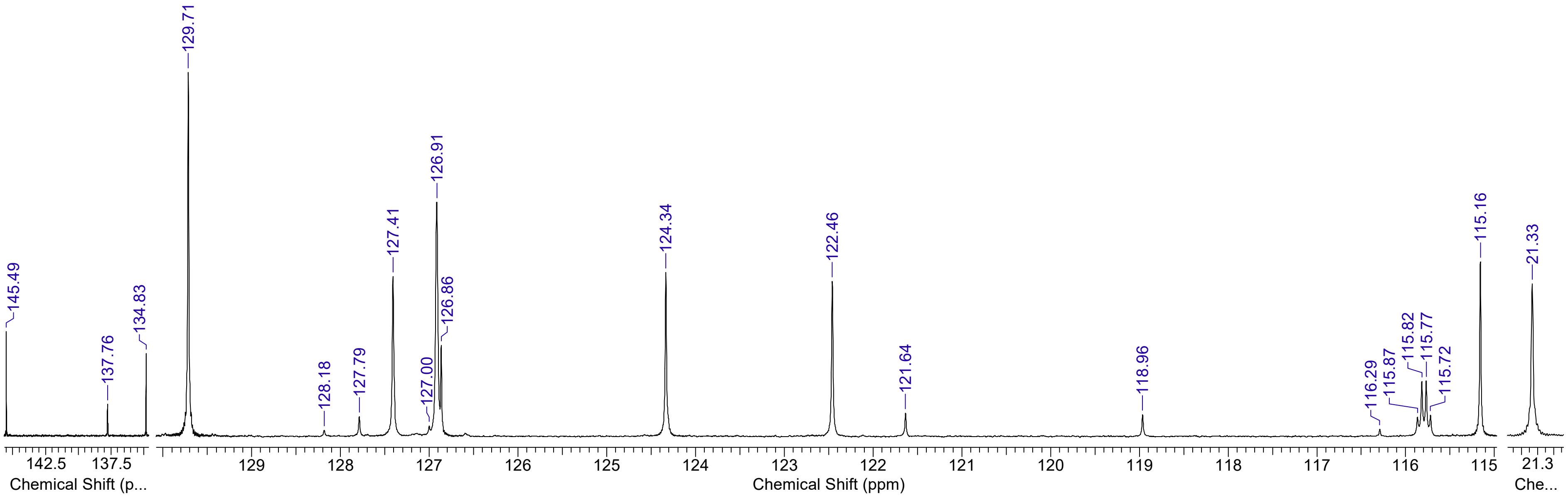

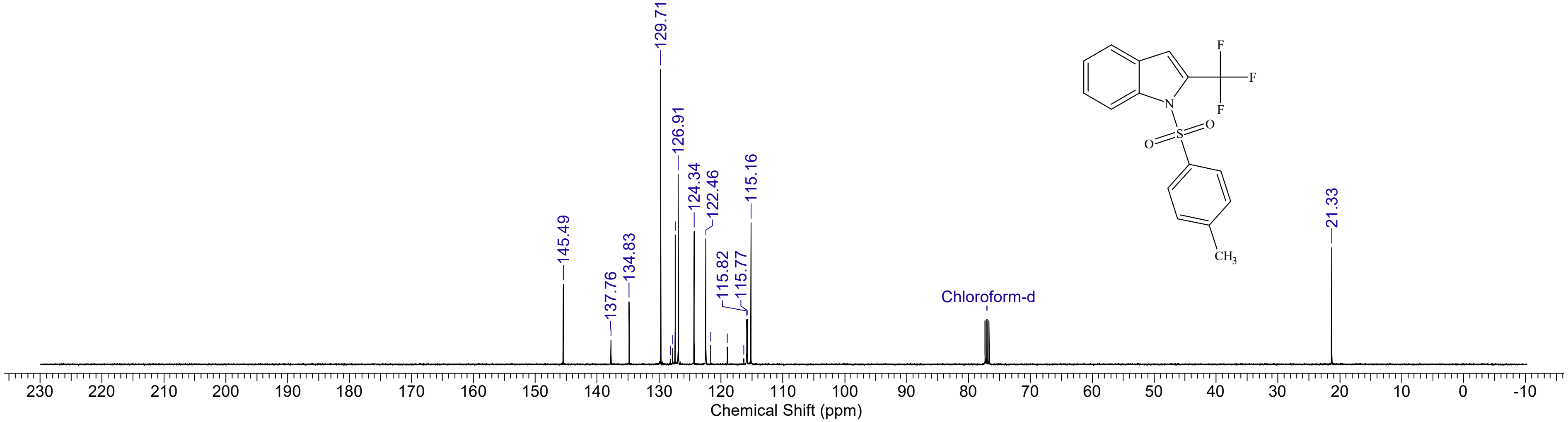

$^{13}C\{^1H\}$ NMR spectrum of **7a** (100.6 MHz, $CDCl_3$)

1 Aug 2021

**Acquisition Time (sec)**	4.0894	**Comment**	Imported from UXNMR.			**Date**	01 Jul 2021 11:23:18
**File Name**	C:\BM_DATA\DOCS\Спектры 30.06-01.07\Спектры 30.06-01.07\SZA-BM-2200-9.H_001001r					**Frequency (MHz)**	400.13
**Nucleus**	1H	**Number of Transients**	4	**Original Points Count**	32768	**Points Count**	131072
**Pulse Sequence**	zg30	**Solvent**	CHLOROFORM-D	**Sweep Width (Hz)**	8012.82	**Temperature (degree C)**	27.000

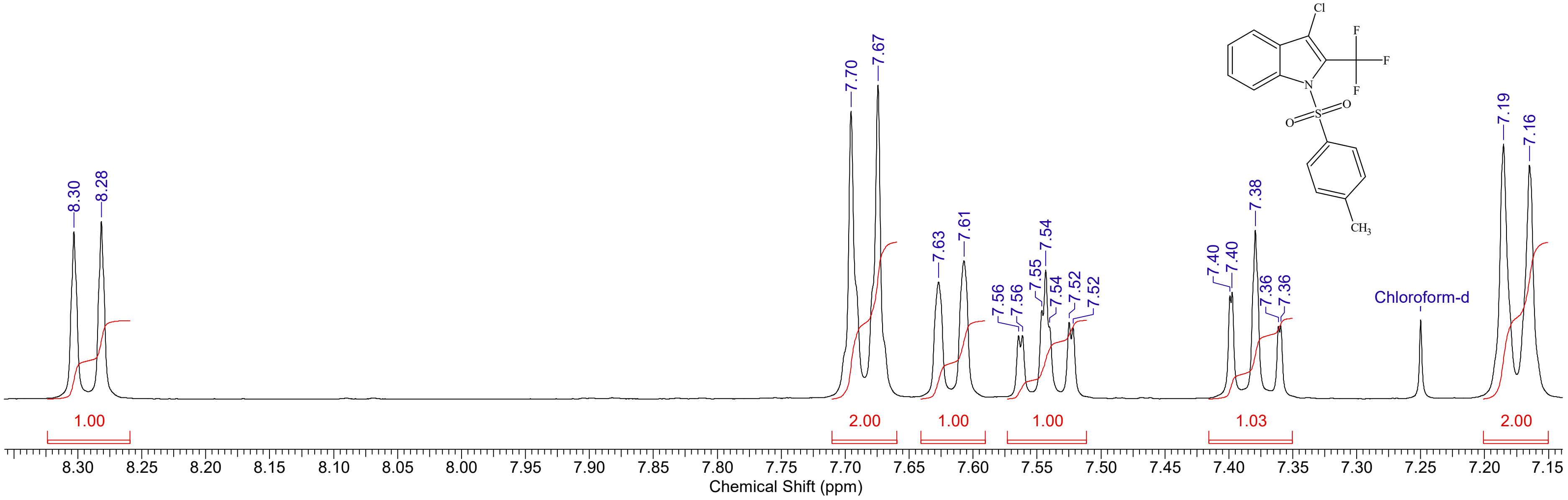

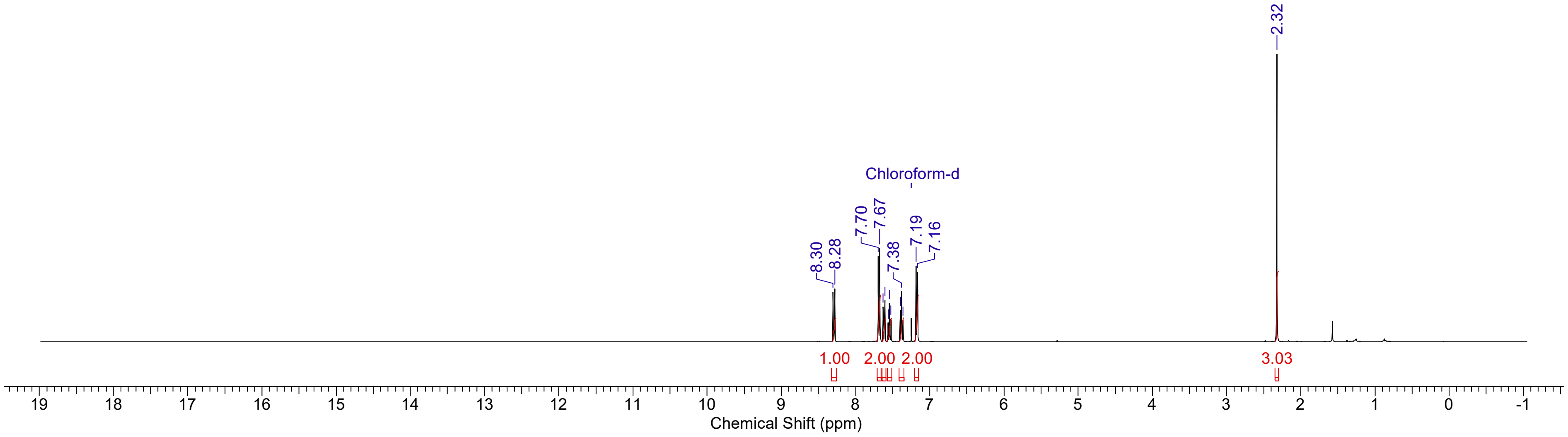

^{1}H NMR spectrum of **7b** (400.1 MHz, $CDCl_3$)

1 Aug 2021

Acquisition Time (sec)	1.7433	***Comment***	Imported from UXNMR.			***Date***	01 Jul 2021 12:47:28
File Name	C:\BM_DATA\DOCS\Спектры 30.06-01.07\Спектры 30.06-01.07\SZA-BM-2200-9.F_005001r					***Frequency (MHz)***	376.50
Nucleus	19F	***Number of Transients***	16	***Original Points Count***	131072	***Points Count***	262144
Pulse Sequence	zgflqn	***Solvent***	CHLOROFORM-D	***Sweep Width (Hz)***	75187.97	***Temperature (degree C)***	27.000

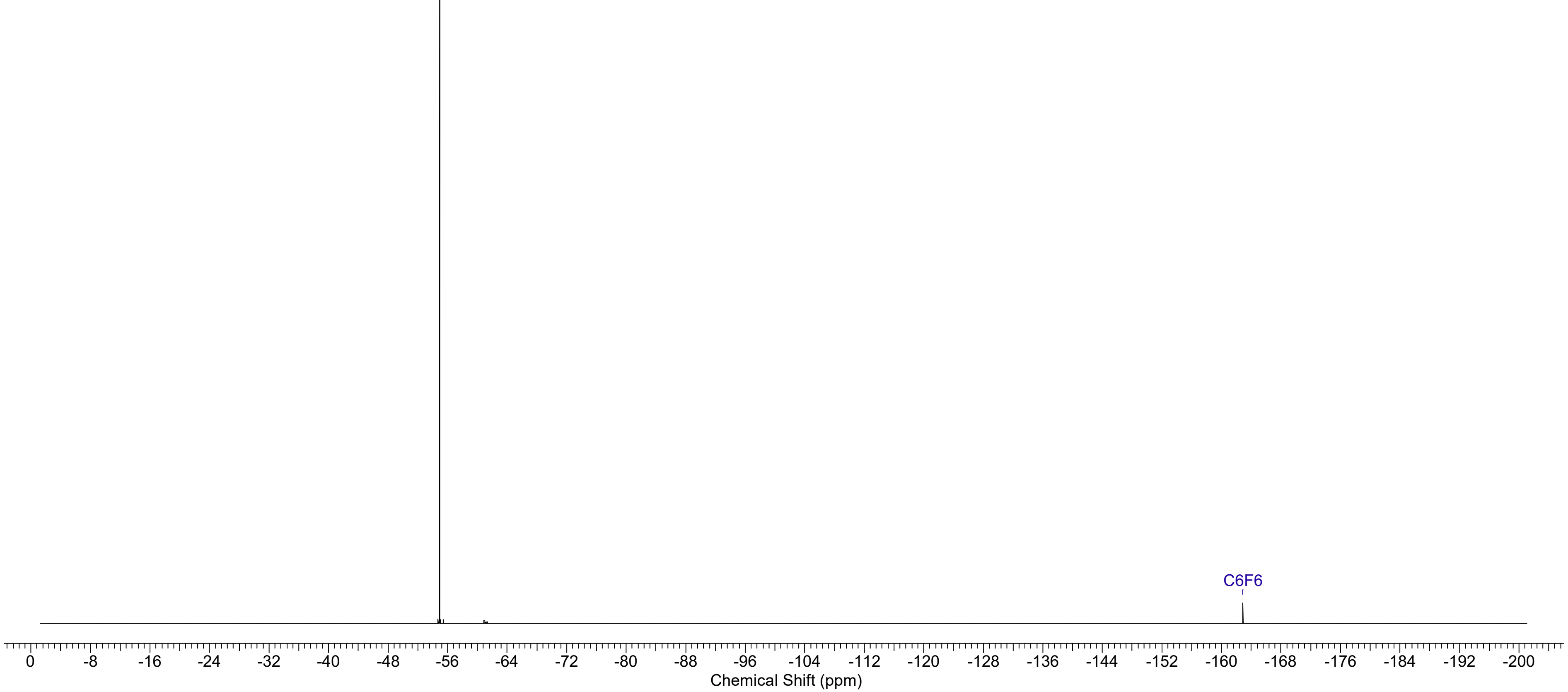

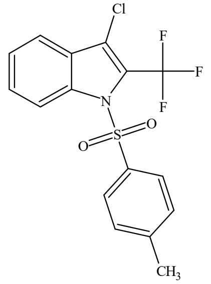

^{19}F NMR spectrum of **7b** (376.5 MHz, $CDCl_3$)

1 Aug 2021

Acquisition Time (sec)	0.6783	Comment	Imported from UXNMR.	Date	02 Jul 2021 15:44:08
File Name	C:\BM_DATA\DOCS\02.07.2021\SZA-BM-2200-9.C_002001r	Frequency (MHz)	100.61	Nucleus	13C
Number of Transients	427	Original Points Count 16384	Points Count 131072	Pulse Sequence	zgpg30
Solvent	ACETONITRILE-D3	Sweep Width (Hz)	24154.59	Temperature (degree C)	27.000

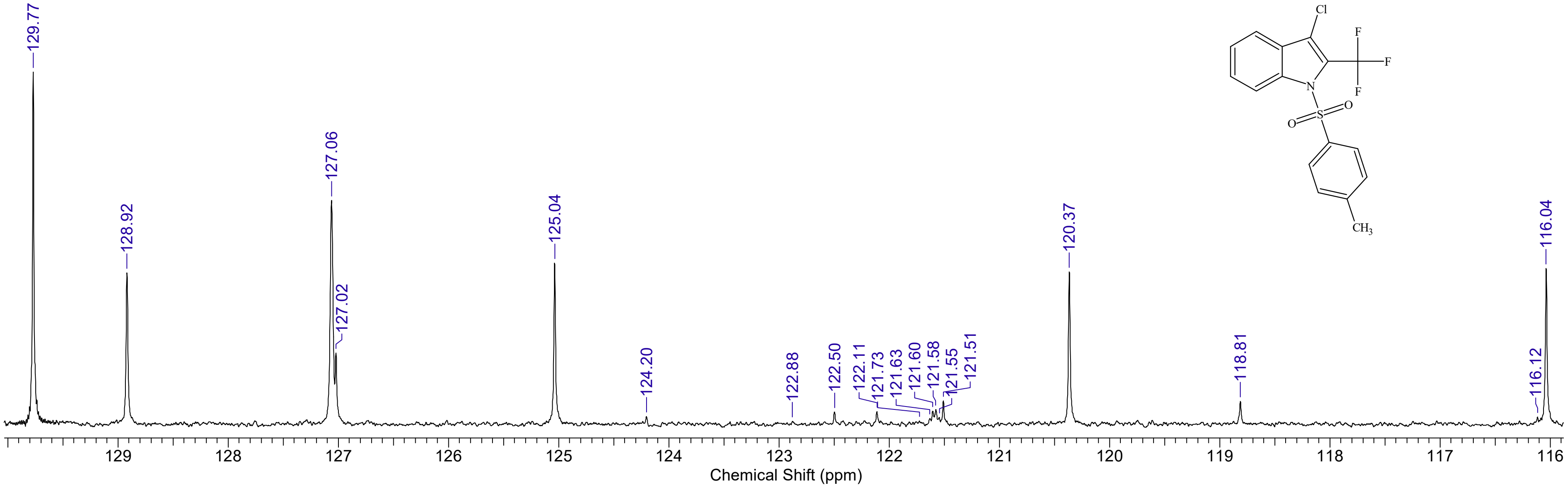

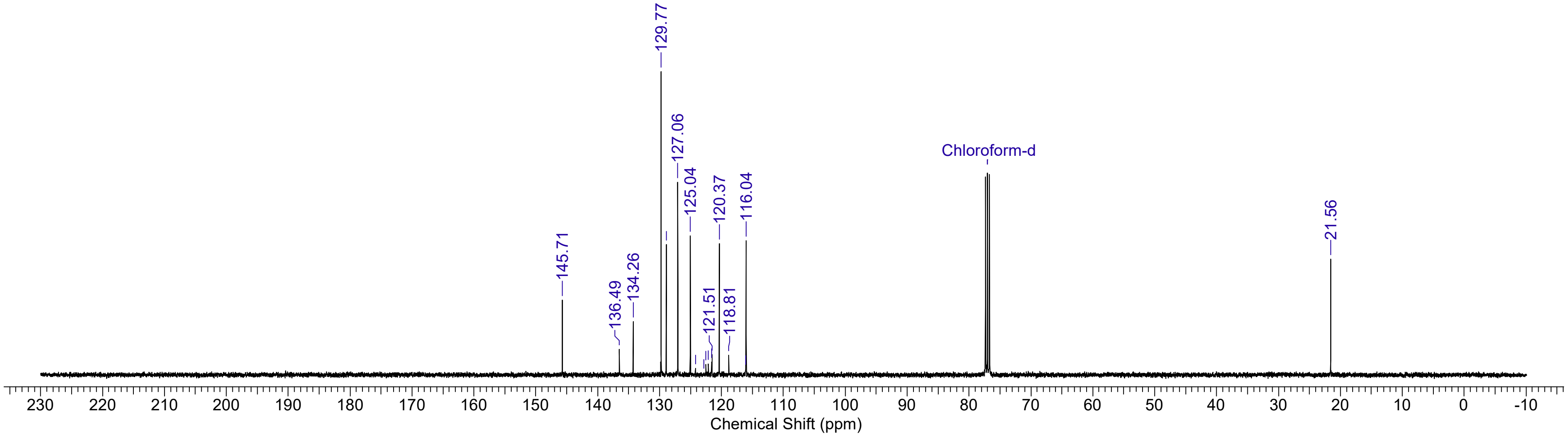

$^{13}C\{^{1}H\}$ NMR spectrum of **7b** (100.6 MHz, $CDCl_3$)

1 Aug 2021

Acquisition Time (sec)	4.0894	***Comment***	Imported from UXNMR.			***Date***	01 Jul 2021 11:25:30
File Name	C:\BM_DATA\DOCS\Спектры 30.06-01.07\Спектры 30.06-01.07\SZA-BM-2205-9a.H_001001r					***Frequency (MHz)***	400.13
Nucleus	1H	***Number of Transients***	4	***Original Points Count***	32768	***Points Count***	131072
Pulse Sequence	zg30	***Solvent***	CHLOROFORM-D	***Sweep Width (Hz)***	8012.82	***Temperature (degree C)***	27.000

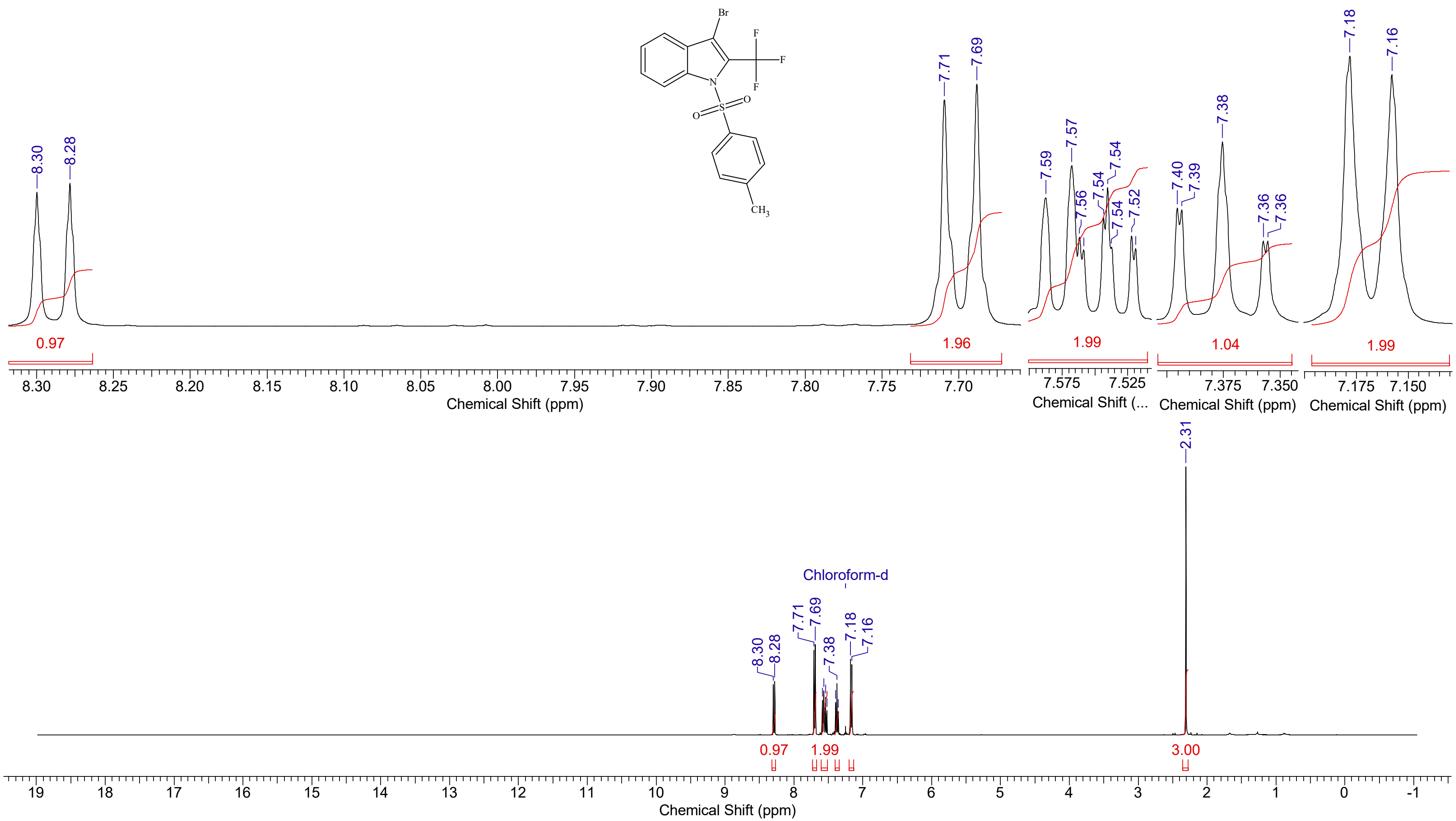

^{1}H NMR spectrum of **7c** (400.1 MHz, $CDCl_3$)

1 Aug 2021

Acquisition Time (sec)	1.7433	Comment	Imported from UXNMR.			Date	30 Jun 2021 17:53:16
File Name	C:\BM_DATA\DOCS\Спектры 30.06-01.07\Спектры 30.06-01.07\SZA-BM-2205-9a.F_005001r					Frequency (MHz)	376.50
Nucleus	19F	Number of Transients	16	Original Points Count	131072	Points Count	262144
Pulse Sequence	zgflqn	Solvent	CHLOROFORM-D	Sweep Width (Hz)	75187.97	Temperature (degree C)	27.000

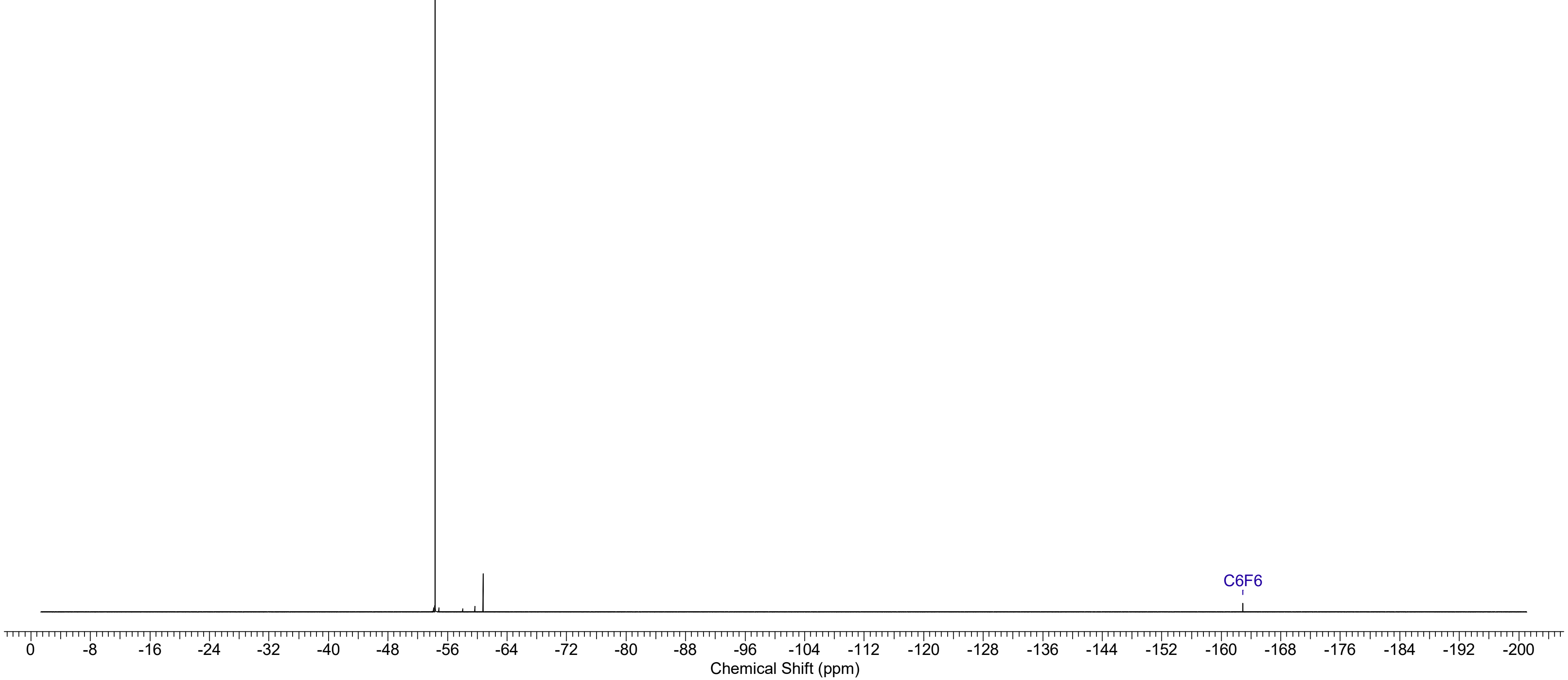

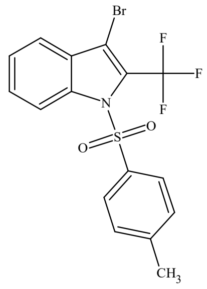

^{19}F NMR spectrum of **7c** (376.5 MHz, $CDCl_3$)

Acquisition Time (sec)	0.6783	Comment	Imported from UXNMR.			Date	01 Jul 2021 12:31:24
File Name	C:\BM_DATA\DOCS\Спектры 30.06-01.07\Спектры 30.06-01.07\SZA-BM-2205.C_002001r					Frequency (MHz)	100.61
Nucleus	13C	Number of Transients	137	Original Points Count	16384	Points Count	131072
Pulse Sequence	zgpg30	Solvent	ACETONITRILE-D3			Sweep Width (Hz)	24154.59
Temperature (degree C)	27.000						

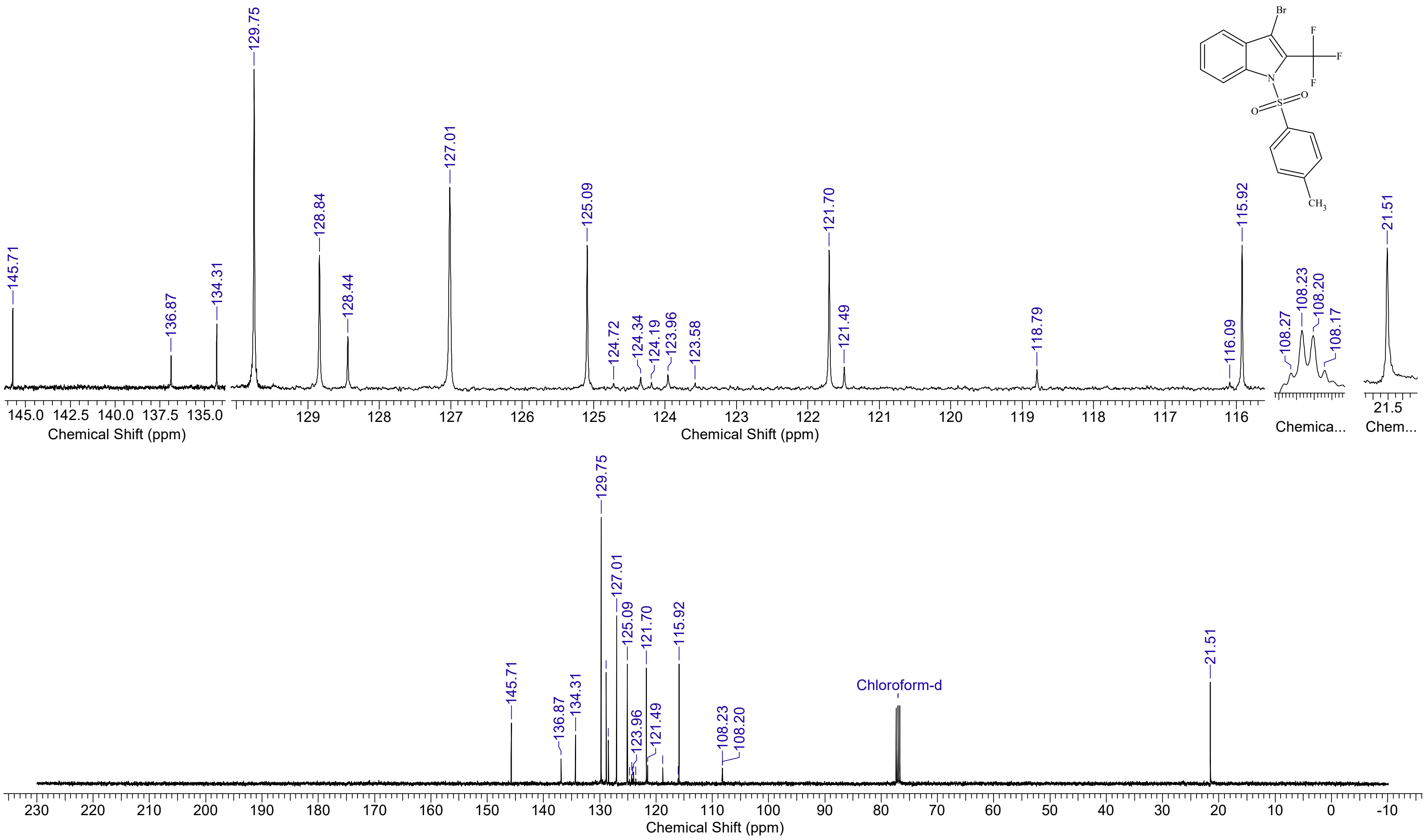

$^{13}C\{^{1}H\}$ NMR spectrum of **7c** (100.6 MHz, $CDCl_3$)

30 Aug 2021

Acquisition Time (sec)	4.0894	Comment	Imported from UXNMR.			Date	30 Aug 2021 15:17:56
File Name	C:\BM_DATA\DOCS\2021.08. август\2021.08. август\SZA-BM-2245-2.H_001001r					Frequency (MHz)	400.13
Nucleus	1H	Number of Transients	4	Original Points Count	32768	Points Count	131072
Pulse Sequence	zg30	Solvent	CHLOROFORM-D			Sweep Width (Hz)	8012.82
Temperature (degree C)	27.000						

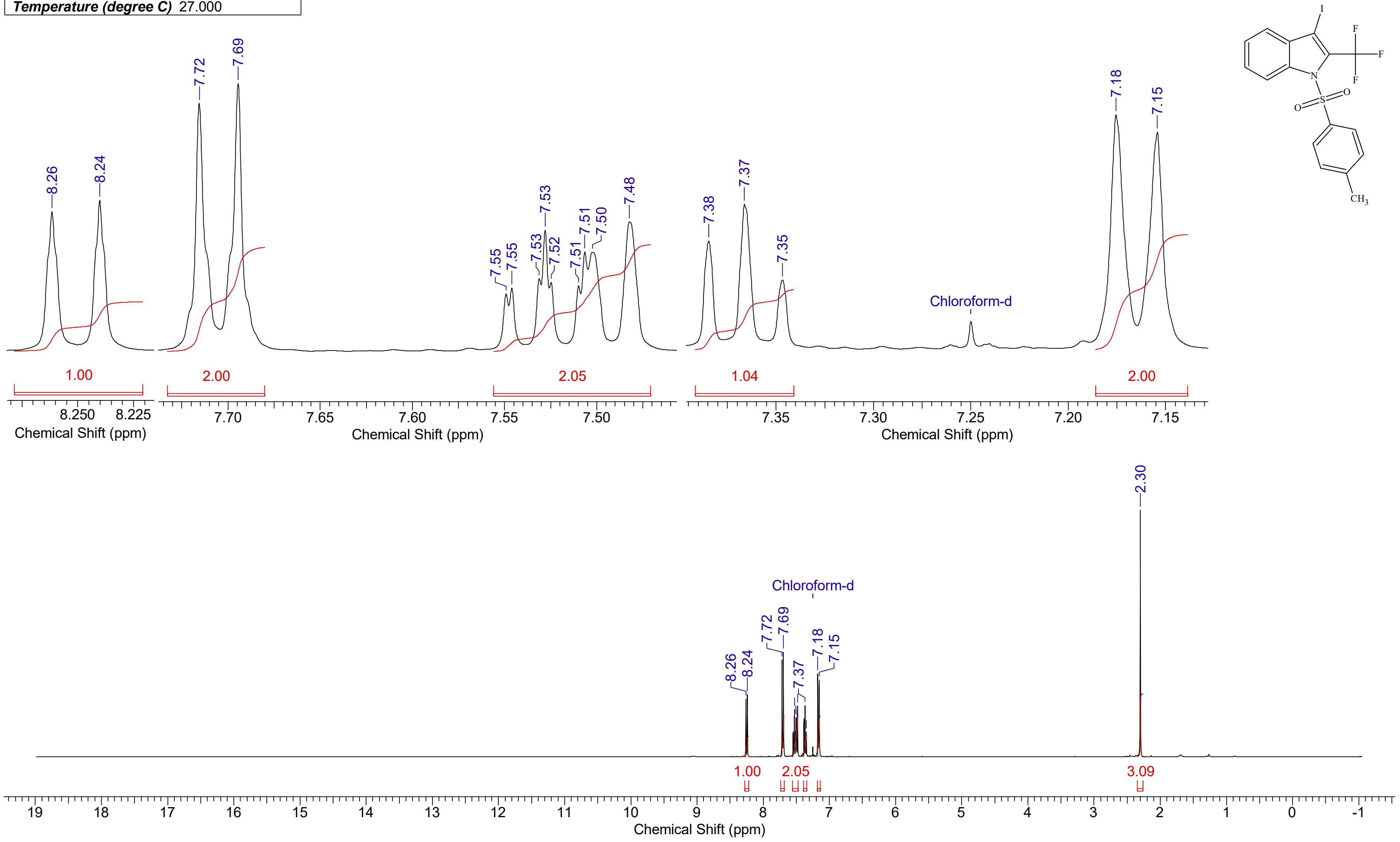

^1H NMR spectrum of **7d** (400.1 MHz, $CDCl_3$)

30 Aug 2021

Acquisition Time (sec) 1.7433	***Comment***	Imported from UXNMR.		***Date***	30 Aug 2021 15:37:00
File Name	C:\BM_DATA\DOCS\2021.08. август\2021.08. август\SZA-BM-2245-2.F_005001r			***Frequency (MHz)***	376.50
Nucleus 19F	***Number of Transients***	16	***Original Points Count*** 131072	***Points Count***	262144
Pulse Sequence zgflqn	***Solvent***	CHLOROFORM-D		***Sweep Width (Hz)***	75187.97
Temperature (degree C) 27.000					

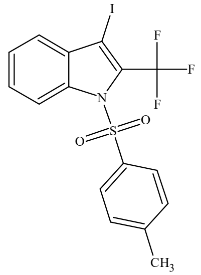

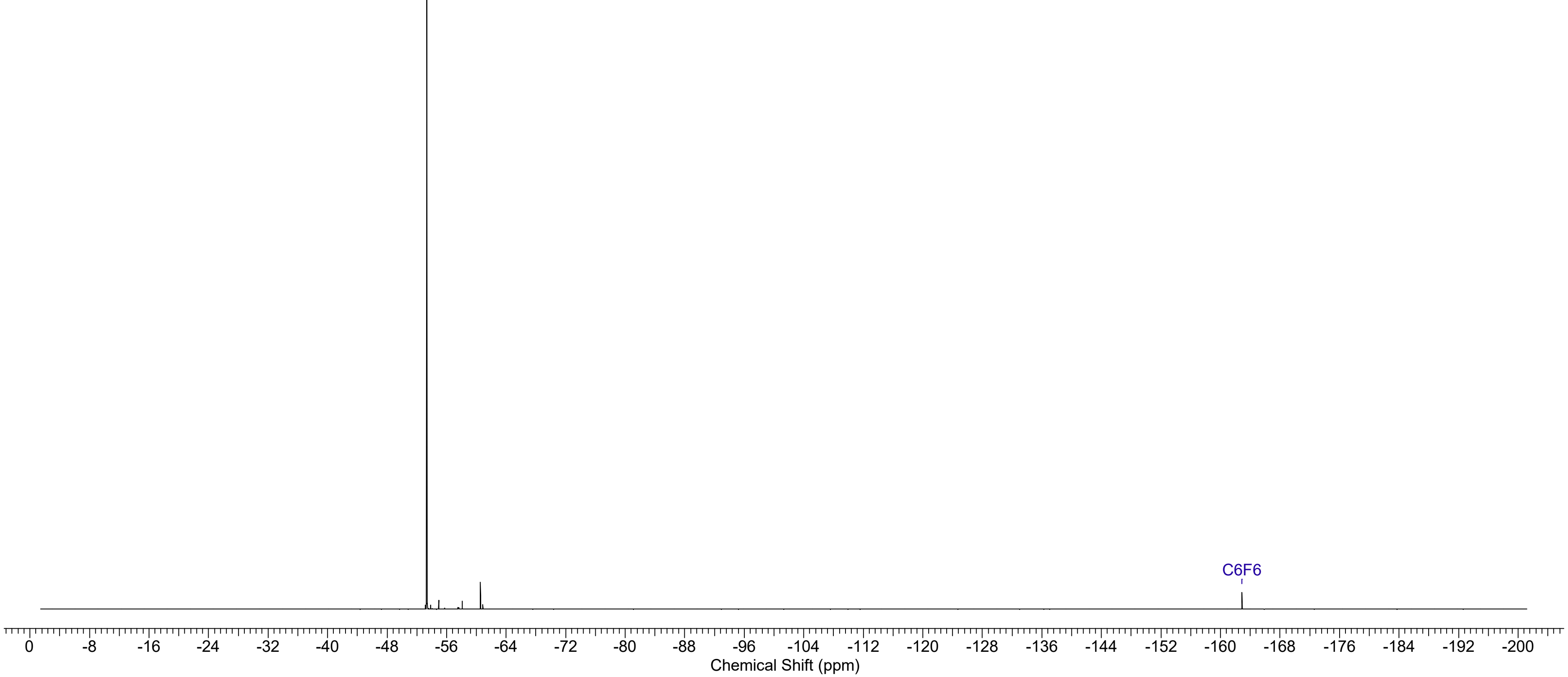

^{19}F NMR spectrum of **7d** (376.5 MHz, $CDCl_3$)

1 Sep 2021

Acquisition Time (sec)	0.6783	Comment	Imported from UXNMR.			Date	31 Aug 2021 14:42:04
File Name	C:\BM_DATA\DOCS\SZA-BM-2245-2.C\SZA-BM-2245-2.C_002001r					Frequency (MHz)	100.61
Nucleus	13C	Number of Transients	321	Original Points Count	16384	Points Count	131072
Pulse Sequence	zgpg30	Solvent	CHLOROFORM-D			Sweep Width (Hz)	24154.59
Temperature (degree C)	27.000						

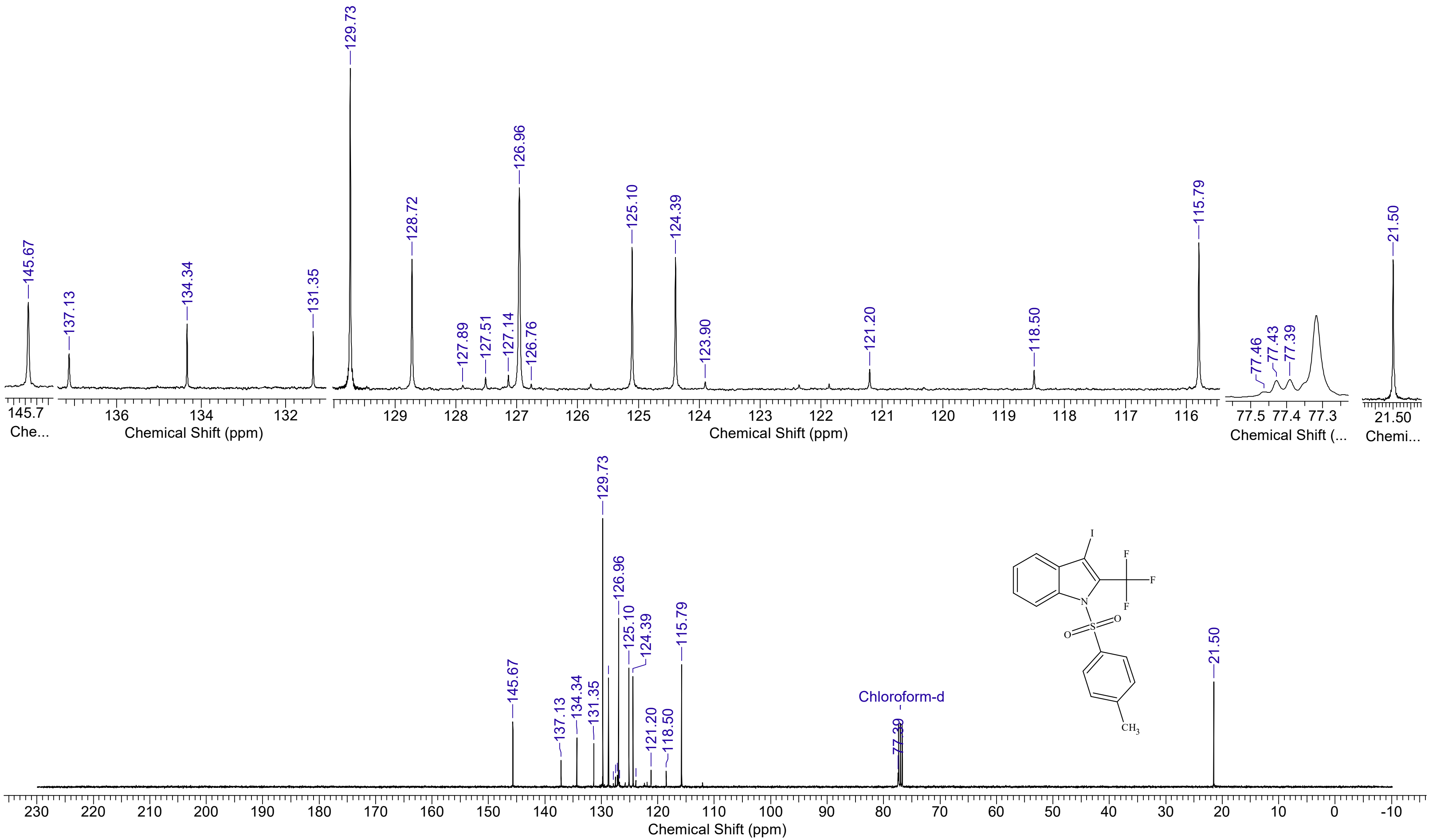

$^{13}C\{^1H\}$ NMR spectrum of **7d** (100.6 MHz, $CDCl_3$)

26 Aug 2021

Acquisition Time (sec)	4.0894	Comment	Imported from UXNMR.			Date	13 Aug 2021 21:31:22
File Name	C:\DOCS\BM\ЯМР_СОСЕДИ\2021\bm210813\BM-2213-2_001001r					Frequency (MHz)	400.13
Nucleus	1H	Number of Transients	8	Original Points Count	32768	Points Count	131072
Pulse Sequence	zg30	Solvent	CHLOROFORM-D			Sweep Width (Hz)	8012.82
Temperature (degree C)	27.000						

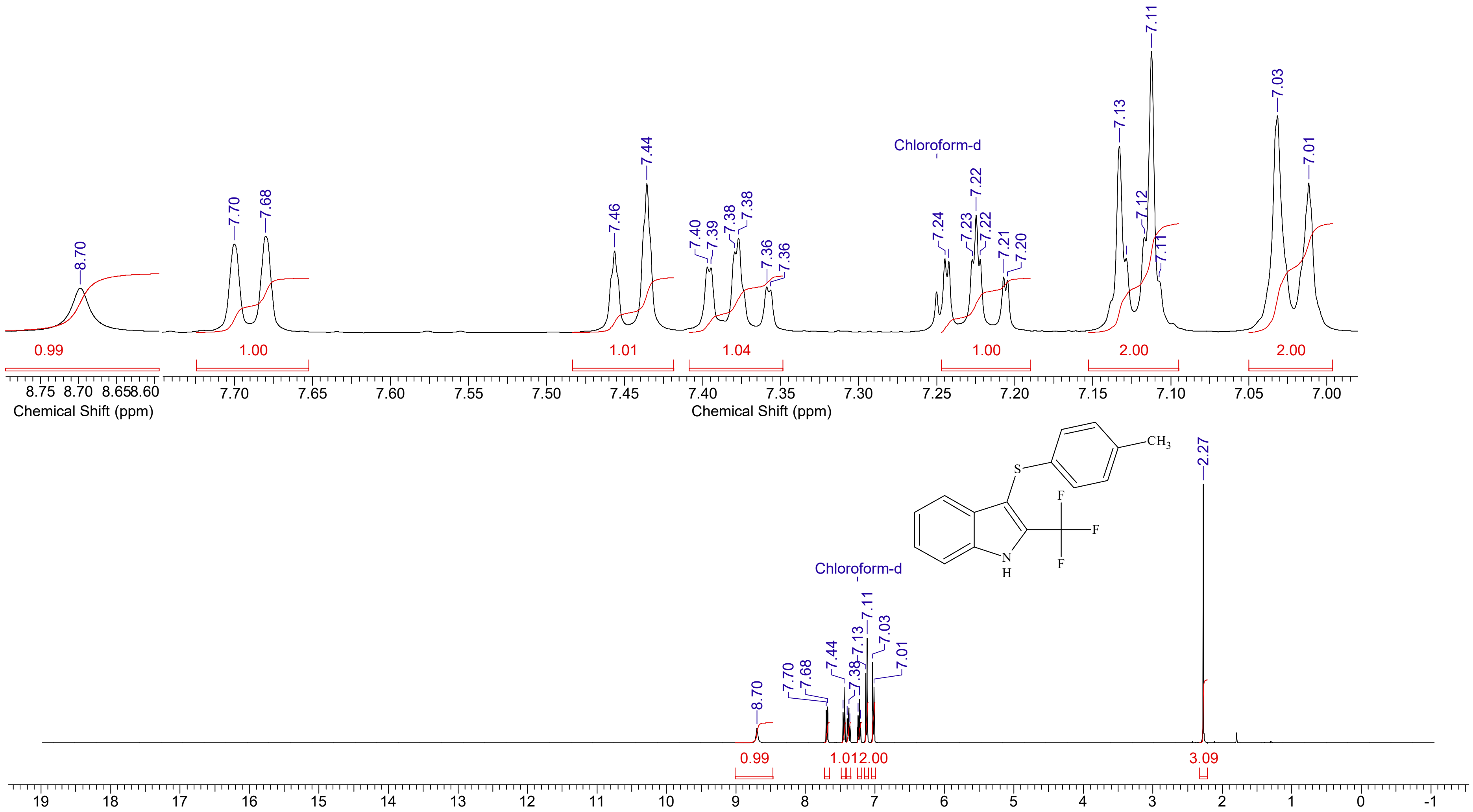

^{1}H NMR spectrum of **9** (400.1 MHz, $CDCl_3$)

26 Aug 2021

Acquisition Time (sec)	1.7433	***Comment***	Imported from UXNMR.	***Date***	14 Aug 2021 22:15:12
File Name	C:\DOCS\ВМ\ЯМР_СОСЕДИ\2021\bm210813-2\BM-2213-2_005001r			***Frequency (MHz)***	376.50
Nucleus	19F	***Number of Transients***	16 ***Original Points Count*** 131072	***Points Count***	262144
Pulse Sequence	zgflqn	***Solvent***	CHLOROFORM-D	***Sweep Width (Hz)***	75187.97
Temperature (degree C)	27.000				

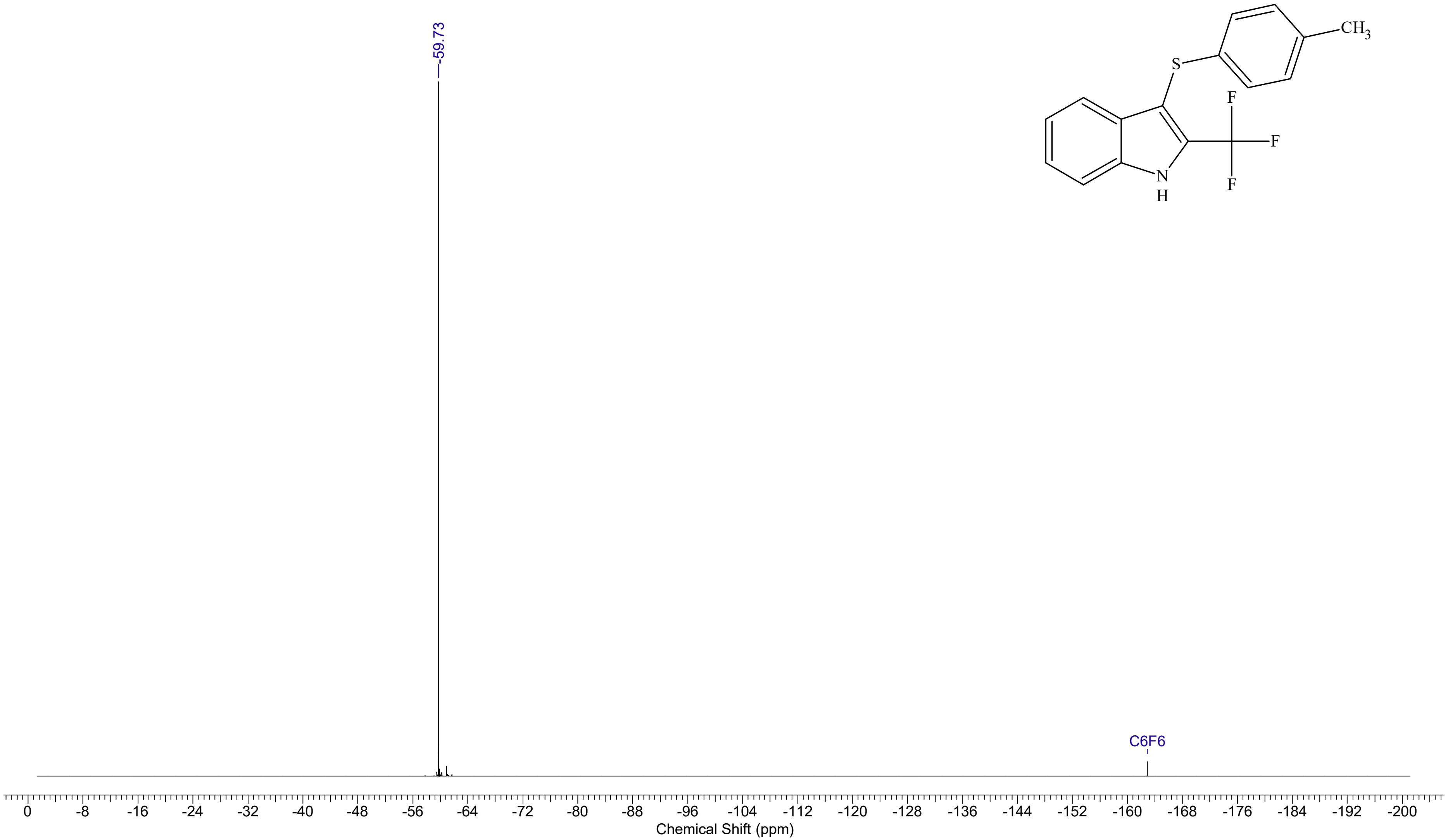

^{19}F NMR spectrum of **9** (376.5 MHz, $CDCl_3$)

26 Aug 2021

Acquisition Time (sec)	0.6783	Comment	Imported from UXNMR.			Date	16 Aug 2021 15:16:48
File Name	C:\DOCS\OUTPUT_301\2021\08.август\BM-2213-2.C_002001r					Frequency (MHz)	100.61
Nucleus	13C	Number of Transients	201	Original Points Count	16384	Points Count	131072
Pulse Sequence	zgpg30	Solvent	CHLOROFORM-D			Sweep Width (Hz)	24154.59
Temperature (degree C)	27.000						

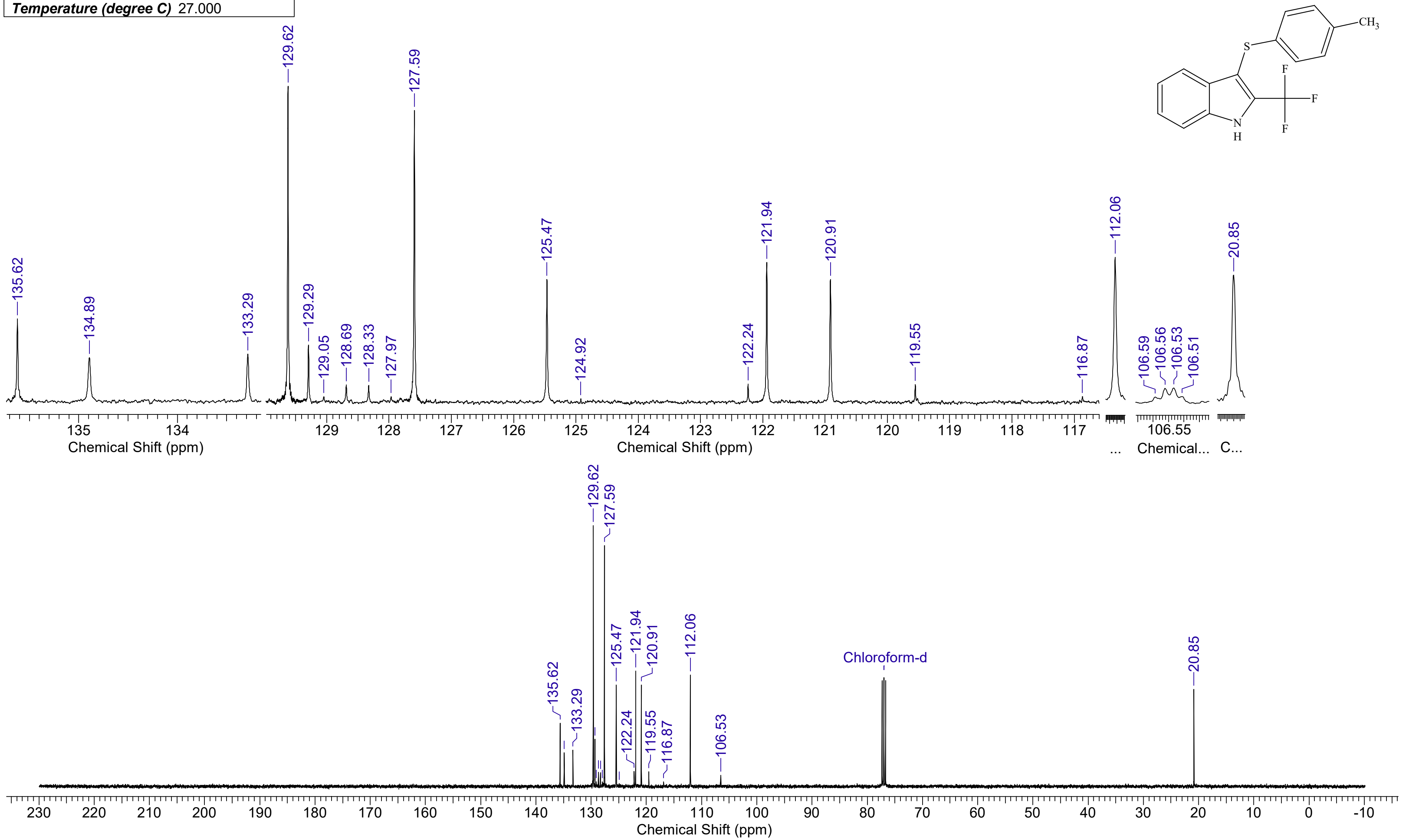

$^{13}C\{^1H\}$ NMR spectrum of **9** (100.6 MHz, $CDCl_3$)

7 Oct 2022

Acquisition Time (sec)	4.0894	***Comment***	Imported from UXNMR.	***Date***	04 Oct 2022 15:39:36
File Name	C:\DOCS\OUTPUT_301\2022\10.октябрь\SZA-BM-2633-11.H_001001r			***Frequency (MHz)***	400.13
Nucleus	1H	***Number of Transients***	4 ***Original Points Count*** 32768	***Points Count***	131072
Pulse Sequence	zg30	***Solvent***	CHLOROFORM-D	***Sweep Width (Hz)***	8012.82
Temperature (degree C)	27.000				

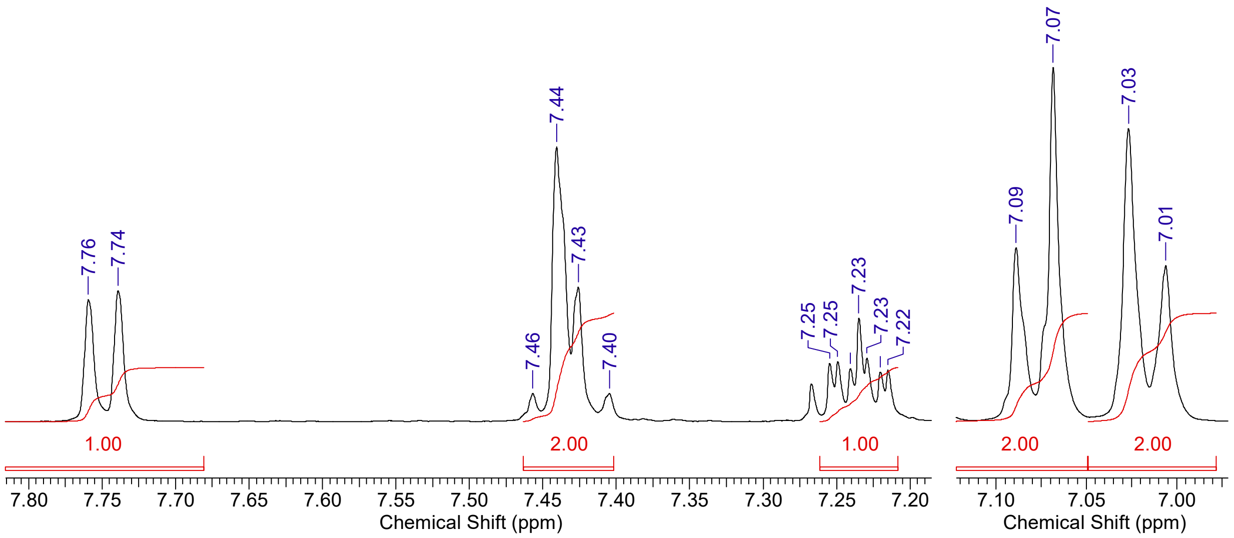

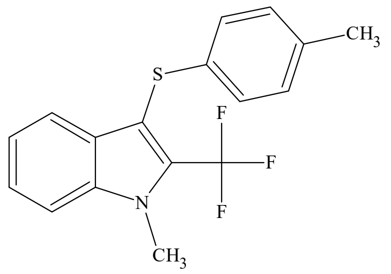

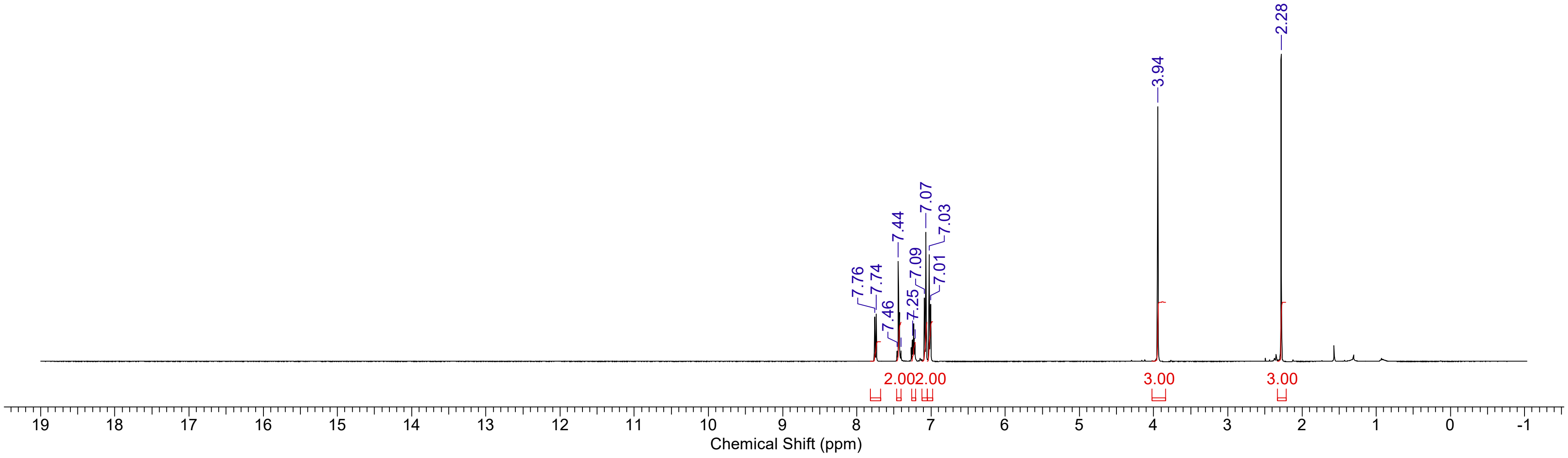

^{1}H NMR spectrum of **10** (400.1 MHz, $CDCl_3$)

7 Oct 2022

Acquisition Time (sec)	1.7433	***Comment***	Imported from UXNMR.			***Date***	04 Oct 2022 15:38:24
File Name	C:\DOCS\OUTPUT_301\2022\10.октябрь\SZA-BM-2633-11.F_005001r					***Frequency (MHz)***	376.50
Nucleus	19F	***Number of Transients***	8	***Original Points Count***	131072	***Points Count***	262144
Pulse Sequence	zgflqn	***Solvent***	CHLOROFORM-D			***Sweep Width (Hz)***	75187.97
Temperature (degree C)	27.000						

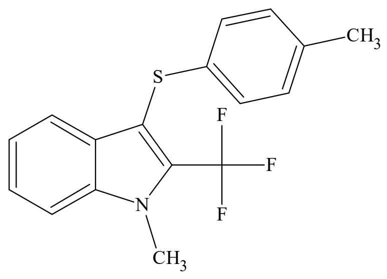

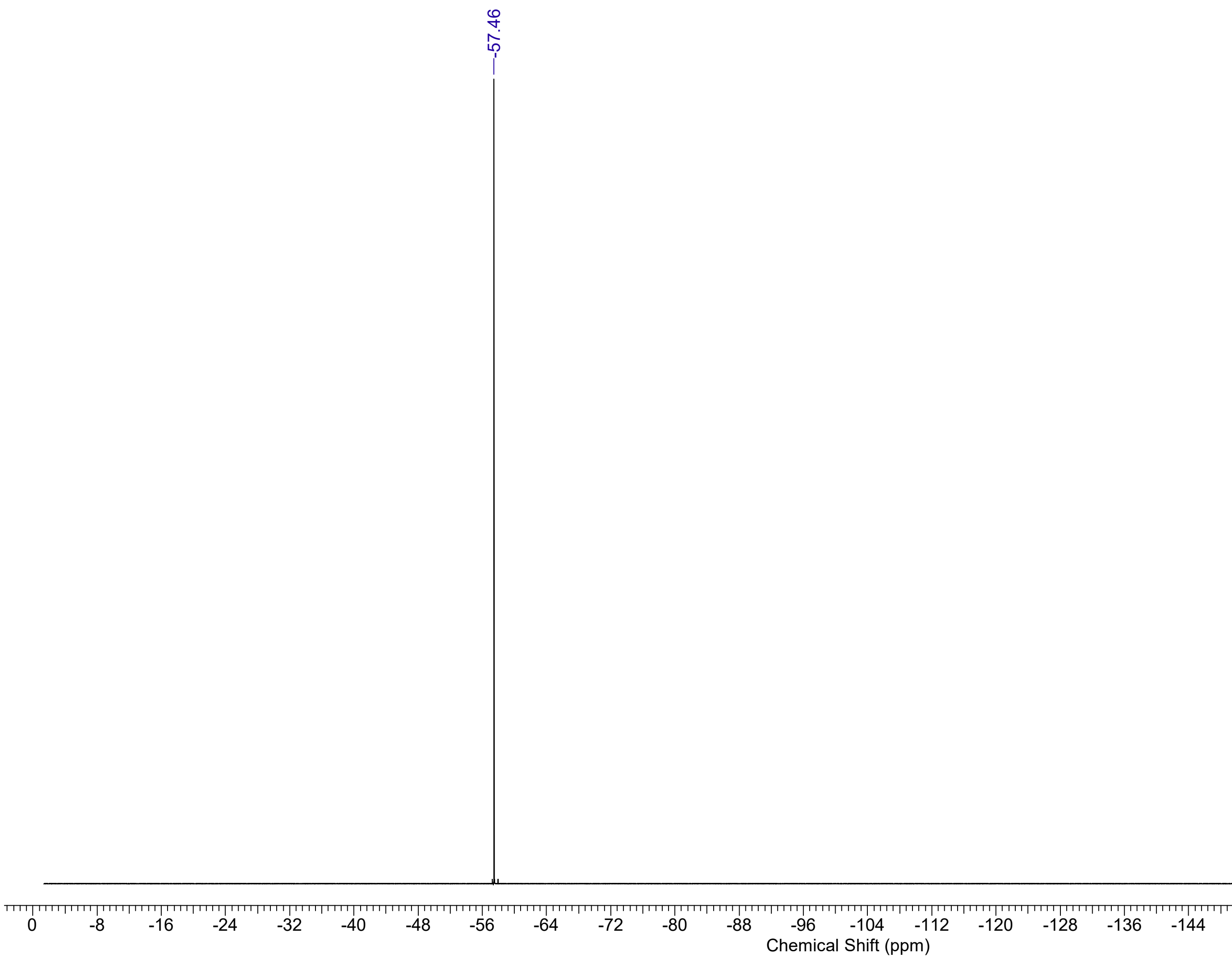

^{19}F NMR spectrum of **10** (376.5 MHz, $CDCl_3$)

7 Oct 2022

Acquisition Time (sec) 0.6783	***Comment***	Imported from UXNMR.		***Date***	05 Oct 2022 17:58:08
File Name	C:\BM_DATA\DOCS\05.10.22\05.10.22\SZA-BM-2633-11.C_002001r			***Frequency (MHz)***	100.61
Nucleus 13C	***Number of Transients*** 321	***Original Points Count*** 16384		***Points Count***	131072
Pulse Sequence zgpg30	***Solvent***	CHLOROFORM-D		***Sweep Width (Hz)***	24154.59
Temperature (degree C) 27.000					

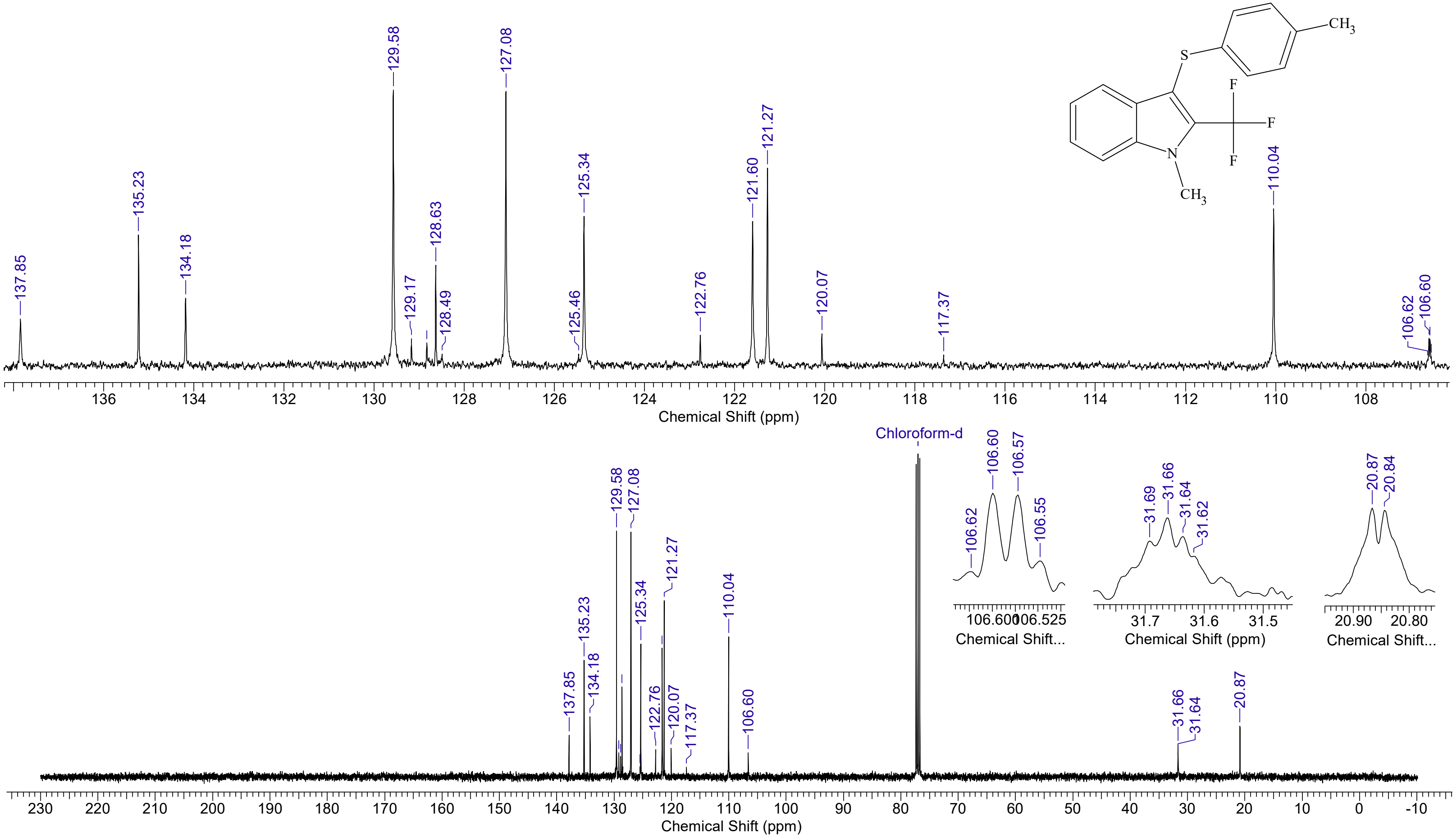

$^{13}C\{^1H\}$ NMR spectrum of **10** (100.6 MHz, $CDCl_3$)

Acquisition Time (sec)	4.0894	***Comment***	Imported from UXNMR.			***Date***	21 Jun 2021 15:13:24
File Name	C:\DOCS\OUTPUT_301\2021\06.июнь\SZA-BM-2176-4.H_001001r					***Frequency (MHz)***	400.13
Nucleus	1H	***Number of Transients***	4	***Original Points Count***	32768	***Points Count***	131072
Pulse Sequence	zg30	***Solvent***	ACETONITRILE-D3			***Sweep Width (Hz)***	8012.82
Temperature (degree C)	27.000						

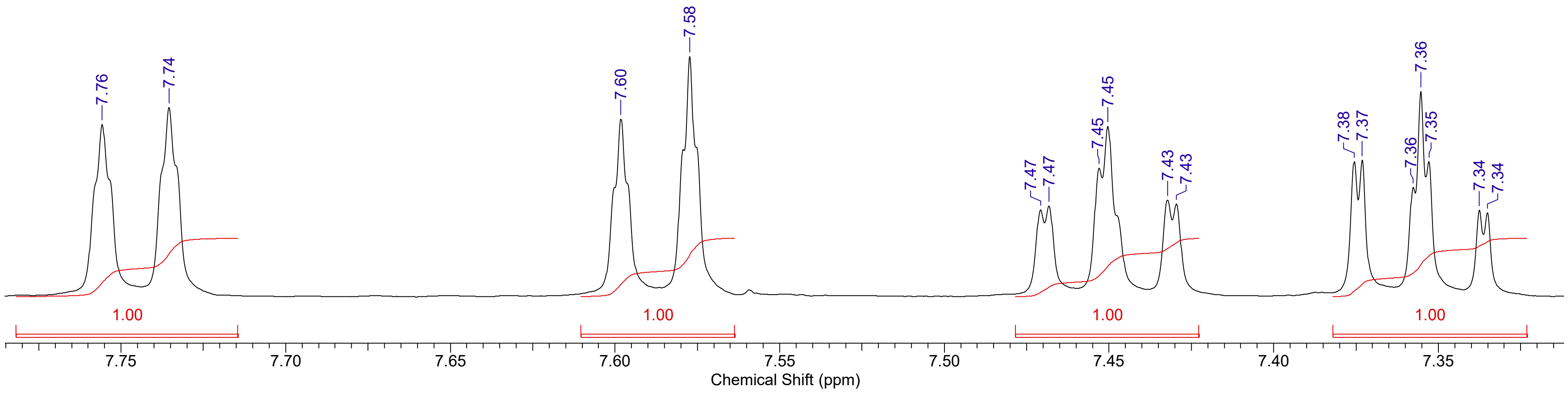

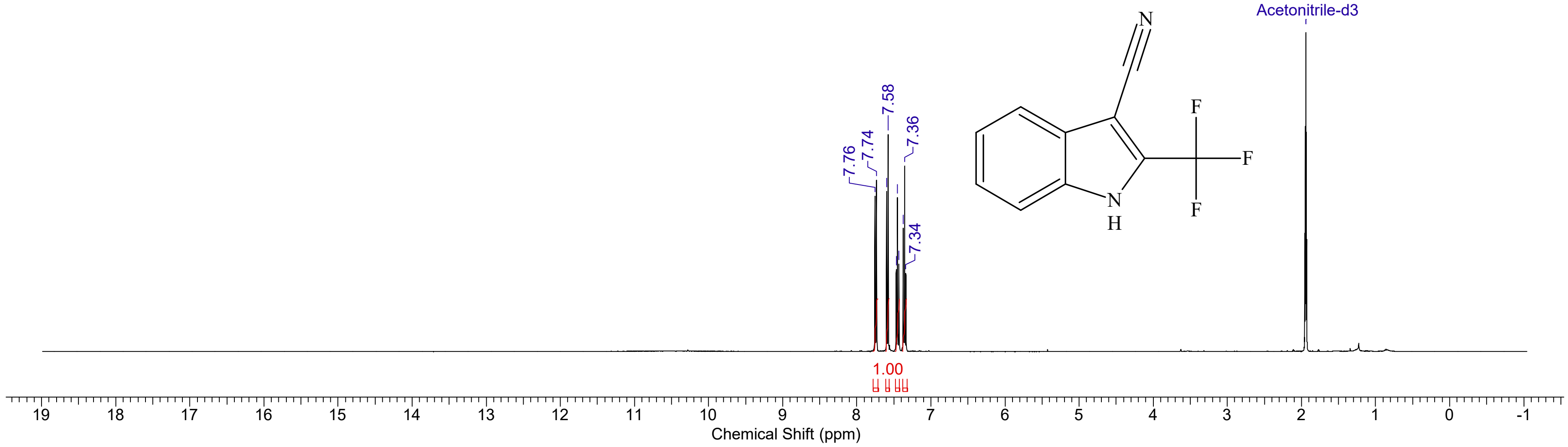

^{1}H NMR spectrum of **11** (400.1 MHz, CD_3CN)

1 Aug 2021

Acquisition Time (sec)	1.7433	***Comment***	Imported from UXNMR.			***Date***	21 Jun 2021 15:17:22
File Name	C:\DOCS\OUTPUT_301\2021\06.июнь\SZA-BM-2176-4.F_005001r					***Frequency (MHz)***	376.50
Nucleus	19F	***Number of Transients***	16	***Original Points Count***	131072	***Points Count***	262144
Pulse Sequence	zgflqn	***Solvent***	CHLOROFORM-D			***Sweep Width (Hz)***	75187.97
Temperature (degree C)	27.000						

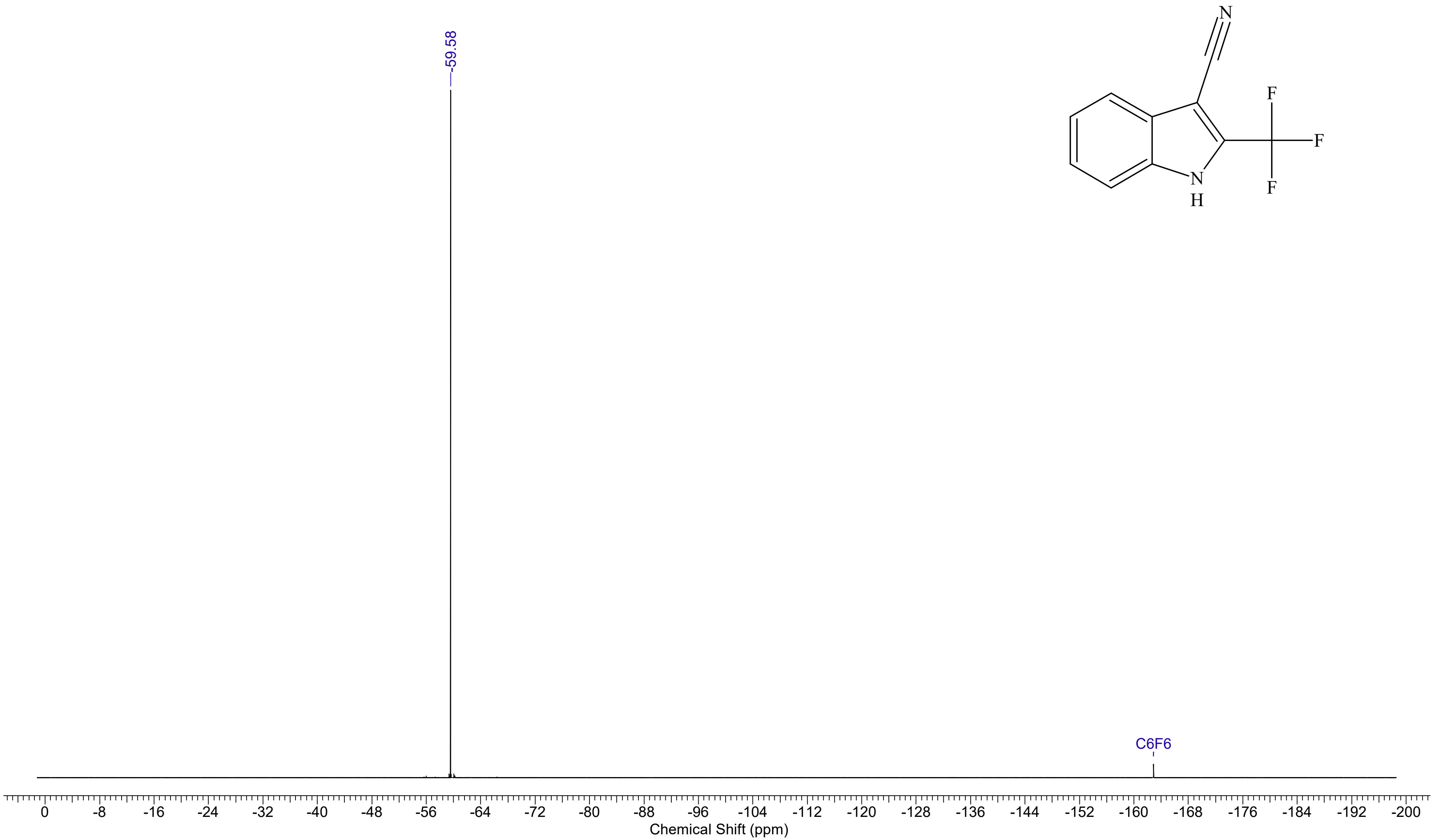

^{19}F NMR spectrum of **11** (376.5 MHz, $CDCl_3$)

1 Aug 2021

Acquisition Time (sec)	0.6783	Comment	Imported from UXNMR.			Date	22 Jun 2021 14:23:46
File Name	C:\DOCS\OUTPUT_301\2021\06.июнь\SZA-BM-2176-4.C_002001r					Frequency (MHz)	100.61
Nucleus	13C	Number of Transients	266	Original Points Count	16384	Points Count	131072
Pulse Sequence	zgpg30	Solvent	ACETONITRILE-D3			Sweep Width (Hz)	24154.59
Temperature (degree C)	27.000						

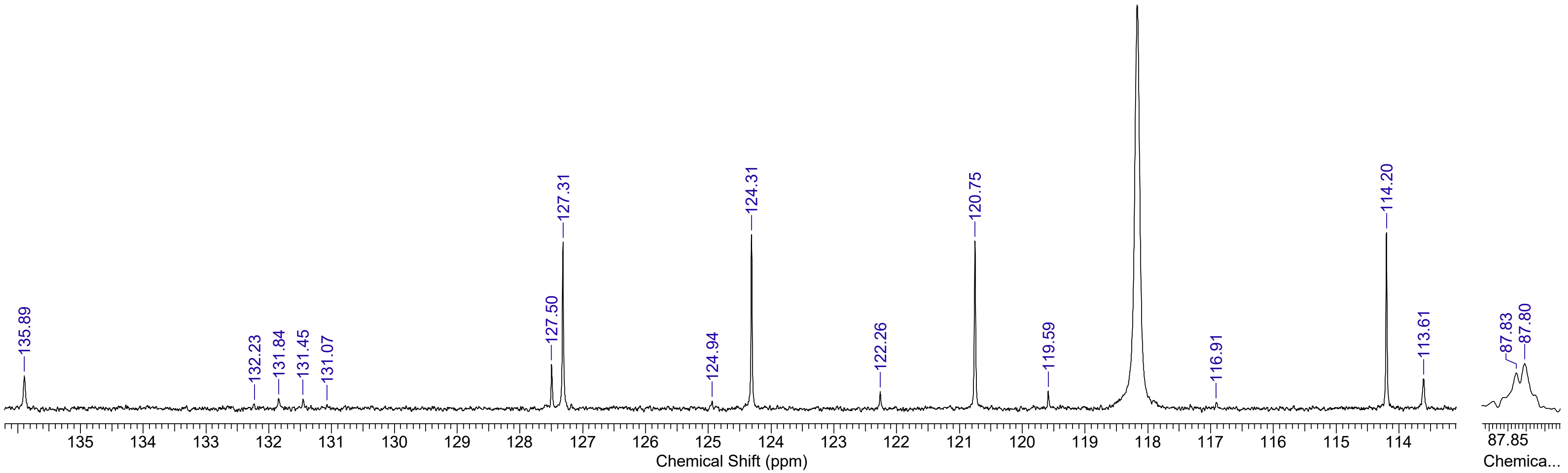

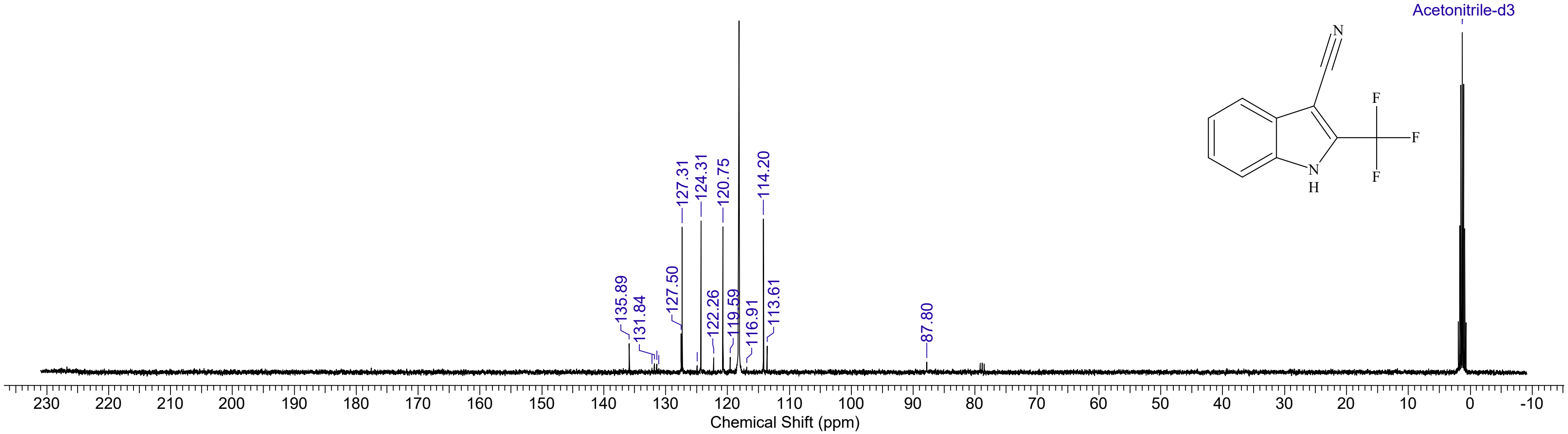

$^{13}C\{^{1}H\}$ NMR spectrum of **11** (100.6 MHz, CD_3CN)

7 Oct 2022

Acquisition Time (sec)	4.0894	Comment	Imported from UXNMR.			Date	23 Sep 2022 15:25:42
File Name	C:\DOCS\OUTPUT_301\2022\09.сентябрь\BM-2611.H_001001r					Frequency (MHz)	400.13
Nucleus	1H	Number of Transients	4	Original Points Count	32768	Points Count	131072
Pulse Sequence	zg30	Solvent	CHLOROFORM-D			Sweep Width (Hz)	8012.82
Temperature (degree C)	27.000						

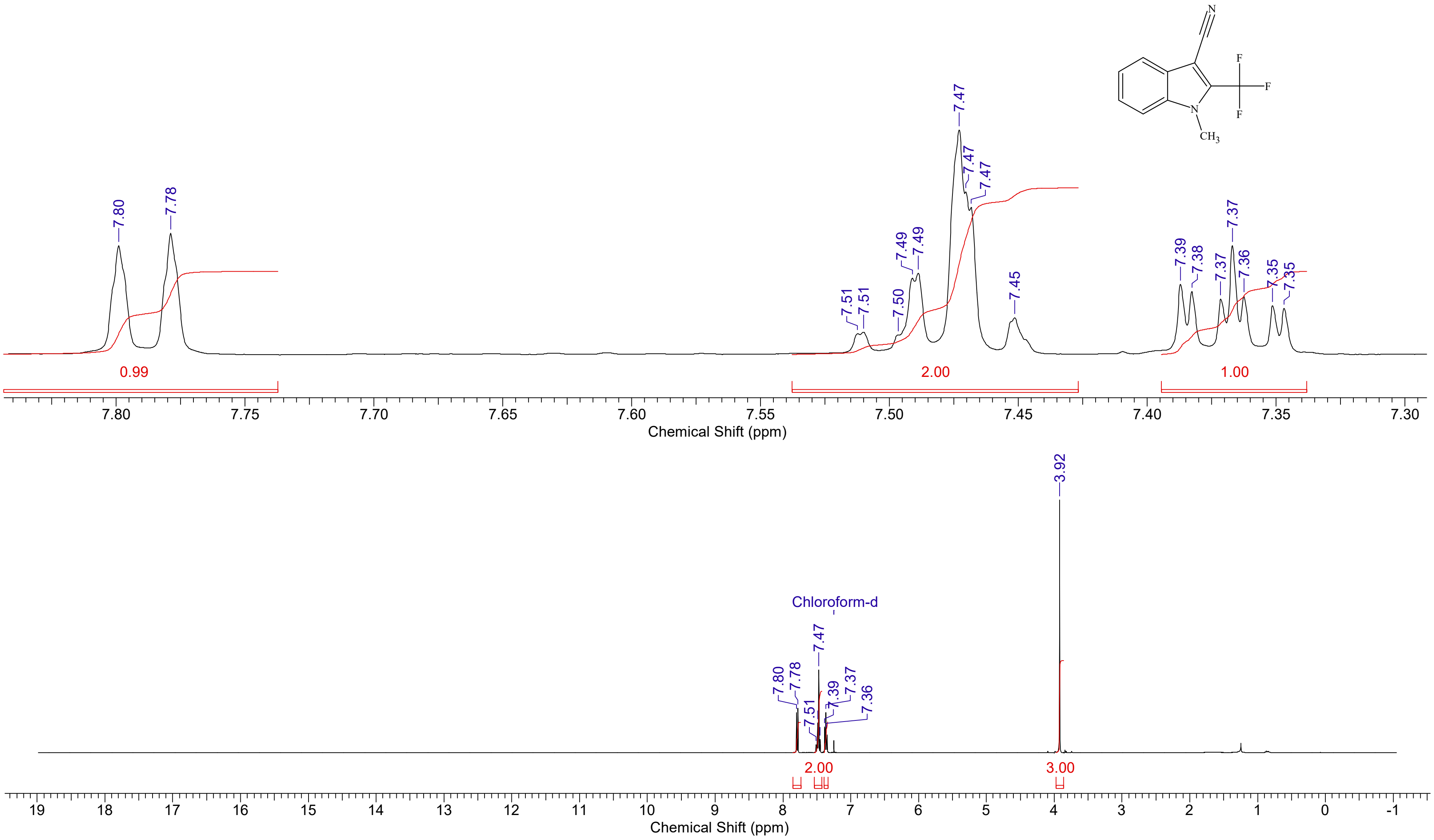

^{1}H NMR spectrum of **12** (400.1 MHz, $CDCl_3$)

7 Oct 2022

Acquisition Time (sec)	1.7433	***Comment***	Imported from UXNMR.			***Date***	23 Sep 2022 15:37:38
File Name	C:\DOCS\OUTPUT_301\2022\09.сентябрь\BM-2611.F_005001r					***Frequency (MHz)***	376.50
Nucleus	19F	***Number of Transients***	16	***Original Points Count***	131072	***Points Count***	262144
Pulse Sequence	zgflqn	***Solvent***	CHLOROFORM-D			***Sweep Width (Hz)***	75187.97
Temperature (degree C)	27.000						

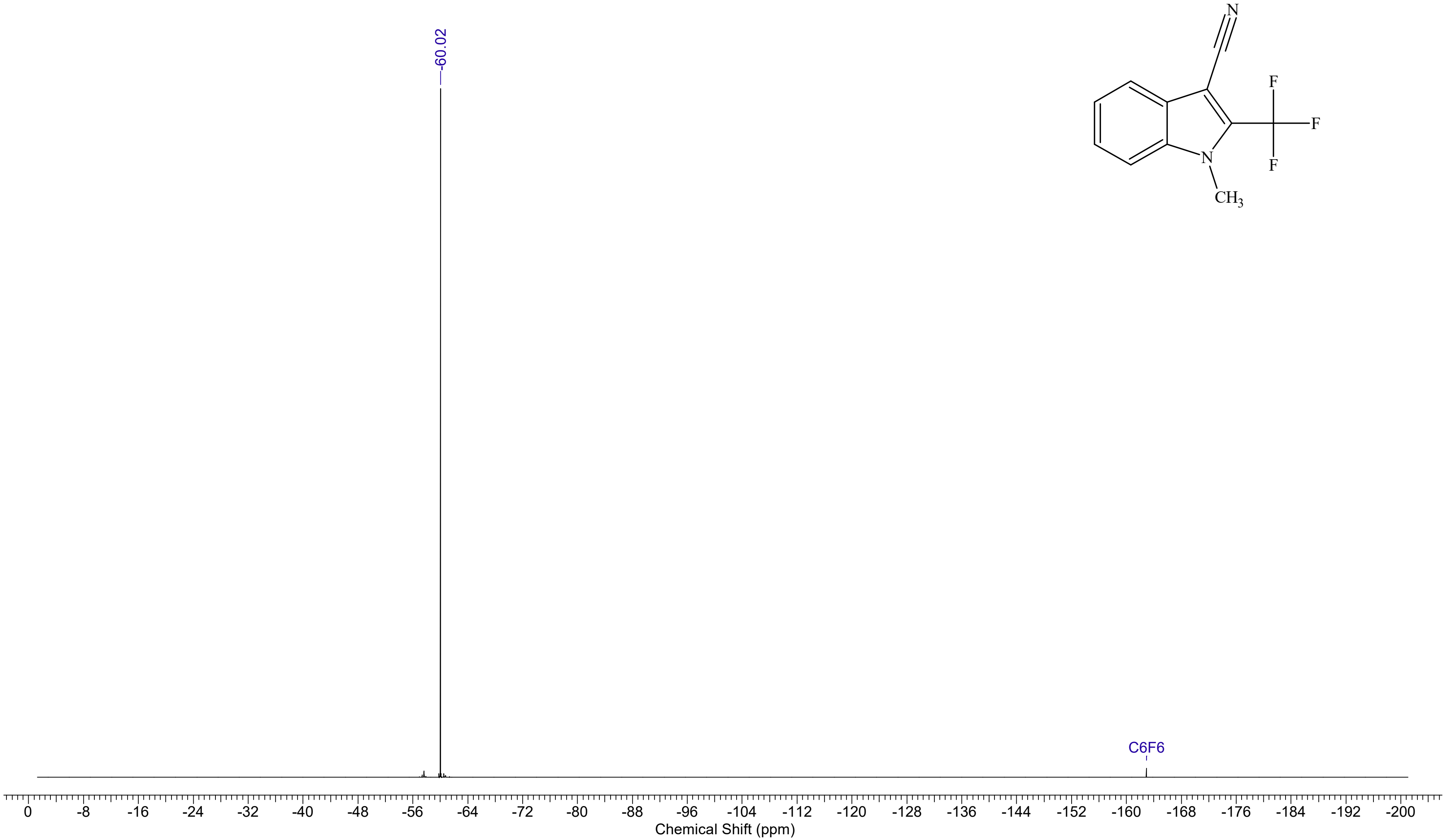

^{19}F NMR spectrum of **12** (376.5 MHz, $CDCl_3$)

7 Oct 2022

Acquisition Time (sec)	0.6783	***Comment***	Imported from UXNMR.			***Date***	23 Sep 2022 15:34:08
File Name	C:\DOCS\OUTPUT_301\2022\09.сентябрь\BM-2611.C_002001r					***Frequency (MHz)***	100.61
Nucleus	13C	***Number of Transients***	201	***Original Points Count***	16384	***Points Count***	131072
Pulse Sequence	zgpg30	***Solvent***	CHLOROFORM-D			***Sweep Width (Hz)***	24154.59
Temperature (degree C)	27.000						

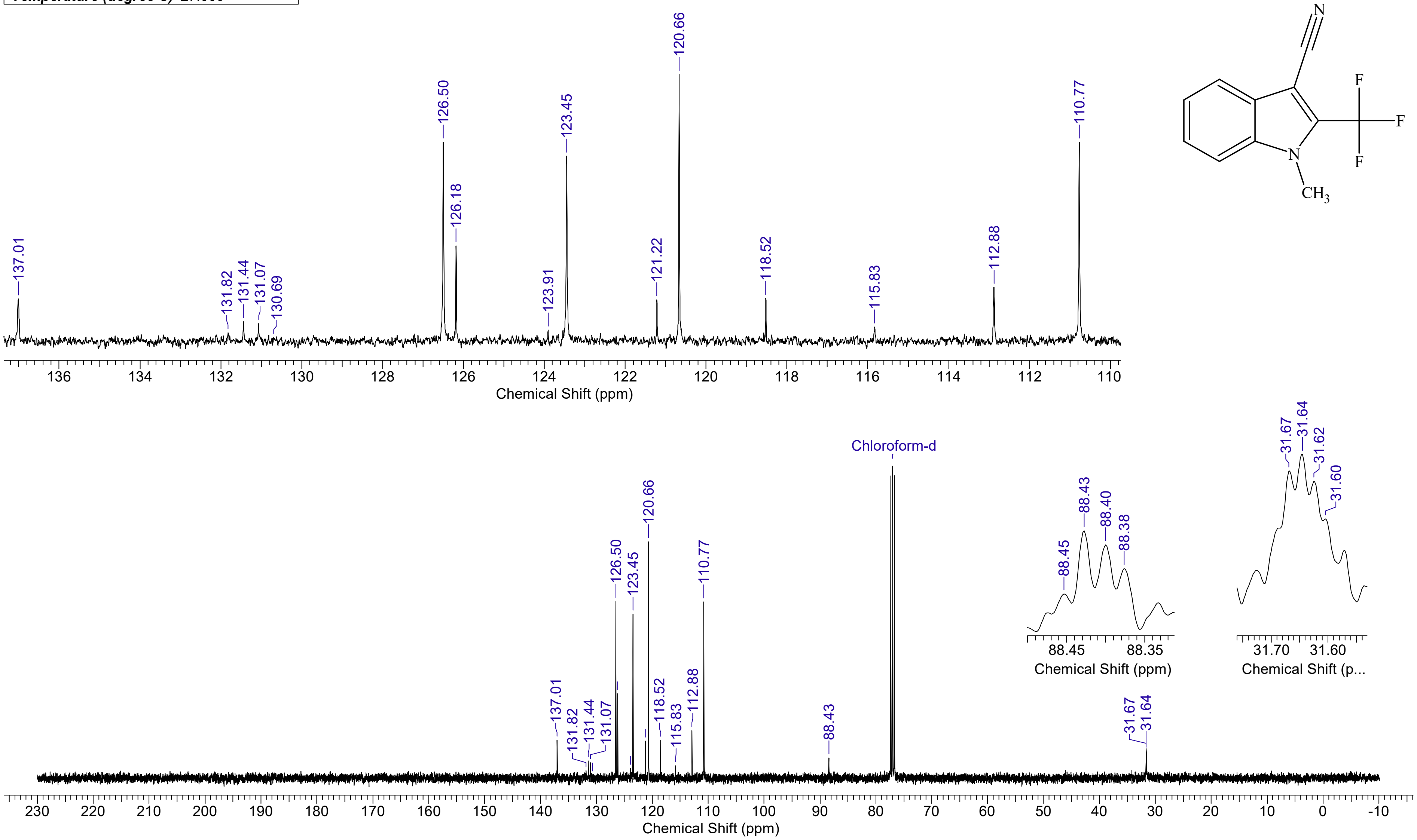

$^{13}C\{^{1}H\}$ NMR spectrum of **12** (100.6 MHz, $CDCl_3$)

7 Oct 2022

Acquisition Time (sec)	4.0894	***Comment***	Imported from UXNMR.	***Date***	16 Sep 2022 15:13:28
File Name	C:\DOCS\OUTPUT_301\2022\09.сентябрь\BM-2592-p.H_001001r			***Frequency (MHz)***	400.13
Nucleus	1H	***Number of Transients***	4 ***Original Points Count*** 32768	***Points Count***	131072
Pulse Sequence	zg30	***Solvent***	CHLOROFORM-D	***Sweep Width (Hz)***	8012.82
Temperature (degree C)	27.000				

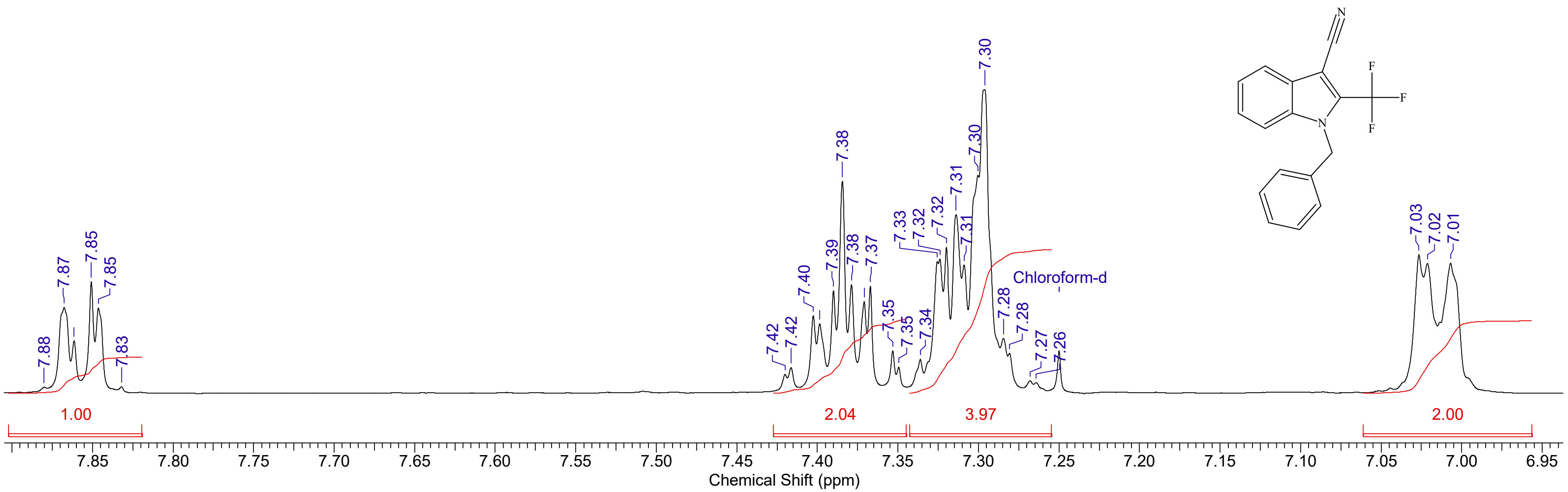

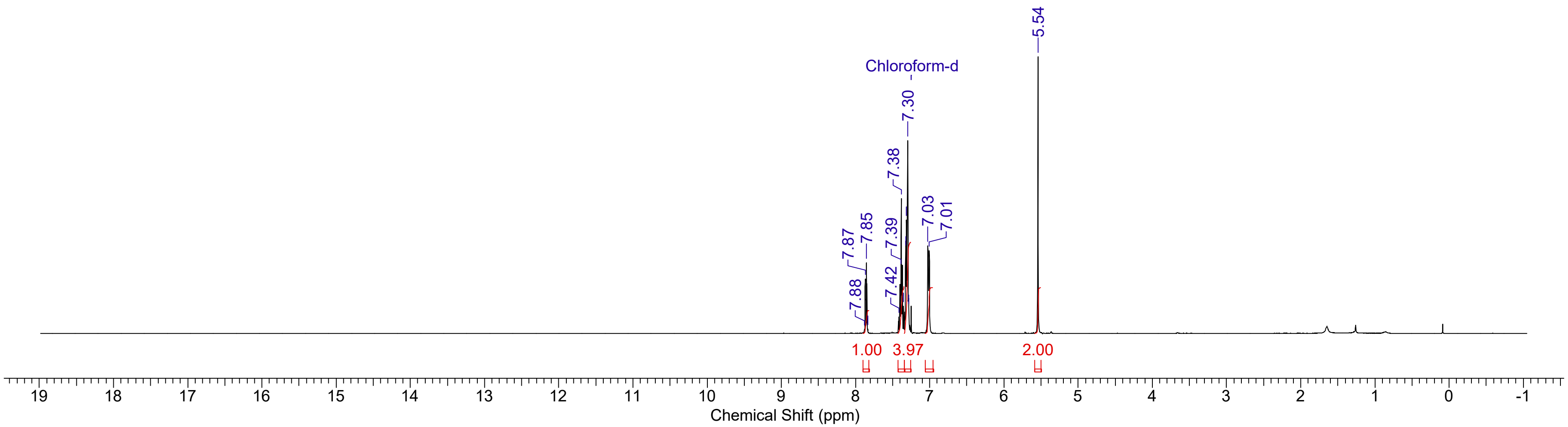

^{1}H NMR spectrum of **13** (400.1 MHz, $CDCl_3$)

7 Oct 2022

Acquisition Time (sec)	1.7433	***Comment***	Imported from UXNMR.			***Date***	16 Sep 2022 15:46:58
File Name	C:\DOCS\OUTPUT_301\2022\09.сентябрь\BM-2592-p.F_005001r					***Frequency (MHz)***	376.50
Nucleus	19F	***Number of Transients***	16	***Original Points Count***	131072	***Points Count***	262144
Pulse Sequence	zgflqn	***Solvent***	CHLOROFORM-D			***Sweep Width (Hz)***	75187.97
Temperature (degree C)	27.000						

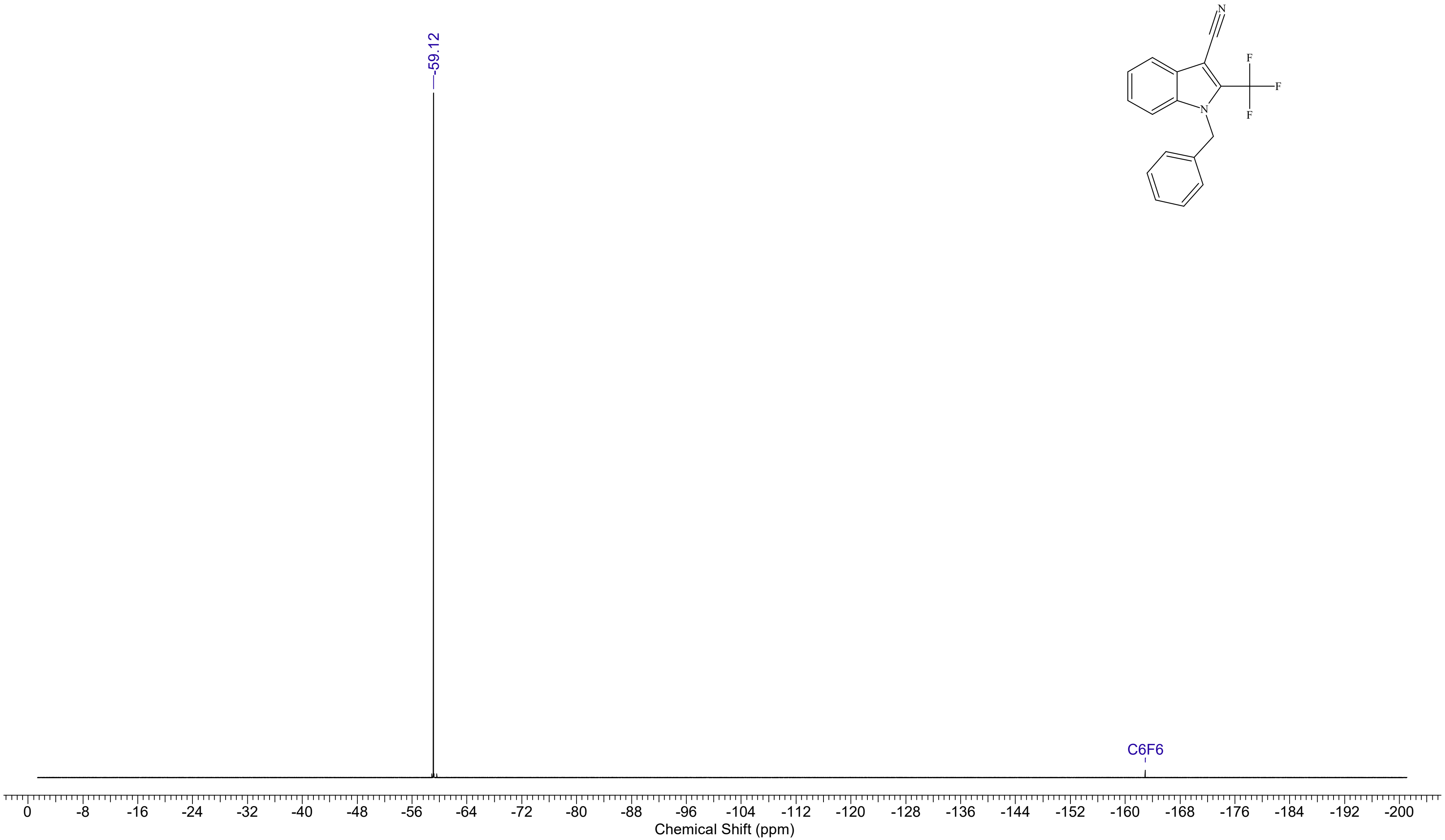

^{19}F NMR spectrum of **13** (376.5 MHz, $CDCl_3$)

7 Oct 2022

Acquisition Time (sec)	0.6783	***Comment***	Imported from UXNMR.			***Date***	17 Sep 2022 00:01:06
File Name	C:\DOCS\BM\ЯМР_СОСЕДИ\2022\bm220916\BM-2592-P_002001r					***Frequency (MHz)***	100.61
Nucleus	13C	***Number of Transients***	360	***Original Points Count***	16384	***Points Count***	131072
Pulse Sequence	zgpg30	***Solvent***	CHLOROFORM-D			***Sweep Width (Hz)***	24154.59
Temperature (degree C)	27.000						

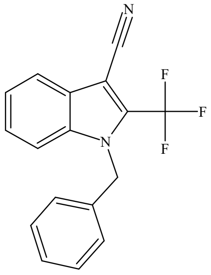

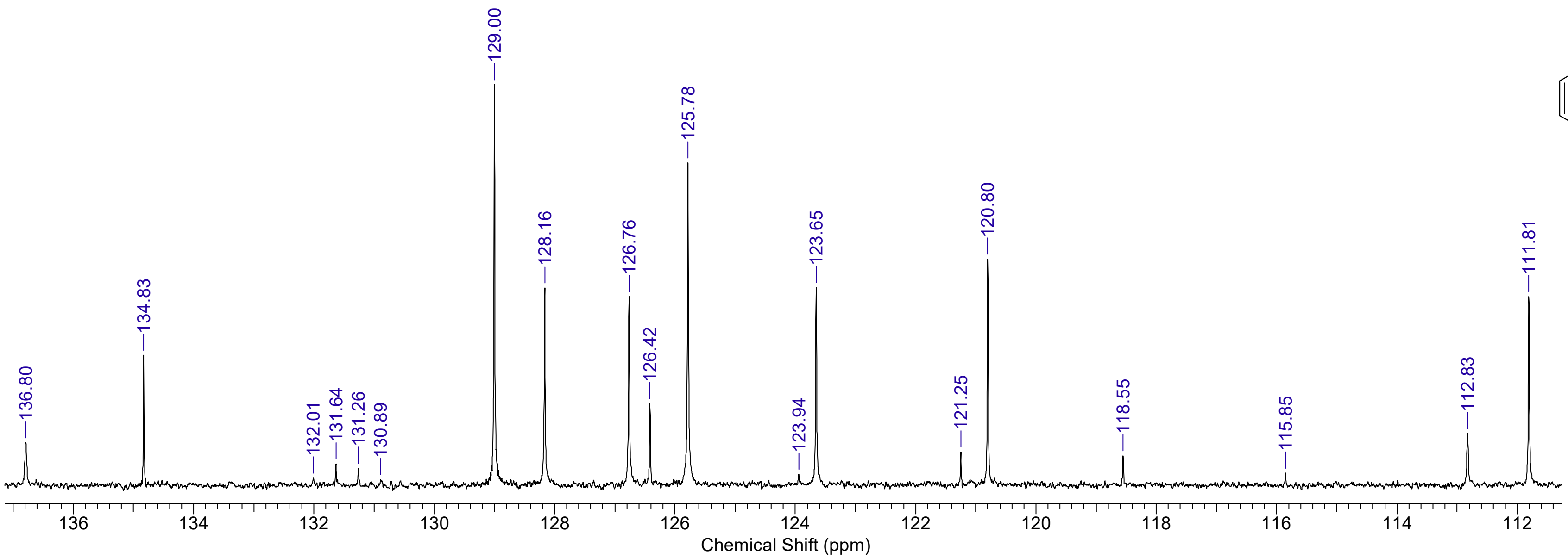

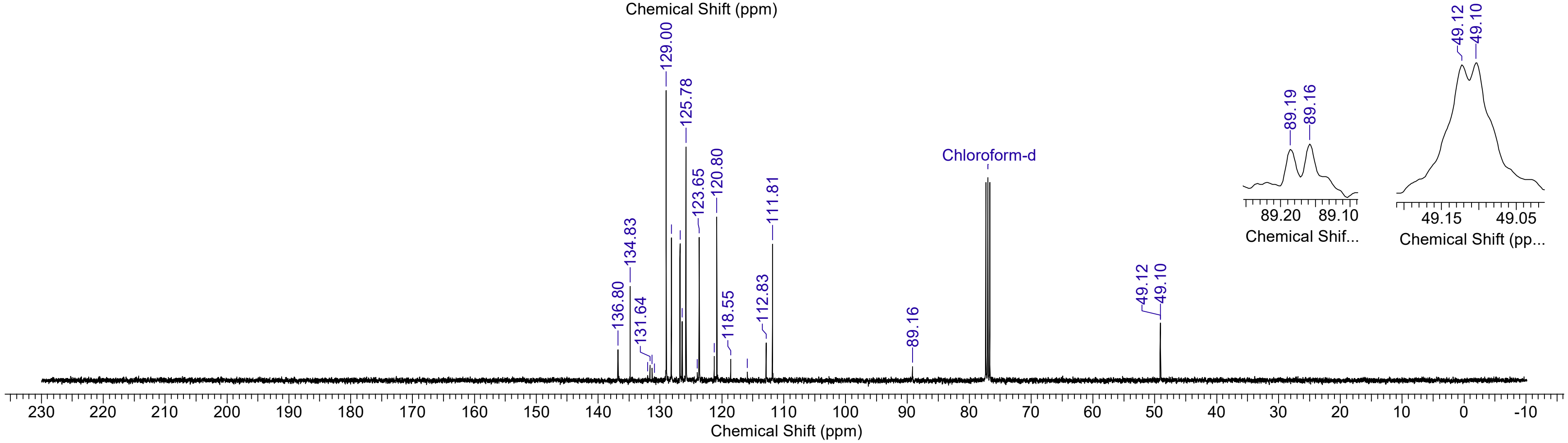

$^{13}C\{^1H\}$ NMR spectrum of **13** (100.6 MHz, $CDCl_3$)

1 Aug 2021

Acquisition Time (sec)	4.0894	Comment	Imported from UXNMR.			Date	10 Jun 2021 11:41:54
File Name	C:\BM_DATA\DOCS\10.06.2021\10.06.2021\SZA-BM-2189-6.H_001001r					Frequency (MHz)	400.13
Nucleus	1H	Number of Transients	4	Original Points Count	32768	Points Count	131072
Pulse Sequence	zg30	Solvent	CHLOROFORM-D			Sweep Width (Hz)	8012.82
Temperature (degree C)	27.000						

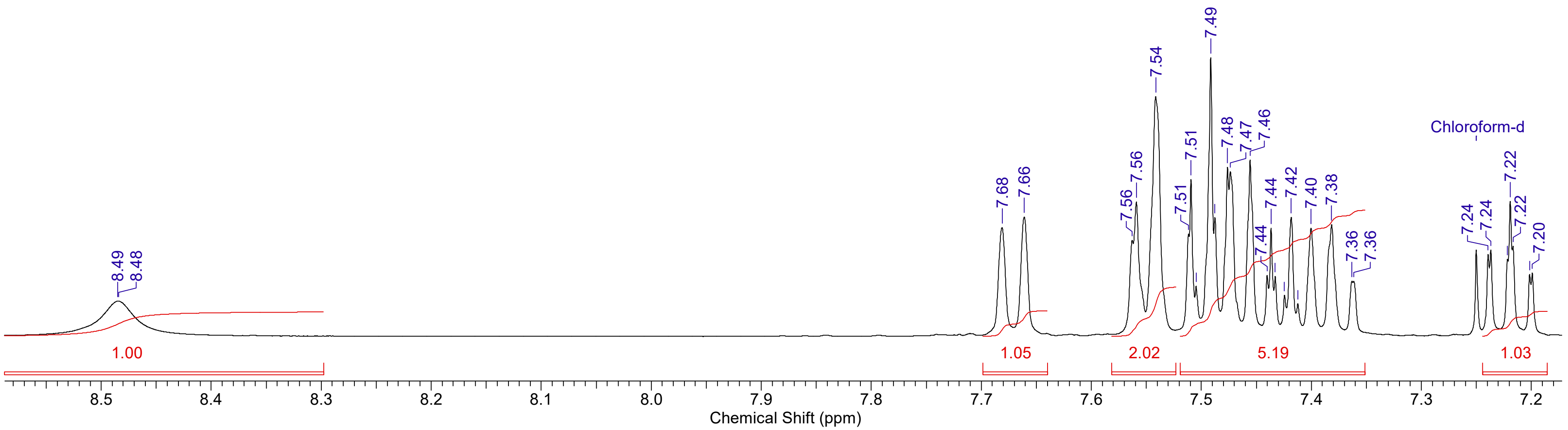

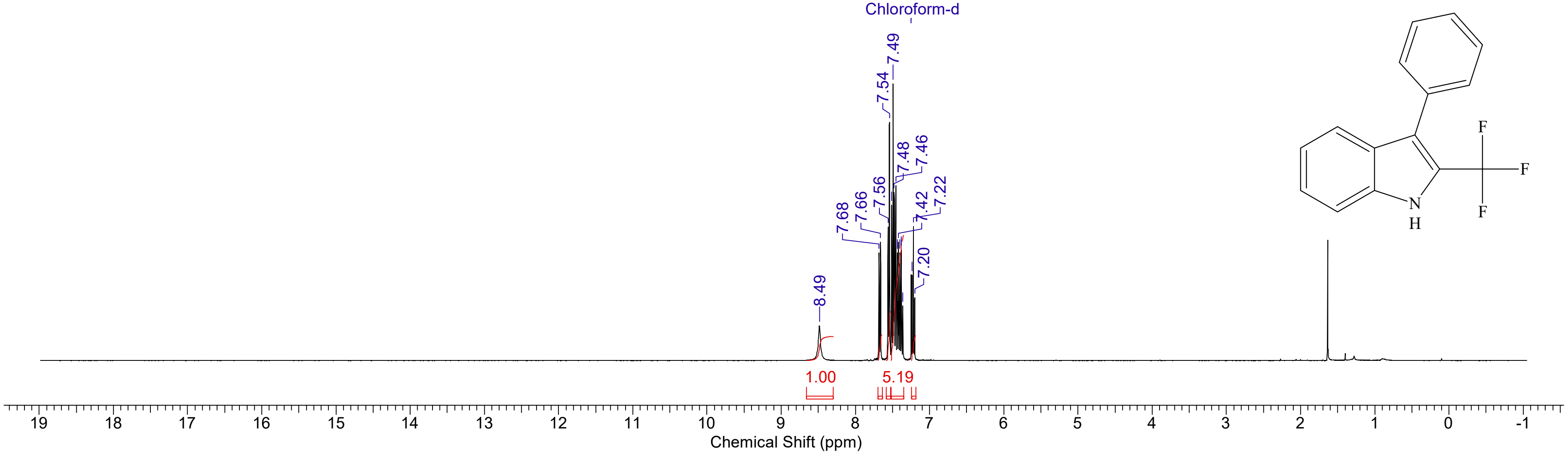

^{1}H NMR spectrum of **14** (400.1 MHz, $CDCl_3$)

1 Aug 2021

Acquisition Time (sec)	1.7433	***Comment***	Imported from UXNMR.	***Date***	10 Jun 2021 12:05:28
File Name	C:\BM_DATA\DOCS\10.06.2021\10.06.2021\SZA-BM-2189-6.F_005001r			***Frequency (MHz)***	376.50
Nucleus	19F	***Number of Transients***	16 ***Original Points Count*** 131072	***Points Count***	262144
Pulse Sequence	zgflqn	***Solvent***	CHLOROFORM-D	***Sweep Width (Hz)***	75187.97
Temperature (degree C)	27.000				

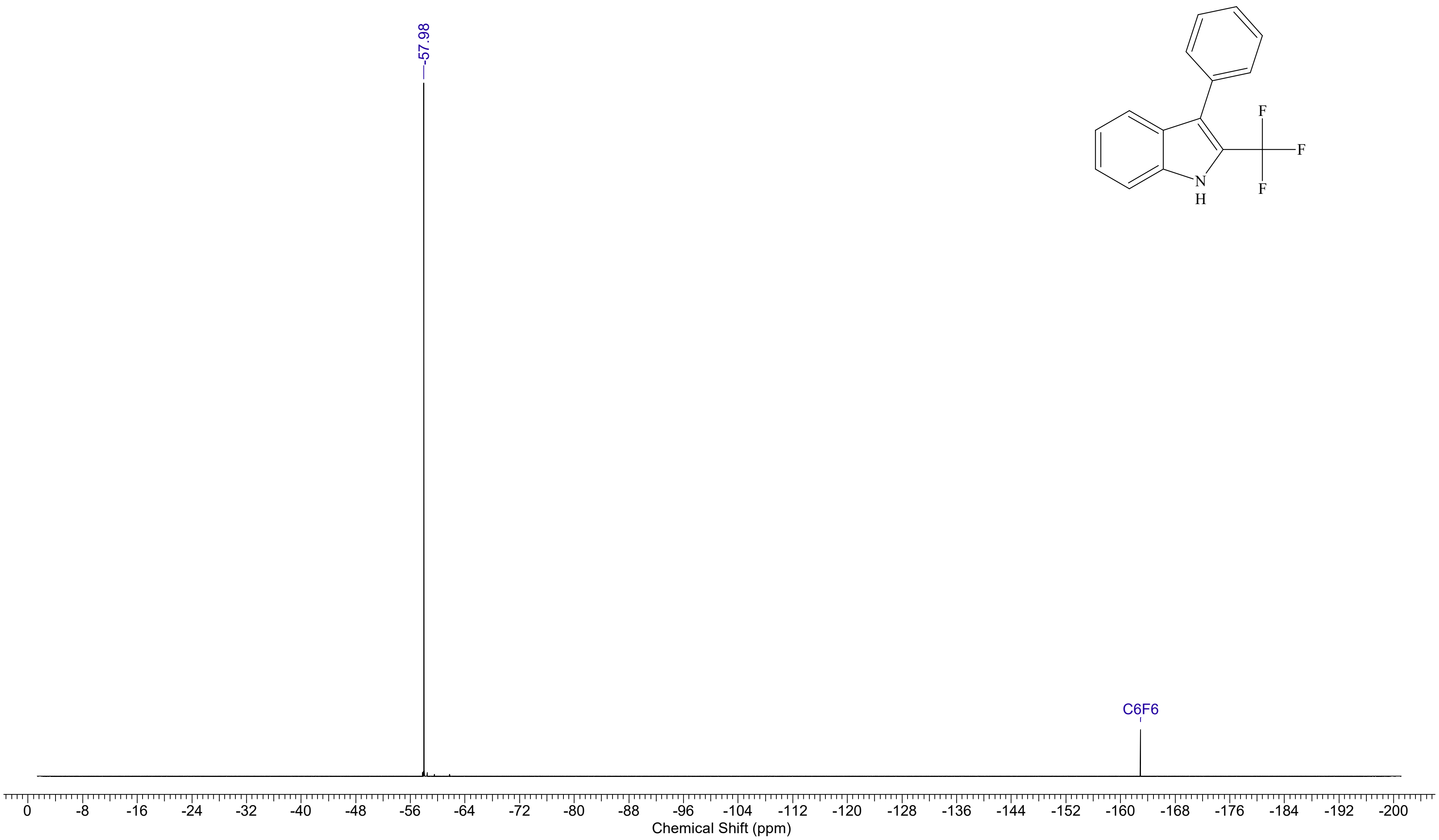

^{19}F NMR spectrum of **14** (376.5 MHz, $CDCl_3$)

1 Aug 2021

Acquisition Time (sec)	0.6783	***Comment***	Imported from UXNMR.			***Date***	11 Jun 2021 16:06:00
File Name	I:\SPEC_H,C_2021\06.июнь\SZA-BM-2189-6.C_002001r			***Frequency (MHz)***	100.61	***Nucleus***	13C
Number of Transients	494	***Original Points Count***	16384	***Points Count***	131072	***Pulse Sequence***	zgpg30
Solvent	DMSO-D6	***Sweep Width (Hz)***	24154.59	***Temperature (degree C)***	27.000		

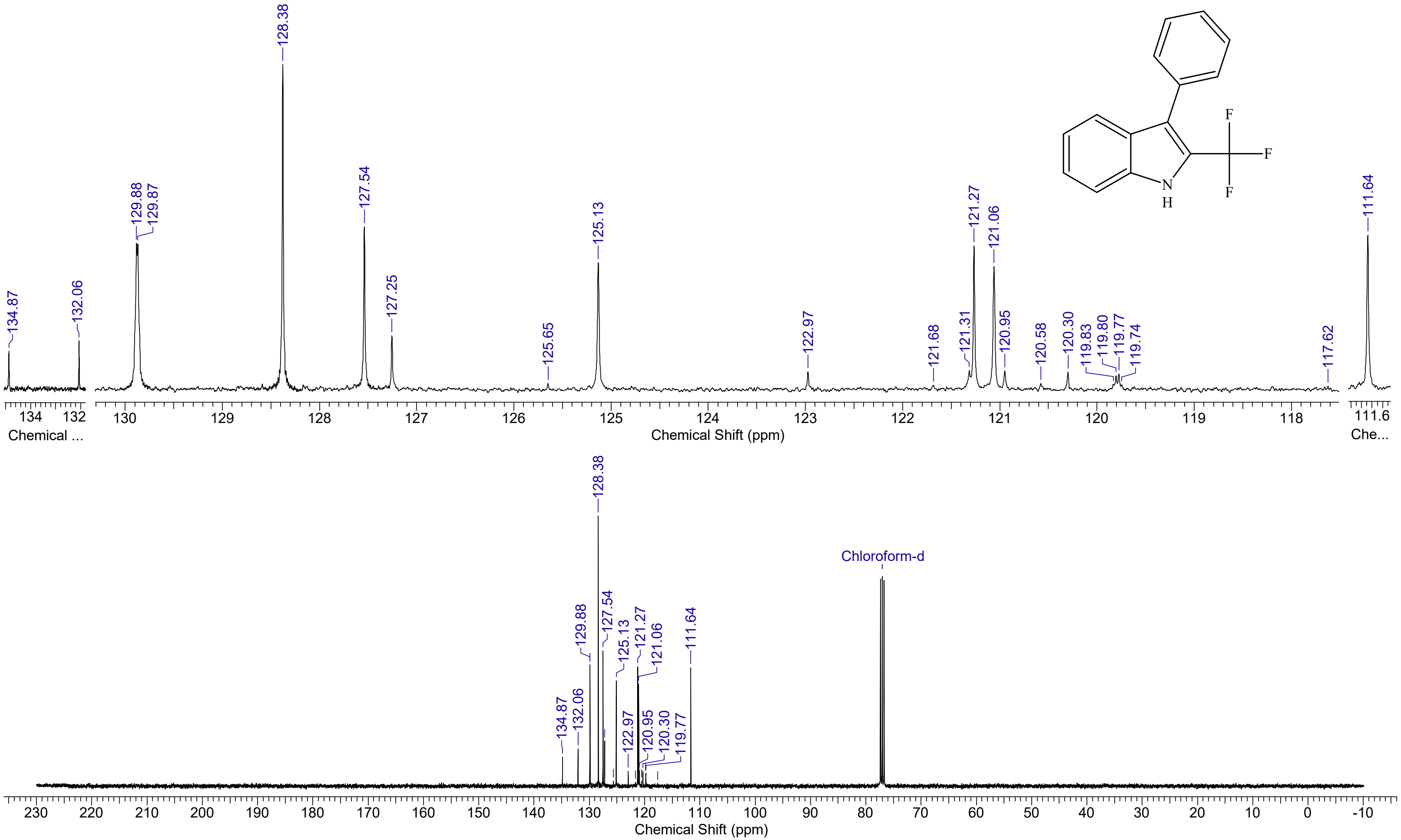

$^{13}C\{^1H\}$ NMR spectrum of **14** (100.6 MHz, $CDCl_3$)

7 Oct 2022

Acquisition Time (sec)	4.0894	Comment	Imported from UXNMR.			Date	30 Sep 2022 22:39:22
File Name	C:\DOCS\BM\ЯМР_СОСЕДИ\2022\BM-2622\BM-2622_001001r					Frequency (MHz)	400.13
Nucleus	1H	Number of Transients	8	Original Points Count	32768	Points Count	131072
Pulse Sequence	zg30	Solvent	CHLOROFORM-D			Sweep Width (Hz)	8012.82
Temperature (degree C)	27.000						

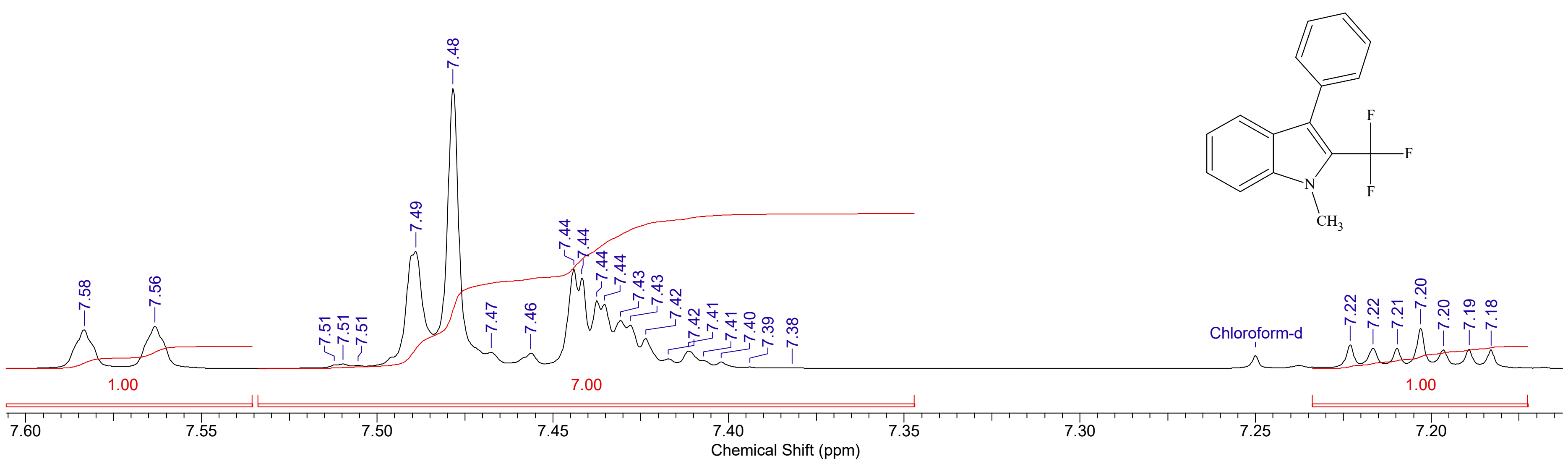

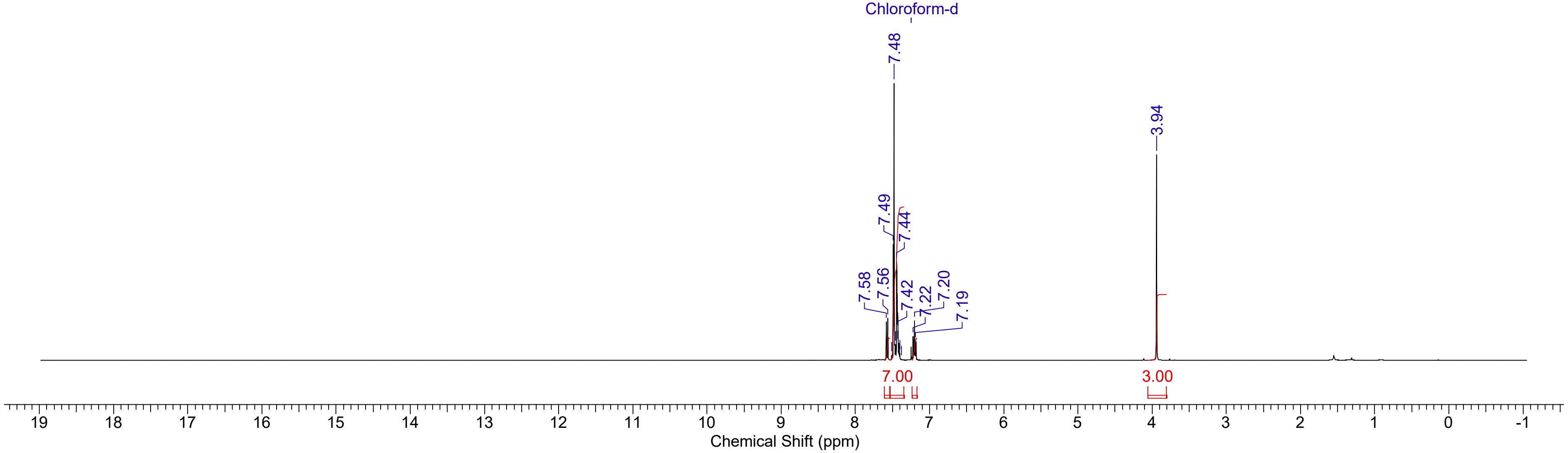

^{1}H NMR spectrum of **15** (400.1 MHz, $CDCl_3$)

Acquisition Time (sec)	1.7433	***Comment***	Imported from UXNMR.			***Date***	03 Oct 2022 15:34:38
File Name	C:\DOCS\OUTPUT_301\2022\10.октябрь\BM-2622.F_005001r					***Frequency (MHz)***	376.50
Nucleus	19F	***Number of Transients***	16	***Original Points Count***	131072	***Points Count***	262144
Pulse Sequence	zgflqn	***Solvent***	CHLOROFORM-D			***Sweep Width (Hz)***	75187.97
Temperature (degree C)	27.000						

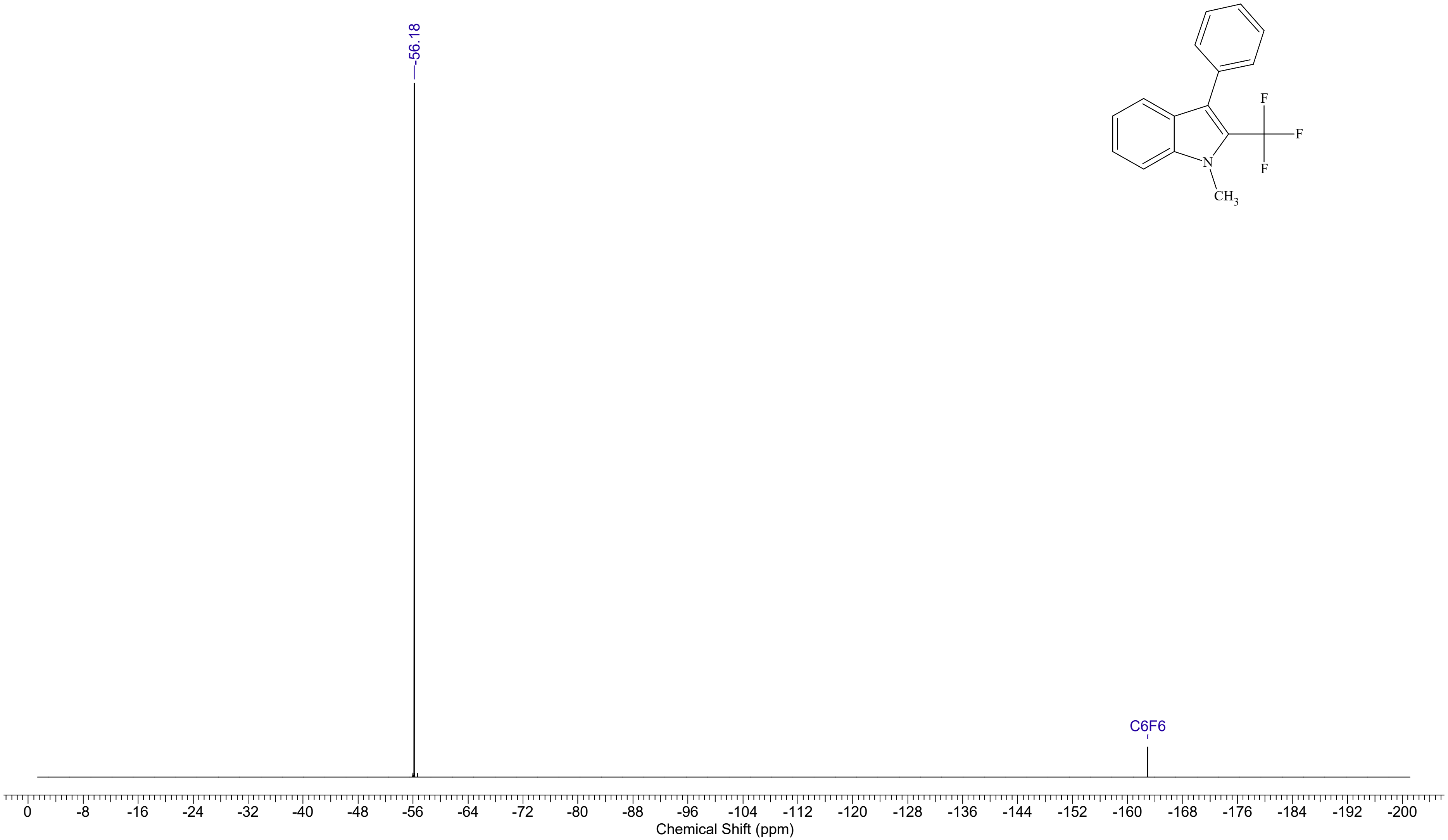

^{19}F NMR spectrum of **15** (376.5 MHz, $CDCl_3$)

Acquisition Time (sec)	0.6783	***Comment***	Imported from UXNMR.			***Date***	03 Oct 2022 15:31:02
File Name	C:\DOCS\OUTPUT_301\2022\10.октябрь\BM-2622.C_002001r					***Frequency (MHz)***	100.61
Nucleus	13C	***Number of Transients***	201	***Original Points Count***	16384	***Points Count***	131072
Pulse Sequence	zgpg30	***Solvent***	CHLOROFORM-D			***Sweep Width (Hz)***	24154.59
Temperature (degree C)	27.000						

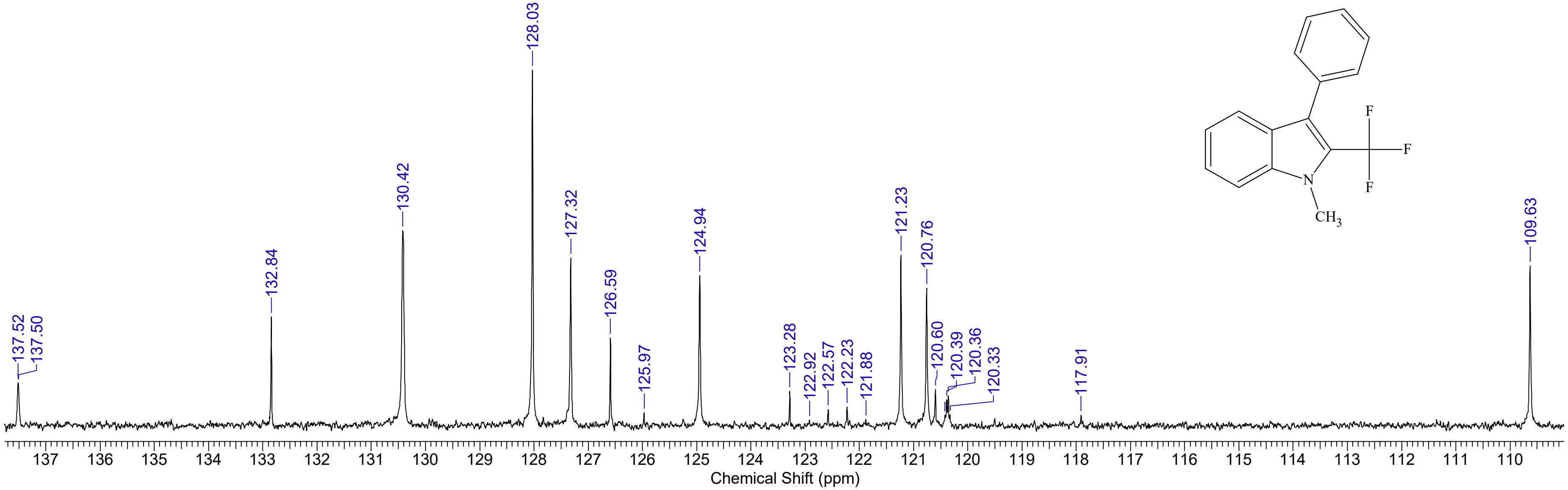

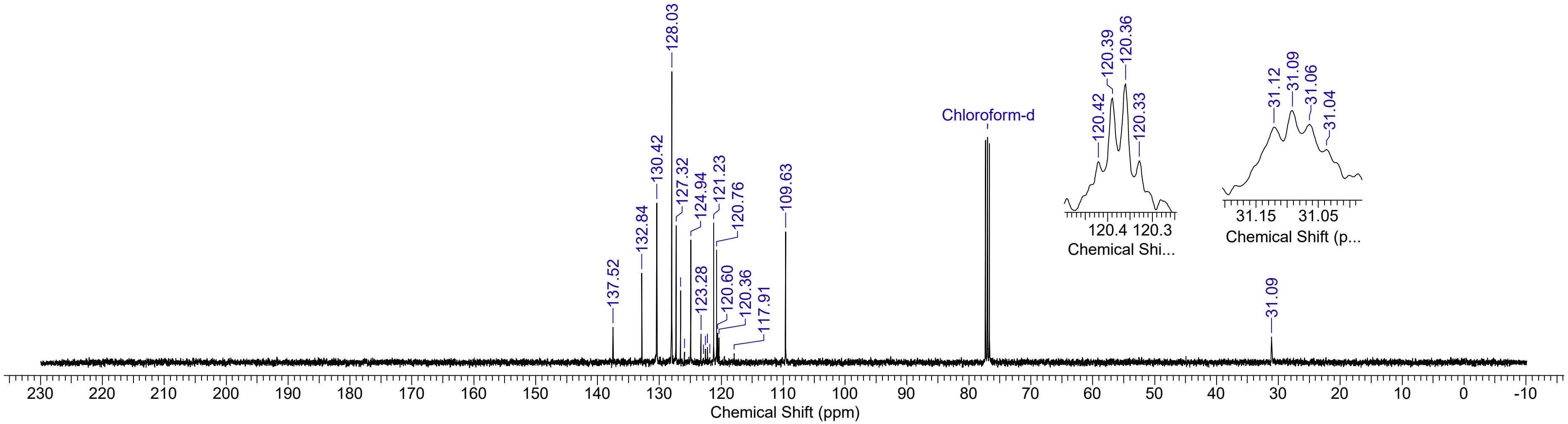

$^{13}C\{^1H\}$ NMR spectrum of **15** (100.6 MHz, $CDCl_3$)

6 Oct 2022

Acquisition Time (sec)	4.0894	***Comment***	Imported from UXNMR.	***Date***	05 Oct 2022 17:00:00
File Name	C:\BM_DATA\DOCS\05.10.22\05.10.22\SZA-BM-2628-12.H_001001r			***Frequency (MHz)***	400.13
Nucleus	1H	***Number of Transients***	4 ***Original Points Count*** 32768	***Points Count***	131072
Pulse Sequence	zg30	***Solvent***	CHLOROFORM-D	***Sweep Width (Hz)***	8012.82
Temperature (degree C)	27.000				

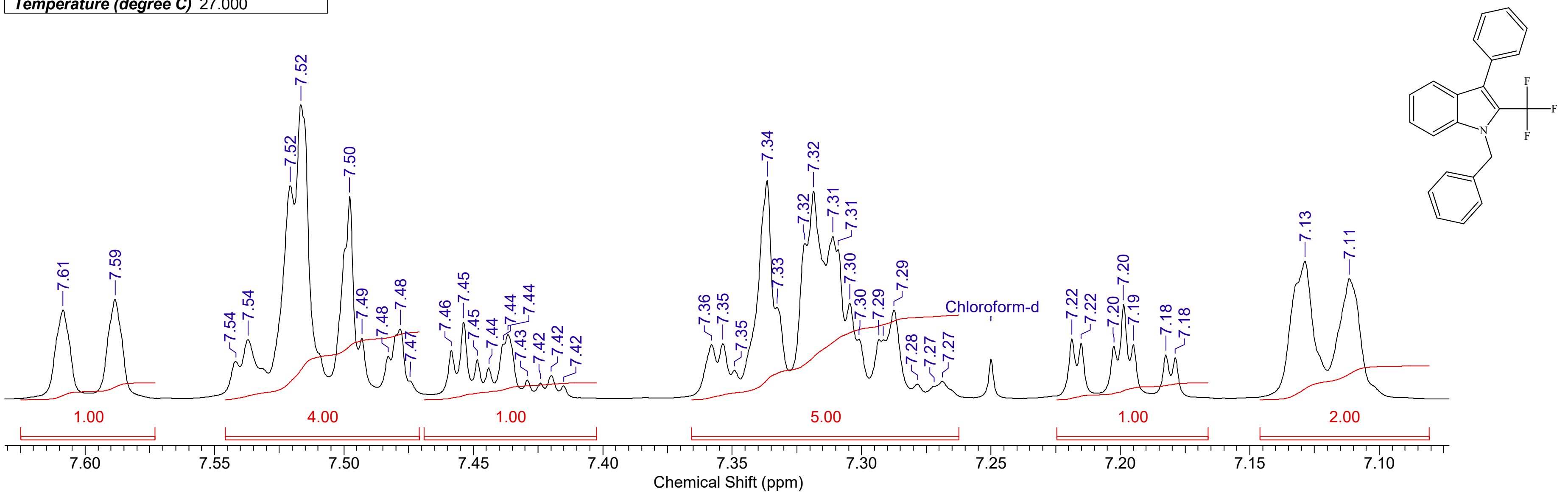

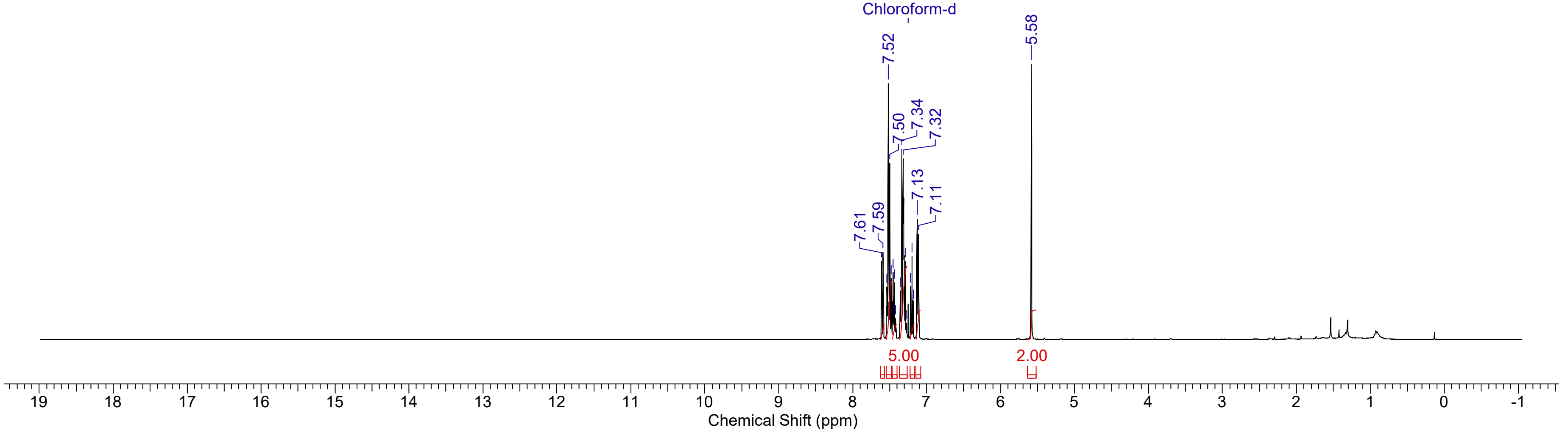

^{1}H NMR spectrum of **16** (400.1 MHz, $CDCl_3$)

Acquisition Time (sec)	1.7433	Comment	Imported from UXNMR.			Date	05 Oct 2022 17:19:14
File Name	C:\BM_DATA\DOCS\05.10.22\05.10.22\SZA-BM-2628-12.F_005001r					Frequency (MHz)	376.50
Nucleus	19F	Number of Transients	16	Original Points Count	131072	Points Count	262144
Pulse Sequence	zgflqn	Solvent	BENZENE-D6	Sweep Width (Hz)	75187.97	Temperature (degree C)	27.000

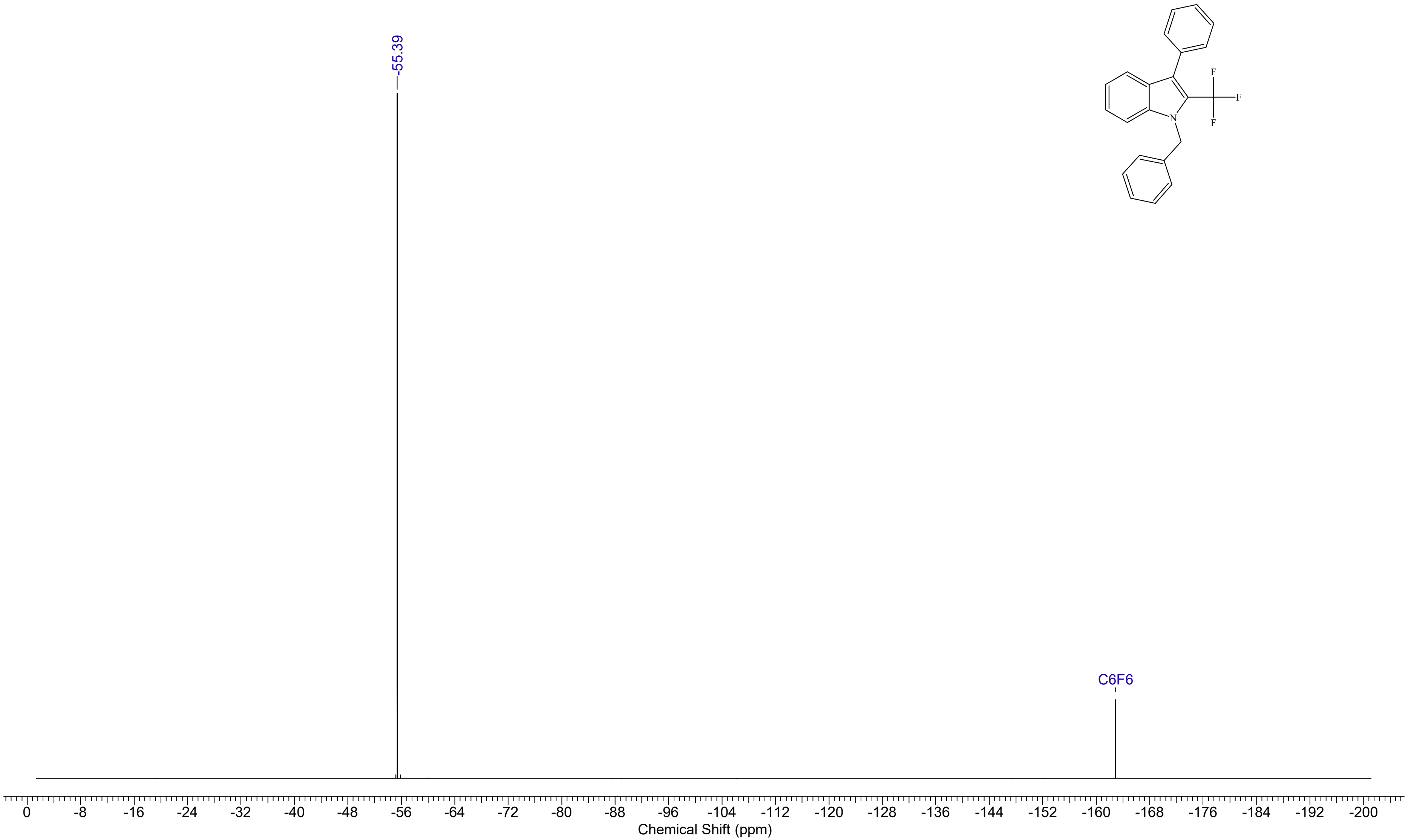

^{19}F NMR spectrum of **16** (376.5 MHz, $CDCl_3$)

6 Oct 2022

Acquisition Time (sec)	0.6783	***Comment***	Imported from UXNMR.			***Date***	06 Oct 2022 11:38:38
File Name	C:\BM_DATA\DOCS\06.10.22\06.10.22\SZA-BM-2628-12.C_002001r					***Frequency (MHz)***	100.61
Nucleus	13C	***Number of Transients***	361	***Original Points Count***	16384	***Points Count***	131072
Pulse Sequence	zgpg30	***Solvent***	CHLOROFORM-D			***Sweep Width (Hz)***	24154.59
Temperature (degree C)	27.000						

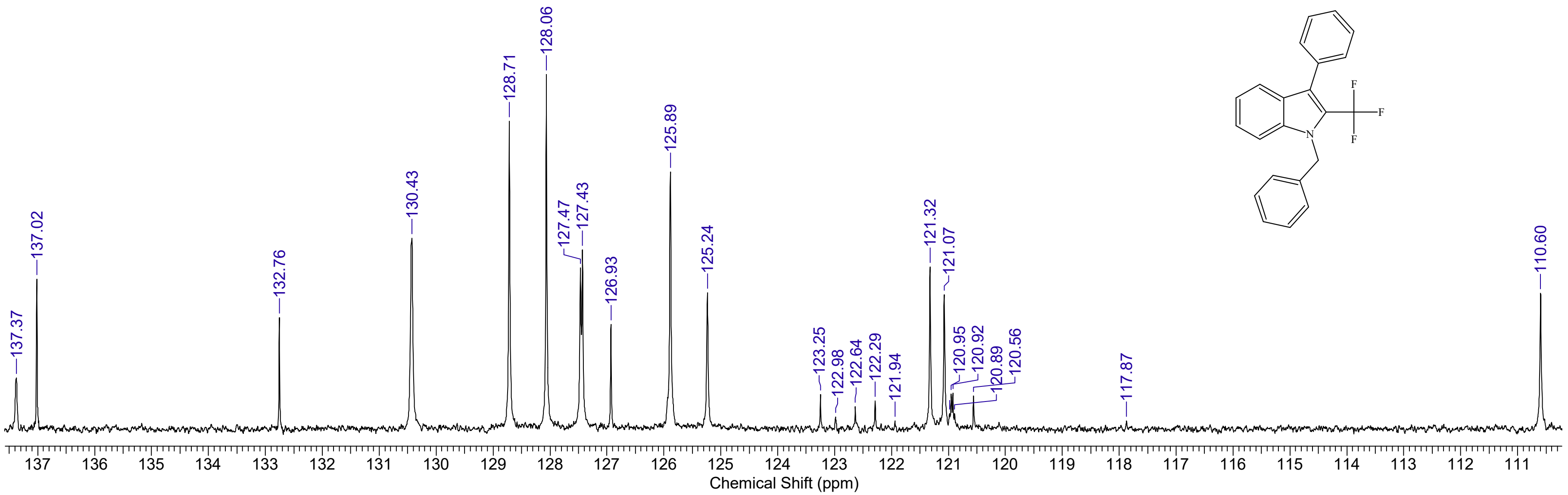

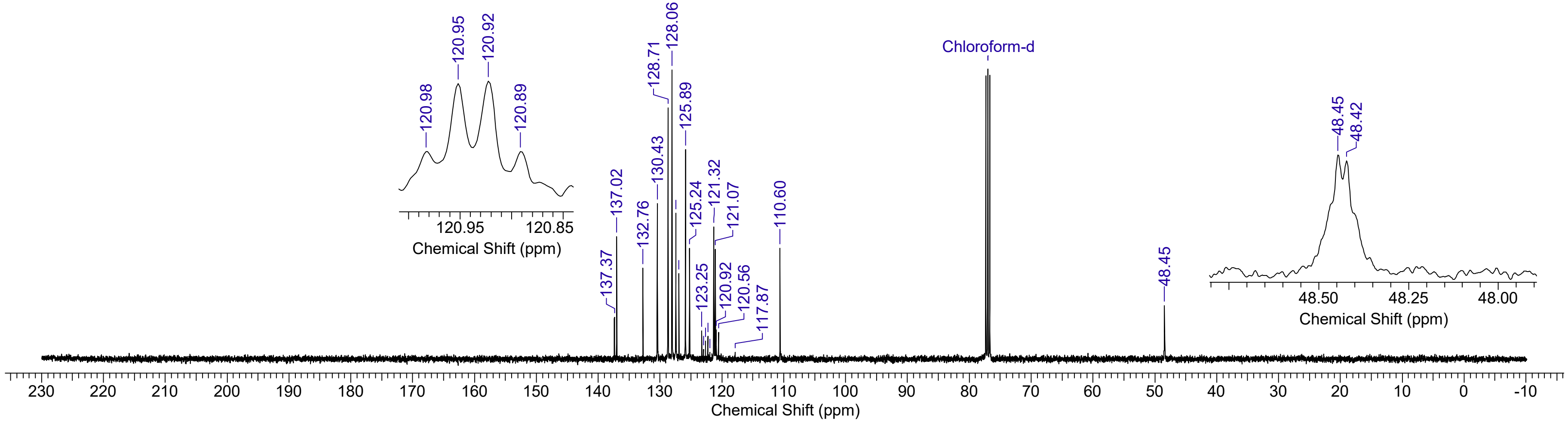

$^{13}C\{^{1}H\}$ NMR spectrum of **16** (100.6 MHz, $CDCl_3$)

6 Oct 2022

Acquisition Time (sec)	4.0894	***Comment***	Imported from UXNMR.	***Date***	27 Sep 2022 15:19:04
File Name	C:\DOCS\OUTPUT_301\2022\09.сентябрь\BM-2623-2.H_001001r			***Frequency (MHz)***	400.13
Nucleus	1H	***Number of Transients***	4 ***Original Points Count*** 32768	***Points Count***	131072
Pulse Sequence	zg30	***Solvent***	CHLOROFORM-D	***Sweep Width (Hz)***	8012.82
Temperature (degree C)	27.000				

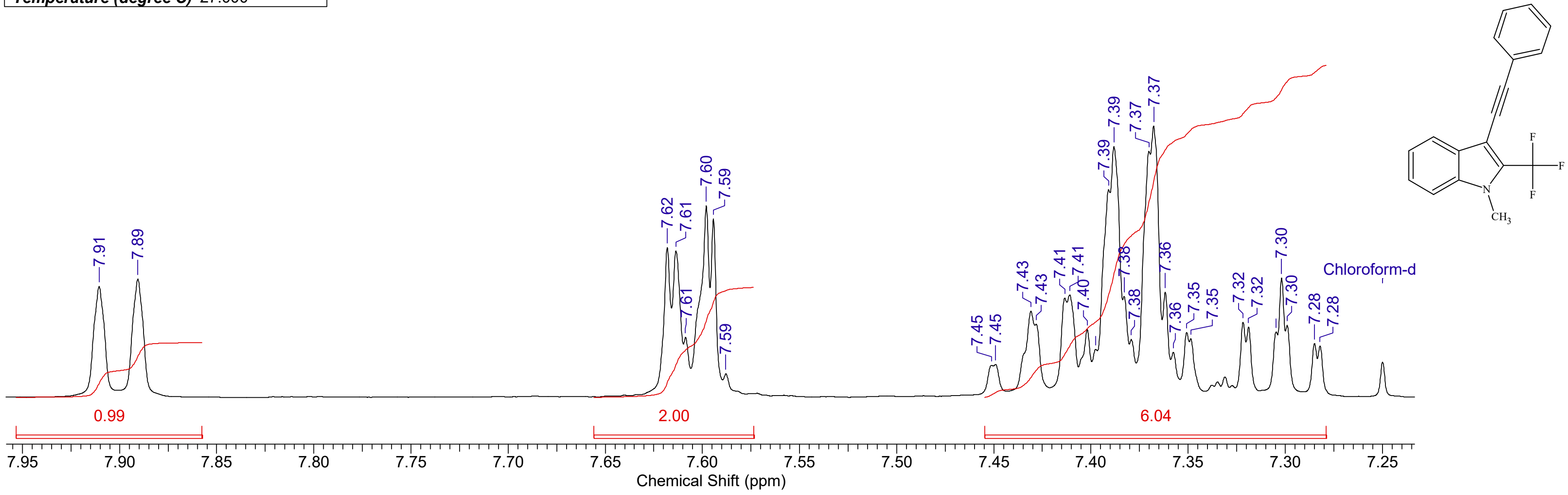

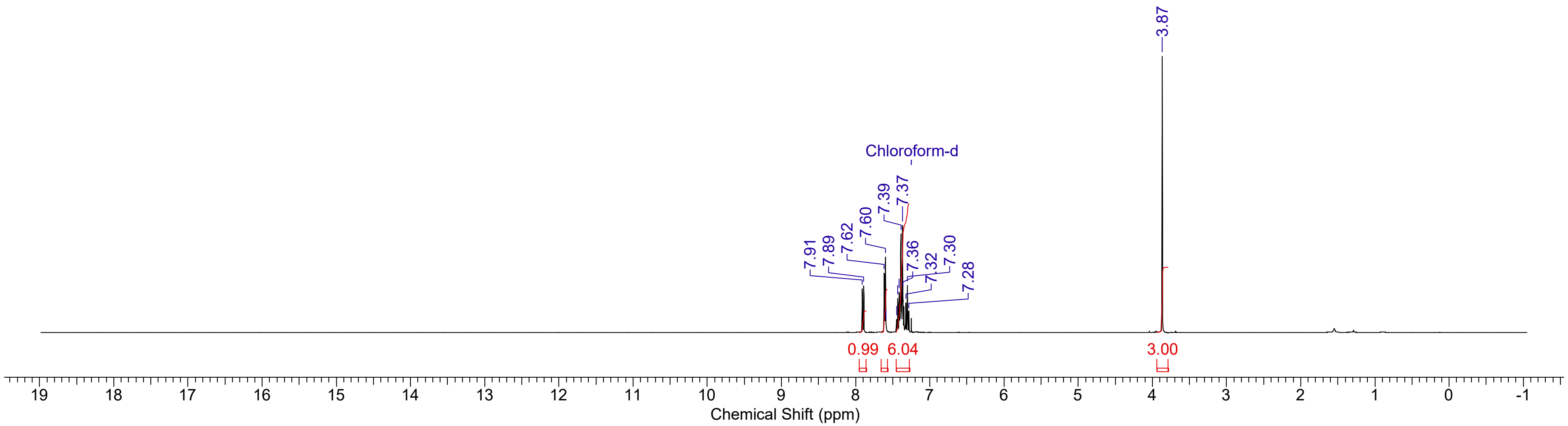

^{1}H NMR spectrum of **17** (400.1 MHz, $CDCl_3$)

6 Oct 2022

Acquisition Time (sec)	1.7433	***Comment***	Imported from UXNMR.			***Date***	27 Sep 2022 15:09:44
File Name	C:\DOCS\OUTPUT_301\2022\09.сентябрь\BM-2623-2.F_005001r					***Frequency (MHz)***	376.50
Nucleus	19F	***Number of Transients***	16	***Original Points Count***	131072	***Points Count***	262144
Pulse Sequence	zgflqn	***Solvent***	CHLOROFORM-D			***Sweep Width (Hz)***	75187.97
Temperature (degree C)	27.000						

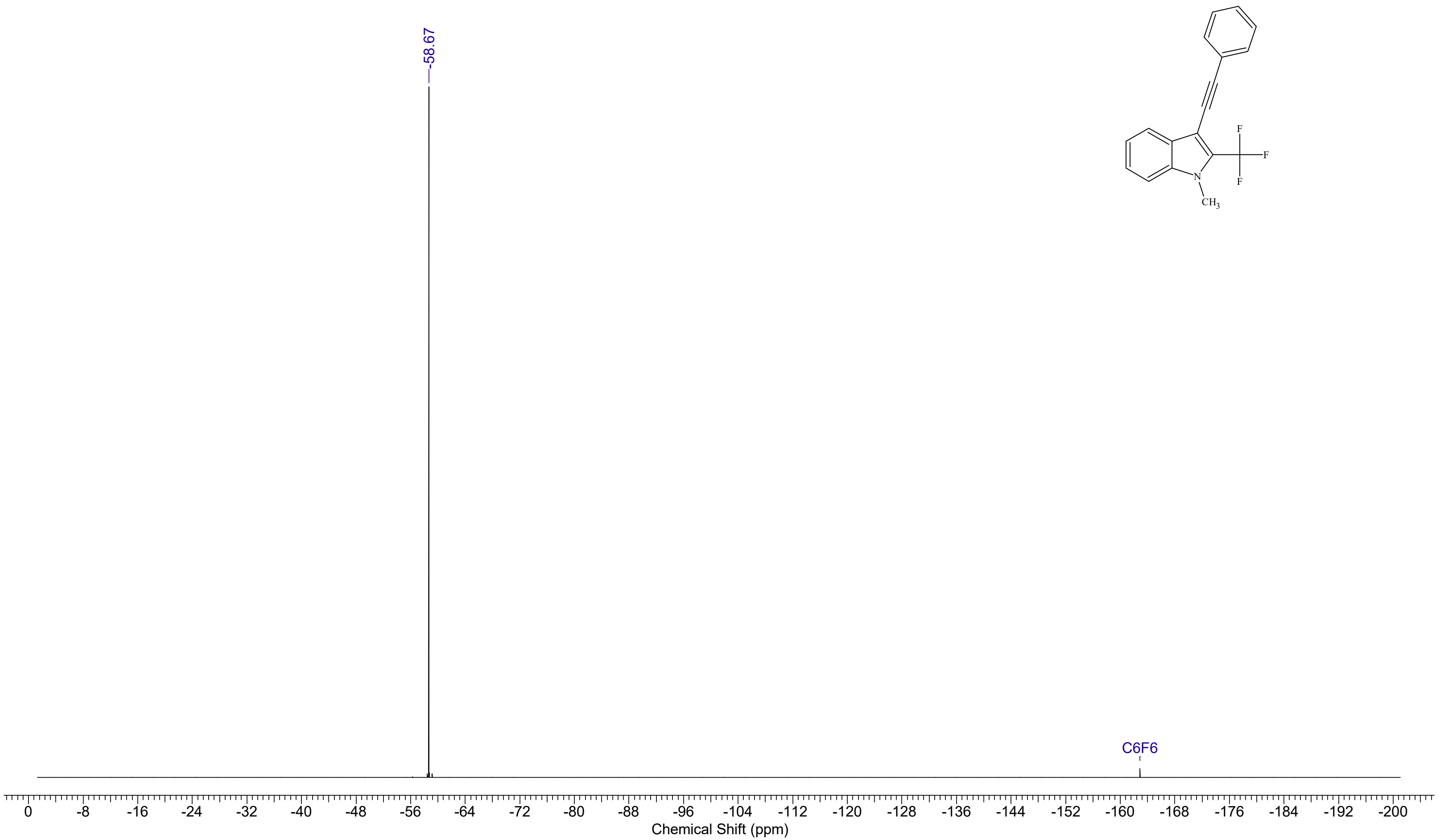

^{19}F NMR spectrum of **17** (376.5 MHz, $CDCl_3$)

6 Oct 2022

Acquisition Time (sec)	0.6783	***Comment***	Imported from UXNMR.	***Date***	28 Sep 2022 17:44:58
File Name	C:\DOCS\OUTPUT_301\2022\09.сентябрь\BM-2623-2.C_002001r			***Frequency (MHz)***	100.61
Nucleus	13C	***Number of Transients***	225 ***Original Points Count*** 16384	***Points Count***	131072
Pulse Sequence	zgpg30	***Solvent***	CHLOROFORM-D	***Sweep Width (Hz)***	24154.59
Temperature (degree C)	27.000				

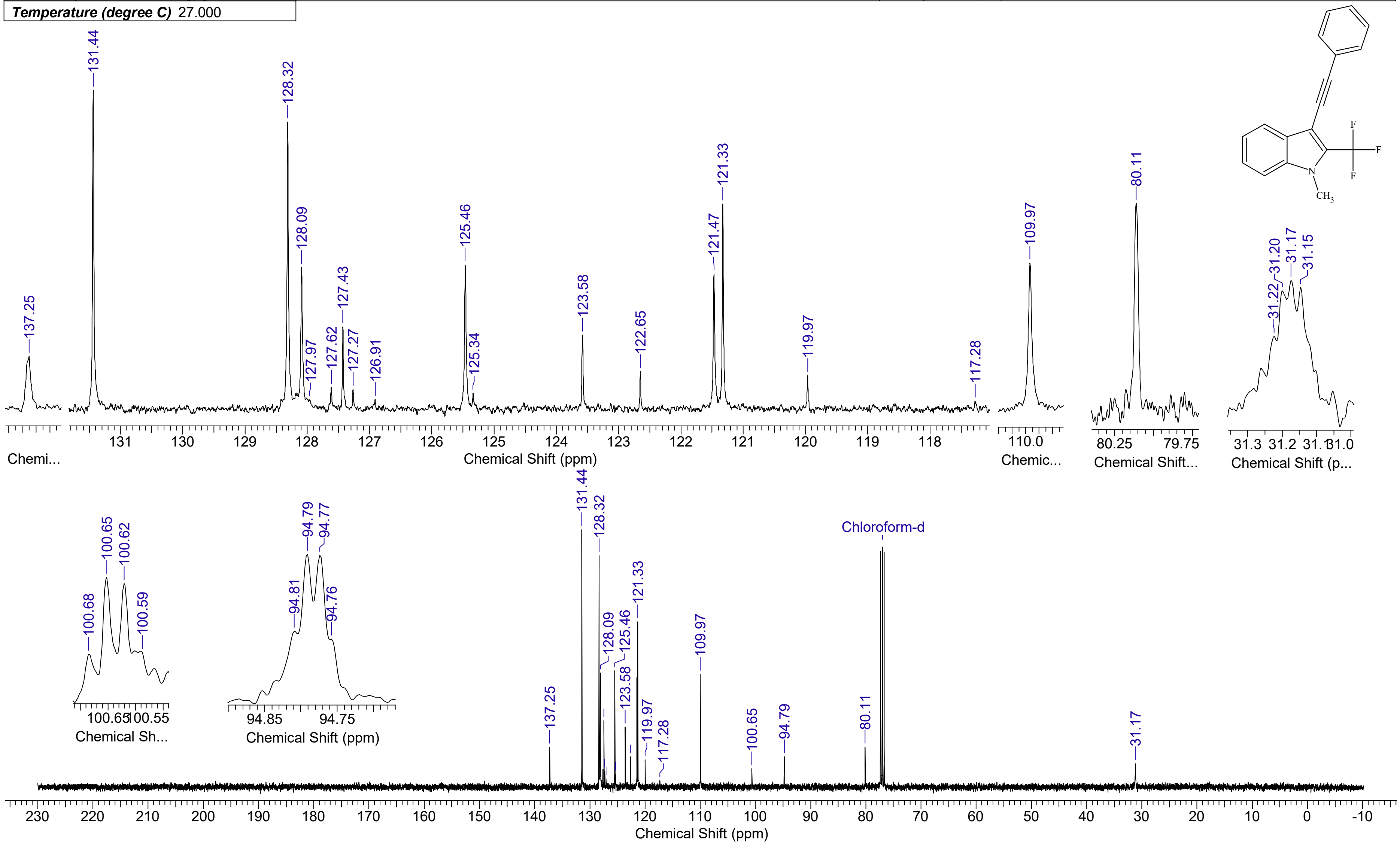

$^{13}C\{^1H\}$ NMR spectrum of **17** (100.6 MHz, $CDCl_3$)

6 Oct 2022

Acquisition Time (sec)	4.0894	***Comment***	Imported from UXNMR.			***Date***	30 Sep 2022 15:16:12
File Name	C:\DOCS\OUTPUT_301\2022\09.сентябрь\SZA-BM-2629-9.H_001001r					***Frequency (MHz)***	400.13
Nucleus	1H	***Number of Transients***	4	***Original Points Count***	32768	***Points Count***	131072
Pulse Sequence	zg30	***Solvent***	CHLOROFORM-D			***Sweep Width (Hz)***	8012.82
Temperature (degree C)	27.000						

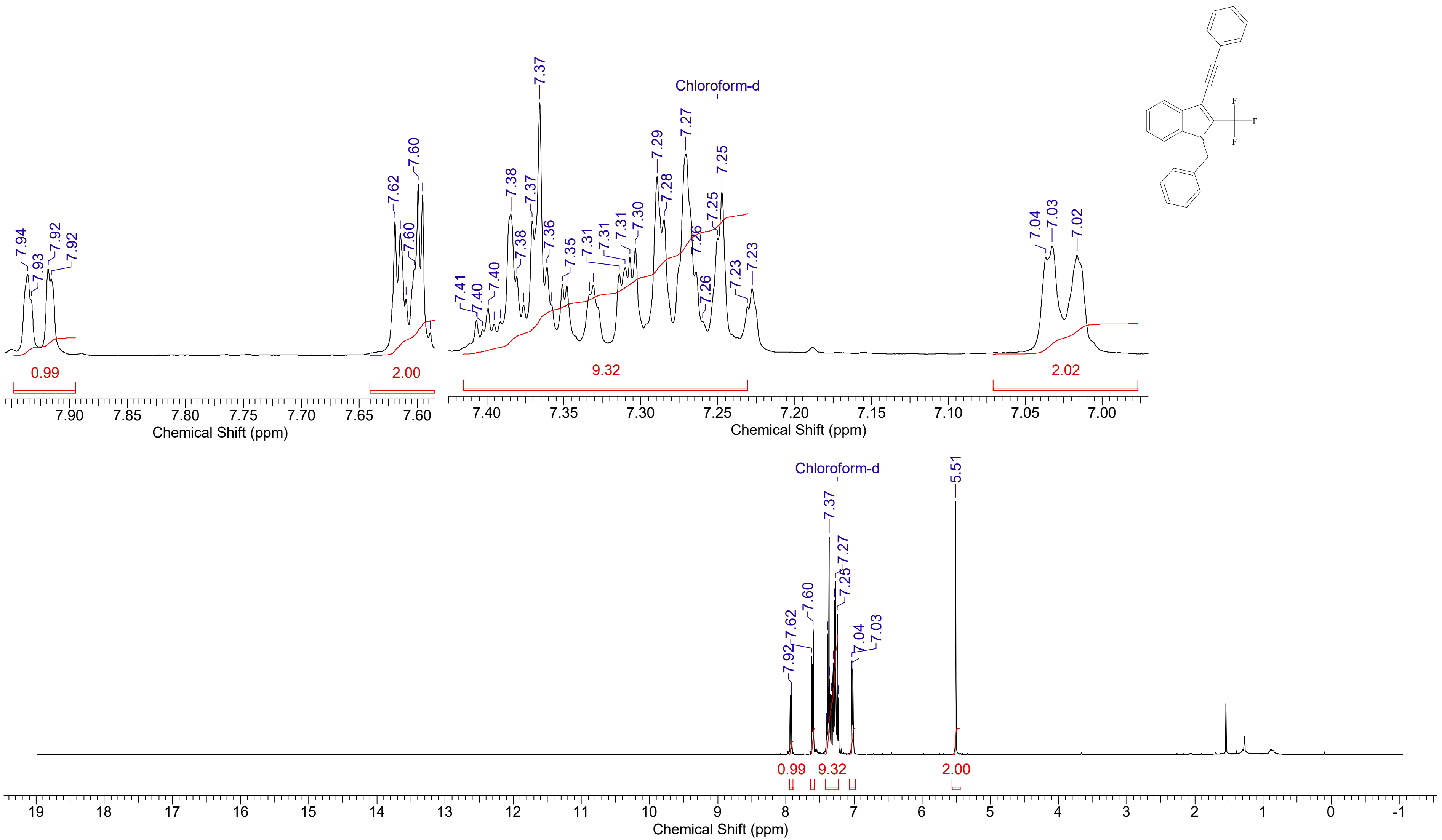

^{1}H NMR spectrum of **18** (400.1 MHz, $CDCl_3$)

6 Oct 2022

Acquisition Time (sec)	1.7433	***Comment***	Imported from UXNMR.		***Date***	22 Sep 2022 12:38:48
File Name	C:\DOCS\OUTPUT_301\2022\09.сентябрь\BM-2593-2.F_005001r				***Frequency (MHz)***	376.50
Nucleus	19F	***Number of Transients***	16	***Original Points Count*** 131072	***Points Count***	262144
Pulse Sequence	zgflqn	***Solvent***	CHLOROFORM-D		***Sweep Width (Hz)***	75187.97
Temperature (degree C)	27.000					

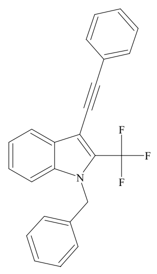

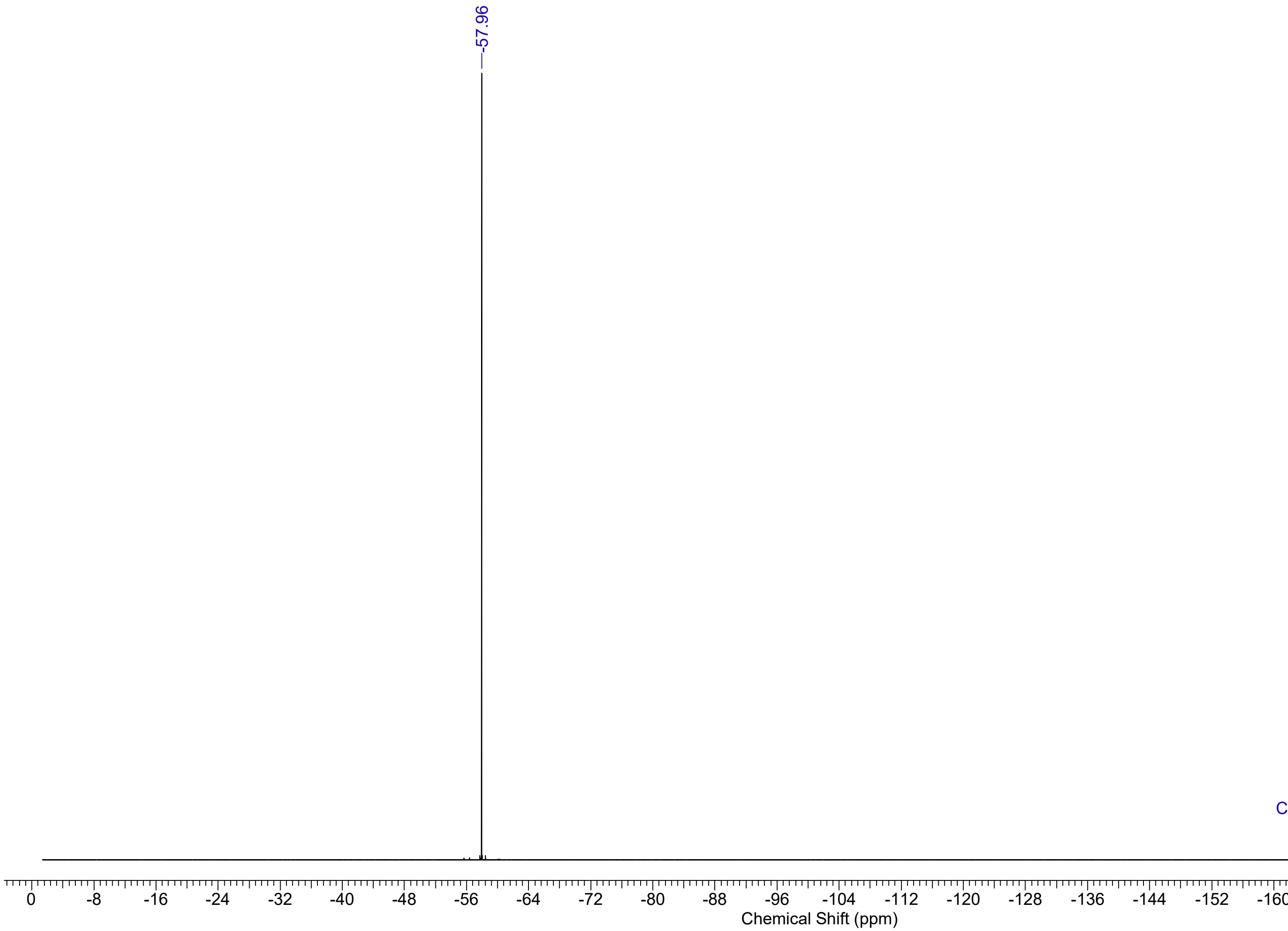

^{19}F NMR spectrum of **18** (376.5 MHz, $CDCl_3$)

6 Oct 2022

Acquisition Time (sec)	0.6783	***Comment***	Imported from UXNMR.			***Date***	06 Oct 2022 12:05:28
File Name	C:\BM_DATA\DOCS\06.10.22\06.10.22\SZA-BM-2629-9.C_002001r					***Frequency (MHz)***	100.61
Nucleus	13C	***Number of Transients***	361	***Original Points Count***	16384	***Points Count***	131072
Pulse Sequence	zgpg30	***Solvent***	CHLOROFORM-D			***Sweep Width (Hz)***	24154.59
Temperature (degree C)	27.000						

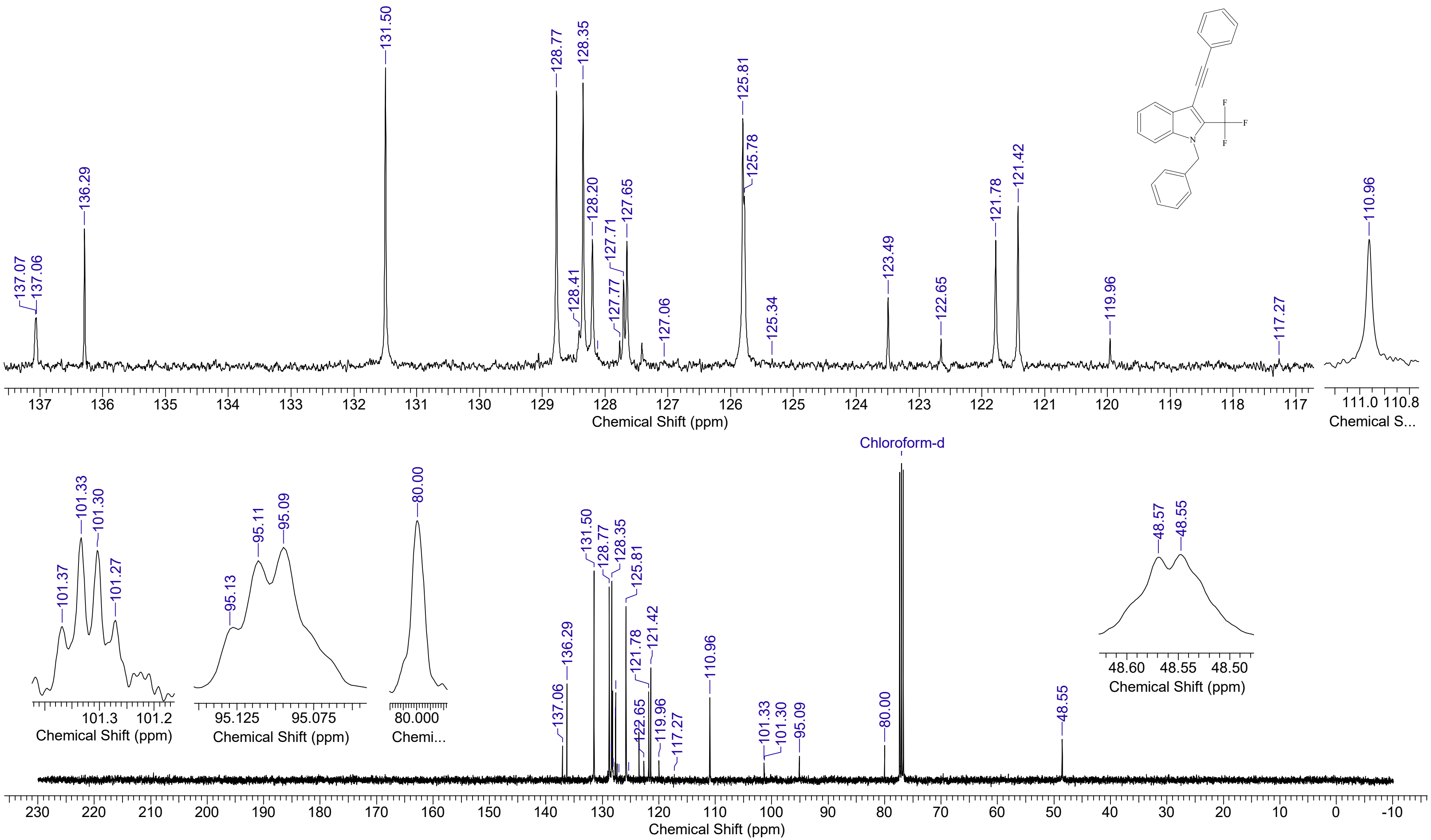

$^{13}C\{^{1}H\}$ NMR spectrum of **18** (100.6 MHz, $CDCl_3$)

6 Oct 2022

Acquisition Time (sec)	4.0894	***Comment***	Imported from UXNMR.			***Date***	04 Oct 2022 14:39:26
File Name	C:\DOCS\OUTPUT_301\2022\10.октябрь\BM-2644-2p.H.H_001001r					***Frequency (MHz)***	400.13
Nucleus	1H	***Number of Transients***	4	***Original Points Count***	32768	***Points Count***	131072
Pulse Sequence	zg30	***Solvent***	CHLOROFORM-D			***Sweep Width (Hz)***	8012.82
Temperature (degree C)	27.000						

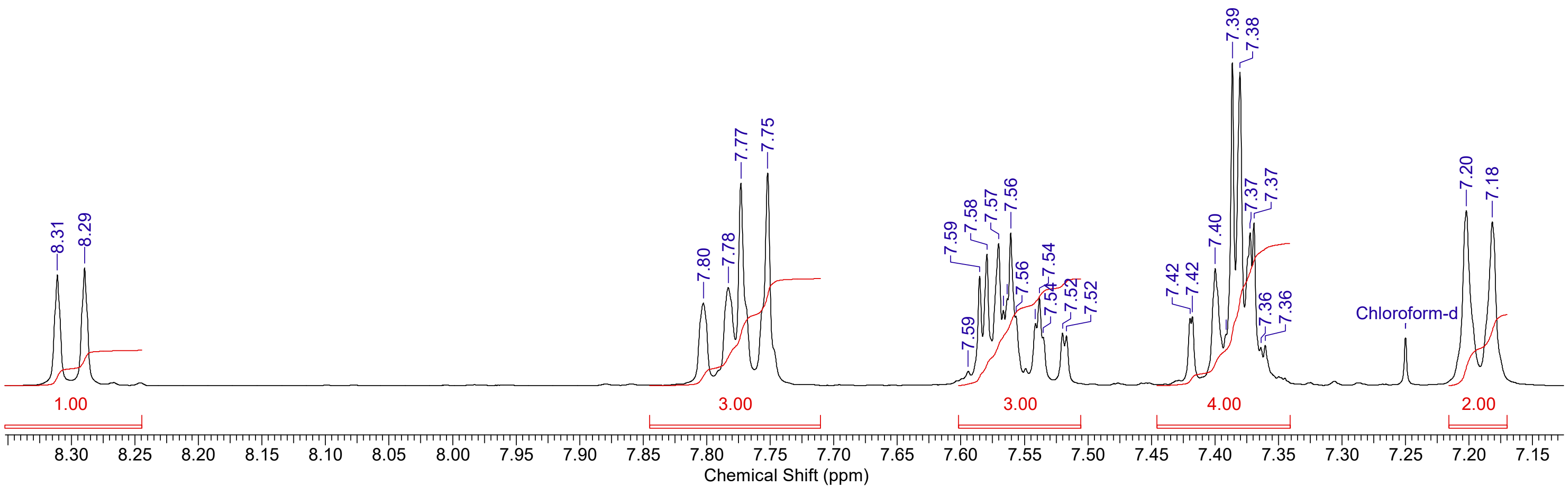

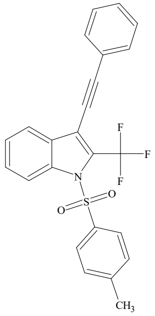

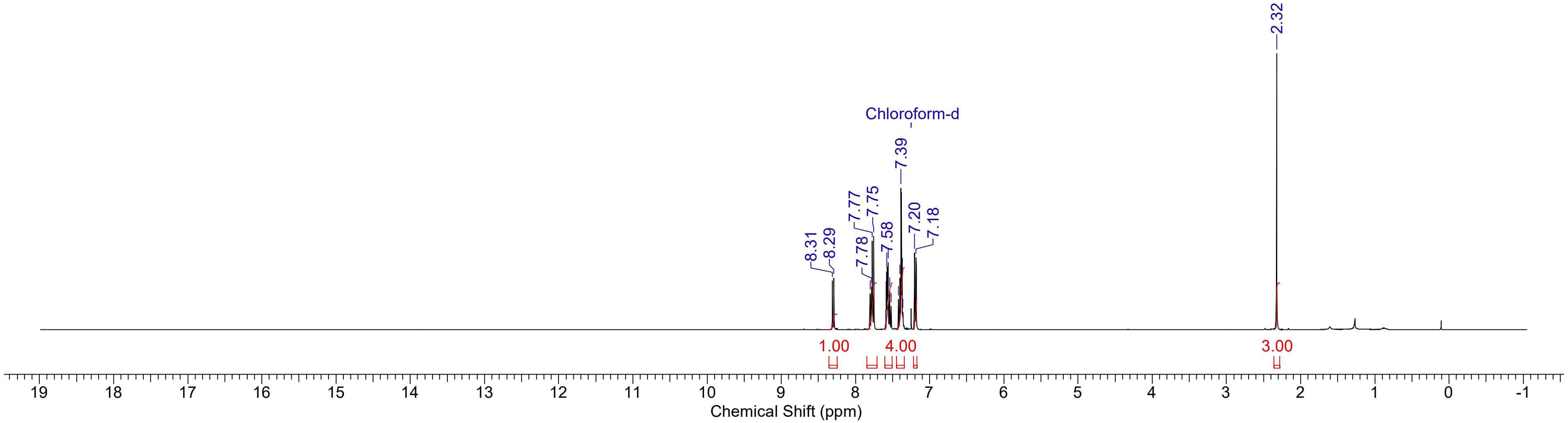

^{1}H NMR spectrum of **19** (400.1 MHz, $CDCl_3$)

6 Oct 2022

Acquisition Time (sec)	1.7433	***Comment***	Imported from UXNMR.		***Date***	04 Oct 2022 15:36:38
File Name	C:\DOCS\OUTPUT_301\2022\10.октябрь\BM-2644-2p.F_005001r				***Frequency (MHz)***	376.50
Nucleus	19F	***Number of Transients***	11	***Original Points Count*** 131072	***Points Count***	262144
Pulse Sequence	zgflqn	***Solvent***	CHLOROFORM-D		***Sweep Width (Hz)***	75187.97
Temperature (degree C)	27.000					

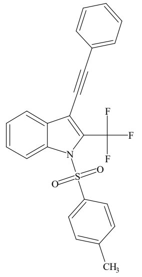

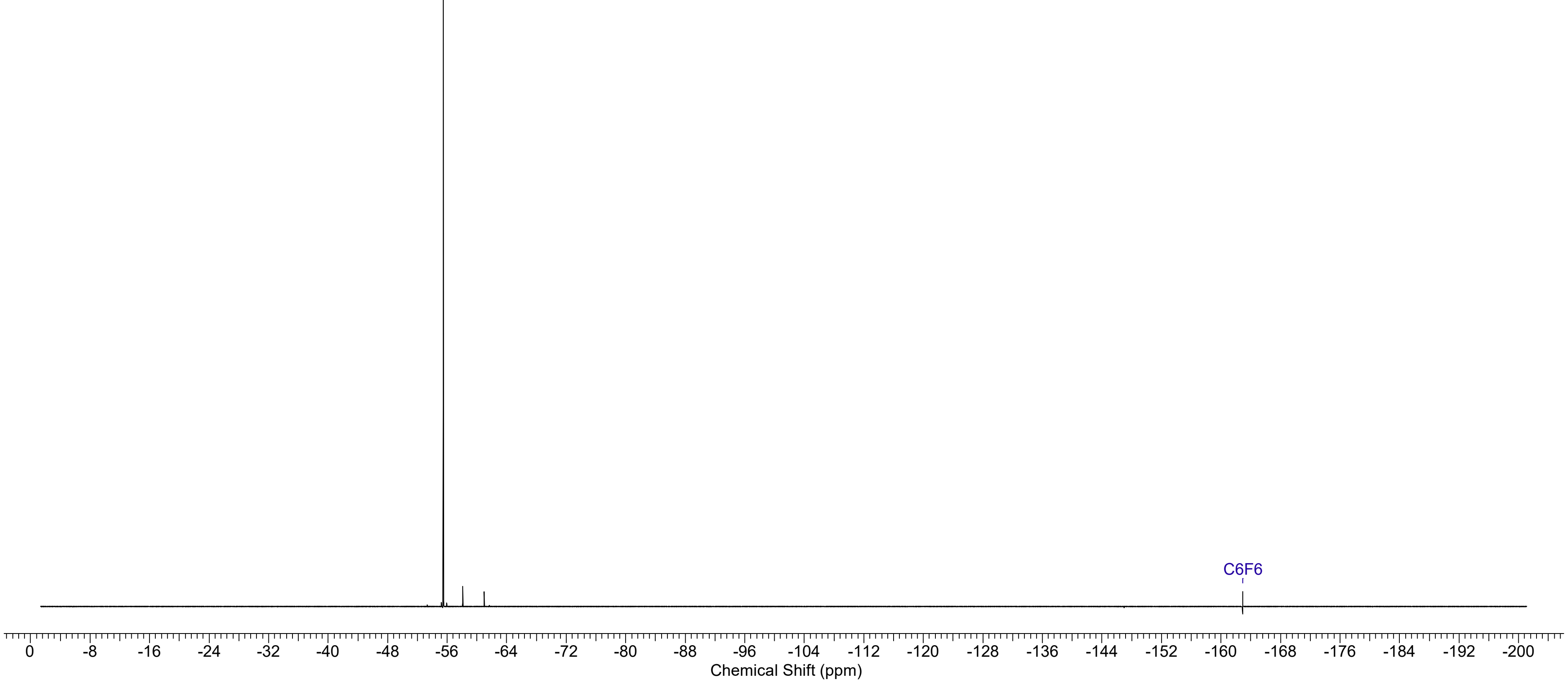

^{19}F NMR spectrum of **19** (376.5 MHz, $CDCl_3$)

6 Oct 2022

Acquisition Time (sec)	0.6783	***Comment***	Imported from UXNMR.			***Date***	04 Oct 2022 14:46:06
File Name	C:\DOCS\OUTPUT_301\2022\10.октябрь\BM-2644-2p.C_002001r					***Frequency (MHz)***	100.61
Nucleus	13C	***Number of Transients***	137	***Original Points Count***	16384	***Points Count***	131072
Pulse Sequence	zgpg30	***Solvent***	CHLOROFORM-D			***Sweep Width (Hz)***	24154.59
Temperature (degree C)	27.000						

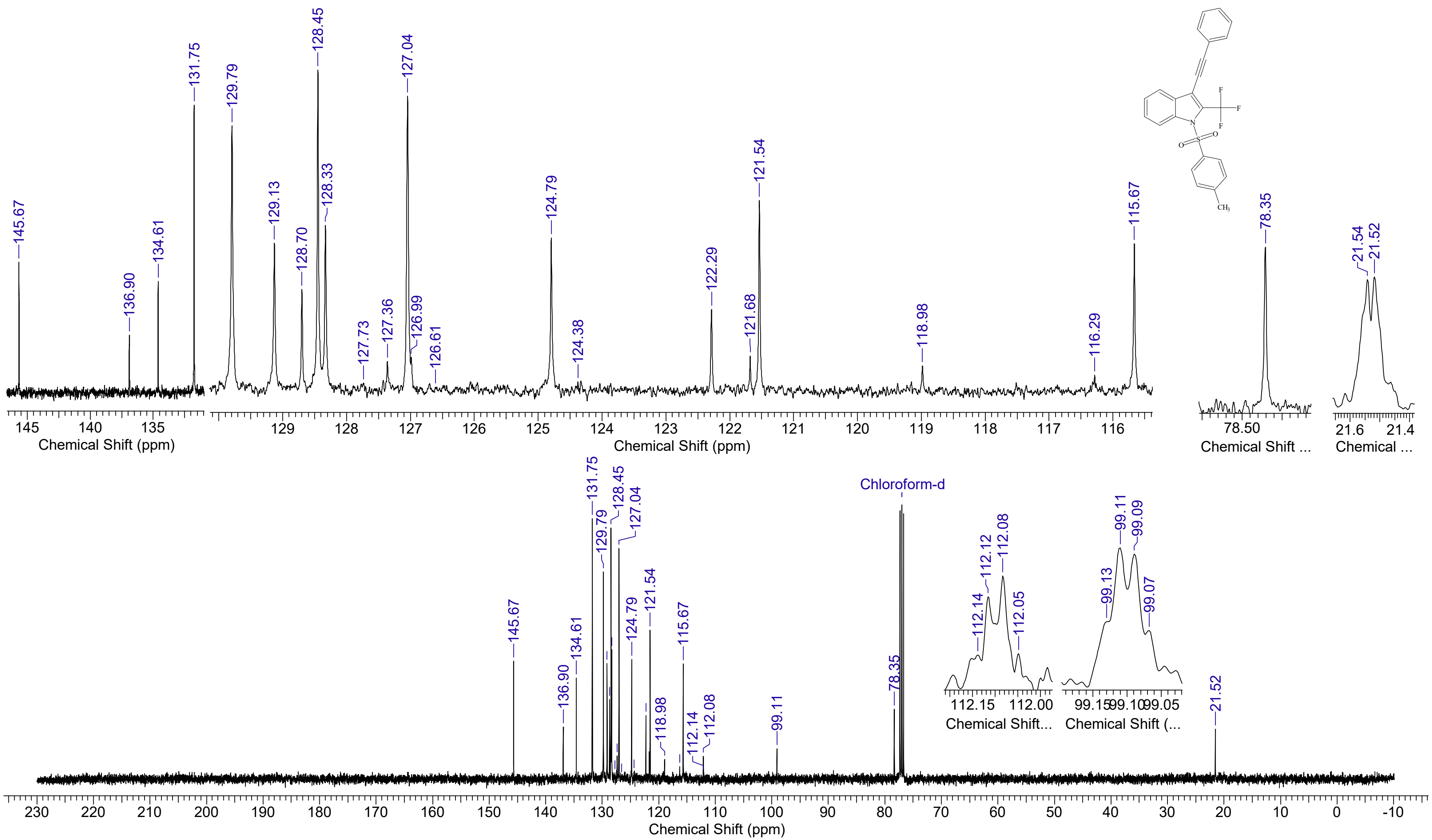

$^{13}C\{^1H\}$ NMR spectrum of **19** (100.6 MHz, $CDCl_3$)
